# Nonretinoid chaperones improve rhodopsin homeostasis in a mouse model of retinitis pigmentosa

**DOI:** 10.1172/jci.insight.153717

**Published:** 2022-05-23

**Authors:** Abhishek Vats, Yibo Xi, Bing Feng, Owen D. Clinger, Anthony J. St. Leger, Xujie Liu, Archisha Ghosh, Chase D. Dermond, Kira L. Lathrop, Gregory P. Tochtrop, Serge Picaud, Yuanyuan Chen

**Affiliations:** 1 Department of Ophthalmology,; 2 Department of Pharmacology and Chemical Biology,; 3 McGowan Institute of Regenerative Medicine, and; 4 Department of Immunology, University of Pittsburgh, Pittsburgh, Pennsylvania, USA.; 5 Department of Bioengineering, University of Pittsburgh Swanson School of Engineering, Pittsburgh, Pennsylvania, USA.; 6 Department of Chemistry, Case Western Reserve University, Cleveland, Ohio, USA.; 7 Sorbonne Université, INSERM, CNRS, Institut de la Vision, Paris, France.

**Keywords:** Neuroscience, Ophthalmology, G protein&ndash;coupled receptors, Pharmacology, Protein misfolding

## Abstract

*Rhodopsin*-associated (*RHO*-associated) retinitis pigmentosa (RP) is a progressive retinal disease that currently has no cure. RHO protein misfolding leads to disturbed proteostasis and the death of rod photoreceptors, resulting in decreased vision. We previously identified nonretinoid chaperones of RHO, including YC-001 and F5257-0462, by small-molecule high-throughput screening. Here, we profile the chaperone activities of these molecules toward the cell-surface level of 27 RP-causing human RHO mutants in NIH3T3 cells. Furthermore, using retinal explant culture, we show that YC-001 improves retinal proteostasis by supporting RHO homeostasis in *Rho^P23H/+^* mouse retinae, which results in thicker outer nuclear layers (ONL), indicating delayed photoreceptor degeneration. Interestingly, YC-001 ameliorated retinal immune responses and reduced the number of microglia/macrophages in the *Rho^P23H/+^* retinal explants. Similarly, F5257-0462 also protects photoreceptors in *Rho^P23H/+^* retinal explants. In vivo, intravitreal injection of YC-001 or F5257-0462 microparticles in PBS shows that F5257-0462 has a higher efficacy in preserving photoreceptor function and delaying photoreceptor death in *Rho^P23H/+^* mice. Collectively, we provide proof of principle that nonretinoid chaperones are promising drug candidates in treating *RHO*-associated RP.

## Graphical abstract

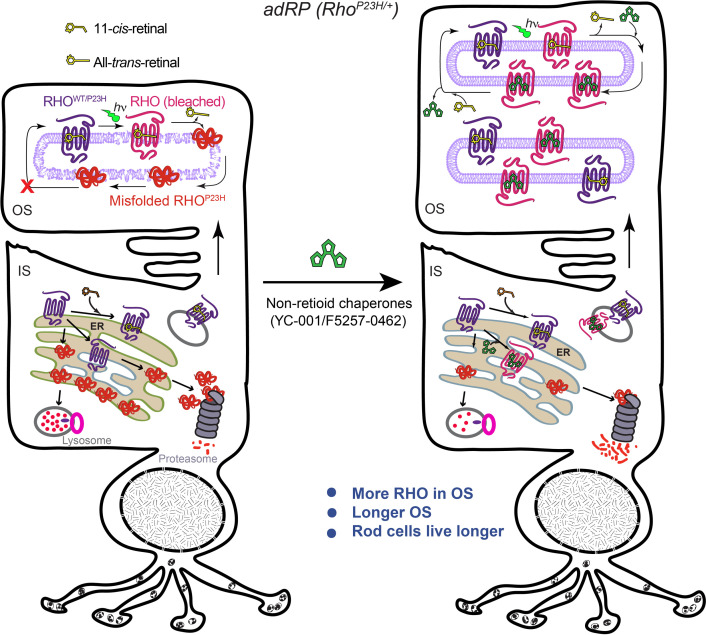

## Introduction

Misfolded proteins are involved in the etiology of many diseases, including neural degenerations (1, 2), lysosome storage disorders (3), cystic fibrosis (4), and type II diabetes (5), as well as visual disorders including retinitis pigmentosa (RP; ref. 6), a progressive retinal degeneration due to loss of rod photoreceptors. *RHODOPSIN* (*RHO*) gene mutations account for about 25% of all autosomal dominant RP (adRP; ref. 6). Although gene therapies have been successful in treating Leber congenital amaurosis (7–10) and show promise for treating some genotypes of RP, no pharmacological treatments are available for RP.

RHO is the visual pigment protein located in rod outer segments (OS; ref. 11). As the most abundant protein in rod cells, RHO misfolding caused by genetic mutation leads to a tremendous load on proteostasis (12, 13). More than 150 mutations have been identified in the *RHO* gene that causes adRP, and only 4 mutations cause autosomal recessive RP (http://www.hgmd.cf.ac.uk). These mutations are grouped into classes according to their biochemical characteristics; among them, class II mutations affect the structural stability of RHO protein and are most numerous. Notably, RHO protein misfolding leads to dominant-negative effects, which include: (a) coaggregation of WT and mutant RHO in the endoplasmic reticulum (ER; refs. 14, 15) leading to their degradation and shorter OS, (b) activated ER stress (16–18), and (c) a chronically overwhelmed proteolytic system (19, 20). These factors are considered to contribute to the death of rod cells carrying class II *RHO* mutations.

Using gene delivery to treat *RHO*-associated adRP is challenging, as it requires the effective knockdown of the mutant gene while maintaining high expression of the healthy gene in rod cells to preserve vision (21). Alternatively, pharmacological chaperone therapy can be a viable strategy to rescue rod cells by stabilizing the mutant RHO protein. The native ligand, 11-*cis*-retinal, is an endogenous chaperone because the chromophore-bound RHO pigment is more stable at its inactive state in the dark than the apo-opsin. The transport of RHO mutants can also be rescued to the plasma membrane in cells treated with 11-*cis*-retinal or 9-*cis*-retinal (11, 15, 22). While vitamin A supplementation was reported to show clinical benefit among some RP patients (23, 24), the lack of genetic information on these patients makes it unclear if patients who responded to the treatment carry *RHO* mutations. One limitation of 11-*cis*-retinal as a chaperone lies in its photosensitivity; once photoisomerized to all-*trans* conformation, the chromophore is released from RHO to form apo-opsin (25) that is less stable in the OS. Thus, a nonretinoid pharmacological chaperone is necessary to provide additional structural support for mutant apo-opsin during RHO biosynthesis and after photobleaching.

We discovered a group of pharmacological chaperones of RHO, including nonretinoids YC-001 and F5257-0462 (26), by a cell-based high-throughput screening (HTS; refs. 27, 28). Notably, YC-001 mentioned in this study is not the soluble guanylyl cyclase activator YC-1 (29). YC-001 binds to rod opsin competitively with 9-*cis*-retinal chromophore and stabilizes rod opsin. Both YC-001 and F5257-0462 improve the glycosylation profile of RHO^P23H^ and rescue multiple misfolded RHO mutants from ER to the plasma membrane, demonstrating the potent pharmacological chaperone activity of these compounds. Furthermore, we found YC-001 protects the retinae from light damage in *Abca4^–/–^, Rdh8^–/–^* mice (26), an acute retinal degeneration model. However, the efficacy of these pharmacological chaperones in retina expressing misfolded RHO was not known. In this study, we test the efficacy of YC-001 and F5257-0462 in the retinae of *Rho^P23H/+^*–knock-in mice in explant culture and in vivo, and we use biochemical assays and RNA-Seq to elucidate the mechanism of actions and safety of these compounds.

## Results

### YC-001 and F5257-0462 rescue multiple adRP-causing RHO mutants.

**Figure 1:**
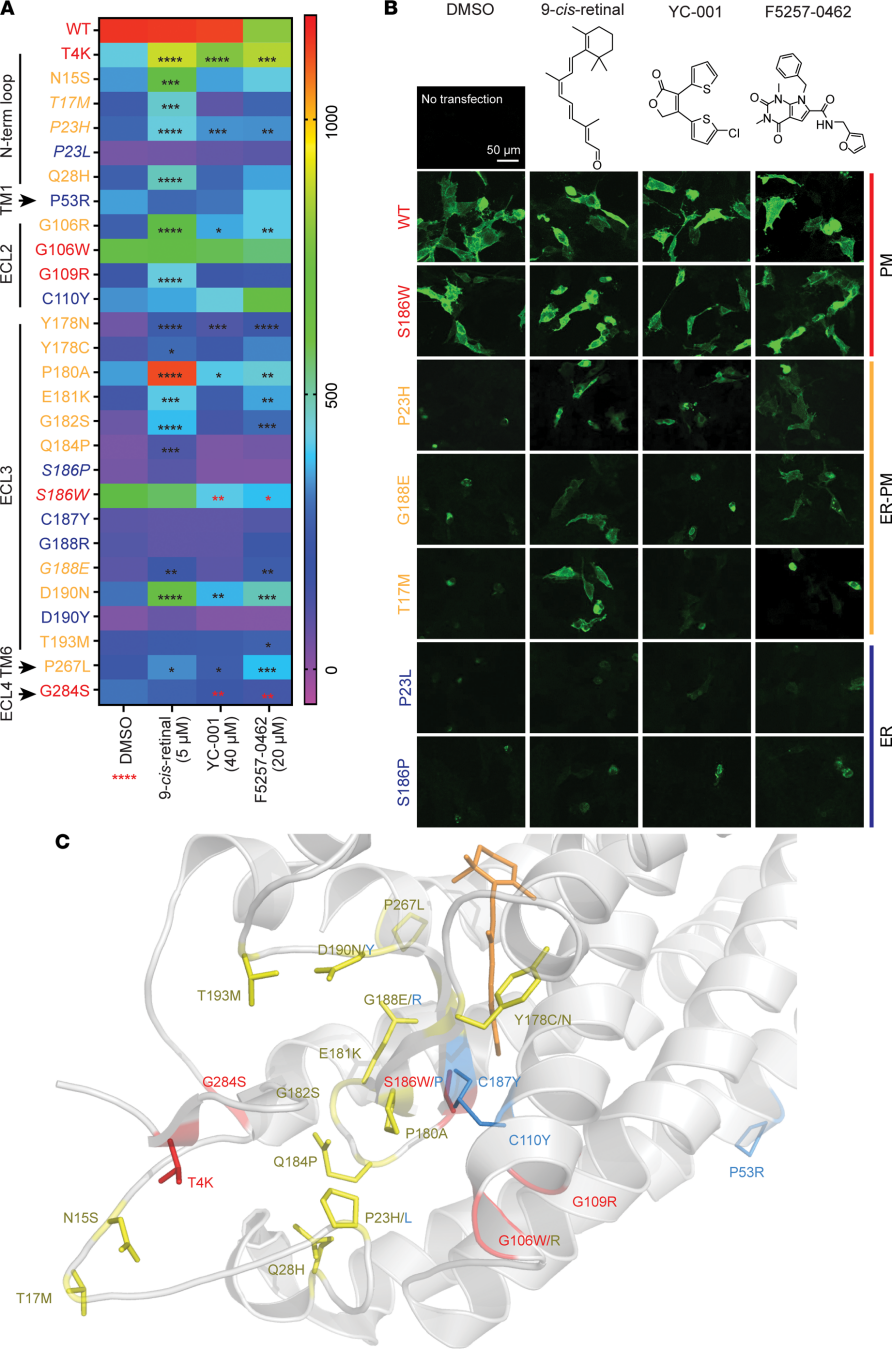
Pharmacological chaperones rescue numerous rhodopsin (RHO) mutants trafficking to the plasma membrane in vitro. NIH3T3 cells transfected with hRHO mutants were treated with either DMSO as vehicle control, 5 μM 9-*cis*-retinal, 40 μM YC-001, or 20 μM F5257-0462 for 24 hours, and high-content images were taken for RHO immunofluorescence on the cell surface. (**A**) Heatmap of cell surface immunofluorescence intensity of WT and 27 RHO mutants per cell under different treatments. *n =* 3, red **** under DMSO, *P* < 0.0001 comparing any mutants versus RHO^WT^ with DMSO by 1-way ANOVA. In the heatmap, *, **, ***, and **** signify *P* < 0.05, 0.01, 0.001, and 0.0001, respectively, comparing compound treated versus DMSO by the Kruskal-Wallis test followed by a multiple comparison test. Black and red asterisks represent significantly higher or lower levels, respectively, in compound treated versus DMSO. WT and RHO mutants that were seen on the plasma membrane under all conditions are marked in red (PM); mutants unseen on the cell surface under any condition are marked in blue (ER); and mutants absent on the cell surface under DMSO treatment but rescued to the plasma membrane by at least 1 pharmacological chaperone are marked in yellow (ER-PM). N-term loop, N-terminal loop; ECL, extracellular loop; TM, transmembrane helix. (**B**) Representative immunofluorescence cell-surface staining of WT and mutant RHO (italics in **A**). Scale bar: 50 μm. Chemical structures are shown on the top. All images are shown in Supplemental Figure 1. (**C**) Residues that were mutated are color labeled on the crystal structure of bovine RHO (PDB ID:1f88). Orange sticks, 11-*cis*-retinal.

A pharmacological profile of adRP causing RHO mutants to chaperones is essential to identify pharmacological chaperone-responding RP patients. We developed a high-content imaging method (30) to quantify the amount of human RHO (hRHO) transported to the plasma membrane, a marker of properly folded RHO protein. We transfected 27 adRP-causing hRHO mutants to NIH3T3 cells and imaged their cell surface levels. Except for P53R and P267L as outlier controls located in the transmembrane helixes 1 and 6, respectively (11), the rest of these class II mutants (14) (http://www.hgmd.cf.ac.uk) have the mutated residues located on the extracellular/intradiscal side of the chromophore pocket of RHO. We quantified the effects of 3 pharmacological chaperones (9-*cis*-retinal, YC-001, and F5257-0462) to the level of each RHO mutant on the cell surface, using dimethyl sulfoxide (DMSO) as vehicle control (Figure 1, Supplemental Figures 1 and 2, and Supplemental Data 1; supplemental material available online with this article; https://doi.org/10.1172/jci.insight.153717DS1). RHO mutants in DMSO-treated cells displayed a wide range of cell surface levels (Figure 1A), indicating heterogeneous structural stabilities of these mutants. We categorized these mutants into 3 groups (Figure 1, A and B, and Supplemental Figure 1): (a) 5 mutants detectable on the cell surface without pharmacological chaperones (T4K, G106W, G109R, S186W, and G284S), indicating highest structural stability; (b) 15 mutants accumulated in the ER but rescued to the plasma membrane by at least 1 pharmacological chaperone (**N15S**, **T17M**, P23H, **Q28H**, G106R, Y178N, **Y178C**, P180A, E181K, G182S, **Q184P**, G188E, D190N, T193M, and P267L; bold marked mutants are only rescued by 9-*cis*-retinal), suggesting mild structural instability; and (c) 7 mutants with severe folding problems and not rescued by any tested pharmacological chaperones (P23L, C110Y, P53R, S186P, C187Y, G188R, and D190Y), showing lowest structural stability. Evaluating these mutations in a structural context (Figure 1C), we found that group A mutant residues are located at regions where the side chains of the affected residues are facing toward open space and hydrophobicity of mutated residues are compatible to the surrounding environment; however, the group c mutant residues incapable of rescue by any pharmacological chaperone led to major structural disturbance by disrupting the disulfide bond that stabilizes the 7-helix scaffold of RHO (C110Y and C187Y), causing spatial or charge clash (P23L, P53R, G188R, and D190Y) or changing the backbone angle (S186P). These results demonstrate that the effect of mutations on the stability of rod opsin structure ranges from mild to severe and can be quantitatively reflected by the cell surface levels of RHO and their sensitivity to pharmacological chaperones. We found 9-*cis*-retinal, F5257-0462, and YC-001 rescued cell surface transport of 16, 11, and 7 mutants, respectively (Figure 1A). Using sensitivity to chaperone rescue as an indicator of structural stability of these mutants, our results show that stabilities of N15S, T17M, Q28H, Y178C, and Q184P (rescued by 9-*cis*-retinal only) are lower than G188E (rescued by 9-*cis*-retinal and F5257-0462), which are less stable than T4K, P23H, G106R, Y178N, P180A, E181K, G182S, D190N, and P267L (rescued by all 3 compounds). T4K and T17M mutants have been reported to lack an ER-based misfolding phenotype (31, 32); however, our results suggest that T4K (group A mutant) is more stable than T17M (group B mutant).

**Figure 2:**
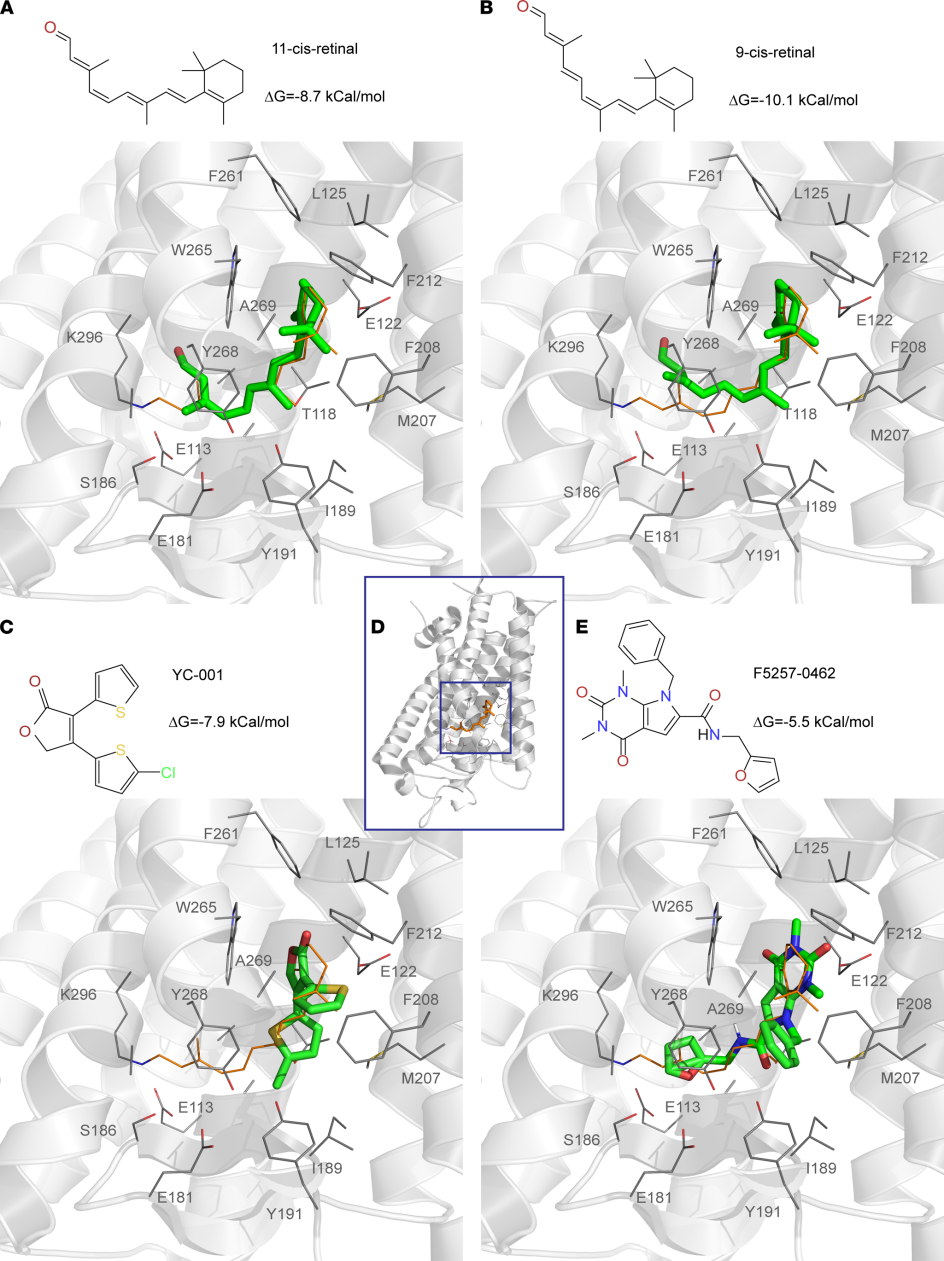
Virtual docking of pharmacological chaperones in the crystal structure of bovine RHO (PDB ID:1f88). (**A**–**C** and **E**) The most stable docking conformations of pharmacological chaperones, 11-*cis*-retinal, 9-*cis*-retinal, YC-001, and F5257-0462, displayed as sticks, respectively. The experimentally bound 11-*cis*-retinal in the actual structure shown in lines was deleted to spare the chromophore pocket for the docking calculations. Chemical structure and ΔG of each docked compound were shown on the top. (**D**) The whole crystal structure of bovine RHO (PDB ID:1f88) was shown in a cartoon display with bound 11-*cis*-retinal shown in orange sticks and surrounding residues in lines. The extracellular/intradiscal side of RHO was facing down. Boxed area centered by 11-*cis*-retinal (orange) was defined as the docking space for AutodockVina.

Our previous study suggests that YC-001 binds in the chromophore pocket of rod opsin (26). While the complex structure of rod opsin with YC-001 is challenging to obtain, we used a docking calculation by AutodockVina (see Supplemental Methods, ref. 33) to see how small-molecule chaperones may bind to rod opsin. We calculated the most stable conformations of 11-*cis*-retinal, 9-*cis*-retinal, YC-001, and F5257-0462 as ligands in the chromophore pocket of the bovine RHO structure (PDB ID:1f88 with the occupied 11-*cis*-retinal deleted; Figure 2). The docked 11-*cis*-retinal (ΔG = –8.7 kcal/mol, calculated free energy of binding) overlapped with its experimental conformation (except for the aldehyde tail due to the lack of Schiff base linkage in the docking), confirming the reliability of the docking algorithm (Figure 2, A and D). Docked 9-*cis*-retinal sat in a similar conformation as 11-*cis*-retinal, which stabilized the complex structure with ΔG = –10.1 kcal/mol (Figure 2B). Docked YC-001, with a smaller scaffold, sat in the β-ionone ring pocket with ΔG = –7.9 kcal/mol (Figure 2C). The α,β-unsaturated butyrolactone of YC-001 was stacked with the side chain of Trp265, and this finding agrees with the Trp-fluorescence quenching when YC-001 was bound to rod opsin (26). Compared with YC-001, F5257-0462 occupied a larger portion of the chromophore pocket (ΔG = –5.5 kcal/mol; Figure 2E). This docking result is in general agreement with our high-content imaging analysis that the chaperone activity of these compounds toward *RHO* mutants is: 9-*cis*-retinal > F5257-0462 > YC-001. As an aldehyde, 9-*cis*-retinal is cytotoxic (26, 28), and its efficacy is highly light-sensitive; thus, YC-001 and F5257-0462 are safer agents for ex vivo analysis.

### Fast ocular clearance of YC-001 and its stability in the culture medium.

**Figure 3:**
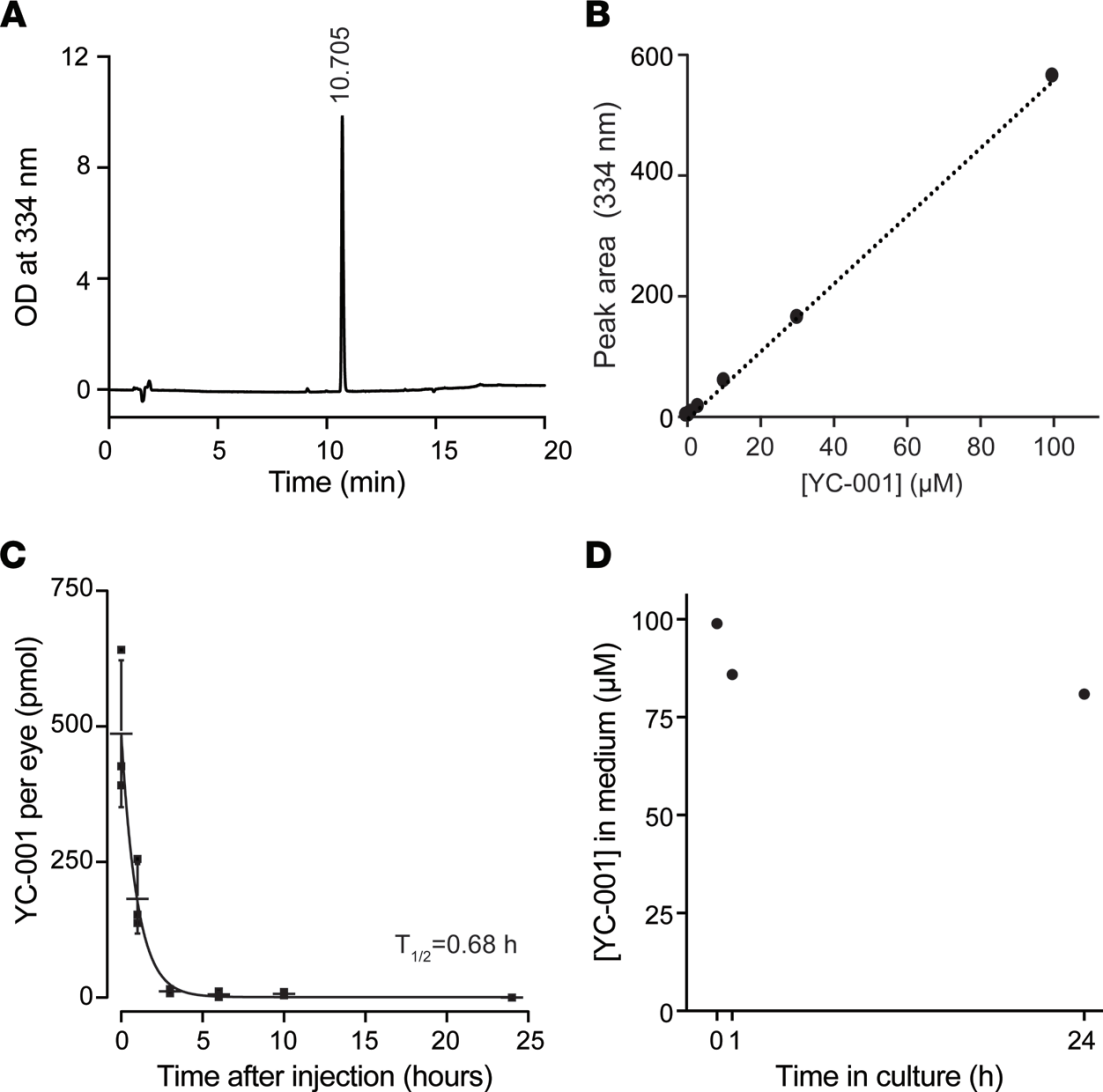
Ocular clearance of YC-001 and its stability in culture medium. YC-001 was dissolved in PEG35 castor oil and administered to C57BL/6J mice via an intravitreal injection (IVI). Ocular YC-001 level at different times after IVI was quantified by high-performance liquid chromatography (HPLC). (**A**) Chromatogram of 10 μM YC-001 showed a single peak at 10.7 minutes, detected with absorption at 334 nm. (**B**) The standard curve of YC-001 showed a linear correlation between peak area and YC-001, *R*^2^=0.999. (**C**) The YC-001 level (pmol) per eye plotted as a function of time after IVI. The curve was fitted by exponential decay using Origin8.1 software. Inset, ocular half-life (t_1/2_) of YC-001. *n =* 3. (**D**) Stability of YC-001 (%) at 0, 1, and 24 hours in cultured medium at 37^o^C.

Due to its short serum half-life (t_1/2_; less than 1 hour; ref. 26), YC-001 cannot be maintained at an efficacious dose in the eye via systemic administration. When dissolved in a biocompatible hydrophobic solvent (PEG35 castor oil) and delivered by intravitreal injection (IVI), YC-001 also showed fast ocular clearance with a t_1/2_ at 0.68 hours (Figure 3, A–C). Thus, an in vivo efficacy study of YC-001 was impeded by its short t_1/2_ using either systemic or intravitreal administration in a hydrophobic vehicle. This necessitated the development of an alternative in vitro retinal culture.

High-performance liquid chromatography (HPLC) analysis showed that YC-001 was stable in culture medium at 37°C for 24 hours (Figure 3D), so a relatively constant YC-001 concentration could be achieved by culturing retinal explants with daily medium changes. Mouse neural retinae were isolated with their retinal pigment epithelium (RPE) and cultured ex vivo (Supplemental Figure 3, A–C); the RPE layer was well maintained and remained attached to photoreceptor cells at 10 days in vitro (DIV) (Supplemental Figure 3, D and E).

### YC-001 increases the percentage of RHO^P23H^ transported to the ciliary protrusions (CP) in the Rho^P23H/P23H^ mouse retinae.

**Figure 4:**
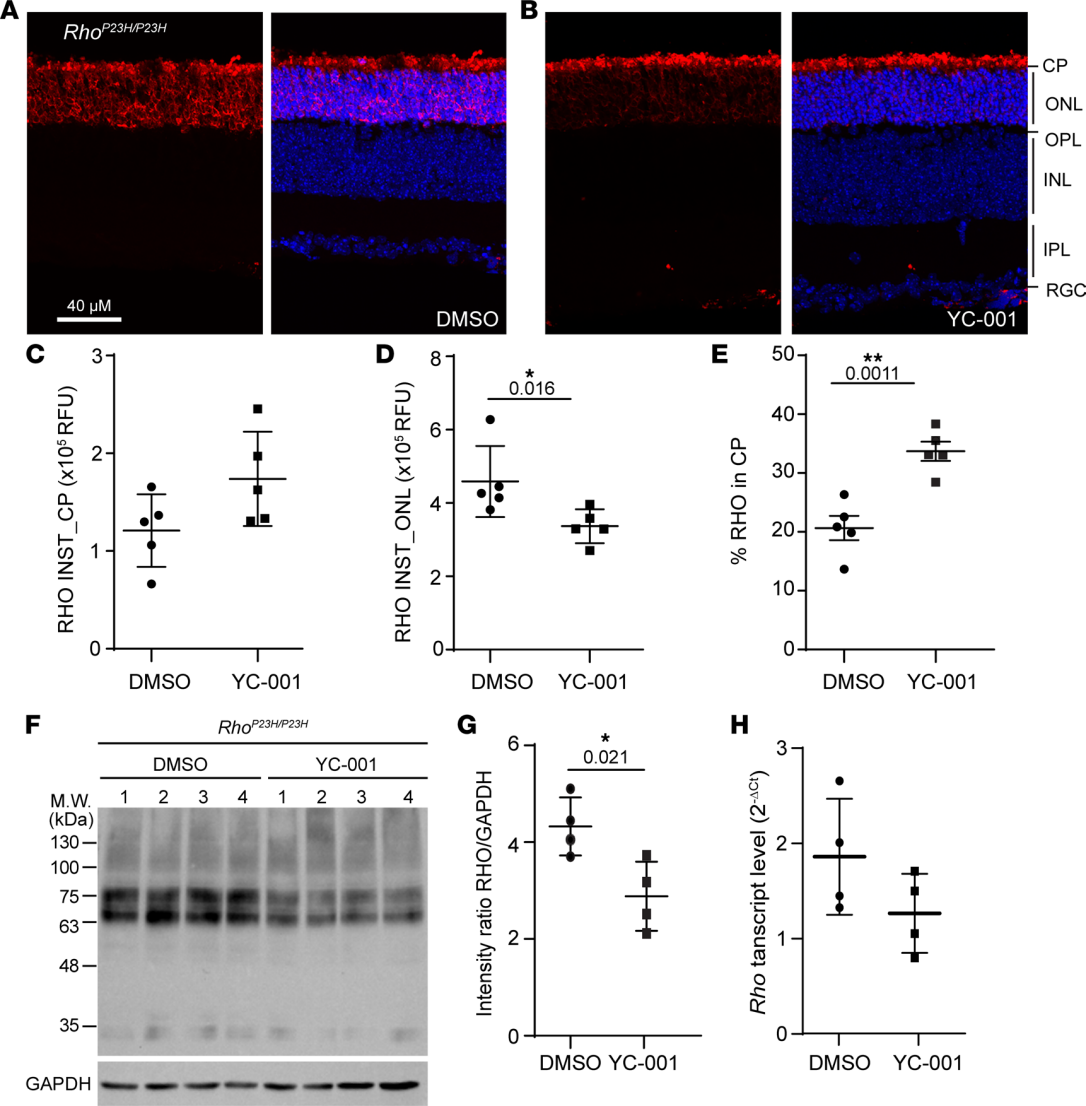
YC-001 rescues RHO^P23H^ from the outer nuclear layer (ONL) to the outer segments (OS) in mouse *Rho^P23H/P23H^* retinal explants. Mouse *Rho^P23H/P23H^* retinal explants were isolated at P15 and cultured with DMSO or 40 μM YC-001 for 48 hours before analyses. (**A** and **B**) Immunofluorescence images of cryosections from retinal explants treated with DMSO (**A**) and YC-001 (**B**). Left panels are immunostainings of RHO alone, and right panels are composite images of staining with RHO (red) and Hoechst33343 (blue) for nuclear stain. (**C**–**E**) Plots of RHO intensities in the ciliary protrusions (RHO INST_CP) and in the ONL (RHO INST_ONL), as well as the percentage of RHO intensity in the ciliary protrusions (% RHO in CP), respectively. RFU, relative fluorescence unit. Data are shown as mean ± SDs. *n =* 5. (**F**) Immunoblots against RHO from retinal explants, with GAPDH as loading control. Each number represents one retina. (**G**) Plot of RHO/GAPDH intensity ratio from immunoblots in **F**. *n =* 4. (**H**) Plot of *Rho* transcript level in *Rho^P23H/P23H^* retinal explants, with β-actin as endogenous control. *n =* 4. * and **, *P* < 0.05 and 0.01, respectively, by the Mann Whitney *U* test.

Because in vitro mammalian cell models lack a primary ciliary structure like the OS of rods, we tested the pharmacological chaperone activity of YC-001 on RHO^P23H^ in mouse *Rho^P23H/P23H^* retinal explant culture. The retinae were isolated at P15 and cultured with 40 μM YC-001 for 48 hours before analyses (Figure 4). Because *Rho^P23H/P23H^* retinae only express RHO^P23H^, most of RHO was stained in the outer nuclear layer (ONL) due to misfolding, and only 20.5% ± 2.0% of RHO was seen at the CP where OS was not properly formed (34) in the retinal explants treated with DMSO control (Figure 4, A and C–E, and Supplemental Figure 4). Treated with YC-001, RHO^P23H^ level at the CP was not significantly increased, but it was decreased in the ONL, resulting in an increase of the percentage of RHO^P23H^ in the CP to 33.5% ± 1.6% (Figure 4, B–E, and Supplemental Figure 4). Immunoblots showed that the total RHO level was not increased; rather, it was decreased in the *Rho^P23H/P23H^* retinae by YC-001 treatment (Figure 4, F and G) (see complete unedited blots in the supplemental material). A nonsignificant change in transcription of *Rho* by YC-001 treatment suggests that the reduced RHO^P23H^ protein level is not due to reduced Rho biosynthesis (Figure 4H). Collectively, these results suggest that YC-001 increases the percentage of ciliary targeted RHO^P23H^ primarily by decreasing the level of misfolded Rho^P23H^ mutant in the ONL, which is contributed by reduced total RHO^P23H^ protein load.

### YC-001 and F5257-0462 preserve ONL thickness in Rho^P23H/+^ retinal explants.

**Figure 5:**
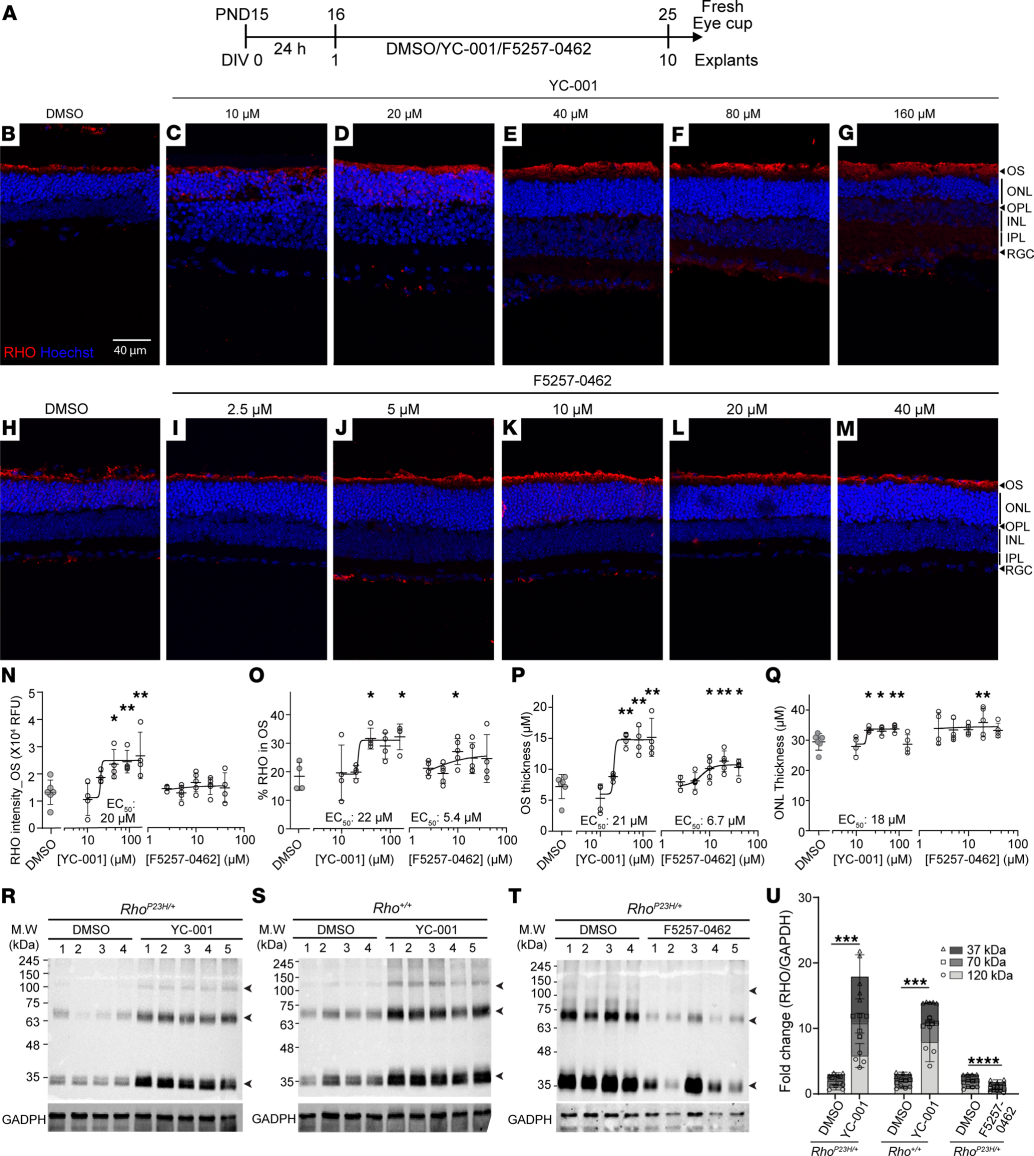
YC-001 and F5257-0462 protects *Rho^P23H/+^* retinal explants. (**A**) Schematic illustration of the experiment design. Mouse *Rho^P23H/+^* retinal explants were isolated at P15 and cultured for 1 DIV in medium; they were then treated with YC-001 (10, 20, 40, 80, and 160 μM), F5257-0462 (2.5, 5, 10, 20, and 40 μM), or DMSO control for 9 DIV. Media were refreshed every day. (**B**–**M**) Immunofluorescence images of *Rho^P23H/+^* retinal explants at 10 DIV treated with DMSO (**B** and **H**) or 10, 20, 40, 80, and 160 μM of YC-001 (**C**–**G**) and 2.5, 5, 10, 20, and 40 μM of F5257-0462 (**I**–**M**). Scale bars: 40 μm. (**N** and **O**) RHO intensity and percentage RHO in OS. (**P** and **Q**) OS/IS thickness and ONL thickness. Data are shown as mean ± SD. *n =* 4–7. *, **, *P* < 0.05 and 0.01, respectively, by the Kruskal-Wallis test. (**R**–**T**) Immunoblots against RHO from the *Rho^P23H/+^* and *Rho^+/+^* treated with 40 μM YC-001 or DMSO (**R** and **S**) and 10 μM F5257-0462 or DMSO (**T**) (30 μg total protein). Arrowheads, RHO monomer, dimer, and tetramer at ~37, 70, and 120 kDa. Each number represents 1 retina. GAPDH was loading control. (**U**) Fold change of RHO/GAPDH intensity ratio measured from **R**–**T**. Data are shown as mean ± SD. *n =* 4–5. ***, ****, *P* < 0.001 and 0.0001, respectively, by the Mann Whitney *U* test.

Temporal changes of retinal explants culture confirmed a faster photoreceptor loss in the *Rho^P23H/+^* versus WT retinae, validating this ex vivo model of adRP for testing the efficacy and safety of the small molecule chaperones (Supplemental Result and Supplemental Figures 5 and 6). We analyzed the retinal explants at 10 DIV (1 DIV with medium followed 9 DIV with compounds) to test the efficacy of compounds because sufficient retinal degeneration was observed while the OS was still preserved at this time point (Supplemental Figure 5, G and H). To determine whether the nonretinoid chaperones rescue RHO proteostasis and ameliorates retinal degeneration in the *Rho^P23H/+^* retinae (a model of adRP), we treated mouse *Rho^P23H/+^* and WT retinal explants with multiple doses of YC-001, F5257-0462, or DMSO for 9 DIV before collecting the tissue for analysis (Figure 5 and Supplemental Figures 7 and 8). We observed a YC-001 dose–dependent increase in RHO intensity in the OS and percentage of RHO in the OS, as well as OS/IS thickness (Figure 5, B–G and N–P; EC_50_, 20–22 μM), while mislocated RHO staining in the ONL was notably reduced, compared with DMSO control. YC-001 treatment did not affect RHO level in the OS of WT retinal explants, but it increased their OS thickness (Supplemental Figure 7, D, F, L, and M). F5257-0462 only increased the percentage of RHO in the OS (10 μM) and OS thickness (10–40 μM) in the *Rho^P23H/+^* retinal explants (Figure 5, H–M and N–P). F5257-0462 did not affect RHO level in the OS, OS thickness, or ONL in the WT retinal explants (Supplemental Figure 7, D–E and K–M). The RHO stabilization by YC-001 was further confirmed by immunoblots, showing a > 3-fold increase of RHO level in both YC-001–treated *Rho^P23H/+^* and YC-001–treated WT retinae compared with corresponding DMSO controls (Figure 5, R, S, and U) (see complete unedited blots in the supplemental material). However, F5257-0462 reduced total RHO protein level in the *Rho^P23H/+^* retinal explants (Figure 5, T and U) (see complete unedited blots in the supplemental material), suggesting that F5257-0462 may affect the transcription or degradation of RHO. The YC-001 treatment led to significantly thicker ONL in both *Rho^P23H/+^* (34.8, 32.8, and 34.5 μm for 20–80 μM YC-001 treatment, respectively, and 30.9 μm for DMSO control) and WT retinal explants compared with DMSO controls (Figure 5, B–G and Q, and Supplemental Figure 7, D, F, and K). F5257-0462 also increased ONL thickness of *Rho^P23H/+^* retinal explants at 20 μM (35.8 ± 5.3 μm; Figure 5, H–M and Q), but it did not affect ONL thickness of WT retinae (Supplemental Figure 7, D–E and M). We also treated the *Rho^P23H/+^* retinal explants with 5 μM 9-*cis*-retinal and kept the culture dish in the dark; notably, 9-*cis*-retinal did not affect RHO level in the OS, OS thickness, or ONL thickness of *Rho^P23H/+^* retinae (Supplemental Figure 7, B, C, and K–M). Due to the cytotoxicity of aldehydes (35, 36), we did not try higher concentrations of 9-*cis*-retinal in the explant culture. Collectively, YC-001 showed its pharmacological chaperone activity in increasing properly transported RHO in OS, which in turn protected photoreceptors from degeneration in the *Rho^P23H/+^* retinal explants. Based on the fact that YC-001 did not stabilize RHO^P23H^ in the *Rho^P23H/P23H^* retinae (Figure 4, F and G), and that it stabilized RHO in WT retinae (Figure 5, S and U), we conclude that YC-001 rescued RHO homeostasis in the *RHO^P23H/+^* retinae mainly via stabilized RHO^WT^. F5257-0462 did not stabilize RHO total protein but led to a thicker OS and ONL, suggesting that only the transport of RHO was rescued in the *Rho^P23H/+^* retinae. Although the 2 compounds showed differences in RHO levels, both preserved the ONL thickness, suggesting their protection to photoreceptors.

### YC-001 improves RHO protein quality and proteostasis of the Rho^P23H/+^ retinae.

**Figure 6:**
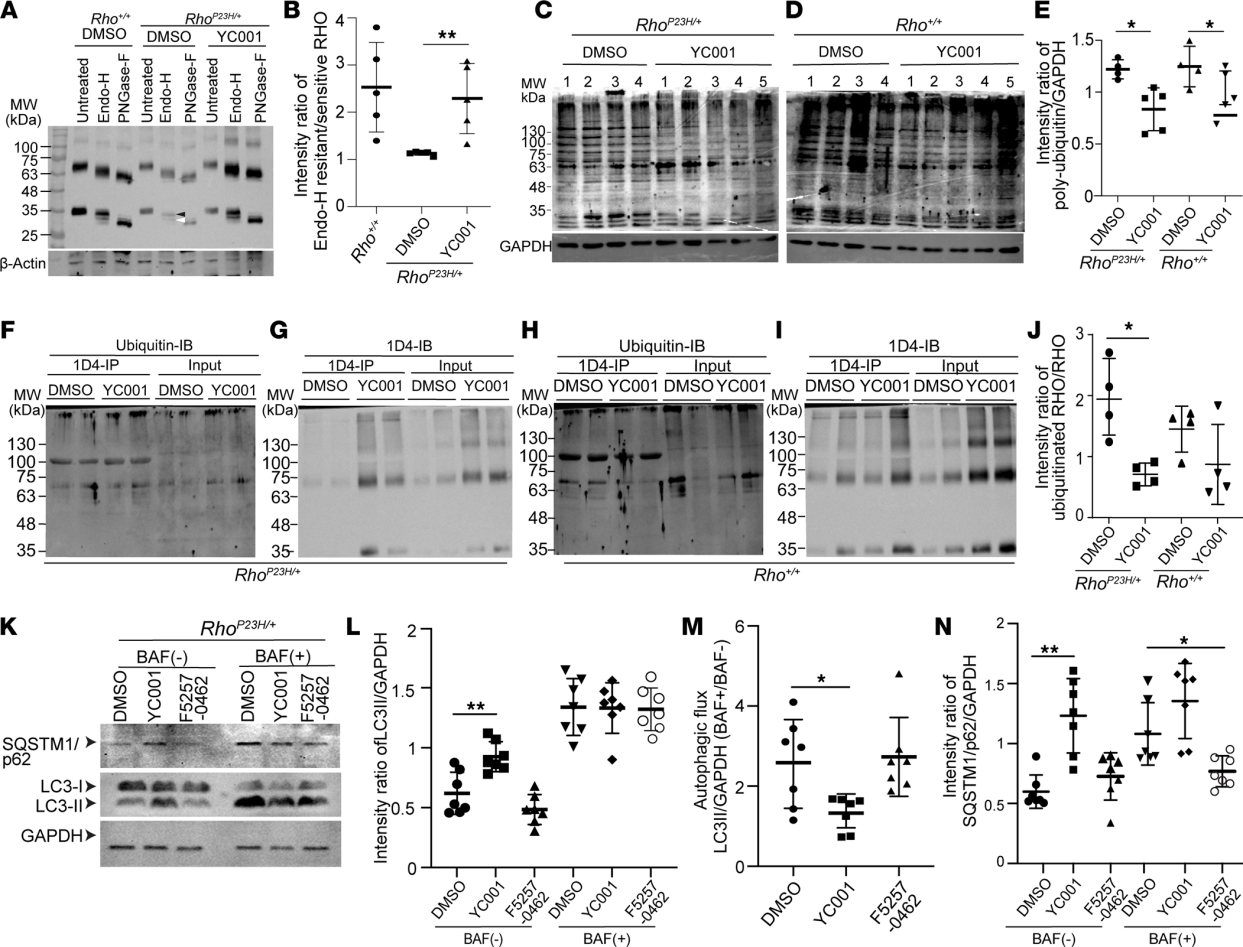
YC-001 improves RHO homeostasis and reduces autophagy flux in the *Rho^P23H/+^* retinal explants. *Rho^P23H/+^* and *Rho^+/+^* mouse retinal explants were treated with DMSO, 40 μM YC-001, or 10 μM F5257-0462 for 9 DIV before immunoblots. β-Actin and GAPDH were loading controls. (**A**) Immunoblots of RHO from retinal lysates (15 μg total protein) that were untreated or were EndoH- or PNGaseF-treated. Black and white arrow heads, EndoH-resistant and cleaved RHO monomers, respectively. (**B**) Intensity ratio of the EndoH-resistant to EndoH-sensitive RHO monomers, measured from **A**. *n =* 5. (**C** and **D**) Immunoblots against poly-ubiquitin from retinal explants (60 μg total protein). Each number represents 1 retina per lane. (**E**) Intensity ratio of poly-ubiquitinated proteins (whole lane) to GAPDH, measured from **C** and **D**. *n =* 4–5. (**F**–**I**) Retinal explant lysates (50 μg total protein) were i.p. with 1D4 anti-RHO antibody and IB against poly-ubiquitin (**F** and **H**) and RHO (**G** and **I**). Input (10 μg total protein), lysates before i.p. **F** and **G** are from *Rho^P23H/+^* retinal explants, and **H** and **I** are from *Rho^+/+^* retinal explants. (**J**) Intensity ratio of ubiquitinated RHO to total RHO measured from **F**–**I**. The band near 100 KDa is the immunoglobulin heavy chain and was excluded from measurements. *n =* 4. (**K**) IB against autophagy flux markers LC3B and SQSTM/p62 from *Rho^P23H/+^* retinal explants (60 μg total protein) cotreated without (–) or with (+) 100 nM bafilomycin-A1 (BAF). (**L**) Intensity ratio of LC3-II (16 kDa) to GAPDH, measured from **K**. (**M**) Plot of autophagic flux, calculated by the ratio of LC3-II (normalized by GAPDH) from retinal explants treated with BAF to that without BAF. (**N**) Intensity ratio of SQSTM/p62 to GAPDH, measured from immunoblots in **K**. *n =* 7. Data are shown as mean ± SD. *, **, *P* < 0.05 and 0.01, respectively, by the Kruskal-Wallis test.

To address whether the glycosylation profile of RHO was improved by YC-001, we cultured *Rho^P23H/+^* retinal explants with YC-001 or DMSO for 9 DIV and treated the retinal lysates with endoglycosidase H (EndoH) or peptide:N-glycosidase F (PNGaseF) before immunoblotting against RHO. While misfolded glycoproteins that have accumulated in the ER are sensitive to EndoH-mediated glycan cleavage, properly folded glycoproteins are further processed by Golgi, and they are resistant to EndoH cleavage (37, 38). PNGaseF cleaves N-glycans nonselectively, resulting in completely deglycosylated peptides (39). While most RHO in the WT retinal explants was resistant to EndoH cleavage, RHO in the DMSO-treated *Rho^P23H/+^* retinae showed a lower ratio of EndoH-resistant (~35 kDa) to EndoH-cleaved monomers (~32 kDa) (Figure 6, A and B, and Supplemental Figure 9A) (see complete unedited blots in the supplemental material), confirming that the *Rho^P23H/+^* retinae contains less maturely glycosylated RHO (37). The ratio was restored by YC-001 treatment, indicating that YC-001–treated *Rho^P23H/+^* retinae had a higher percentage of RHO with Golgi-processed glycans. This result showed that YC-001 improves the glycosylation profile of RHO in the *Rho^P23H/+^* retinal explants, potentially due to a higher level of RHO^WT^ stabilized by YC-001.

Ubiquitination is a marker of misfolded proteins. Immunoblotting of poly-ubiquitin showed that YC-001 significantly reduced the total amount of ubiquitinated proteins in both *Rho^P23H/+^* and WT retinae (Figure 6, C–E), suggesting that YC-001 improved the overall protein homeostasis in the retinal explants (see complete unedited blots in the supplemental material). We then i.p. RHO and immunoblotted (IB) against poly-ubiquitin and RHO (Figure 6, F–J, and Supplemental Figure 9B). The total yield of RHO increased in both the YC-001–treated *Rho^P23H/+^* and WT retinal explants compared with DMSO controls (Figure 6, G and I), and the ubiquitinated RHO was not changed by YC-001, resulting in reduced relative intensity of ubiquitinated RHO to total RHO from 2.21 ± 0.80 in DMSO to 0.97 ± 0.35 in YC-001–treated retinae (similar as in WT retinae) (Figure 6, F–J). Because the level of ubiquitinated RHO was not decreased by YC-001, the reduced percentage of ubiquitinated RHO was mainly due to increased total RHO level, which is mainly RHO^WT^. This result provides further evidence of chaperone activity of YC-001 in stabilizing RHO and improving RHO homeostasis in the *Rho^P23H/+^* retinae.

Autophagy is a major proteolytic pathway degrading misfolded proteins, so we tracked the autophagy flux in the retinal explants. We IB 2 autophagosome components — microtubule-associated proteins 1A/1B light chain 3B (LC3) and sequestosome-1 (SQSTM1/p62) (40) — in the *Rho^P23H/+^* retinal explants treated with 40 μM YC-001, 10 μM F5257-0462, or DMSO for 9 DIV. YC-001 significantly increased the lipidated LC3 (LC3-II) in *Rho^P23H/+^* retinae (1.5 fold; Figure 6, K and L, and Supplemental Figure 9C), suggesting that autophagosomes was increased (see complete unedited blots in the supplemental material). Cotreated with Bafilomycin A1 (BAF), an inhibitor of lysosome activity, YC-001–treated retinal lysate showed no difference in LC3-II accumulation than DMSO control. Thus, the autophagy flux, calculated by the ratio of normalized LC3-II in the presence of BAF (BAF^+^) to that in the absence of BAF (BAF^–^), was reduced in the YC-001–treated *Rho^P23H/+^* retinal explants, compared with DMSO control (Figure 6M). Reduced autophagy flux by YC-001 was also confirmed by the increased SQSTM1/p62 level, which is a cargo of autophagosome (Figure 6, K and N). Interestingly, F5257-0462 did not change LC3-II or SQSTM1/p62 levels (Figure 6, K–N), suggesting that it does not affect autophagy flux in the *Rho^P23H/+^* retinae (see complete unedited blots in the supplemental material).

### Efficacious doses of YC-001 and F5257-0462 are safe.

**Figure 7:**
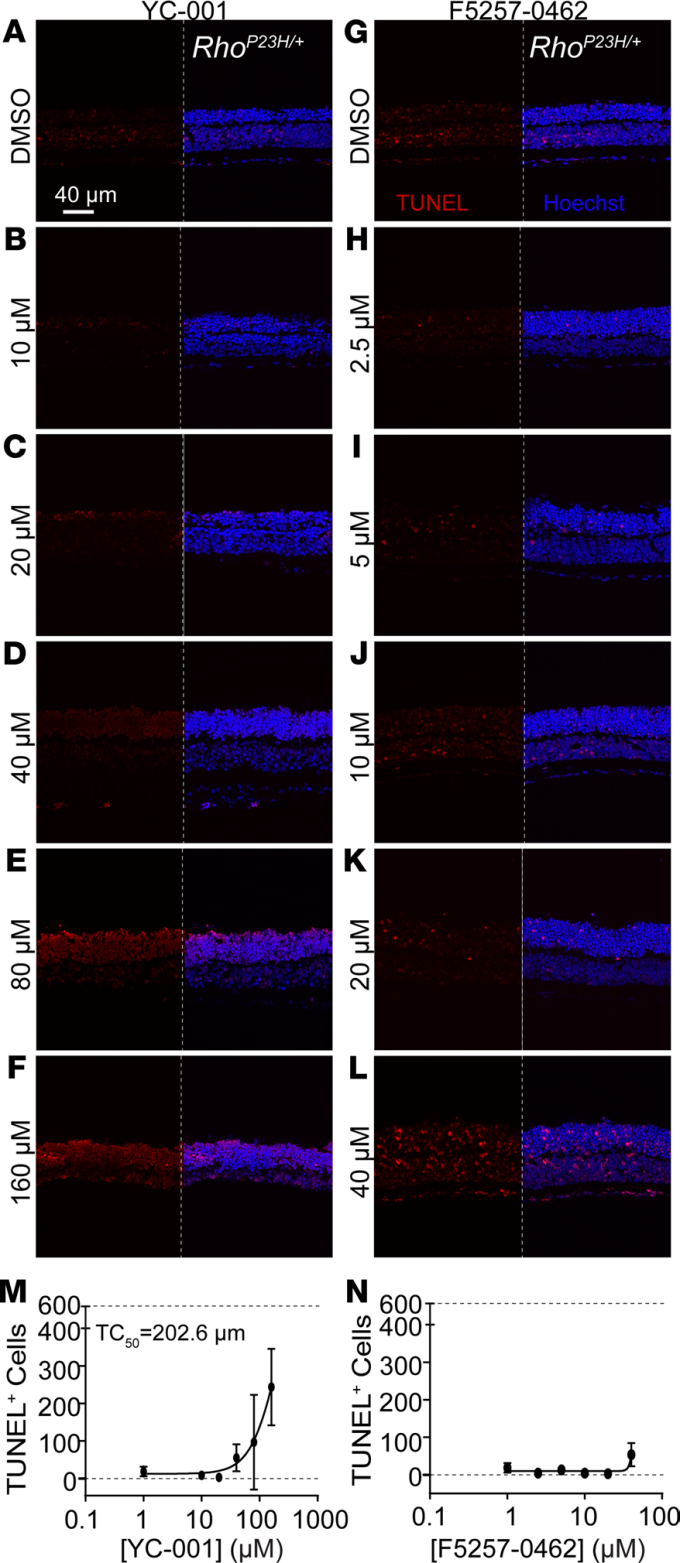
The efficacious dose of YC-001 and F5257-0462 are safe for the retinal explant. TUNEL staining was performed to *Rho^P23H/+^* mouse retinal explants isolated at P15 and treated with YC-001 (10, 20, 40, 80, and 160 μM) and F5257-0462 (2.5, 5, 10, 20, and 40 μM) or DMSO for 9 DIV. (**A**–**L**) TUNEL-stained *Rho^P23H/+^* retinal explants treated with YC-001 doses (**A**–**F**) and F5257-0462 doses (**G**–**L**). The left panel of all immunofluorescence images represent the TUNEL staining (red, TUNEL^+^ cells), and right panel is composite image of TUNEL (red) and Hoechst 33342 (blue, nucleus). Scale bars: 40 μm. (**M** and **N**) TUNEL^+^ cell number in *Rho^P23H/+^* retinal explants as a function of concentrations of YC-001 and F5257-0462, respectively. Dose response curve was fitted by a modified Hill function. Dotted lines on the top and bottom of graph represents the upper and lower limit, respectively. *n =* 4–7. Data are shown as mean ± SD.

We then evaluated the safety of YC-001 and F5257-0462 by testing each compound at 5 doses and counting dead cells in the retinal explants via TUNEL (Figure 7). Normalized by the positive control (DNase-I–treated) as 100% and DMSO group as 0%, YC-001 showed a TC_50_ (the toxic concentration of a chemical that causes the cells growing 50% as well as control) at 202 μM in *Rho^P23H/+^* retinal explants (Figure 7, A–F and M) and 64 μM in WT retinae (Supplemental Figure 10, A–E). The efficacious dose of YC-001 at 40 μM did not increase the number of TUNEL^+^ cells compared with DMSO control in both *Rho^P23H/+^* and WT retinae (Figure 7, A, D, and M, and Supplemental Figure 10, A, B, and E). F5257-0462 showed no toxicity at most tested concentrations suggesting a TC_50_ > 40 μM, and its efficacious dose (10 μM) showed no toxicity in both *Rho^P23H/+^* and WT retinae (Figure 7, G–L, and Supplemental Figure 10, F, G, and J). This result showed the efficacious concentrations of both compounds are safe for the retina, and F5257-0462 showed a more significant therapeutic window than YC-001.

### Transcriptome analysis shows a time-dependent change of molecular pathways affected by YC-001.

**Figure 8:**
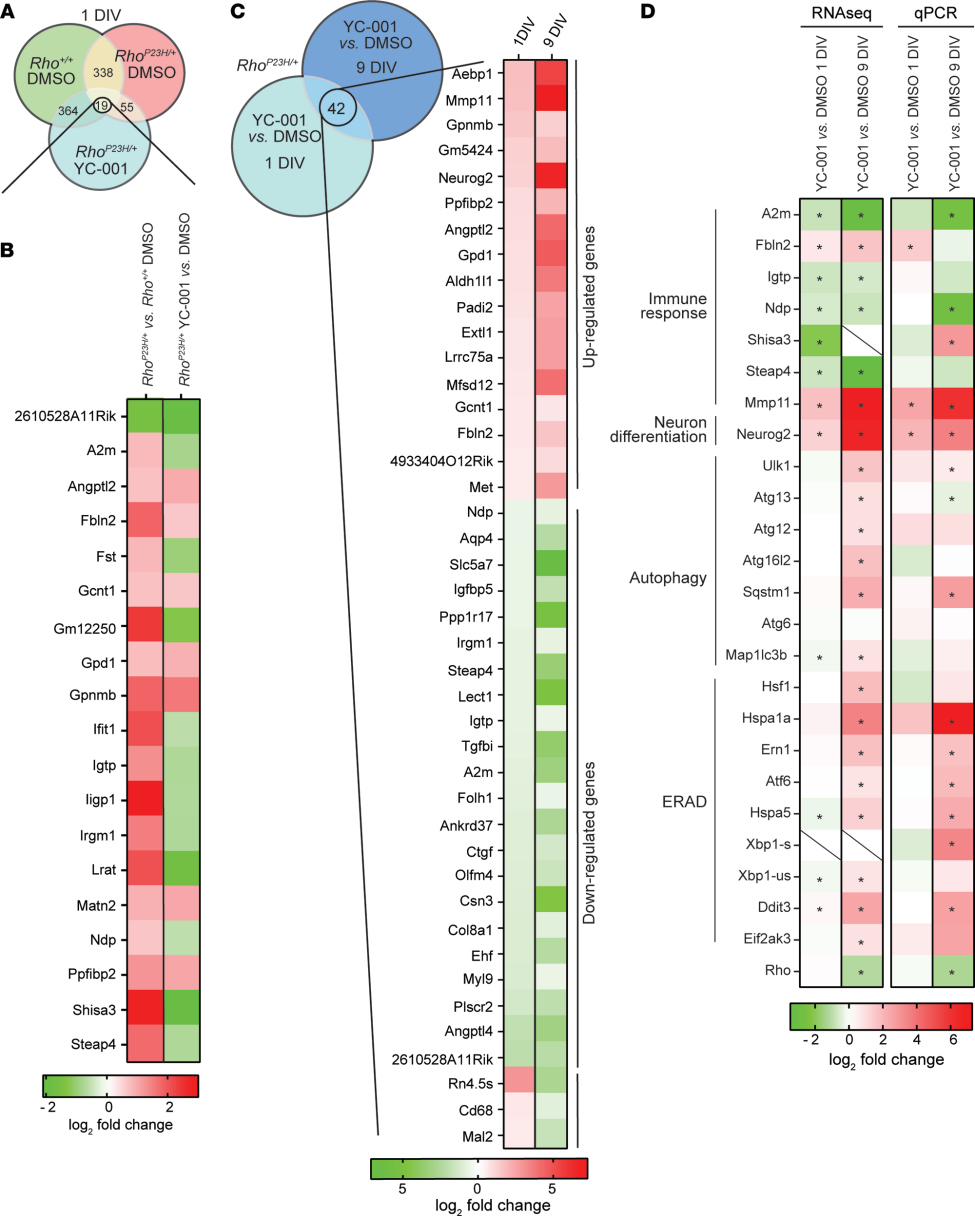
Transcriptome analysis from RNA-Seq data comparing *Rho^+/+^* and *Rho^P23H/+^* retinal explants treated with DMSO or YC-001. Total RNA was isolated from *Rho^P23H/+^* and *Rho^+/+^* mouse retinal explants at P15 and treated with DMSO or 40 μM YC-001 for 1 and 9 DIV. (**A**) Venn diagram showing the differentially expressed genes (DEGs) between *Rho^+/+^* retinae treated with DMSO (*Rho^+/+^* DMSO), *Rho^P23H/+^* retinae treated with DMSO (*Rho^P23H/+^* DMSO), or YC-001 (*Rho^P23H/+^* YC-001) at 1 DIV. (**B**) Heatmap of the log_2_ fold change of 19 shared DEGs between *Rho^P23H/+^* DMSO versus *Rho^+/+^* DMSO, and *Rho^P23H/+^* YC-001 versus *Rho^P23H/+^* DMSO at 1 DIV. (**C**) Heatmap represents the comparative log_2_ fold change of 42 common DEGs shared between *Rho^P23H/+^* YC-001 versus *Rho^P23H/+^* DMSO at 1 and 9 DIV. (**D**) Heatmaps showing log_2_ fold changes of selected DEGs from the RNA-Seq (left) and qPCR (right) data analysis involved in immune response, autophagy, and ER associated protein degradation pathways (ERAD). Slashed boxes represent those not applicable or detected. *n =* 3. * signifies *P* < 0.05 by the Kruskal-Wallis Test.

To identify early retinal changes caused by the *RHO^P23H^* mutation before massive degeneration has occurred, we isolated retinae from *Rho^P23H/+^* and WT mice at P15 and cultured them for 1 DIV followed by YC-001 or DMSO treatment for 1 DIV. After this, we performed RNA-Seq (data accessible through GEO series accession no. GSE179754, https://www.ncbi.nlm.nih.gov/geo/query/acc.cgi?acc=GSE179754) and transcriptome analysis. A total of 338 differentially expressed genes (DEGs) were identified comparing the *Rho^P23H/+^* versus WT retinal explants using the stringency of > 1.5-fold change with FDR < 0.05 (Figure 8A and Supplemental Figure 11). Gene ontology pathway analysis showed affected biological processes indicating cell stress in the *Rho^P23H/+^* retinae included defense response to other organisms, response to radiation, detection of external stimulus, and response to reactive oxygen species among others (Supplemental Table 1). Photoreceptor functional changes were also reflected by reduced expression of genes in pathways including detection of visible light, RHO-mediated signaling pathway, and visual perception (Supplemental Table 1). In response to photoreceptor stress, retinal immune responses were affected, including changes in the IFN pathways, innate immune response, regulation of immune system process, lymphocyte-mediated immunity, and response to cytokines (Supplemental Table 1). In summary, cell stress, altered photoreceptor function, and immune response are already emerging before noticeable retinal degeneration occurs in the *Rho^P23H/+^* retinae.

To understand the early molecular events affected by YC-001, we compared the transcriptome of YC-001– versus DMSO-treated *Rho^P23H/+^* retinal explants at 1 DIV and found a total of 55 DEGs. Among these affected genes, 19 were also overlapping DEGs between *Rho^P23H/+^* versus WT retinae (both treated with DMSO at 1 DIV) and YC-001– versus DMSO-treated *Rho^P23H/+^* retinal transplants at 1 DIV (Figure 8, A and B, and Supplemental Figure 11). Furthermore, 11 of these overlapped 19 DEGs were upregulated in the *Rho^P23H/+^* versus WT retinal explants but downregulated when comparing YC-001– versus DMSO-treated *Rho^P23H/+^* retinal explants, indicating these abnormally expressed genes in the *Rho^P23H/+^* retinal explants were reverted by YC-001 treatment as early as 1 day. Among these 11 genes, we noticed that *Ifit1*, *Igtp*, and *Iigp1* are involved in the IFN-β signaling pathway (Gene Ontology, http://amigo.geneontology.org/amigo/term/GO:0035458). The IFN pathway is among the first innate immune responses to cellular damage or external insults (41); thus, reverted expression associated with YC-001 suggests alleviation of early innate immune responses in the *Rho^P23H/+^* retinae. Other immune response–related genes among these 11 restored genes include *A2M* and *Irgm1* (Figure 8B). Additionally, YC-001 treatment also reverted the RHO^P23H^-associated upregulation of genes involved in the retinal vascularization pathway, including *Ndp* and *Shisa3* (Figure 8B). Pathway analysis of YC-001– versus DMSO-treated retinal explants at 1 DIV showed changes in pathways, including regulation of stress-activated MAPK cascade, innate immune response, cellular response to IFN-β, epithelial cell proliferation, and changes of metabolic processes, revealing the first set to our knowledge of molecular events affected by YC-001 treatment, focusing on modulations of cell stress and immune responses (Supplemental Table 2). These results suggest that YC-001 alleviated some cellular stress signals and reversed some of the early immune response signals.

We then followed the temporal changes of the transcriptome by comparing the DEGs of YC-001 and DMSO in the *Rho^P23H/+^* retinae at 1 and 9 DIV. At 9 DIV when YC-001–mediated retinal protection was observed, 4268 downregulated and 4329 upregulated DEGs were identified when comparing YC-001– versus DMSO-treated *Rho^P23H/+^* retinae, suggesting a profound transcriptome change. A total of 42 DEGs were shared between YC-001– versus DMSO-treated *Rho^P23H/+^* retinae at 1 DIV and 9 DIV (Figure 8C and Supplemental Figure 11). Except for 3 genes (*Rn4.5s*, *Cd68*, and *Mal2*), the remaining 39 shared DEGs showed the same trend in changes by YC-001 at 1 and 9 DIVs with higher amplitude of changes at 9 DIV. This result suggests that the effect of the changed gene expression by YC-001 at 1 DIV was magnified with time, leading to profound transcriptome change at 9 DIV in *Rho^P23H/+^* retinal explants. We also compared proteolytic pathways, including autophagy and ER-associated protein degradation (ERAD) pathways, which are closely related to RHO homeostasis. We observed that genes in these 2 pathways were upregulated in the YC-001–treated *Rho^P23H/+^* retinae versus DMSO control at 9 DIV, but not at 1 DIV, in both RNA-Seq data and quantitative PCR (qPCR) confirmations. This result suggests that these pathways are affected as secondary effects of YC-001 treatment (Figure 8D). The genes upregulated in the autophagy pathway by YC-001 at 9 DIV suggest increased autophagosome formation, although autophagy flux was reduced by YC-001 (Figure 6, K–N, and Figure 8D). Collectively, our results show that YC-001 treatment also indirectly affects the proteolytic capacity of cells in *Rho^P23H/+^* retinal explants.

### YC-001 reduces macrophage number in retinal explants.

**Figure 9:**
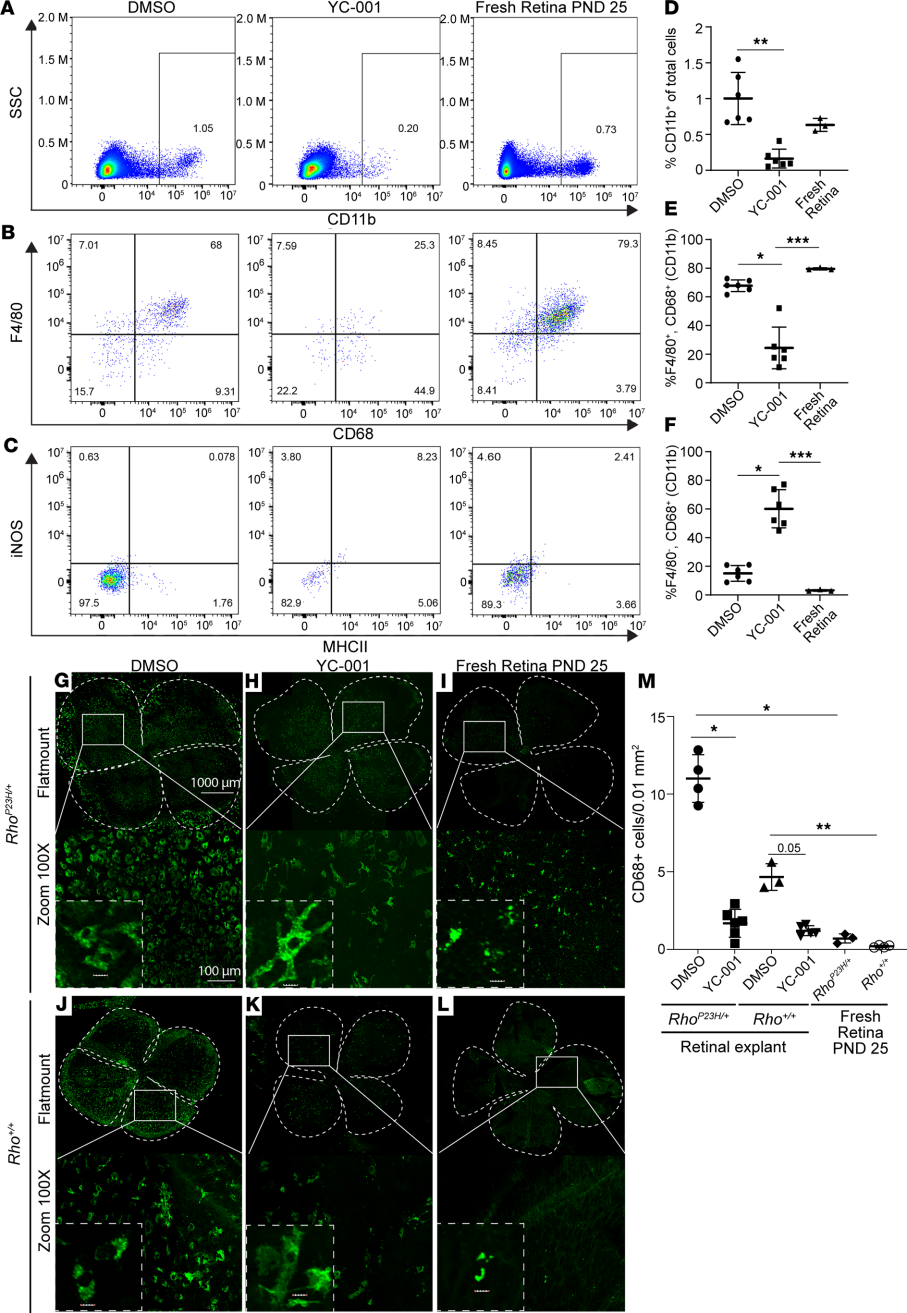
YC-001 reduces number of macrophages in the retinal explants. Mouse retinal explants were isolated at P15 and cultured for 1 DIV followed by treatment with 40 μM YC-001 or DMSO in the medium for 9 DIV, with the medium changed every day. Fresh retinal flat mounts at P25 are in vivo controls. (**A**–**F**) Retinae were dissociated to single-cell suspensions, stained, and profiled for immune cell markers including CD11b (for leukocytes including monocytes, macrophages, granulocytes, and NK cells), CD68 (macrophage/microglia), F4/80 (proinflammatory macrophage), inducible nitric oxide synthase (iNOS, M1 macrophage), and MHCII (marker of antigen presenting cells) through flow cytometry. (**A**) Gating of CD11b^+^ cells against side scattering (SSC). (**B**) Gated CD11b^+^ cells population were plotted for CD68 against F4/80. (**C**) Gated CD11b^+^ cell population plotted for MHCII against iNOS in DMSO and YC-001 retinal explant at DIV10 and fresh retina at P25 (in vivo control). (**D**, **E**, and **F**) Plots of percent of CD11b^+^ cells (**D**) calculated from **A**; F4/80^+^ and CD68^+^ cells (**E**) and F4/80^–^ and CD68^+^ cell (**F**) calculated from **B**. *n =* 6 for retinal explants and *n =* 3 for fresh retina. (**G**–**L**) Images of retinal flat mounts immunostained against CD68 in the *Rho^P23H/+^* and *Rho^+/+^,* respectively. Top panel (20×; scale bar: 1000 μm) and bottom panel (100×; scale bar: 100 μm), and inset in the bottom panel (500×; scale bar: 10 μm). (**M**) Plot of the number of CD68^+^ cells/0.01 mm^2^. *n =* 3–5. Data are shown as mean ± SD. *, **, ***, *P* < 0.05, 0.01, and 0.001, respectively, by the Kruskal-Wallis test.

To further understand the strong modulation of immune responses by YC-001 indicated by the RNA-Seq data, we profiled the microglia/macrophages/leukocytes in the *Rho^P23H/+^* retinal explants treated with DMSO or 40 μM YC-001 for 9 DIV using flow cytometry (Figure 9, A–F). Firstly, we observed that the percentage of CD11b^+^ cells reduced ~6 fold in the YC-001–treated retinae, compared with the DMSO control (Figure 9, A and D). Among these CD11b^+^ cells, YC-001 treatment led to a 2.78-fold decrease in the percentage of F4/80^+^CD68^+^ cells from 67.8% ± 4.1% (DMSO) to 24.3% ± 14.5%, while the percentage of F4/80^–^CD68^+^ cells increased from 15.0% ± 5.5% to 60.2% ± 13.3% (Figure 9, B, D, and F). Our results suggest that there are likely at least 2 populations of myeloid-derived cells within the retina of *Rho^P23H/+^* mice. We observed that the F4/80^+^CD68^+^ population presumably tissue-resident macrophages is selectively targeted by YC-001 treatments. Conversely, the F4/80^–^CD68^+^ (42), which could be tissue infiltrating monocytes, do not appear to be inhibited by YC-001. Regardless of treatment, the inflammatory markers, inducible nitric oxide synthase (iNOS) and MHCII (43), are scarce in the *Rho^P23H/+^* retinal explants, suggesting that mutation does not appear to induce inflammatory monocytes or the M1 lineage of macrophages (Figure 9C).

We then quantified the number of microglia/macrophages by immunostaining the microglia/macrophage marker CD68 in flat-mounts of retinal explants (Figure 9, G–M, and Supplemental Figure 12). Under DMSO treatment, *Rho^P23H/+^* retinal explants showed over 2 times as many CD68^+^ cells compared with WT retinal explants, confirming that the rod cell stress and death indeed activate retinal microglia as seen in vivo (Figure 9, G, J, and M) (44, 45). YC-001 decreased CD68^+^ cell number in both *Rho^P23H/+^* and WT retinal explants by > 5 fold compared with DMSO controls (Figure 9, H, K, and M), suggesting a potent inhibition of microglia/macrophages by YC-001. Interestingly, YC-001 also changed the morphology of CD68^+^ cells from rounded cell shape to a more elaborated shape with multiple projections, suggesting the change of macrophages from a phagocytotic state to a surveillance state (46). The number of CD68^+^ cells was significantly higher in the retinal explants at 9 DIV compared with retinae freshly isolated from age-matched eyes in both WT and *Rho^P23H/+^* mice (Figure 9, G, I, J, and L), indicating that the culture condition stimulates the activation and proliferation of microglia. Collectively, microglia proliferation was strongly inhibited by YC-001, possibly via an unknown mechanism independent of its chaperone activity. Müller glia marker glial fibrillary acidic protein (GFAP) level was similar between *Rho^P23H/+^* and WT retinal explants and remained unchanged by YC-001 treatment, indicating that YC-001 does not affect Müller glia activity (Supplemental Figure 13).

### F5257-0462 and YC-001 protect Rho^P23H/+^ retinae in vivo.

**Figure 10:**
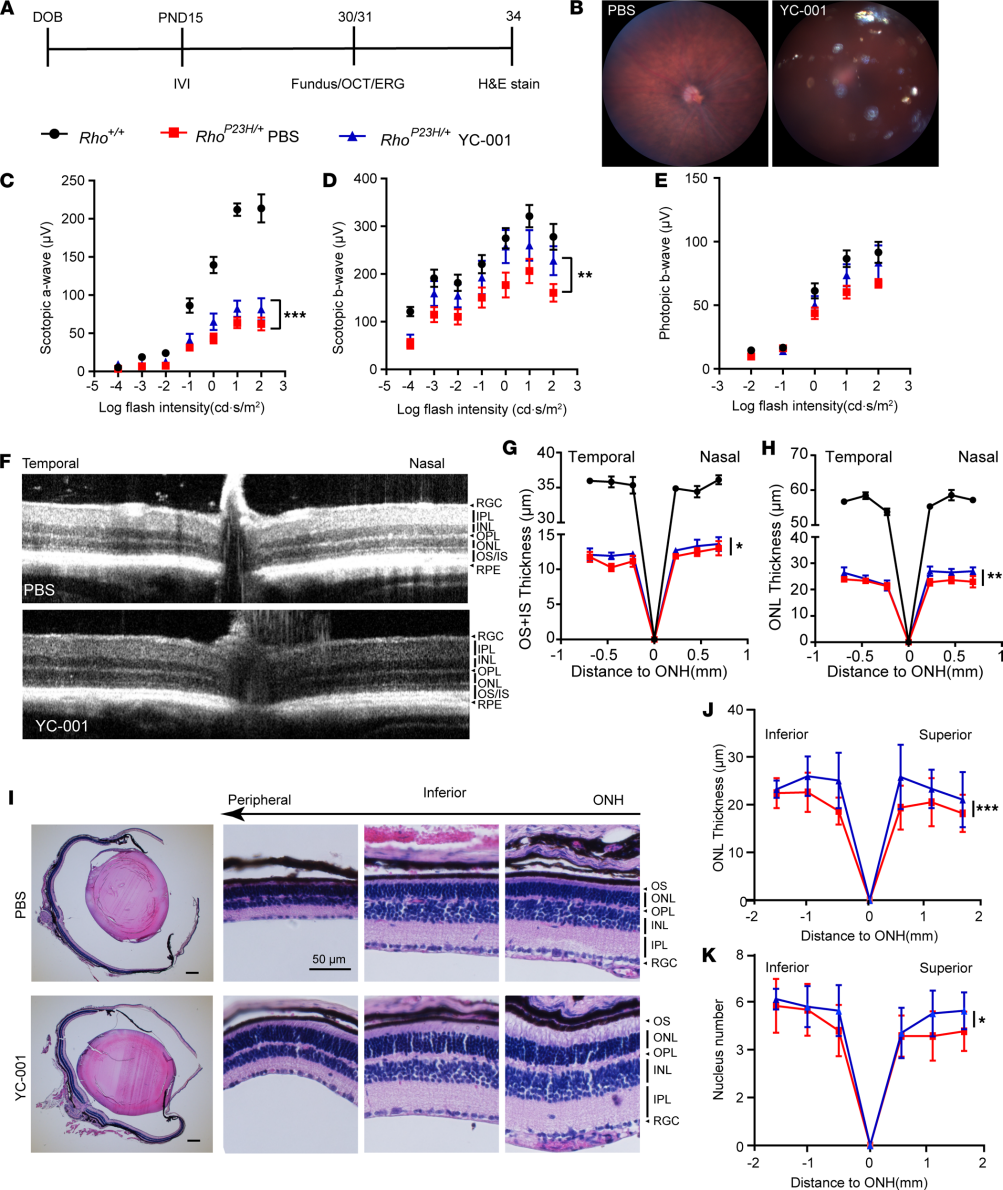
An intravitreal injection (IVI) of YC-001 slurry increased electroretinogram (ERG) response and ONL thickness in *Rho^P23H/+^* mice. (**A**) Timeline of the experimental procedures. *Rho^P23H/+^*–knock-in mouse eyes were injected intravitreally with PBS or 35 nmol/eye of YC-001 microparticles suspended in PBS at P15. Fundus and OCT images were collected at P30 followed by ERG recording at P31. H&E staining were performed at P34. Blue triangles and red squares are from *Rho^P23H/+^* mice treated with YC-001 and PBS, respectively, and black circles were from age-matched *Rho^+/+^* mice as normal control. (**B**) Fundus images of mice treated with PBS (left) and YC-001 (right). The yellow dots in the right panel were the YC-001 microparticles in the eye. (**C** and **D**) ERG scotopic a- and b-wave responses, respectively. *n =* 8. (**E**) Photopic b-wave responses. *n =* 8. (**F**) SD-OCT images of *Rho^P23H/+^* retinae. Top, PBS-treated; bottom, YC-001–treated. RGC, retinal ganglion cells; IPL, inner plexiform layer; INL, inner nuclear layer; OPL, outer plexiform layer; ONL, outer nuclear layer; OS/IS, outer/inner segments; RPE, retinal pigmented epithelium. (**G** and **H**) Spidergrams plotting the thicknesses of OS+IS and ONL, respectively, measured from SD-OCT images. *n =* 4–7. **(I**) H&E-stained retinal sections. Left panels, whole eye at low (4×) magnification (scale bar: 200 μm); and right panels, high magnification images (40×) at inferior side (scale bar: 50 μm). (**J** and **K**) Spidergrams plotting the ONL thickness and nucleus number, respectively, measured from **I**. *n =* 7. Data are shown as mean ± SEM. *, **, and ***, *P* < 0.05, 0.01, and 0.001, calculated by 2-way ANOVA.

Because YC-001 and F5257-0462 showed retinal protection ex vivo, we asked if we could observe the efficacy of these compounds in vivo. To achieve a continuous release of the compound, we firstly sonicated YC-001 into microparticles suspended in PBS, and then we administered 0.5 μL of this slurry containing 35 nmol of YC-001 to the *Rho^P23H/+^*–knock-in mice by 1 IVI at P15 (Figure 10A). Residual yellow particles of YC-001 could still be seen in the vitreous space 2 weeks after IVI (Figure 10B), suggesting that the slow release of YC-001 from the insoluble particles can be sustained for about 2 weeks. YC-001–treated eyes showed significantly higher scotopic electroretinogram (ERG) a- and b-waves at 2 weeks after IVI (Figure 10, C and D) without affecting the photopic ERG responses (Figure 10E). Spectral domain–optical coherence tomography (SD-OCT) showed significantly thicker ONL and outer/inner segment (OS/IS) layers in YC-001–treated eyes compared with PBS-treated*Rho^P23H/+^* controls at P30 (Figure 10, F–H). H&E staining of retinal sections at P34 confirmed the thicker ONL and more photoreceptor cells in the YC-001–treated eyes versus control (Figures 10, I–K, and Supplemental Figure 14). Compared with WT control, the YC-001–treated eyes still showed much lower scotopic ERG a-wave and thinner ONL and OS (Figure 10, C, D, G, and H). As the compound disappeared completely between 2 and 3 weeks after IVI, the ONL thickness and ERG responses showed no difference between the YC-001–injected eyes versus control eyes after 5 weeks of IVI (data not shown).

**Figure 11:**
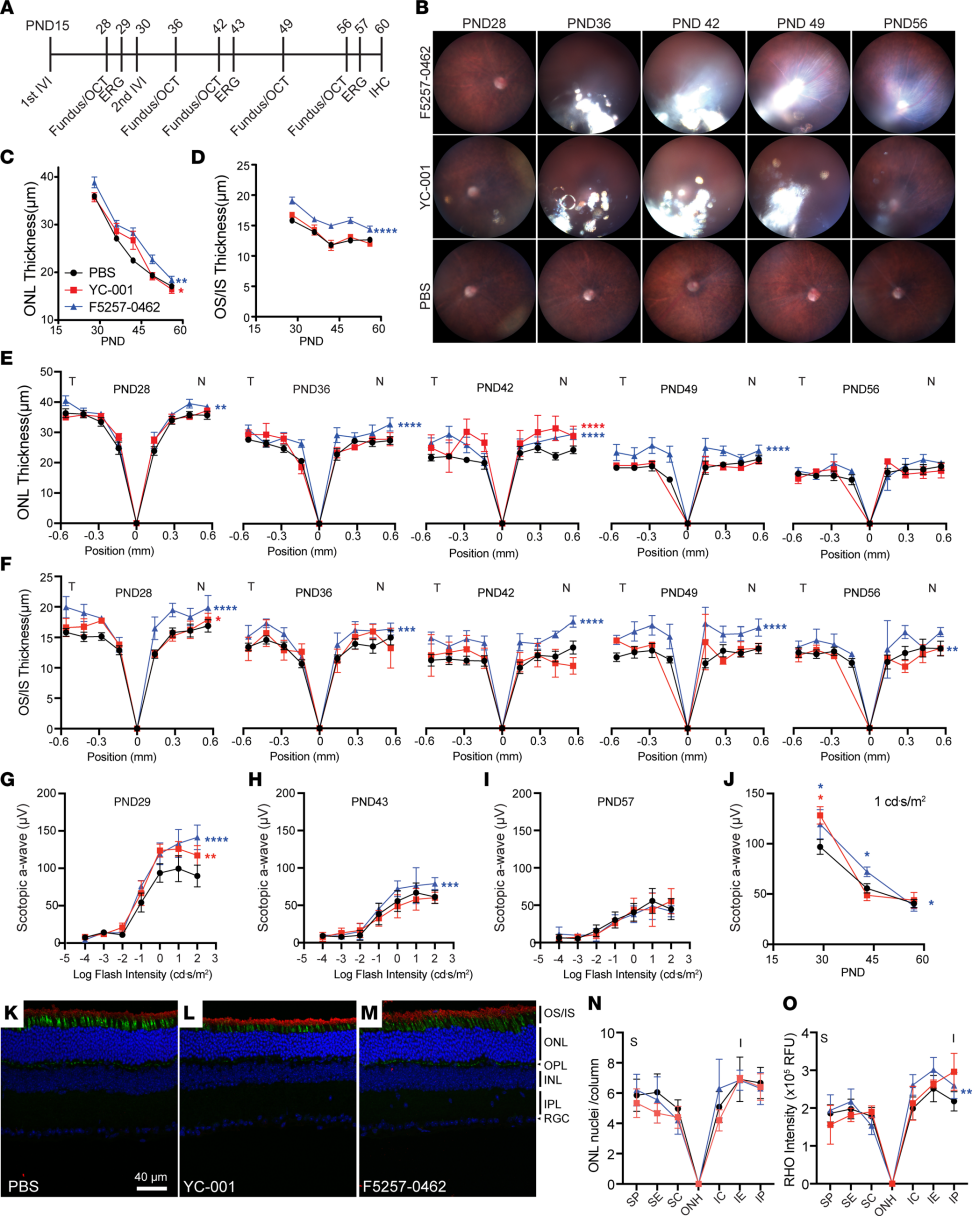
Retinal protection in the *Rho^P23H/+^* mice by small-molecule chaperons requires constant drug availability. (**A**) Timeline of the experimental procedures. *Rho^P23H/+^* mice were treated with 35 nmol of YC-001 or 25 nmol of F5257-0462 nanoparticles suspended in PBS by 2 IVIs at P15 and P30. Fundus imaging and OCT scanning were performed at P28, P36, P42, P49, and P56. ERGs were taken at P29, P43, and P67. Eyes were taken for IHC at P60. (**B**) Fundus images of animals at different time points. (**C** and **D**) Time-dependent changes of ONL and OS/IS thicknesses, respectively, measured by OCT scanning. Data are shown as mean ± SEMs Black circles, red squares, and blue triangles are PBS-, YC-001–, and F5257-0462–treated eyes, respectively. *n =* 5. *, **, ***, and ****, *P* < 0.05, 0.01, 0.001, and 0.0001, respectively, calculated by 2-way ANOVA. Red and blue asterisks show significant differences between YC-001– versus PBS-treated eyes, and between F5257-0462– versus PBS-treated eyes, respectively. (**E** and **F**) Spidergrams of ONL and OS/IS thickness, measured from OCT scanning at P28, P36, P42, P49, and P56. T, temporal; N, nasal. (**G**–**I**) Multiflash scotopic ERG a-wave responses from animals at P29, P43, and P57. (**J**) Time-dependent change of scotopic ERG a-wave responses stimulated by 1 cd·s/m^2^ flash. On top of each time point, * signifies *P* < 0.05 by Mann Whitney *U* test. By the side, * signifies *P* < 0.05 by 2-way ANOVA. (**K**–**M**) Immunofluorescence images of retinal cross-sections from PBS-, YC-001–, and F5257-0462–treated eyes. Red, rhodopsin staining; green, PNA for staining of cones; blue, Hoechst33343 staining for nucleus. (**N** and **O**) ONL nucleus number/column and rhodopsin immunofluorescence intensity, respectively, measured at peripheral (P), equatorial (E), and central (C) regions on superior (S) and inferior (I) sides from IHC images of retinal cross-sections. OS/IS, outer/inner segments; ONL, outer nuclear layer; OPL, outer plexiform layer; INL, inner nuclear layer; IPL, inner plexiform layer; RGC, retinal ganglion cells. *n =* 3–4. **P <0.01 by 2-way ANOVA comparing F5257-0462 treated vs. PBS group.

We then evaluated the efficacy of YC-001 and F5257-0462 by 2 IVIs at P15 and P30. Each IVI delivered 35 nmol YC-001 or 25 nmol F5257-0462 as a slurry of microparticles in PBS (Figure 11A). While the compounds from the first shot were mostly dissolved at P28, they stayed in the vitreous space for a longer time after the second shot, until P49–P56, when they were mostly dissolved (Figure 11B). OCT scanning showed that the ONL thickness was most prominently higher than PBS controls at P42 for both F5257-0462– and YC-001–treated eyes, compared with other time points. While YC-001–treated eyes showed significantly thicker ONL only at P42, we found that F5257-0462–treated eyes showed thicker ONL at P28, P36, P42, and P49, but not at P56 (Figure 11, C–E), compared with PBS control eyes. A slightly thicker OS/IS layer was seen in the YC-001 group only at P28, while F5257-0462 led to significantly thicker OS/IS layer at all time points of OCT measurements (Figure 11, D and F). Consistent with the effects on OS/IS thickness, YC-001–treated eyes showed higher ERG scotopic a-wave responses only at P29, whereas F5257-0462–treated eyes showed higher scotopic a-wave responses at P29 and P43, but not P57 (Figure 11, G–J). IHC of retinae collected at P60 (1 month after second IVI) showed that ONL nuclei numbers in both treatment groups were similar to the PBS control group, whereas the RHO level in the OS was significantly higher in the F5257-0462–treated eyes than in the control group (Figure 11, K–O, and Supplemental Figure 15). YC-001–treated eyes did not show a difference in RHO level. Collectively, we showed that F5257-0462, with a higher potency than YC-001 in vitro (Figure 1), also showed higher efficacy in vivo on preserving RHO level, OS thickness, rod photoreceptor function, and survival. However, the efficacy of both compounds was no longer visible in the eyes at P56–P60, when the compounds are fully dissolved and cleared out. This result shows that the efficacy of retinal protection by a pharmacological chaperone of RHO requires consistent retinal bioavailability of the agent. Compared with PBS control, no significant toxicity was observed in the retinae of *Rho^P23H/+^* mice at P60 after 2 IVIs of YC-001 or F5257-0462 by TUNEL staining (Supplemental Figure 16), suggesting the procedure and treatments are safe.

## Discussion

In this study, we characterized the pharmacological chaperone activity of YC-001 and F5257-0462 using in vitro, ex vivo, and in vivo models of *RHO*-associated RP. Firstly, our high-content imaging data in vitro suggest that the structural stability of the RHO mutants on the extracellular loops is varied, reflected by their different cell surface levels and their susceptibility to rescue by pharmacological chaperones. Additionally, our pharmacological profiles of these hRHO mutants can be used for selecting RP patients who may benefit from this type of therapy in the future.

Although 9-*cis*-retinal, under darkness, rescues the highest number of RHO mutants in vitro by forming the isorhodopsin pigment, light exposure leads to its photobleaching and production of apo-opsin mutant misfolding in the OS. Instead, the nonretinoid chaperones can provide additional structural support for apo-opsins independent of light. F5257-0462 showed a higher potency and rescued more RHO mutants than YC-001 in vitro, suggesting that it is a better drug candidate. These nonretinoid chaperones would, thus, be useful in rescuing not only the misfolding mutants that accumulate in ER, but also those that lack structural stabilities after photobleaching, such as T4K and T17M (32). Whereas T4K affects N2 glycosylation, T17M affects the glycosylation on N15 (47). Cell surface RHO^T4K^ was already seen in the DMSO group, which was increased by YC-001 and F5257-0462, whereas the T17M mutant can only be detected on the cell surface with 9-*cis*-retinal treatment (Figure 1, A and B). This result suggests that the RHO^T17M^ is not as stable as the T4K mutant, which is consistent with a previous study showing that glycosylation on N15, but not N2, is essential for RHO structural stability (48). T17M hRHO causes sector degeneration and is reported to be properly transferred to OS in *Xenopus laevis* retinae (32). The discrepancy between our results and this report may be due to a higher proteostasis capacity in the photoreceptor cell than the fibroblast.

We also noticed the difference of RHO between species and their sensitivity to chaperones. For example, the efficacy of YC-001 on rescuing the cell surface transport of hRHO^P23H^ (Figure 1, A and B) was not as high as that on mRHO^P23H^, as we reported (26). A recent study using the Förster resonance energy transfer assay also showed that the aggregation properties of bovine and human P23H RHO are different (49). Even though mouse, bovine, and hRHO proteins are highly conserved in sequence and structure, these subtle differences cannot be ignored and should be considered when generating disease models for drug development.

Chemical chaperones such as 4-phenylbutarate (4-PBA) showed nonselective chaperone activities toward different misfolded protein targets (50–53), including RHO (54, 55). However, the potency of target-specific pharmacological chaperones is much higher (μM versus mM levels), providing potential for less side effects and better safety. Mattle et al. identified a group of ligands that stabilize the human rod opsin structure closer to the activated Meta II state (56). Additionally, retinoid analogues including 5,8-epoxy-13-*cis* retinoic acid (57) and SRD005825 (58) have been shown to have potent chaperone activities in vitro. Oral vitamin A supplementation (59) or oral SRD005825 (58) have also shown retinal protection in the T17M transgenic mice. Collectively, multiple chemical scaffolds are currently available for the future development of more potent pharmacological chaperones of RHO. Our study, for the first time to our knowledge, thoroughly shows that well-characterized nonretinoid chaperones of RHO indeed improve RHO homeostasis in the *Rho^P23H/+^* retinae.

YC-001 rescues RHO homeostasis in mouse *Rho^P23H/+^–*knock-in retinal explants observable in the following aspects: (a) increased total RHO protein level, (b) increased RHO level in the OS, (c) improved glycosylation profile of RHO, and (d) reduced ubiquitinated to total RHO ratio. Interestingly, YC-001 increased RHO level in the WT retinae. The ex vivo culture led to reduced OS length and RHO staining intensity even in WT retinae (Supplemental Figure 5), potentially because the explants lack sufficient 11-*cis*-retinal to stabilize RHO^WT^. Thus YC-001 stabilized RHO in the WT retinal explant culture (Supplemental Figure 5) via its chaperone activity. Additionally, YC-001 reduced RHO level in the *Rho^P23H/P23H^* retinae, but it increased RHO level in the *Rho^P23H/+^* retinae. Collectively, it is likely that the stabilized RHO by YC-001 in the heterozygous retinae is mainly the RHO^WT^, but not RHO^P23H^. Thus, the improved RHO homeostasis in the YC-001–treated *Rho^P23H/+^* retinal explants could be due to more RHO^WT^. Coaggregation between RHO^WT^ and RHO^P23H^ leads to their codegradation and reduced RHO level, so YC-001 may also improve the RHO homeostasis by dissociating the RHOWT:RHOP23H aggregates. Additionally, YC-001 may increase the degradation of the aggregated RHO^P23H^ because YC-001 increased the percentage of RHO^P23H^ in the OS of *Rho^P23H/P23H^* retinae (Figure 4) without affecting Rho transcription. Due to the technical challenge of purifying P23H opsin (60), we have no direct evidence that these compounds stabilize the mutant opsin protein, although they rescue the cellular transport of RHO^P23H^ and stabilize WT opsin (26). We did not see the chaperone activity by 9-*cis*-retinal in the *Rho^P23H/+^* retinae kept in dark, either (Supplemental Figure 7). These discrepancies may be due to the differences between cell culture and retinal tissue and need to be further investigated.

RNA-Seq data, immune cell profiling, and immunostaining of macrophage markers showed that YC-001 modulates retinal immune responses. Because the retinal explants lack the infiltrated macrophages from blood, the immune responses are mainly attributed to the residential microglia. YC-001 substantially reduced the number of CD11b^+^ cells and CD68^+^ cells, indicating that its effect on inhibiting macrophage activation/infiltration (Figure 9) is even higher than its efficacy on chaperoning RHO in the retinal explants (Figures 4, 5, and 6). This finding indicates that YC-001, independent of its role as a chaperone, is also a potent inhibitor of macrophages and retinal immune responses. Because macrophages have high LC3-associated phagocytotic activities utilizing lapidated LC3 and SQSTM/p62 (61), the reduced autophagy flux by YC-001 in the *Rho^P23H/+^* retinal explants (Figure 6, K–N) may be due to reduced number of macrophages and inhibited macrophage activities. One future direction includes in-depth medicinal chemistry studies to develop more potent YC-001 derivatives without the activity of immune response modulation.

Our in vivo results show that 2 IVIs of YC-001 yielded a higher efficacy in rescuing *Rho^P23H/+^* retinae than 1 IVI, although effects were transient. F5257-0462, administered at a smaller dose, showed a higher efficacy in rescuing OS thickness, RHO level, rod function, and ONL thickness compared with the effects of YC-001. This in vivo result is consistent with our in vitro data that show that F5257-0462 is a more potent drug candidate (26). Although the retinal protection we show here does not last long, our comprehensive data set clearly proves the concept that misfolded *RHO-*associated RP mutations can be mitigated by nonretinoid chaperones. Our results also highlight a challenge for long-term pharmacological chaperone treatments that the drug should have continual retinal bioavailability. Thus, a future direction is to develop a formula or retinal-targeted linker of the chaperones that can be effectively delivered to the retina by systemic or intraocular administration to maintain long-term efficacious concentrations.

By IVI of compounds in slurry, we provided sufficient chaperone molecules to the retinae for slow release. Each mouse retinae contains about 520 pmol RHO protein (62), reaching a millimolar concentration in the OS. Supplied by RPE cells, the ratio of 11-*cis*-retinal to rod opsin protein level is about 1:1 in the native retina (63); thus, the retina has already provided endogenous support to RHO folding. For the adRP condition, the RHO^P23H^/RHO^WT^ ratio is less than 1:10 because most of RHO^P23H^ was degraded (37). RHO forms homodimers (64, 65), and thus, less than 10% of RHO^WT^ may dimerize with RHO^P23H^ and forms insoluble aggregates (15). Supplementation with a nonretinoid chaperone, such as YC-001 or F5257-0462, can provide additional structural support for the apo-opsin during its biosynthesis and after photobleach. Therefore, it is reasonable that we observed rescued RHO homeostasis even with 40 μM YC-001 in the *Rho^P23H/+^* retinal explants (Figure 5). The solubility of YC-001 and F5257-0462 is between 40 and 80 μM, and these numbers can be maintained when the insoluble particles remain in the vitreous. In total, the 25–35 nmol of YC-001 or F5257-0462 (>50 times of total RHO/retina) is sufficient for maintaining a saturating concentration of the compound in the retina in a slow-release manner. The more potent F5257-0462 showed higher efficacy than YC-001 in vivo, potentially due to higher binding affinity to opsin.

Here, we have illustrated clear proof-of-principle for nonretinoid RHO-binding molecules as a viable strategy for treating *RHO*-associated RP. Given the tractable nature of small-molecule pharmacologic treatment, YC-001 and F5257-0462 show great promise for this inherited retinal disease. Moving forward, our goals will be focused on taking these promising molecules into a preclinical candidate.

## Methods

Supplemental Methods are available online with this article and reagents are listed in Supplemental Table 3.

### Animals.

The C57BL/6J (*Rho*^+/+^) (stock no. 000664) and (*Rho*^P23H/P23H^) knock-in (stock no. 017628) mice (37) were purchased from the Jackson Laboratory and maintained under standard 12-hour light/12-hour dark conditions. Crossing *Rho*^+/+^ with *Rho*^P23H/P23H^ animals generated the *Rho*^P23H/+^ mice. Mice were anesthetized by i.p. injection of ketamine/xylazine cocktail containing 80 mg/kg body weight (bw) ketamine and 7 mg/kg bw xylazine (Henry Schein).

### Isolation and ex vivo culture of mouse retinal explants.

The procedure of mouse retinal ex vivo culture has been described previously (66, 67). Briefly, mice were euthanized at P15, and eyes were enucleated and incubated in Ames’ solution containing L-cysteine (0.22 mM) and papain (20 U/mL) at 37°C for 30 minutes, followed by incubation in DMEM with 10% FBS at 4°C for 5 minutes. Each eye cup was made and flattened by 4 cuts, and sclera was removed leaving only RPE attached to retina. Each retinal explant (RPE layer facing down) was cultured in a transwell (140640, Thermo Fisher Scientific) in a 6-well plate with neurobasal plus medium containing 2% B27 supplement and 5 μg/mL of plasmocin at 37°C with 5% CO_2_ for 24 hours. The retinal explants were then treated with the same medium containing compounds or DMSO. Fresh medium with the compound was changed daily. For 9-*cis*-retinal treatment, the compound was added under dim red light, and the culture plate was wrapped with foil during the entire time to avoid light exposure.

### Microglia profiling by flow cytometry.

Retinal explants isolated at P15 were treated with 40 μM YC-001 or 0.1% DMSO for 9 DIV and then dissociated into single-cell suspension using neural tissue dissociation kit for postnatal neurons following manufacturer instructions; fresh eyes at P25 were dissociated and processed immediately. The single-cell suspensions of retinal cells were incubated with Zombie UV viability dye and Fc Block in 1× PBS for 15 minutes at 4°C, followed by an incubation with cocktails of fluorescent antibodies for microglia profiling, diluted in Brilliant Stain Buffer for 30 minutes at 4°C. Fluorescent antibodies include CD11b (PE/Dazzle 594), F4/80 (APC), and MHCII I-A/I-E (BV510) (all from Biolegends). Stained cells were washed with PBS + 2% FBS and then fixed and permeabilized using BD Pharmingen cytofix/cytoperm, followed by staining with fluorescent antibodies of the intracellular proteins, iNOS (eF450, ThermoFisher Scientific) and CD68 (BV785, Biolegends). After 30 minutes of incubation at 4°C, cells were washed in Pharmingen Permwash and resuspended in PBS + 2% FBS. Stained samples were acquired on the Cytoflex LX (Beckman Coulter) and analyzed using Flowjo v10 (Flowjo LLC). Flow gates were set according to fluorescence minus one (FMO) control.

### IVI.

IVI was performed as described previously (68). Briefly, 20 mg/mL of pure YC-001 or F5257-0462 suspensions in PBS were sonicated on ice to generate a slurry of YC-001 or F5257-0462 microparticles. The stability of the compounds after sonication was confirmed by HPLC. The suspension of each compound was pushed through an insulin needle several times for homogenization before loading in the Hamilton syringe (33 G). Pupils were dilated with 1% tropicamide and 2.5% phenylephrine drops. A tetracaine (0.5%) eye drop was applied to each eye followed by 1 drop of Genteal lubricant eye gel for hydration. A heating pad was used to maintain body temperature. A puncture was made behind the limbus by a 30-gauge needle (Medline), and a 33-gauge blunt needle (Hamilton) was then fitted into the punctured hole to slowly deliver a total of 0.5 μL of sterile PBS or YC-001 (35 nmol) or F5257-0462 (25 nmol) slurry to the vitreous space. Tri-antibiotic ointment (Medline) was applied after injection. The left or right eye was randomly selected for injecting YC-001 or F5257-0462 or PBS to avoid lateral bias.

### Statistics.

Statistical analysis was performed by GraphPad Prism (v8). Multiple comparisons were performed by the Kruskal-Wallis test, followed by a multiple-comparison test. Multiflash ERG responses and multiposition ONL thickness measurements from OCT or H&E staining images were analyzed by 2-way ANOVA, with the treatment conditions as factor 1 and flash intensity or position to optic nerve head (ONH) as factor 2. Significant differences between treatments were claimed when *P* < 0.05. Data are shown as mean ± SD or mean ± SEM, as described in figure legends.

### Study approval.

Procedures on animals were approved by the University of Pittsburgh IACUC complying with the NIH Policy on the Humane Care and Use of Laboratory Animals (IACUC protocol no. 20047092).

## Author contributions

AV and YC designed the study, analyzed the data, and drafted the manuscript. AV performed most of the experiments. YX performed the IVI, OCT, and ERG experiments. BF, ODC, and YC performed the high-content imaging and docking calculations. AJSL performed flow cytometry experiments. XL performed pharmacokinetics of YC-001. AG analyzed retinal images. CDD participated in sample processing, genotyping, and managing mouse colonies. KLL assisted in imaging and image analysis. GPT synthesized YC-001. SP guided the retinal explant culture. All authors participated in manuscript writing.

## Supplementary Material

Supplemental data

## Version 1 04/26/2022

In-Press Preview

## Version 2 05/23/2022

Electronic publication
