# Supplementary material for: Nonretinoid chaperones improve rhodopsin homeostasis in a mouse model of retinitis pigmentosa
Source: JCI Insight. 2022 May 23;7(10):e153717. doi: 10.1172/jci.insight.153717 (PMC9220944; doi:10.1172/jci.insight.153717)

Title: Non-retinoid chaperones improve rhodopsin homeostasis in a mouse model of retinitis pigmentosa

Abhishek Vats[123], Yibo Xi[123], Bing Feng[123], Owen D. Clinger[123], Anthony J. St. Leger[14], Xujie Liu[123], Archisha Ghosh[123], Chase D. Dermond[123], Kira L. Lathrop[15], Gregory P. Tochtrop[6], Serge Picaud[7], Yuanyuan Chen[123*]

Affiliations:
1. Department of Ophthalmology, University of Pittsburgh, Pittsburgh, PA 15213 USA
2. Department of Pharmacology and Chemical Biology, University of Pittsburgh, Pittsburgh, PA 15213 USA
3. McGowan Institute of Regenerative Medicine, University of Pittsburgh, Pittsburgh, PA 15213 USA
4. Department of Immunology, University of Pittsburgh, Pittsburgh, PA 15213 USA
5. Department of Bioengineering, University of Pittsburgh Swanson School of Engineering, Pittsburgh, PA 15213 USA
6. Department of Chemistry, Case Western Reserve University, Cleveland, OH 44106 USA
7. Sorbonne Université, INSERM, CNRS, Institut de la Vision, 17 rue Moreau, Paris, 75012 France

* Correspondence: Yuanyuan Chen

Address: BST-3 Rm 10043, 3501 Fifth Avenue, Pittsburgh, PA 15213 USA

Phone: +1 412-624-5444

Email: cheny1@pitt.edu

Supplementary Result:

Faster photoreceptors degeneration in *Rho*$^{P23H/+}$ retinal culture.

Photoreceptors of the *Rho*$^{P23H/+}$ mice degenerate quickly from 100% to 60% within the first month of age *in vivo* (1, 2). Here, we characterized retinal degeneration in the explant culture (**Figures S5 and S6**). Co-cultured with RPE, the OS layer marked by RHO immunofluorescence remained until 10 DIV (**Figure S5A-N**). The OS thickness and RHO level were consistently lower in the *Rho*$^{P23H/+}$ than the WT retinae, both in culture and *in vivo*, because most RHO^{P23H} was degraded (2) (**Figure S5P-S**). From 1 to 21 DIV, the ONL thickness of *Rho*$^{P23H/+}$ retinae degenerated at a faster rate and with a steeper curve of decay ($t_{1/2}$=2.0 days, amplitude of decay=44 µm, **Figure S5T&U**) than that of the WT retinae in culture ($t_{1/2}$= 4.2 days, amplitude of decay=25 µm), as well as *in vivo*. Therefore, the degeneration in the mouse Rho$^{P23H/+}$ retinal explant effectively recapitulates the progressive photoreceptor death seen *in vivo* and provides an *in vitro* tool for investigating the efficacy, safety and mechanism of actions of pharmacological agents in the retina.

Supplementary Tables

Table S1. Top 40 biological processes identified by Gene ontology (GO) analysis of differentially expressed genes (DEGs) comparing *Rho*$^{P23H/+}$ vs. *Rho*$^{+/+}$ retinal explants with DMSO at 1 days *in vitro* (DIV).

#	GO_ID	Description	Gene Ratio	Bg Ratio	*P* value
1	GO:0034097	response to cytokine	42/331	493/23313	9.80E-21
2	GO:0045087	innate immune response	38/331	469/23313	4.25E-18
3	GO:0071345	cellular response to cytokine stimulus	31/331	401/23313	2.16E-14
4	GO:0098542	defense response to other organism	30/331	482/23313	1.47E-11
5	GO:0050778	positive regulation of immune response	26/331	405/23313	1.82E-10
6	GO:0043900	regulation of multi-organism process	23/331	387/23313	9.11E-09
7	GO:0001817	regulation of cytokine production	26/331	492/23313	1.09E-08
8	GO:0002683	negative regulation of immune system process	21/331	363/23313	6.46E-08
9	GO:0031349	positive regulation of defense response	16/331	240/23313	3.75E-07

10	GO:0044403	symbiosis; encompassing mutualism through parasitism	17/331	274/23313	4.47E-07
11	GO:0044419	interspecies interaction between organisms	17/331	274/23313	4.47E-07
12	GO:0031589	cell-substrate adhesion	17/331	281/23313	6.36E-07
13	GO:0090257	regulation of muscle system process	13/331	190/23313	3.52E-06
14	GO:0072593	reactive oxygen species metabolic process	14/331	221/23313	3.67E-06
15	GO:0045785	positive regulation of cell adhesion	17/331	324/23313	4.42E-06
16	GO:0002521	leukocyte differentiation	21/331	474/23313	4.86E-06
17	GO:0040017	positive regulation of locomotion	19/331	406/23313	6.38E-06
18	GO:0051250	negative regulation of lymphocyte activation	10/331	120/23313	8.49E-06
19	GO:0050663	cytokine secretion	11/331	147/23313	8.51E-06
20	GO:0030509	BMP signaling pathway	11/331	149/23313	9.68E-06
21	GO:0002483	antigen processing and presentation of endogenous peptide antigen	4/331	11/23313	1.22E-05
22	GO:0051051	negative regulation of transport	20/331	465/23313	1.26E-05
23	GO:0045446	endothelial cell differentiation	8/331	78/23313	1.50E-05
24	GO:0007162	negative regulation of cell adhesion	13/331	219/23313	1.64E-05
25	GO:1901342	regulation of vasculature development	13/331	222/23313	1.89E-05
26	GO:0052547	regulation of peptidase activity	17/331	366/23313	2.16E-05
27	GO:0010035	response to inorganic substance	17/331	371/23313	2.56E-05
28	GO:0002250	adaptive immune response	16/331	339/23313	3.10E-05
29	GO:1902105	regulation of leukocyte differentiation	13/331	233/23313	3.13E-05
30	GO:0030198	extracellular matrix organization	12/331	203/23313	3.60E-05
31	GO:0003158	endothelium development	8/331	88/23313	3.64E-05
32	GO:0044764	multi-organism cellular process	13/331	237/23313	3.74E-05
33	GO:0009583	detection of light stimulus	6/331	46/23313	4.54E-05
34	GO:0010951	negative regulation of endopeptidase activity	11/331	179/23313	5.33E-05
35	GO:0006979	response to oxidative stress	15/331	318/23313	5.50E-05
36	GO:0051272	positive regulation of cellular component movement	17/331	395/23313	5.60E-05
37	GO:0042886	amide transport	15/331	321/23313	6.12E-05
38	GO:0000302	response to reactive oxygen species	10/331	152/23313	6.58E-05
39	GO:0014074	response to purine-containing compound	8/331	97/23313	7.33E-05
40	GO:0032606	type I interferon production	6/331	50/23313	7.35E-05

Note: GeneRatio, ratio of number of DEGs in the corresponding GO to the total number of DEGs; BgRatio, ratio of the total number of genes in the corresponding GO to the total number of genes in mouse genome.

Table S2. Top 30 biological processes identified by Gene ontology analysis of DEGs comparing YC-001- *vs.* DMSO-treated *Rho*$^{P23H/+}$ retinal explants at 1 DIV.

No.	GO_ID	Description	Gene Ratio	Bg Ratio	*P* value
1	GO:0035458	cellular response to interferon-beta	3/051	30/23313	3.84E-05
2	GO:0001886	endothelial cell morphogenesis	2/051	12/23313	0.000305
3	GO:0030336	negative regulation of cell migration	4/051	194/23313	0.000856
4	GO:0050673	epithelial cell proliferation	5/051	346/23313	0.000939
5	GO:2000146	negative regulation of cell motility	4/051	203/23313	0.001013
6	GO:0006760	folic acid-containing compound metabolic process	2/051	24/23313	0.001256
7	GO:0051271	negative regulation of cellular component movement	4/051	216/23313	0.001274
8	GO:0010811	positive regulation of cell-substrate adhesion	3/051	99/23313	0.001334
9	GO:0007565	female pregnancy	3/051	112/23313	0.001901
10	GO:0040013	negative regulation of locomotion	4/051	242/23313	0.001931
11	GO:0042558	pteridine-containing compound metabolic process	2/051	31/23313	0.002095
12	GO:0031069	hair follicle morphogenesis	2/051	34/23313	0.002517
13	GO:0071320	cellular response to cAMP	2/051	36/23313	0.002819
14	GO:0006732	coenzyme metabolic process	4/051	271/23313	0.002906
15	GO:0006935	chemotaxis	5/051	458/23313	0.0032
16	GO:0042330	taxis	5/051	459/23313	0.003229
17	GO:0048730	epidermis morphogenesis	2/051	39/23313	0.003301
18	GO:0031589	cell-substrate adhesion	4/051	281/23313	0.003309
19	GO:0045087	innate immune response	5/051	469/23313	0.00354
20	GO:0032924	activin receptor signaling pathway	2/051	42/23313	0.00382
21	GO:0001523	retinoid metabolic process	2/051	43/23313	0.004001
22	GO:0010828	positive regulation of glucose transport	2/051	44/23313	0.004186
23	GO:0016101	diterpenoid metabolic process	2/051	45/23313	0.004374
24	GO:0001764	neuron migration	3/051	151/23313	0.004419
25	GO:0050920	regulation of chemotaxis	3/051	156/23313	0.004838
26	GO:0044706	multi-multicellular organism process	3/051	162/23313	0.005373
27	GO:0006006	glucose metabolic process	3/051	171/23313	0.006239
28	GO:0006575	cellular modified amino acid metabolic process	3/051	178/23313	0.006967
29	GO:0006094	gluconeogenesis	2/051	58/23313	0.007172
30	GO:0032872	regulation of stress-activated MAPK cascade	3/051	180/23313	0.007183

Supplementary Figures

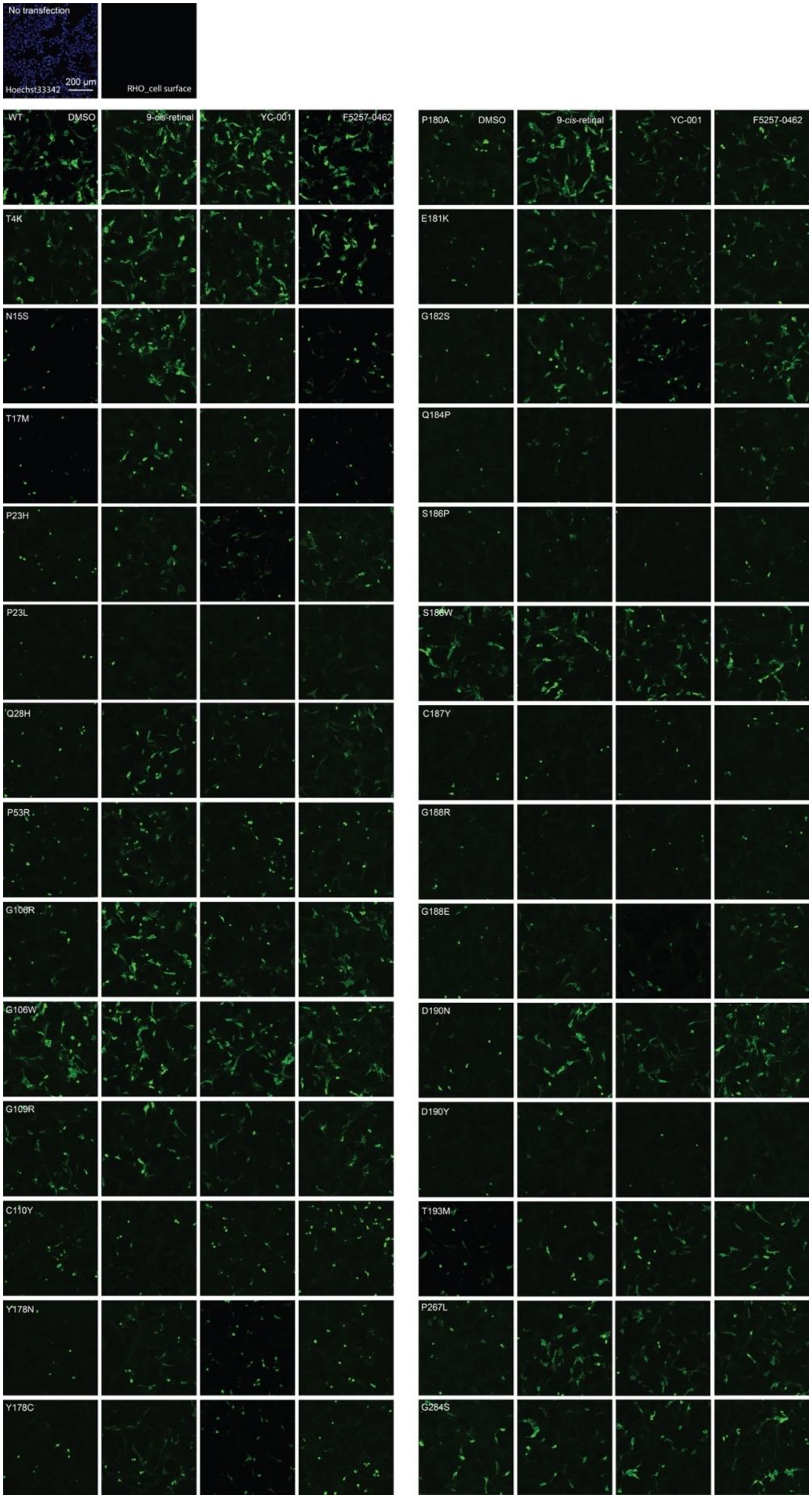
No transfection
Hoechst33342
200 μm
RHO_cell surface
WT
DMSO
9-cis-retinal
YC-001
F5257-0462
T4K
N15S
T17M
P23H
P23L
Q28H
P53R
G106R
G106W
G109R
C110Y
Y178N
Y178C
P180A
DMSO
9-cis-retinal
YC-001
F5257-0462
E181K
G182S
Q184P
S186P
S186W
C187Y
G188R
G188E
D190N
D190Y
T193M
P267L
G284S

Figure S1. High-content images of cell-surface staining of all RHO-RP mutants. RHO cell surface staining on the extracellular side of the chromophore pocket, transfected in NIH3T3 cells for 24 h followed by treatment with DMSO (0.1%), 5 µM 9-*cis*-retinal, 40 µM YC-001, or 20 µM F5257-0462 for 24 h. Scale bar, 200 µm. Untransfected cells showed no cell-surface stain of RHO in the top panels as control. Cell surface intensities of RHO mutants and representative images were shown in **Figure 1.**

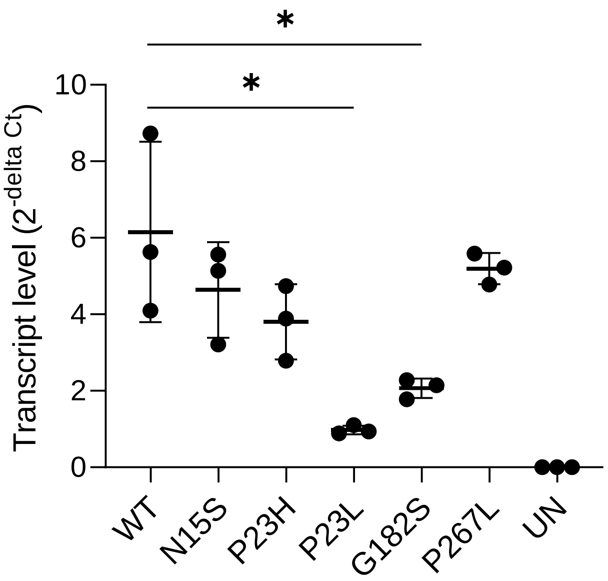

Figure S2. Transcript levels of RHO mutants in NIH3T3 cells by qPCR. NIH3T3 cells were transiently transfected with WT and six randomly selected mutant RHO clones. Total RNA was isolated after 48 h of transfection followed by cDNA synthesis, using 1 µg of total RNA. The graph represents the transcript levels of WT and mutant RHO calculated using $2^{-delta\ Ct}$ method. Ten nano gram of cDNA was used for qPCR and beta-actin was the endogenous control. Middle and error bars are means±SD. N=3 Mann Whitney U test was performed to determine statistical significance. *, $P<0.05$ by Kruskal-Wallis test. Only RHO P23L and G182S were expressed at significantly lower levels, due to cell spontaneous suppression of expression triggered by the potential cytotoxicity of these mutants, while the remaining mutants showed similar expression level compared to WT RHO transfected control.

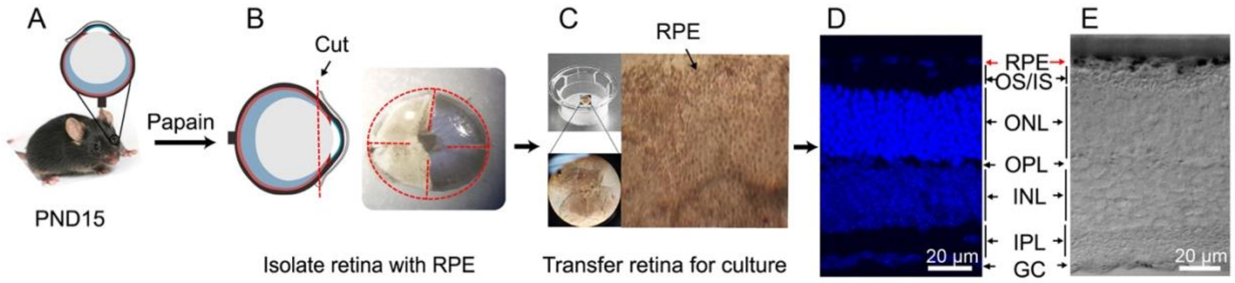

Figure S3. Schematic procedure for the *ex vivo* retinal culture. A. Mice were euthanized at postnatal day (PND) 15 and eyes were enucleated and treated with papain to dissociate the retinal pigmented epithelia (RPE) from sclera. **B.** Eye cup was made by a circular cut (left) behind the limbus, followed by removing lens, and vitreous body, and the sclera, leaving the neural retina with the RPE (right). **C.** Isolated retina was transferred to a transwell with the RPE side facing the membrane (top left), and the isolated retinal explant showed RPE attaching to the neural retina at low (bottom left) and high (right) magnifications. **D** and **E.** The Hoechst 33342 staining of nuclei and bright field images of a cryosection from wildtype (WT) mouse retinal explant at 10 days *in vitro* (DIV), respectively, showed the RPE layer as well as the neural retina stayed intact. Scale bar, 20 µm.

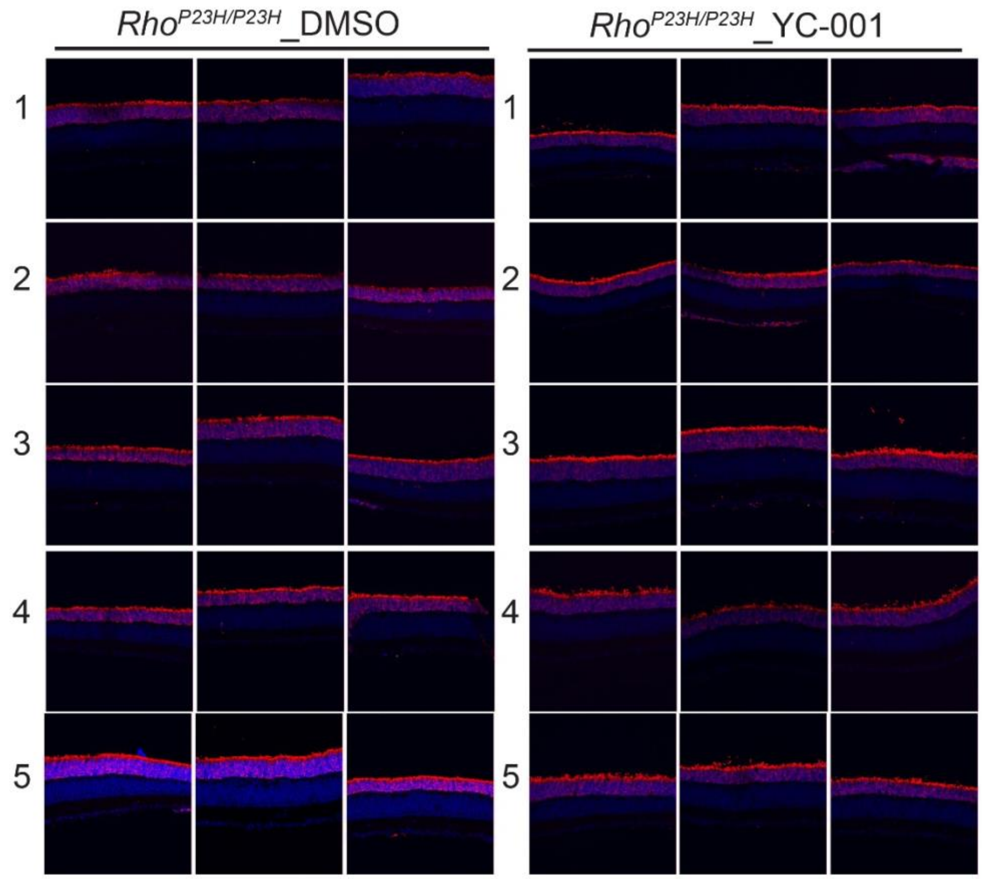

Figure S4. Immunofluorescence images of *Rho*$^{P23H/P23H}$ retinal explant. The immunostaining images of cryosections from all *Rho*$^{P23H/P23H}$ retinal explants treated with 40 µM YC-001 or DMSO. Scale bars, 40 µm. Red, rhodopsin; and blue, Hoechst33342. The number on the y-axis represents the replicates, N=5. Representative images and quantifications are shown in **Figure 5**.

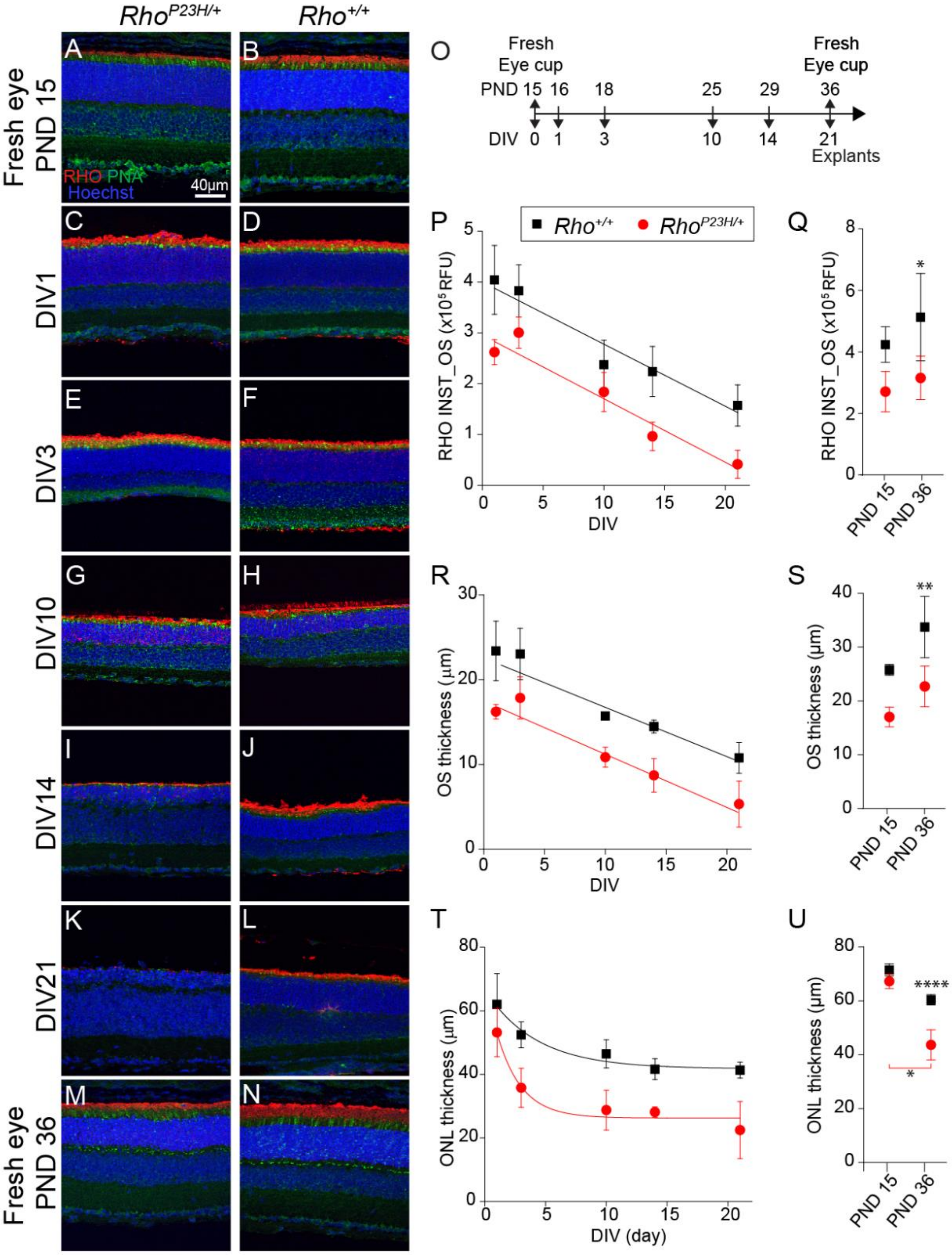

Rho^P23H/+
Rho^+/+
Fresh eye PND 15
RHO PNA
Hoechst
40μm
DIV1
DIV3
DIV10
DIV14
DIV21
Fresh eye PND 36
A B C D E F G H I J K L M N
O
Fresh Eye cup
Fresh Eye cup
PND 15 16 18 25 29 36
DIV 0 1 3 10 14 21
Explants
P
Rho^+/+
Rho^P23H/+
RHO INST_OS (x10^5 RFU)
DIV
Q
PND 15
PND 36
R
OS thickness (μm)
DIV
S
OS thickness (μm)
T
ONL thickness (μm)
DIV (day)
U
ONL thickness (μm)

Figure S5. Progressive degeneration in *Rho*$^{P23H/+}$ retinal explants. A-N. Immunofluorescence images of *Rho*$^{P23H/+}$ and *Rho*$^{+/+}$ retinal explants at 1 to 21 days *in vitro* (DIV) with fresh retinae at PND 15 and 36 as *in vivo* controls. Red, RHO; green, lectin peanut agglutinin (PNA) for cones; and blue, Hoechst 33342 for nucleus staining, respectively. Scale bar, 40 µM. **A**, **C, E, G, I, K,** and **M** are from *Rho*$^{P23H/+}$ mice, and **B, D, F, H, J, L,** and **N** are from *Rho*$^{+/+}$ mice. **O.** Schematic illustration of experiment design. **P** and **Q.** RHO intensity in the outer segment (OS) of retinal explants and fresh retinae, respectively. **R** and **S.** OS thickness of retinal explants and fresh retinae, respectively. **T** and **U.** Outer nuclear layer (ONL) thickness of retinal explants and fresh retinae, respectively. Black squares, *Rho*$^{+/+}$ retinae; red circles, *Rho*$^{P23H/+}$ retinae. Data and error bars are means±SDs. Data were fit linear in P and R, and with exponential decay in T. N=3. *, **, ****, P<0.05, 0.01, and 0.0001, respectively, by the Mann Whitney test.

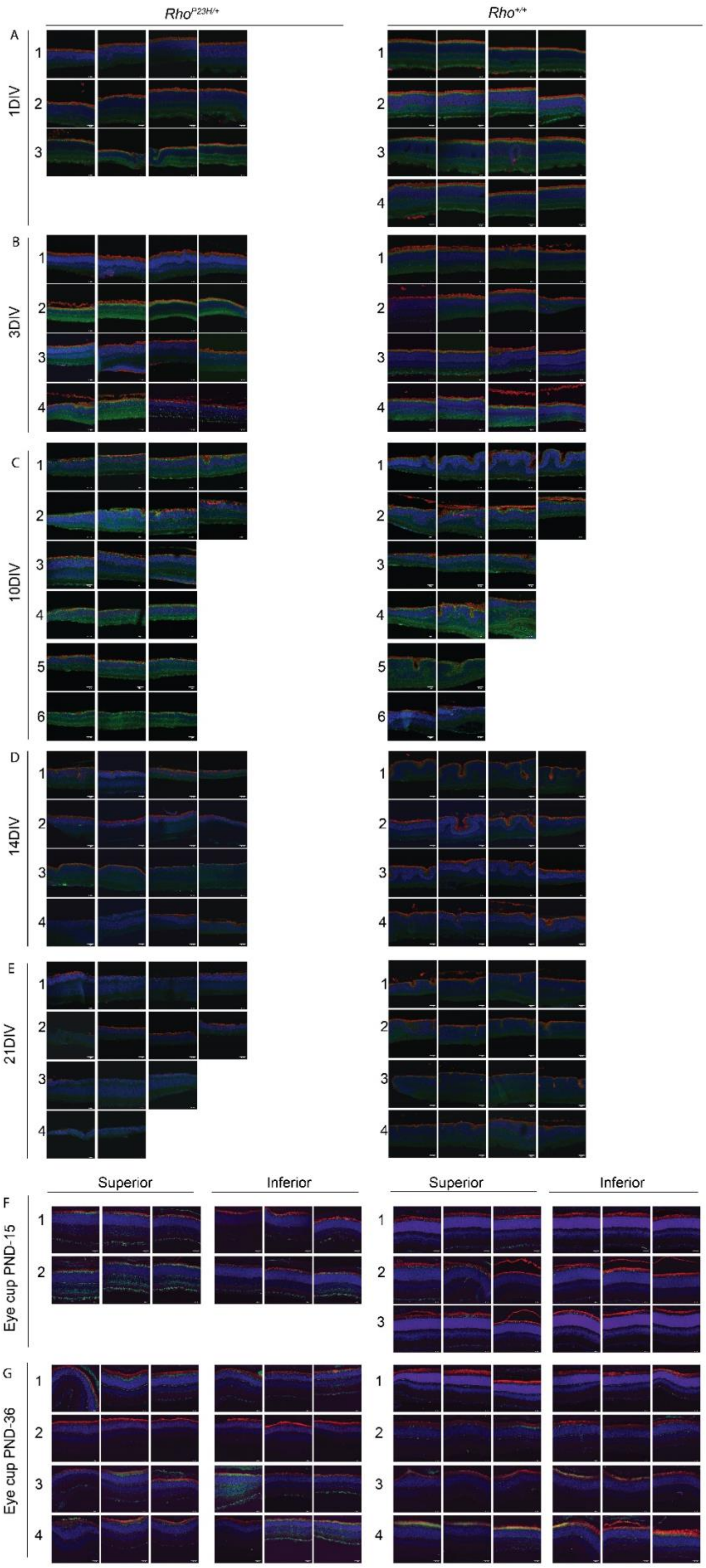

Figure S6. Immunostaining images from cryosections of *Rho*$^{P23H/+}$ and *Rho*$^{+/+}$ retinal explants at different DIV. A-E. Immunofluorescence images from *Rho*$^{P23H/+}$ (left panel) and *Rho*$^{+/+}$ (right panel) at 1, 3, 10, 14, and 21 DIV, respectively. **F and G.** represents the fresh eye cup at PND 15 and 36, respectively. Left two panels shows the superior and inferior part of *Rho*$^{P23H/+}$ retina and right two panels show the superior and inferior part of *Rho*$^{+/+}$ retina. Scale bars, 40 µm. Red, rhodopsin; green, PNA; and blue, Hoechst33342. The numbers on the left side of all images are replicates, N=2-6. Representative images and quantifications are shown in **Figure S5**.

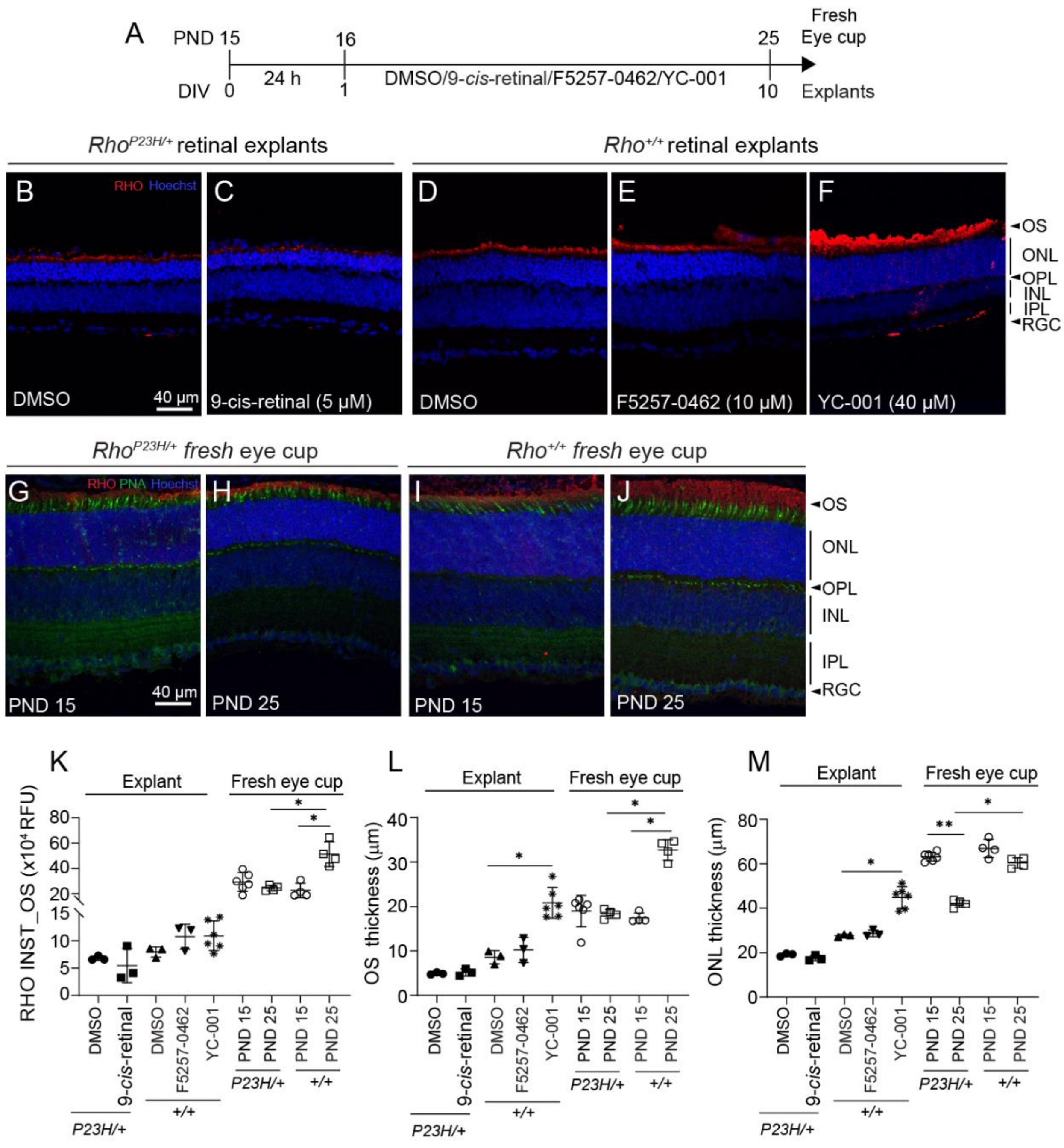

Figure S7. Effects of compounds in *Rho*$^{P23H/+}$ and *Rho*$^{+/+}$ retinal explants. Mouse (*Rho*$^{P23H/+}$ and *Rho*$^{+/+}$) retinal explants were isolated at PND 15 and cultured for 1 DIV followed by treatment with 5 µM 9-*cis*-retinal, 10 µM F5257-0462, 40 µM YC-001 or DMSO for 9 DIV, with medium changed every day. Fresh eye cups at PND15 and 25 were *in vivo* controls. **A.** Schematic illustration of the experiment design. **B** and **C.** Immunofluorescence (IF) images of *Rho*$^{P23H/+}$ treated with DMSO and 5 µM 9-*cis*-retinal, respectively. **D-F.** Immunofluorescence images of *Rho*$^{+/+}$ treated with DMSO **(D),** 10 µM F5257-0462 **(E)** and 40 µM YC-001 **(F)**. **G-J.** Immunofluorescence images of fresh retinae. **G and H.** Superior *Rho*$^{P23H/+}$ retinae at PND15 and

25, respectively. **I** and **J.** Superior mouse *Rho*$^{+/+}$ retinae at PND15 and 25, respectively. Red, RHO; blue, Hoechst 33342 for nucleus staining. Scale bar, 40 µm. **K**-**M.** Bar plots of Rho intensity in OS **(K)**, OS thickness **(L)** and ONL thickness **(M)** quantified using (IF) images in **B**-**J**. N=3-6. Scale bars, 40 µm. Data and error bars are means±SDs. * and **, P<0.05 and 0.01, respectively, by the Kruskal-Wallis test.

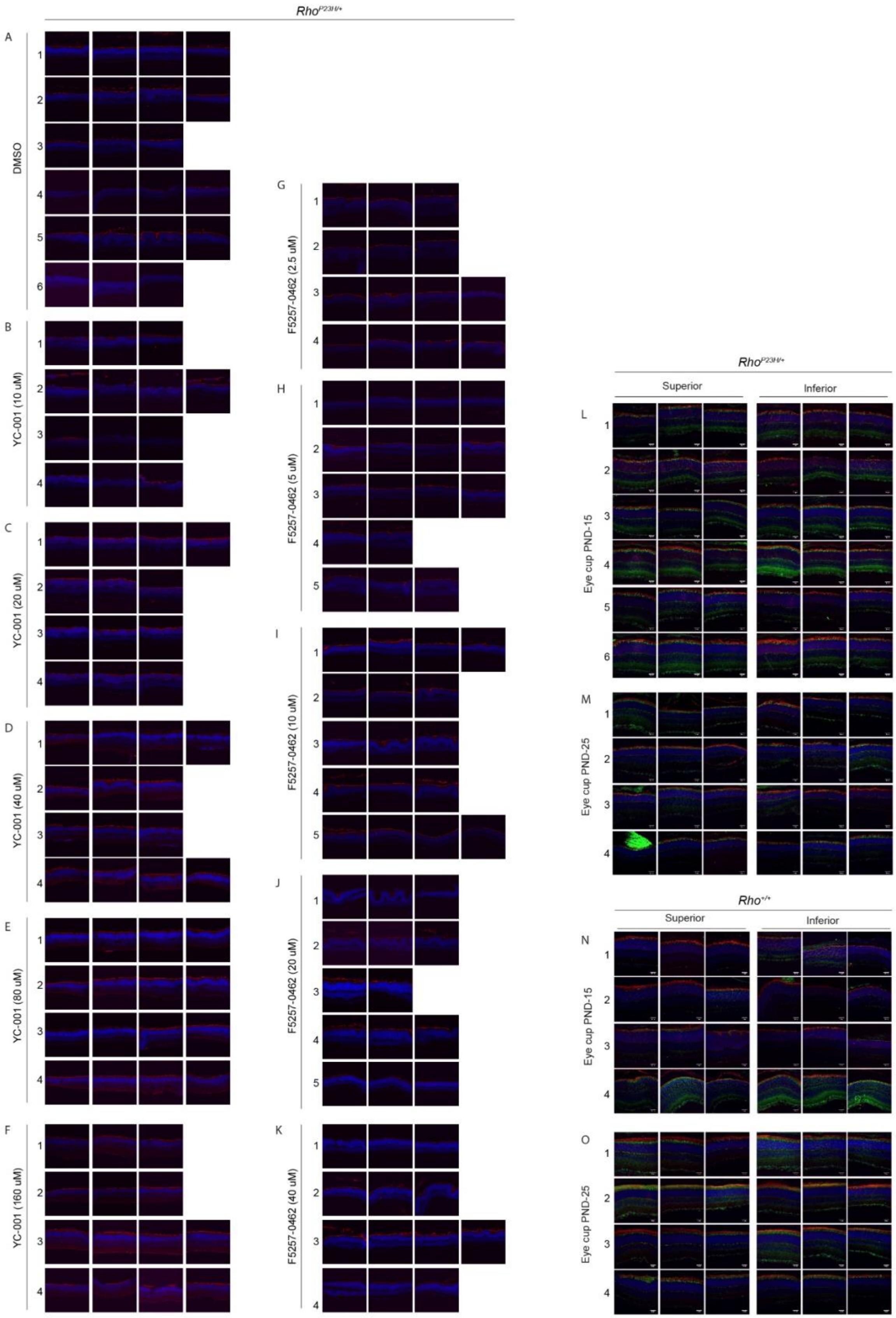

Rho^P23H/+
A DMSO 1 2 3 4 5 6
B YC-001 (10 uM) 1 2 3 4
C YC-001 (20 uM) 1 2 3 4
D YC-001 (40 uM) 1 2 3 4
E YC-001 (80 uM) 1 2 3 4
F YC-001 (160 uM) 1 2 3 4
G F5257-0462 (2.5 uM) 1 2 3 4
H F5257-0462 (5 uM) 1 2 3 4 5
I F5257-0462 (10 uM) 1 2 3 4 5
J F5257-0462 (20 uM) 1 2 3 4 5
K F5257-0462 (40 uM) 1 2 3 4
Rho^P23H/+
Superior
Inferior
L Eye cup PND-15 1 2 3 4 5 6
M Eye cup PND-25 1 2 3 4
Rho^+/+
Superior
Inferior
N Eye cup PND-15 1 2 3 4
O Eye cup PND-25 1 2 3 4

Figure S8. All the immunostaining images of *Rho*$^{P23H/+}$ with DMSO and YC-001, F5257-0462 at 5 does and eye cups at PND-15 and PND-25. Immunostaining images of cryosections from all *Rho*$^{P23H/+}$ retinal explants treated with DMSO **(A)**, YC-001 doses **(B-F)** and F5257-0462 doses **(G-K)**. **L** and **M** are the fresh eye cups of *Rho*$^{P23H/+}$ at PND15 and PND-25 respectively, **N** and **O** are eyecups of *Rho*$^{+/+}$ at PND-15 and PND-25 respectively. Scale bars, 40 µm. Red, rhodopsin; green, PNA; and blue, Hoechst33342. N=4-6. Representative images and quantifications are shown in **Figure 5 and Fig S7**.

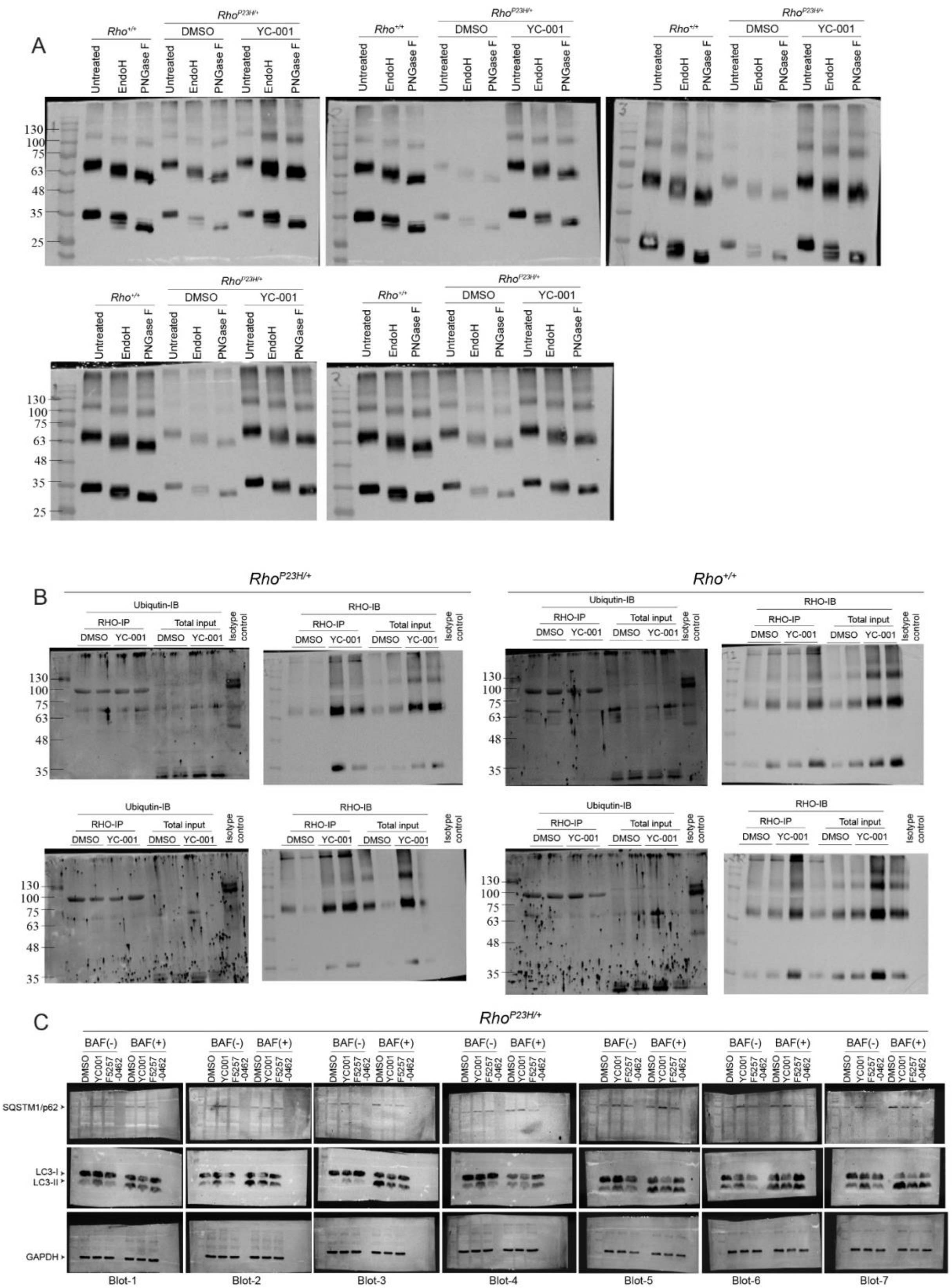
A
Rho+/+
RhoP23H/+
DMSO
YC-001
Untreated
EndoH
PNGase F
130
100
75
63
48
35
25
B
RhoP23H/+
Rho+/+
Ubiqutin-IB
RHO-IB
RHO-IP
Total input
Isotype control
DMSO
YC-001
C
BAF(-)
BAF(+)
DMSO
YC001
F5257 -0462
SQSTM1/p62
LC3-I
LC3-II
GAPDH
Blot-1
Blot-2
Blot-3
Blot-4
Blot-5
Blot-6
Blot-7

Figure S9. All the replicates of immunoblots used for Endo-H digestion assay (**A**, N=5) and RHO immunoprecipitation (**B**, N=4), and LC3B, SQSTM1 (**C**, N=7) using retinal explants, representative images and quantification are shown in **Figure 6**.

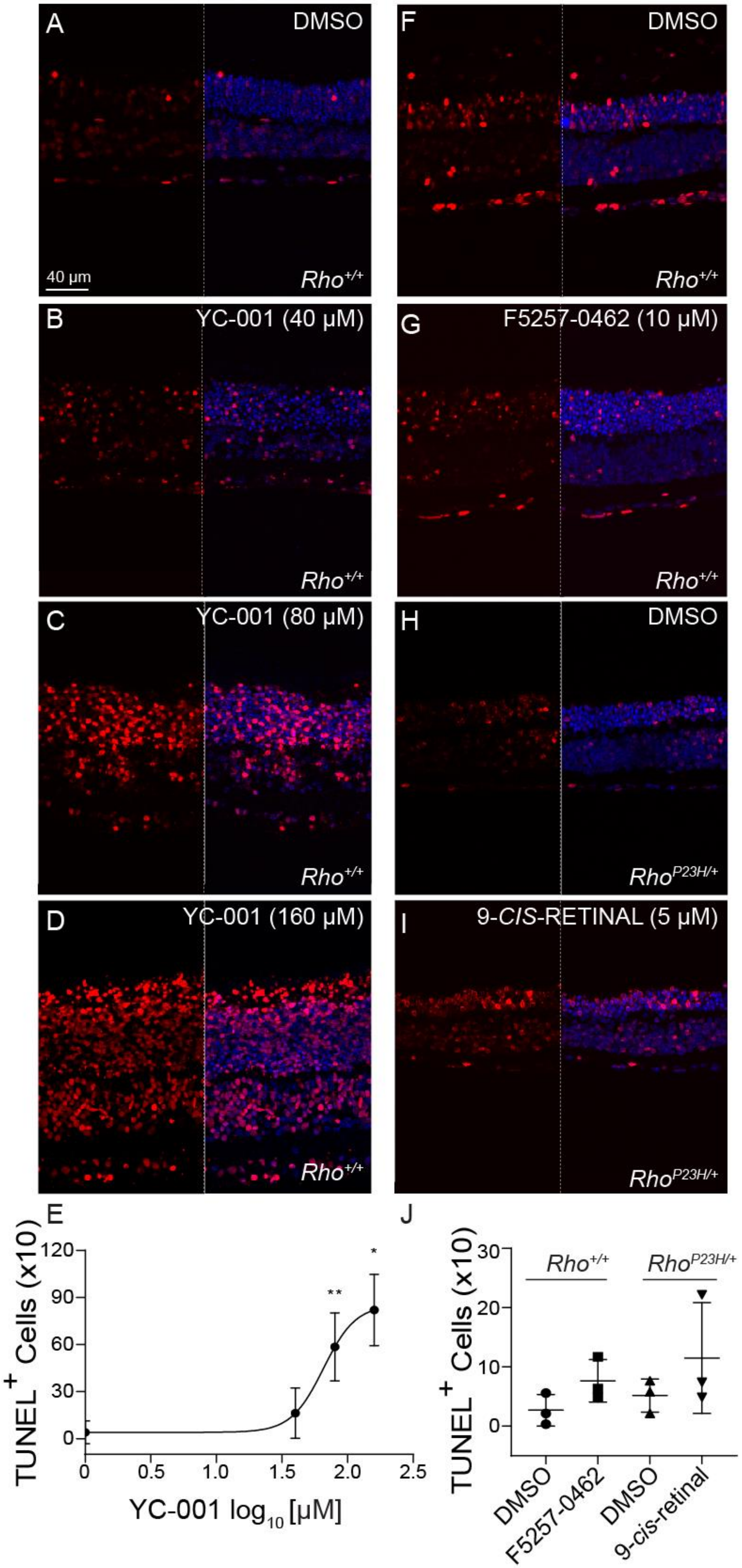

A
DMSO
40 µm
Rho+/+
B
YC-001 (40 µM)
Rho+/+
C
YC-001 (80 µM)
Rho+/+
D
YC-001 (160 µM)
Rho+/+
E
TUNEL+ Cells (x10)
120
90
60
30
0
0
0.5
1.0
1.5
2.0
2.5
YC-001 log10 [µM]
**
*
F
DMSO
Rho+/+
G
F5257-0462 (10 µM)
Rho+/+
H
DMSO
RhoP23H/+
I
9-CIS-RETINAL (5 µM)
RhoP23H/+
J
TUNEL+ Cells (x10)
30
20
10
0
Rho+/+
RhoP23H/+
DMSO
F5257-0462
DMSO
9-cis-retinal

Figure S10. TUNEL stain of WT retinal explant with YC-001 and F5257-0462. Terminal deoxynucleotidyl transferase dUTP nick end labeling (TUNEL) staining was performed to *Rho*$^{+/+}$ and *Rho*$^{P23H/+}$ mouse retinal explants isolated at PND 15 and treated with YC-001 (40, 80 and 160 µM) and F5257-0462 (10 µM) or DMSO for 9 DIV. Red, TUNEL^{+} cells; blue, Hoechst 33342 to stain the nucleus. Scale bars, 40 µm. **A-D.** TUNEL stained *Rho*$^{+/+}$ retinal explants treated with DMSO **(A)**, 40 **(B)**, 80 **(C)** and 160 µM **(D)** of YC-001. **E.** Bar plots of TUNEL^{+} cell number as a function of [YC-001] quantified using images from **A**-**D. F** and **G.** *Rho*$^{+/+}$ retinal explants treated with DMSO and 10 µM F5257-0462, respectively. **H** and **I.** *Rho*$^{P23H/+}$ retinal explants treated with DMSO and 5 µM 9-*cis*-retinal, respectively. **J.** Bar plots of TUNEL^{+} cell number quantified using images from **F**-**I**. The left panel of all immunofluorescence images represent the TUNEL staining (red) and right panel is composite image of TUNEL and Hoechst 33342 (blue)**.** Dose response curve was fitted by a modified Hill function. N=3-6. Middle and error bars are the means±SDs. * and **, $P<0.05$ and 0.01, respectively, by the Kruskal-Wallis test.

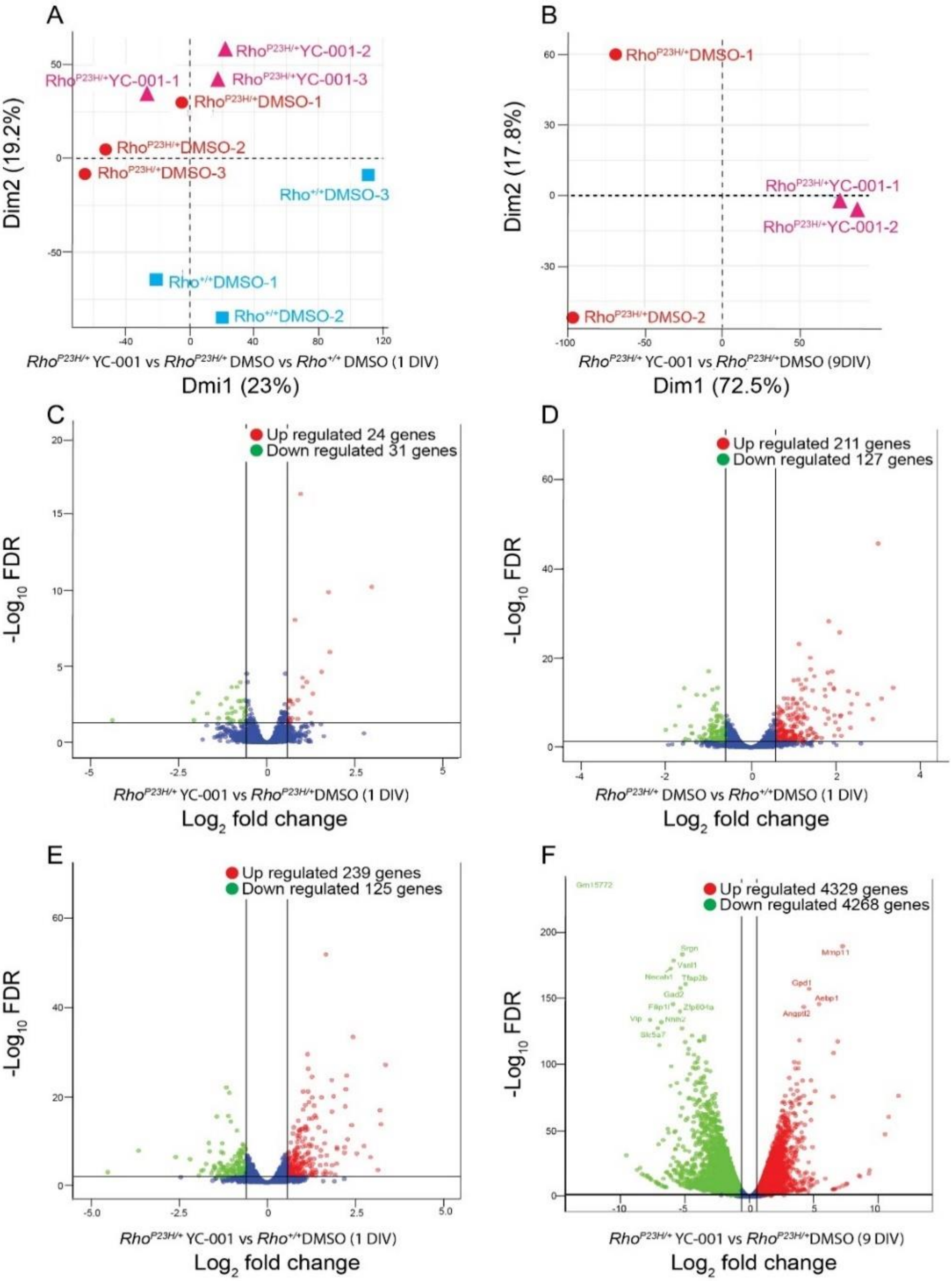
A
Dim2 (19.2%)
Rho^P23H/+ YC-001-2
Rho^P23H/+ YC-001-1
Rho^P23H/+ YC-001-3
Rho^P23H/+ DMSO-1
Rho^P23H/+ DMSO-2
Rho^P23H/+ DMSO-3
Rho+/+ DMSO-3
Rho+/+ DMSO-1
Rho+/+ DMSO-2
Rho^P23H/+ YC-001 vs Rho^P23H/+ DMSO vs Rho+/+ DMSO (1 DIV)
Dmi1 (23%)
B
Dim2 (17.8%)
Rho^P23H/+ DMSO-1
Rho^P23H/+ YC-001-1
Rho^P23H/+ YC-001-2
Rho^P23H/+ DMSO-2
Rho^P23H/+ YC-001 vs Rho^P23H/+ DMSO (9DIV)
Dim1 (72.5%)
C
Up regulated 24 genes
Down regulated 31 genes
-Log10 FDR
Rho^P23H/+ YC-001 vs Rho^P23H/+ DMSO (1 DIV)
Log2 fold change
D
Up regulated 211 genes
Down regulated 127 genes
-Log10 FDR
Rho^P23H/+ DMSO vs Rho+/+ DMSO (1 DIV)
Log2 fold change
E
Up regulated 239 genes
Down regulated 125 genes
-Log10 FDR
Rho^P23H/+ YC-001 vs Rho+/+ DMSO (1 DIV)
Log2 fold change
F
Gm15772
Up regulated 4329 genes
Down regulated 4268 genes
Srgn
Vsnl1
Necab1
Tfap2b
Gad2
Zfp804a
Vip
Slc5a7
Mmp11
Gpd1
Aebp1
Angptl2
-Log10 FDR
Rho^P23H/+ YC-001 vs Rho^P23H/+ DMSO (9 DIV)
Log2 fold change

Figure S11. PCA plot and volcano plot of RNA-seq data from retinal explants at 1 and 9 DIV. *Rho*$^{P23H/+}$ and *Rho*$^{+/+}$ mouse retinal explant were cultured and treated with YC-001 and DMSO vehicle control and total RNA were isolated at 1DIV and 9 DIV. **A** and **B.** Two-dimension principal component analysis (PCA) plots of RNAseq data at 1 and 9 DIV, respectively. Red circles, the *Rho*$^{P23H/+}$ retinal explants treated with DMSO; magenta triangles, *Rho*$^{P23H/+}$ retinal explants treated with 40 µM YC-001; and cyan squares, *Rho*$^{+/+}$ retinal explants treated with DMSO. N=3 for A. N=3 for B. **C-F.** volcano plots plotted for $\log_2$ fold change in x-axis and $\log_{10}$ false discovery rate (FDR) value in y-axis. **C.** *Rho*$^{P23H/+}$ YC-001 *vs. Rho*$^{P23H/+}$ DMSO at 1 DIV; **D.** *Rho*$^{P23H/+}$ DMSO *vs. Rho*$^{+/+}$ DMSO at 1 DIV; **E.** *Rho*$^{P23H/+}$ YC-001 *vs. Rho*$^{+/+}$ DMSO at 1 DIV; and **F.** *Rho*$^{P23H/+}$ YC-001 *vs. Rho*$^{P23H/+}$ DMSO at 9 DIV. Red circles are the upregulated differentially expressed genes (DEGs) and green circles are the downregulated DEGs. DEGs are identified with fold change ≥1.5 and FDR ≤0.05. N=3 for RNA-seq data of retinal explants at 1 DIV. N=2 for data from Retinal explants at 9 DIV due to exclusion of one outlier in each group.

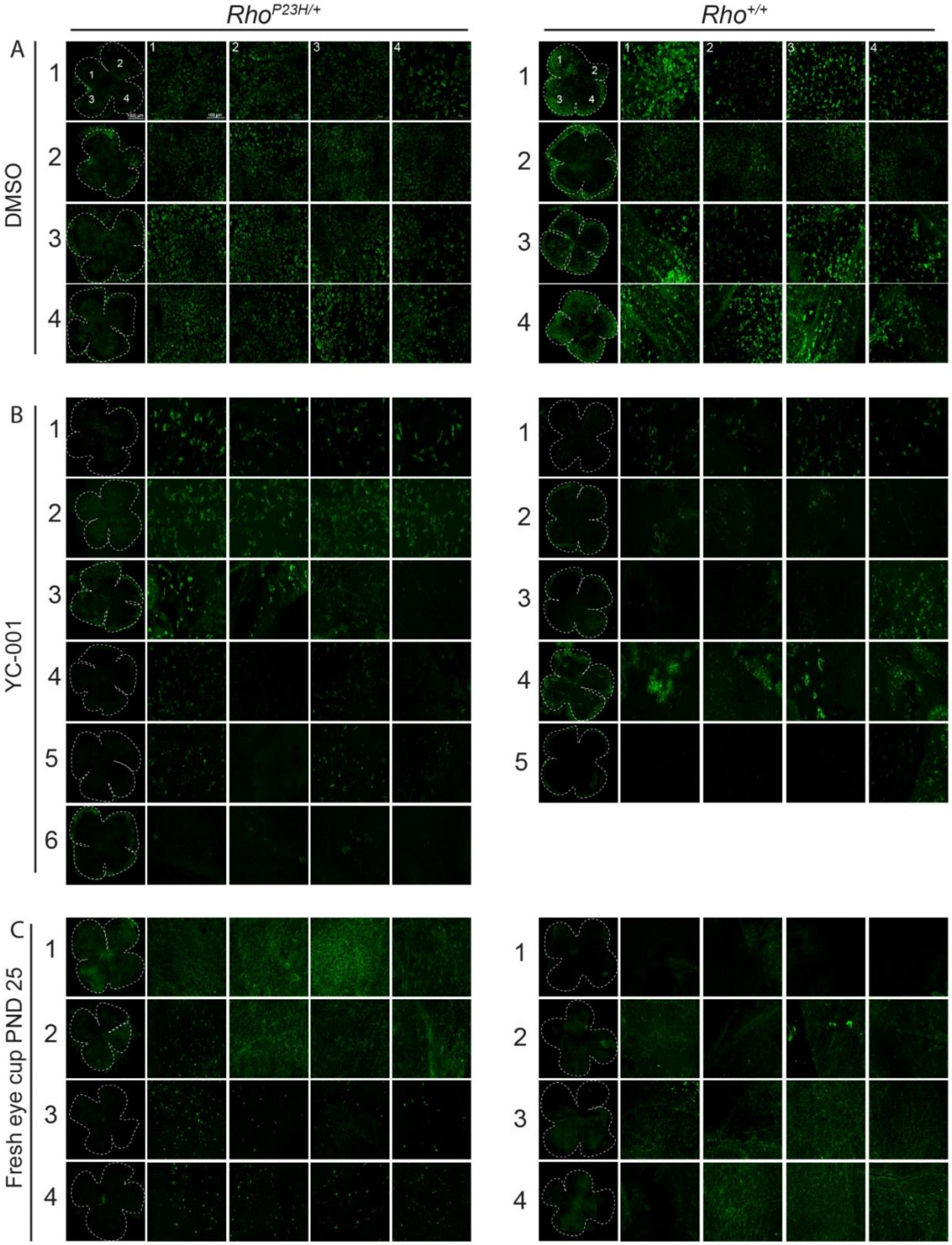

Rho^P23H/+
Rho^+/+
A
DMSO
B
YC-001
C
Fresh eye cup PND 25
1
2
3
4
5
6

Figure S12. Immunostaining images of all flat mounts of *Rho*$^{P23H/+}$ and *Rho*$^{+/+}$ retinal explants. A and B. Retinal explants were treated with DMSO or 40 µM YC-001 for 9 DIV, respectively. **C.** Fresh eye cups at PND 25 were used as *in vivo* age matched controls. Left panel represent the *Rho*$^{P23H/+}$ and right panel represents the *Rho*$^{+/+}$. The first image of each panel are stitched images at 20x, (Scale bars, 1000 µm) all other images are digitally zoomed (100x, Scale bars, 100 µm). The number of the left side are replicates, N=4-6. Representative images and quantifications are shown in **Figure 8G-M**.

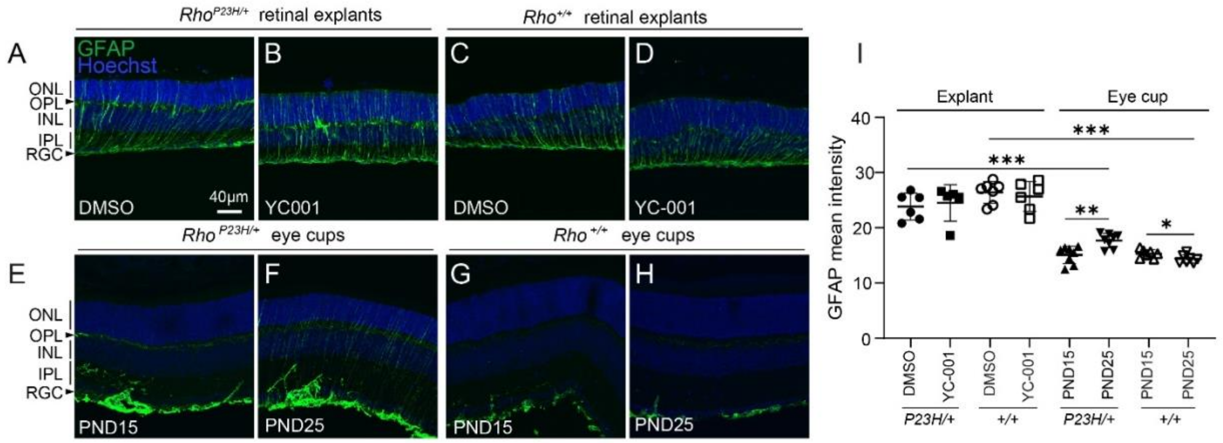

Figure S13. GFAP staining showed Müller glia activation in the *Rho*$^{P23H/+}$ and *Rho*$^{+/+}$ mouse retinal explants were not affected by YC-001. *Rho*$^{P23H/+}$ and *Rho*$^{+/+}$ mouse retinal explants were isolated at PND15 and cultured and then treated with 40 µM YC-001 or DMSO vehicle control for 9 DIV. Fresh media with YC-001 and DMSO were changed every day. Eye cups at PND15 and 25 were collected as *in vivo* controls. The cryosections were immunostained against Glial fibrillary acidic protein (GFAP) to track Müller glia activity. **A-H.** Immunofluorescence images of *Rho*$^{P23H/+}$ and *Rho*$^{+/+}$ retinal explants at 9 DIV **(A-D)** and eye cups at PND 15 and 25 **(E-H)**, stained against GFAP. **I.** Graphs represent the GFAP mean intensity in *Rho*$^{P23H/+}$ and *Rho*$^{+/+}$ retinal explants and eye cups. N=5-8. Significance of the analysis between two groups were calculated by the Mann Whitney test. Middle and error bars are the means±SDs and *, **, ***, P<0.05, <0.01 and <0.001, respectively, by the Kruskal-Wallis test.

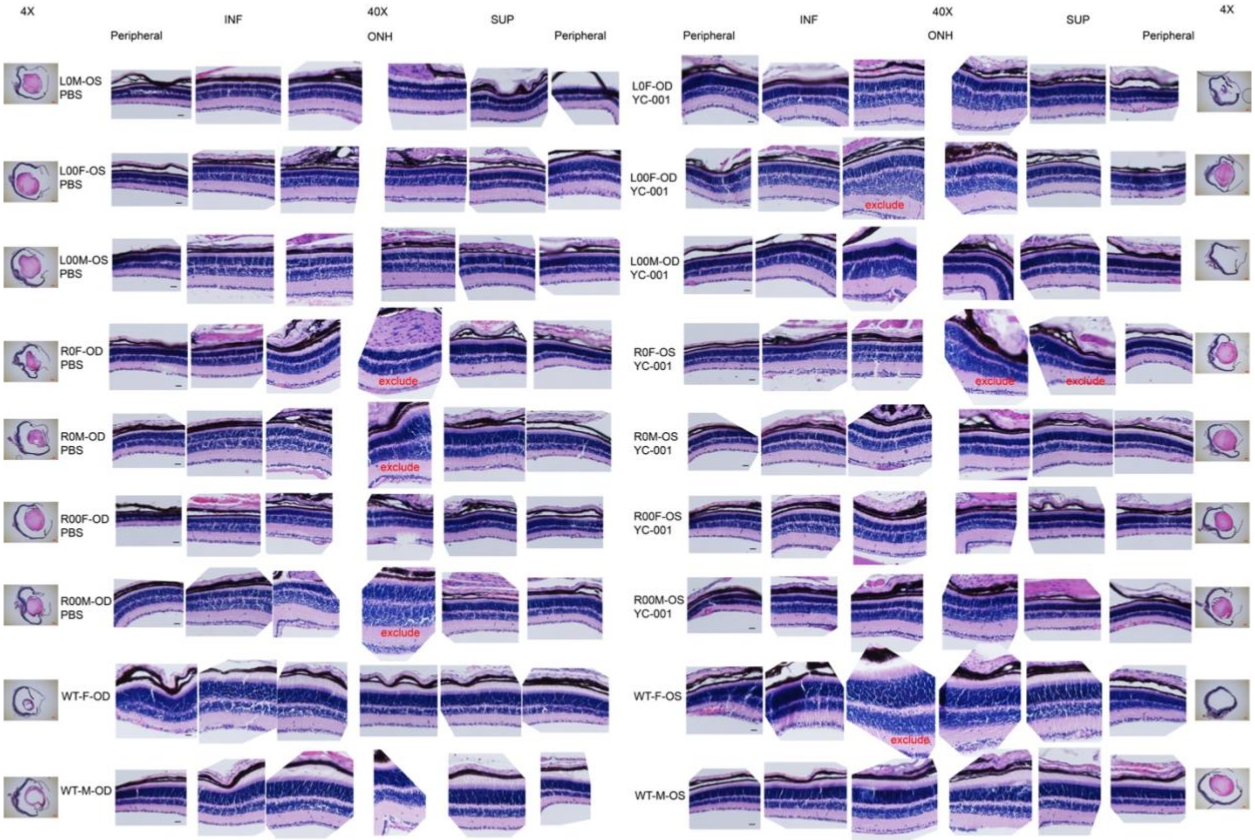

Figure S14. H&E staining images at low (4x) and high (40x) magnifications. PBS or YC-001 treated eyes were from *Rho*$^{P23H/+}$ knock-in mice, and WT are shown at the bottom two rows as normal control. Images marked with “exclude” were excluded from measurements because of their abnormal morphology caused by sectioning. ONL thickness measured from these images were summarized in **Figure 12J**.

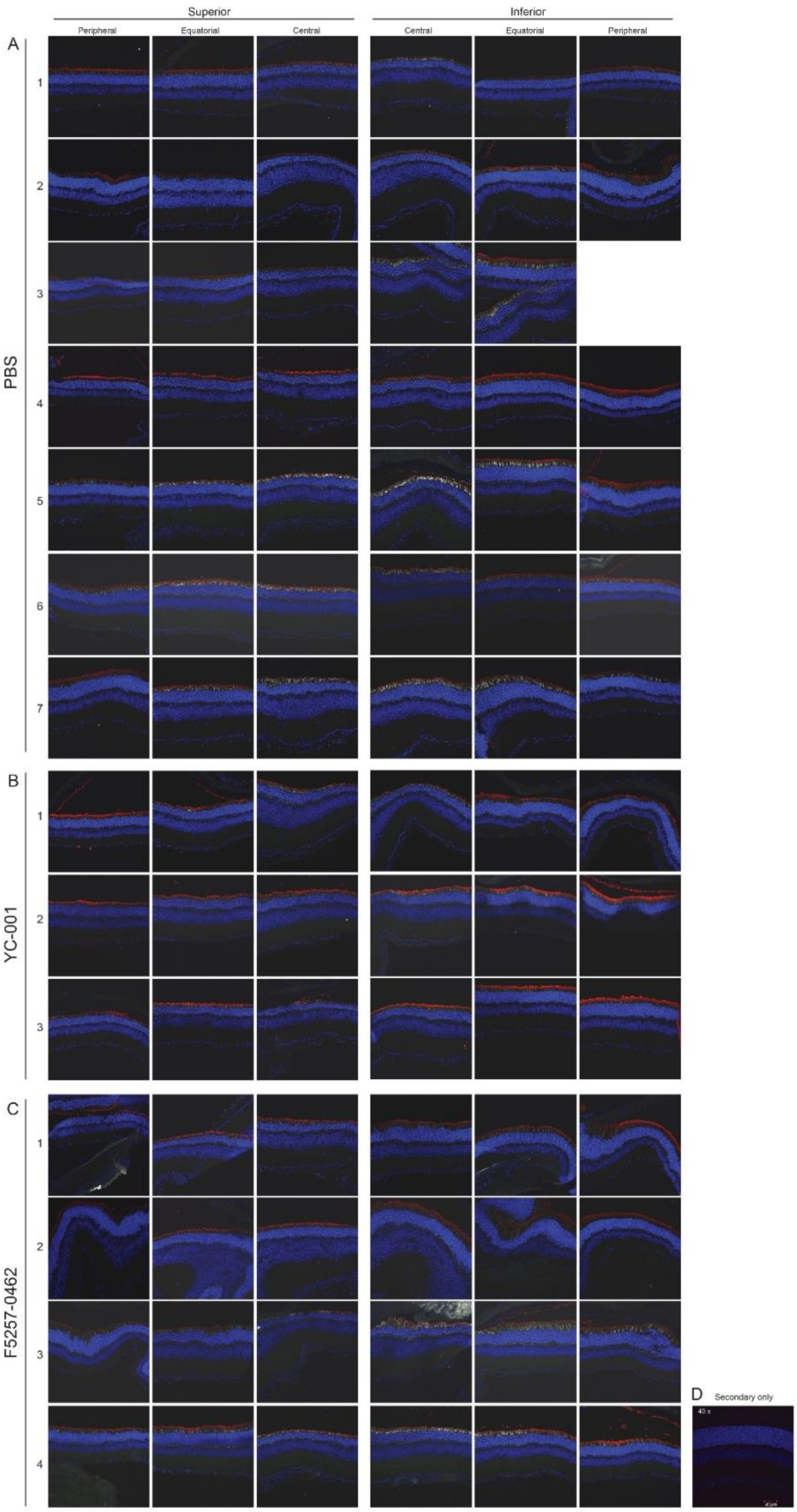
Superior
Inferior
Peripheral
Equatorial
Central
Central
Equatorial
Peripheral
A
PBS
1
2
3
4
5
6
7
B
YC-001
1
2
3
C
F5257-0462
1
2
3
4
D
Secondary only
40 x

(3)

Figure S15. All the immunostaining images of *Rho*$^{P23H/+}$ at PND 60 treated with intravitreal injection of PBS, YC-001, or F5257-0462 used in figure 11. Immuno-stained eye cups from *Rho*$^{P23H/+}$ retinal explants treated with IVI of PBS **(A)**, YC-001 **(B)** and F5257-0462 **(C)**. The left panel of A, B, and C are superior part of the retina (Left to right; center, equatorial, and peripheral). The right panel of A, B, and C are inferior part of the retina (Right to left; center, equatorial, and peripheral). **D** is the secondary only stained WT retina. The number on the right side represent the corresponding replicates.

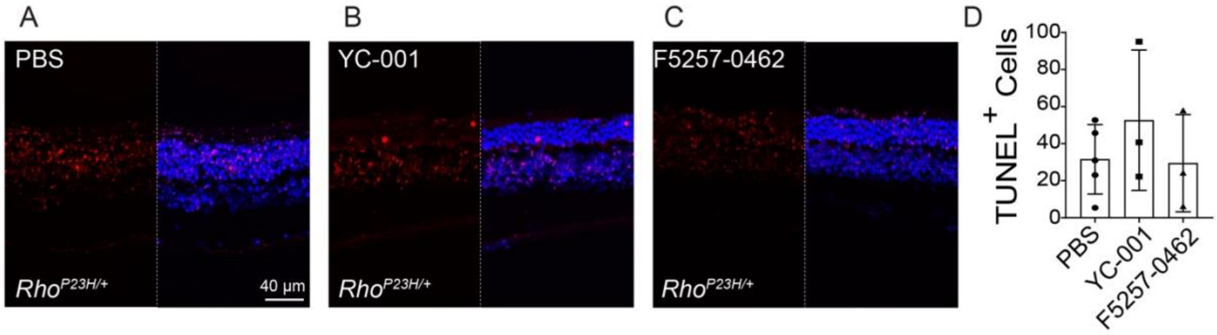

Figure S16. TUNEL staining of retinae from mice treated with two IVIs of YC-001 or F5257-0462 showed no toxicity from either compound. *Rho*$^{P23H/+}$ mice were treated with two IVIs of YC-001 or F5257-0462 as for 35 nmol per eye as the slurry of microparticles in PBS at PND15 and 30, with the contralateral eyes treated with PBS as control. Eyes were isolated at PND 60 and TUNEL staining was performed to retinal cryosections. **A-C.** TUNEL staining images of retinae from PBS-, YC-001-, and F5257-0462-treated eyes. Left, TUNEL staining in red channel only; right, composite images of TUNEL in red and hoechst33343 staining in blue for nucleus staining. Scale, 40 µm. **D.** Bar plot of TUNEL^{+} cells per image averaged from 3 positions per retina. Each data point represents an eye. Columns and error bars are means±SDs. No significant differences were seen between groups by Mann Whitney U Test.

Supplementary Methods

Medium and chemicals. Reagents, media, antibodies, chemicals, and materials are listed in **Table S3**. YC-001 was synthesized as described previously by a two-step reaction (4): 1) The 2-bromo-1-(5-chlorothiophen-2-yl)ethan and 2-(thiophen-2-yl)acetic acid were mixed with trimethylamine (Et3N) and acetonitrile (CH3CN) at room temperature for 20 min to generate the 2-(5-chlorothiophen-2-yl)-2-oxoethyl 2-(thiophen-2-yl)acetate via α-substitution; 2) the product in step 1 was mixed with 1,8-diazabbicycolo[5.4.0]undec-7-ene(DBU) for 20 min at room temperature to produce YC-001 after purification over silica.

Table S3. List of reagents.

No.	Product Name	Catalogue#	Company	City and Country
1	Ame's solution	A1420	Sigma-Aldrich	St louis, MO, USA
2	L-cysteine	C7602	Sigma-Aldrich	St louis, MO, USA
3	Papain	LS003126	Wortington Corp.	Lakewood, USA
4	Neurobasal plus medium	A35829-01	Life Technologies	Carlsbad, CA, USA
5	B27 plus supplement 50x	A35828-01	Life Technologies	Carlsbad, CA, USA
6	Penicillin-streptomycin 100x	PSL01	Genesee Scientific Corporation	San Diego, USA
7	Plasmocin prophylactic	ANT-MPP	InvivoGen	San Diego CA, USA
8	Dulbecco's modified Eagle's medium (DMEM)	25-500	Genesee Scientific Corporation	San Diego, USA
9	Fetal bovine serum (FBS)	10438026	Gibco	Grand island, NY, USA
10	Phosphate buffered saline (PBS)	25-507xB	Genesee Scientific Corporation	San Diego, USA
11	Prolong gold mounting solution	P36930	Thermo Fisher Scientific	Waltham, MA, USA
12	Hoechst33342	H3570	Thermo Fisher Scientific	Waltham, MA, USA
13	Chemiluminescent substrates	34580	Thermo Fisher Scientific	Waltham, MA, USA
14	RNAzol	R4533	Sigma-Aldrich	St louis, MO, USA
15	High-Capacity RNA-to-cDNA™ Kit	4388950	Applied Biosystems	Vilnius, Lithuania
16	PowerUp™ SYBR™ Green Master Mix	A25742	Applied Biosystems	Vilnius, Lithuania
17	DMSO	D2650	Sigma-Aldrich	St louis, MO, USA
18	O.C.T. Compound Cryostat Embedding Medium	23-730-625	Scigen Scientific	Gardena, CA, USA
19	Click-iT™ Plus TUNEL Assay for In Situ Apoptosis	C10618	Invitrogen	Eugene, Oregon, USA

	Detection, Alexa Fluor™ 594 dye			
20	10% Goat serum	50062Z	Life Technologies	Carlsbad, CA, USA
21	16% Paraformaldehyde (PFA)	28908	Thermo Fisher Scientific	Waltham, MA, USA
22	Phire Tissue Direct PCR Master mix	F170S	Thermo Fisher Scientific	Waltham, MA, USA
23	Anti-microtubule-associated proteins 1A/1B light chain 3B (LC3A/B) (Rabbit)	4108	Cell Signaling Technology	Danvers, MA, USA
24	Anti-gapdh antibody (mouse)	Ma5-15738	Thermo Fisher Scientific	Waltham, MA, USA
25	Anti-β actin antibody (mouse)	MA5-15739	Thermo Fisher Scientific	Waltham, MA, USA
26	Anti sequestosome-1 (SQSTM1/p62) antibody (Rabbit)	5114	Cell Signaling technology	Danvers, MA, USA.
27	Anti-poly-ubiquitin antibody (Rabbit)	U5779	Sigma Aldrich	Waltham, MA, USA
28	Anti-CD68 antibody (Rabbit)	ab125212	Abcam	Cambridge, MA, USA
29	1D4 RHO antibody (Mouse)	Shared by Dr. Krzysztof Palczewski, University of California at Irvine		Irvine, CA, USA
30	HRP-1D4 (HRP-1D4 RHO) was generated using a HRP conjugation kit (Mouse)	ab102890	Abcam	Cambridge, MA, USA
31	Anti-GFAP antibody (Rabbit)	Z0334	Agilent Technologies	Santa Clara, CA
32	HRP conjugated goat polyclonal anti-mouse antibody	32230	Thermo Fisher Scientific	Waltham, MA, USA
33	HRP conjugated goat polyclonal anti-rabbit antibody	32260	Thermo Fisher Scientific	Waltham, MA, USA
34	Cy3-conjugated goat anti-mouse IgG	A10521	Thermo Fisher Scientific	Waltham, MA, USA
35	Alexa Fluor™ 488-conjugate peanut agglutinin (PNA)	L21409	Thermo Fisher Scientific	Waltham, MA, USA
36	Goat anti-rabbit IgG secondary antibody, Alexa Fluor Plus 488	A32731	Thermo Fisher Scientific	Waltham, MA, USA
37	F5257-0462	F5257-0462	Life Chemicals Inc.	Niagara-on-the-lake, ON, Canada
38	Dimethyl sulfoxide (DMSO)	276855	Sigma-Aldrich	St louis, MO, USA
39	Neural tissue dissociation kit for postnatal neurons	130-094-802	Miltenyi biotec	Germany
40	Zombie UV viability dye	423107	BioLegend	
41	BD cytofix/cytoperm			
42	Fc Block	422301	BioLegend	
43	Stain buffer			
44	Quick Change II XL Site-directed Mutagenesis Kit	200522	Agilent	Santa Clara, CA, USA
45	Lipofectamine3000	L3000008	Invitrogen	
46	Tropicamide		Akorn	Lake forest, IL, USA

47	Phenylpherine		Sigma	St louis, MO, USA
48	Tetracaine		TCI	Tokyo, Japan
49	GenTeal tears lubricant eye gel		Alcon	Fort Worth, TX, USA
50	Tri-antibiotic ointment		Medline	Northfield, IL, USA
51	PEG35 Castor oil	1547200	Sigma	St louis, MO, USA
52	CD11b PE/Dazzle 594	Clone: M1/70	Biolegend	
53	F4/80 APC	Clone: BM8	Biolegend	
54	MHCII, I-A/I-E BV510	Clone: M5/114.15.2	Biolegend	
55	iNOS eF450	Clone: CXNFT	Biolegend	
56	CD68 BV785	Clone: FA-11	Biolegend	

Cell line. NIH3T3 cells were originally obtained from American Type Culture Collection, Manassas, VA, USA.

Mutagenesis, transfection, treatment, cell surface immunostaining of WT and RHO mutants and high content imaging. The human *RHO* cDNA in the pCDNA3.1 (+) vector was shared by Dr. Krzysztof Palczewski (University of California at Irvine, Irvine, CA, USA) and mutagenesis was performed using the Quick Change II XL Site-directed Mutagenesis Kit (200522, Agilent) following manufacture's manual. A total of 27 mutants were generated including: T4K, N15S, T17M, P23H, P23L, Q28H, P53R, G106R, G106W, G109R, C110Y, Y178N, Y178C, P180A, E181K, G182S, Q184P, S186P, S186W, C187Y, G188R, G188E, D190N, D190Y, T193M, P267L, and G284S. Cell culture, transfection and high-content imaging were performed as described (4, 5). Briefly, NIH3T3 cells were seeded at 5000 cells/well and cultured in a black-wall, clear bottom, and poly-L-lysine-treated 384-well plate at 37 °C with 5% CO_2 on day 1, and then transfected with the plasmids containing *hRHO* cDNA mutants using Lipofectamine3000 (L3000008, Invitrogen) on day 2. On day 3, cells were treated with 0.1% DMSO, 5 µM 9-*cis*-retinal (under dim red light), 40 µM YC-001, or 20 µM F5257-0462 in DMEM with 10% FBS for 24 h. The concentration of each compound was determined using the most efficacious and safe dose obtained from dose-response curves from our previous study *in vitro* (4). On day 4, treated cells were fixed with 4% paraformaldehyde (PFA) in the dark for 20 min and immunostained with 15

µL/well of 20 µg/mL Alexa488-conjugated B6-30 anti-RHO antibody and Hoechst 33342 (1:10,000 dilution). Fluorescence images were taken using an ImageXpress high-content imager (Molecular Devices, San Jose, CA, USA) and analyzed using MetaXpress (Molecular Devices). Transfection efficiency of WT and cherry-picked mutant RHO were confirmed by qPCR (**Figure S2**). Average total fluorescence intensity from surface staining of RHO per cell was calculated from five fields of images per well containing 600-1000 cells. N=3.

Docking calculations. We used AutoDock Vina (6) to calculate the most stable conformations of YC-001, F5257-0462, 9-*cis*-retinal, and 11-*cis*-retinal in the chromophore pocket of the crystal structure of bovine RHO (PDB ID: 1F88) (7) by deleting the experimentally observed 11-*cis*-retinal. A 50 x 50 x 50 $Å^3$ cubic box was centered by the chromophore pocket as a center, which was set as the docking space. Up to seven rotary bonds were set for each ligand, and the most stable conformation for each molecule was selected as the result with the lowest ΔG.

Tissue collection and histology. For paraffinized sections, eyes were enucleated and fixed in 4% PFA and 1% glutaraldehyde for 24 h. Fixed eyes were dehydrated, paraffinized and sectioned at 10 µm thickness followed by H&E stain. For cryosections, retinal explants and eye cups were fixed in 4% PFA for 2 h followed by sequential sucrose gradient dehydration (5, 10, 20 and 40% sucrose for 30 min each). Eyes were incubated overnight in the mixture of 40% sucrose in PBS and O.C.T. compound (1:1 v/v) before being embedded and flash frozen. Cross-sections (14 µm) containing optic nerve head were cut and mounted onto Superfrost glass slides (12-550-15, Fisher Scientific) for IHC and TUNEL assay. For retinal flat mounts, eye cups were prepared and flattened by four cuts. Sclera was removed leaving the neural retina fixed in 4% PFA for 2 h before immunostaining.

Immunohistochemistry (IHC) and TUNEL staining. IHC was performed as described previously (8) on cryosections or retinal flat mounts. Primary antibodies include mouse 1D4 anti-

RHO antibody (20 µg/mL), and rabbit anti-CD68 antibody (0.5 µg/ml). Secondary antibodies were applied: Cy3-conjugated goat anti-mouse antibody (5 µg/mL) and Alexa488-conjugated goat anti-rabbit secondary antibody (2 µg/mL). *In Situ* apoptosis detection was performed on the cryosections of mouse retinae or retinal explants using Click it plus TUNEL assay, Alexa fluor 594 following the manufacture's manual. Alexa-488 conjugated PNA (5 µg/mL) and Hoechst 33342 (2 µg/mL) were used to stain cones and nucleus, respectively. Immunofluorescence images were taken using an Olympus FV1200 confocal microscope and image analyses including thickness measurements and cell counting were performed using Fiji ImageJ (imagj.nih.gov/ij) (9). $TUNEL^+$ cells were quantified with Metamorph v7.8 software.

Immunoblots. Immunoblots were performed as described previously (4, 8). For detection against RHO, thirty µg of total protein per retina was resolved on 12% SDS-PAGE gel and immunoblotted on nitrocellulose membrane with 0.2 µg/mL HRP-conjugated 1D4 anti-RHO antibody. For detecting polyubiquitinated proteins, sixty µg of heated total protein per sample was loaded on an 8% SDS-PAGE followed by immunoblotting with the rabbit anti-ubiquitin antibody (1:100 dilution). For detection of LC3 and SQSTM/p62, thirty µg of heated total protein per sample was loaded on a 15% SDS-PAGE. The transferred membrane (PVDF) was incubated with antibodies against LC3 (rabbit, 1:1000 dilution) and SQSTM/p62 (rabbit, 1 µg/ml). Immunoblots against Glycereldehyde 3-phosphate dehydrogenase (GAPDH, mouse, 1 µg/ml) and β-Actin (mouse, 1 µg/ml) were used as loading controls. Corresponding secondary anti-mouse-HRP (0.1 µg/ml, 1:5000) and anti-rabbit-HRP (0.1 µg/ml, 1:5000) were used before development with the Supersignal west pico chemiluminescent substrate. Fiji ImageJ was used for the quantification of band intensities.

RHO immunoprecipitation. RHO was immunoprecipitated from total protein isolated from retinal explants using Dynabeads Protein G following manufactures instructions. Briefly, lysates (60 µg total protein/sample) were precleaned with Dynabeads and immunoprecipitated with 10 µg of 1D4

anti-RHO antibody-bound Dynabeads Protein G complex. The resulting elutes were immediately mixed with equal volume of 1M Tris/HCl, pH 7.5 for immunoblotting against poly-ubiquitin followed by RHO. The ratio of immunoblot band intensities of ubiquitinated RHO to total RHO reflected the relative amount of misfolded RHO to total RHO. Mouse serum was used as isotype control.

Glycosylation assay. Retinal explants were lysed, and 15 µg of total protein was diluted in 13 µL of lysis buffer containing PBS with 0.1% SDS and 1% DDM. Each fraction of normalized retinal lysate was mixed with 2 µL of glycoprotein denaturing buffer (10x), two µL of Glycobuffer 3 (10x) and either 3 µL water for undigested, three µL EndoH (1500 unit), or 3 µL PNGaseF (1500 unit). The resulting reaction mixture was then incubated at 37 °C for 1 h and resolved on a gradient SDS-PAGE (lower half 12%, upper half 10%) and immunoblotted with HRP-conjugated 1D4 anti-RHO antibody.

RNA-seq and transcriptome analysis. Total RNA was isolated from cultured retinal explants by RNAzol following manufacturer's instructions. The concentration and quality of purified total RNA was examined by a Nanodrop spectrophotometer and a bioanalyzer. RNA samples with RNA Integrity Number (RIN) > 7 were used for poly-A capture mRNA library preparation. The library size was analyzed using bioanalyzer and RNA-seq was performed using Illumina HiSeq platform by QuickBiology Inc. The reads were first mapped to the hg38 UCSC transcript set using Bowtie2 version 2.1.0 and the gene expression level was estimated using RSEM v1.2.15 (10). Differentially expressed genes (DEGs) were identified using the edgeR program (11). Genes showing altered expression with FDR < 0.05 and more than 1.5-fold changes were considered DEGs. Goseq (12) was used to perform the GO enrichment analysis and Kobas (13) was used to perform the pathway analysis.

qPCR. Total RNA was isolated as described above and converted to cDNA using High-Capacity RNA-to-cDNA™ Kit following manufacturer protocol. qPCR was carried out by QuantStudio™ 3

real-time PCR (RT-PCR) cycler using PowerUp™ SYBR™ Green Master Mix (A25742, ABI) with primers listed in **Table S4**. β-actin was used as an endogenous control. Fold change was calculated by dividing the $2^{-\Delta Ct}$ of YC-001 treated by averaged $2^{-\Delta Ct}$ of DMSO controls

Table S4. Primers used for qPCR confirming the RNA-seq data.

Number	Gene ID	Forward (5'-3')	Reverse (5'-3')	Amplicon size (bp)
1	A2m	CCAAAGCGTACTGCTCACAA	ATTGACTCCAAGGCGTTCAC	289
2	Fbln2	CCAAAGCGTACTGCTCACAA	ATTGACTCCAAGGCGTTCAC	255
3	Igtp	CCAGAGCGTTCACTCAGACA	ATTTAGACCACGGGCTGATG	172
4	Lrat	TATGGCTCTCGGATCAGTCC	CAGATTGCAGGAAGGGTCAT	155
5	Ndp	GCTGGCCATAATGGGAGATA	GAACGGAAAGGTTGCTTGAG	222
6	Shisa3	GCAACTATCACGAGGGGTTC	TGATGAAGGCGATGAAGATG	225
7	Steap4	CAAATGCGGAATACCTTGCT	GGTGAGCCCAAGAGTACGAG	182
8	Mmp11	GGTTCTTCCAAGGTGCTCAG	GGAAACGCCAATAGTCTCCA	171
9	Neurog2	GATGCCAAGCTCACGAAGAT	ACGTGGAGTTGGAGGATGAC	238
10	Ulk1	CCCAGAGTACCCGTACCAGA	TAGGGTTTCCGTGTGCTCTT	244
11	Atg13	GAAGGCTTCCAGACAGTTCG	GGGACGGTCAACAAAGTGAT	162
12	Atg12	AACAAAGAAATGGGCTGTGG	TGCCTGGGATTTGCAGTAAT	208
13	Atg16L2	GACAGGTGTTCAGGGCAGAT	CACACTCACAACGTGGTTCC	218
14	Sqstm1	GCTGCCCTATACCCACATCT	CGCCTTCATCCGAGAAAC	94
15	Atg6	TAAAAGGCAGCAGCTGGAGT	CAAGCGACCCAGTCTGAAAT	172
16	Lc3	CGTCCTGGACAAGACCAAGT	CAGGAAGCCGTCTTCATCTC	193
17	Hsf1	AGGCAGGAGCATAGATGAGA	AGGATGGAGTCAATGAAGGC	165
18	Hsp70	GAGGAGTTCAAGAGGAAG	TGATGGATGTGTAGAAGTC	166
19	Ire1	ACACCGACCACCGTATCTCA	CTCAGGATAATGGTAGCCATGTC	110
20	Atf6	TGCCTTGGGAGTCAGACCTAT	GCTGAGTTGAAGAACACGAGTC	141
21	Grp78	CCTGCGTCGGTGTGTTCAAG	AAGGGTCATTCCAAGTGCG	201
22	Chop	TATCTCATCCCCAGGAAACG	GGGCACTGACCACTCTGTTT	219
23	Perk	CGGATTCATTGAAAGCACCT	ACGCGATGGGAGTACAAAAC	194
24	Sxbp1	GAGTCCGCAGCAGGTG	GTGTCAGAGTCCATGGGA	65
25	Usxbp1	AAGAACACGCTTGGGAATGG	ACTCCCCTTGGCCTCCAC	67
26	Actb	CTACAGCTTCACCACCACAG	CTCGTTGCCAATAGTGATGAC	169

Measurement of YC-001 clearance in eyes and YC-001 stability in culture medium. One nmol per eye of YC-001 was dissolved in PEG35 castor oil and administered to C57BL/6J mice at 4-8 wks of age by IVI; eyes were isolated at 0, 1, 3, 6, 10 and 24 h after treatment, and YC-001

was extracted and quantified by HPLC modified from previously described method (4). Briefly, each isolated eyeball was flash frozen and homogenized in 1 mL PBS: methanol mixture (1:1 v/v) on ice, 1 mL chloroform (FisherScientific) was added to the mixture followed by 2 min of vigorous shaking. The mixture was centrifuged at 3,578 x g at 4 °C for 10 min. The bottom chloroform phase was collected, dried, and dissolved in 120 µL methanol and filtered through a 0.22 µm syringe filter before loading on a HPLC system connected to an Agilent C18 SiI column (5 µm, 4.6 x250 mm; Agilent Technologies). Samples from two eyes were pooled together and a total of six eyes were used for each time point (N=3). YC-001 was resolved using 1 mL/min flow rate with 30-100% acetonitrile: H_2O gradient from 0-15 min followed by 5 min with 100% acetonitrile, which can be detected at 10.7 min with absorption at 334 nm. Using purified YC-001 (0.01, 0.03, 0.1, 1, 3, 10, 30, and 100 µM) as standards, YC-001 level in the eyes was calculated. Culture medium with YC-001 was taken at 0, 1 and 24 h of culture time and YC-001 was extracted and quantified using the same method.

Electroretinogram (ERG). ERG was performed using the Celeris system (Diagnosys, Lowell, MA, USA) as described previously (8). Mice were dark-adapted overnight, and procedures were performed under dim red light. Pupils were dilated and eyes were lubricated before mice were anesthetized and put on a heated platform at 37 °C. Scotopic ERG responses to seven flash intensities (0.0001 to 100 cd·s/m^2) were recorded and averaged from 3-5 sweeps with 10 to 30 s intervals for each intensity. Animals were light-adapted under 10 cd/m^2 white light for 10 min, and photopic ERG responses (0.01 to 100 cd·s/m^2) were performed under 10 cd/m^2 background light.

Fundus imaging and SD-OCT. Fundus imaging and SD-OCT scanning were performed together. Pupils were dilated, eyes were lubricated, mice were anesthetized and put on a heating pad. Fundus images were taken by a MicronIV fundus camera (Phoenix Technology Group, Pleasanton, CA, USA) using the white light channel. Horizontal SD-OCT (Bioptigen, Morrisville,

NC, USA) scanning was taken with A/B scan ratio at 1200 lines and averaged from five frames. Thickness of layers was measured using Fiji ImageJ.

1. Chiang WC, Kroeger H, Sakami S, Messah C, Yasumura D, Matthes MT, et al. Robust Endoplasmic Reticulum-Associated Degradation of Rhodopsin Precedes Retinal Degeneration. *Mol Neurobiol.* 2015;52(1):679-95.
2. Sakami S, Maeda T, Bereta G, Okano K, Golczak M, Sumaroka A, et al. Probing mechanisms of photoreceptor degeneration in a new mouse model of the common form of autosomal dominant retinitis pigmentosa due to P23H opsin mutations. *J Biol Chem.* 2011;286(12):10551-67.
3. Lamothe M, Chang FJ, Balashova N, Shirokov R, and Beuve A. Functional characterization of nitric oxide and YC-1 activation of soluble guanylyl cyclase: structural implication for the YC-1 binding site? *Biochemistry.* 2004;43(11):3039-48.
4. Chen Y, Chen Y, Jastrzebska B, Golczak M, Gulati S, Tang H, et al. A novel small molecule chaperone of rod opsin and its potential therapy for retinal degeneration. *Nat Commun.* 2018;9(1):1976.
5. Feng B, Liu X, and Chen Y. A Rhodopsin Transport Assay by High-Content Imaging Analysis. *J Vis Exp.* 2019(143).
6. Trott O, and Olson AJ. AutoDock Vina: improving the speed and accuracy of docking with a new scoring function, efficient optimization, and multithreading. *J Comput Chem.* 2010;31(2):455-61.
7. Palczewski K, Kumasaka T, Hori T, Behnke CA, Motoshima H, Fox BA, et al. Crystal structure of rhodopsin: A G protein-coupled receptor. *Science.* 2000;289(5480):739-45.
8. Liu X, Feng B, Vats A, Tang H, Seibel W, Swaroop M, et al. Pharmacological clearance of misfolded rhodopsin for the treatment of RHO-associated retinitis pigmentosa. *FASEB J.* 2020;34(8):10146-67.
9. Schindelin J, Arganda-Carreras I, Frise E, Kaynig V, Longair M, Pietzsch T, et al. Fiji: an open-source platform for biological-image analysis. *Nat Methods.* 2012;9(7):676-82.
10. Li B, and Dewey CN. RSEM: accurate transcript quantification from RNA-Seq data with or without a reference genome. *BMC Bioinformatics.* 2011;12:323.
11. Robinson MD, McCarthy DJ, and Smyth GK. edgeR: a Bioconductor package for differential expression analysis of digital gene expression data. *Bioinformatics.* 2010;26(1):139-40.
12. Young MD, Wakefield MJ, Smyth GK, and Oshlack A. Gene ontology analysis for RNA-seq: accounting for selection bias. *Genome Biol.* 2010;11(2):R14.
13. Xie C, Mao X, Huang J, Ding Y, Wu J, Dong S, et al. KOBAS 2.0: a web server for annotation and identification of enriched pathways and diseases. *Nucleic Acids Res.* 2011;39(Web Server issue):W316-22.

Supplementary Data 1

Experimental Conditions

Cell Lines (Opsin Variants)			Transfection	Cell Count	Compounds Treated	Staining	Incubation Time
WT	G109R	C187Y	Lipofectamine 2000 into NIH3T3 cells seeded in 384 well plate	4000 cells / well	DMSO (0.1%)	RHO Cell Surface Stain (green):<br>20 µg / mL B6-30 + Alexa 488	24 hours
T4K	C110Y	G188R			9-*cis*-retinal (5 µM)	Nucleus Stain (blue):<br>Hoechst33343	
N15S	Y178N	G188E			YC-001 (40 µM)		
T17M	Y178C	D190N			F5257-0462 (20 µM)		
P23H	P180A	D190Y					
P23L	E181K	T193M					
Q28H	G182S	P267L					
P53R	Q184P	G284S					
G106R	S186P						
G106W	S186W						

Control:
No transfection

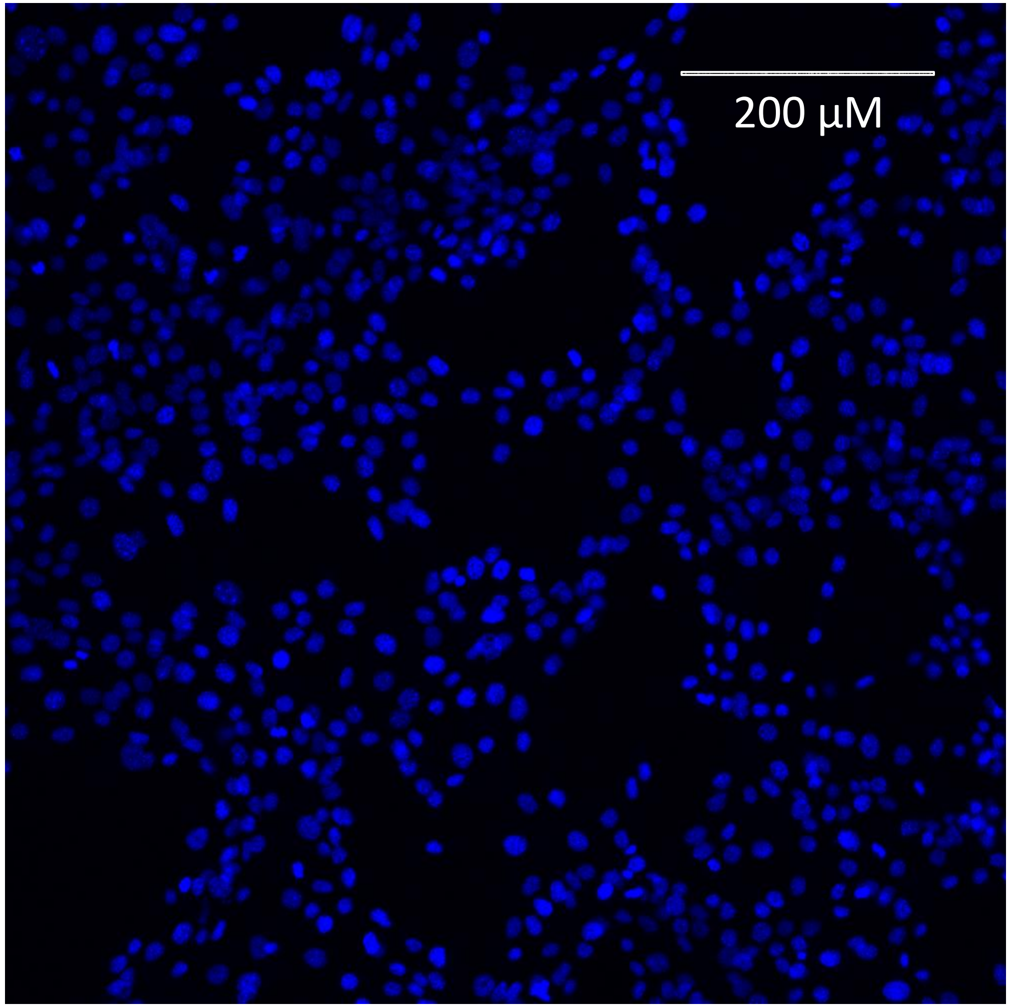

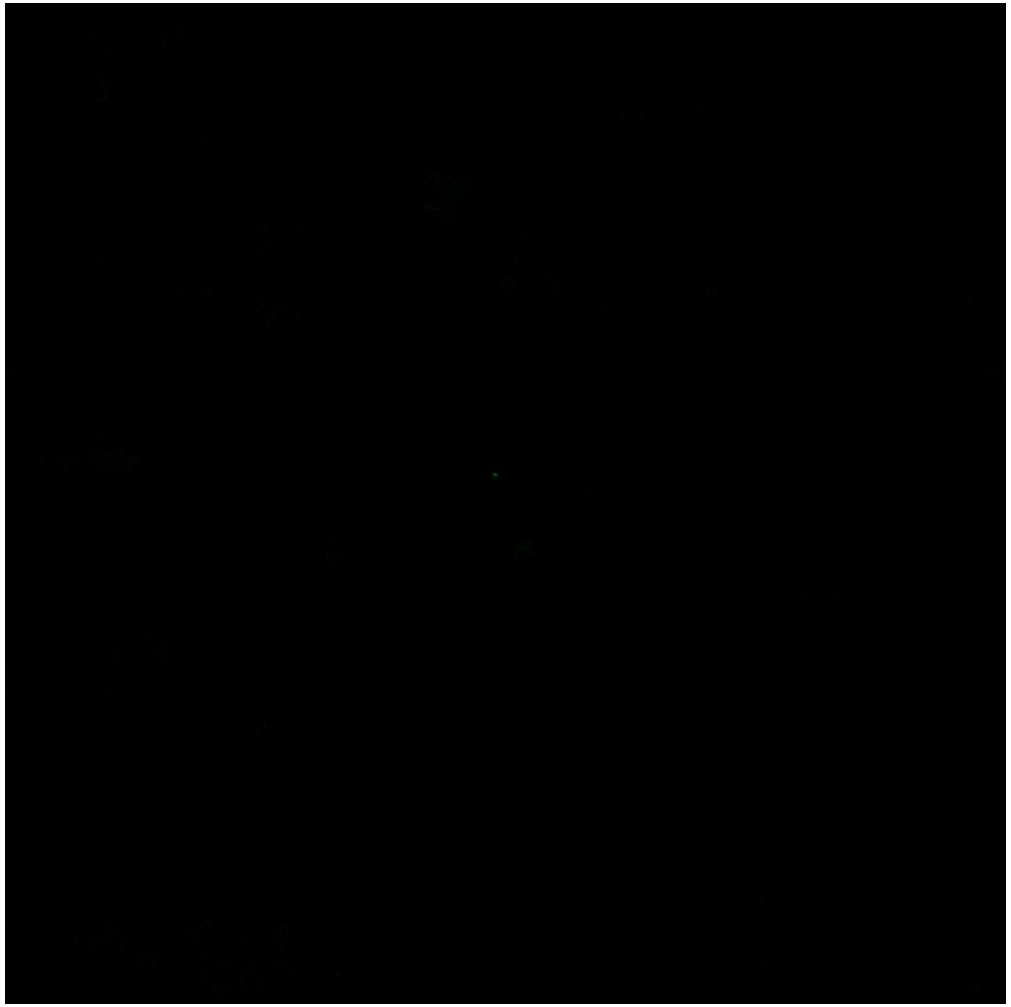

Control:
No transfection

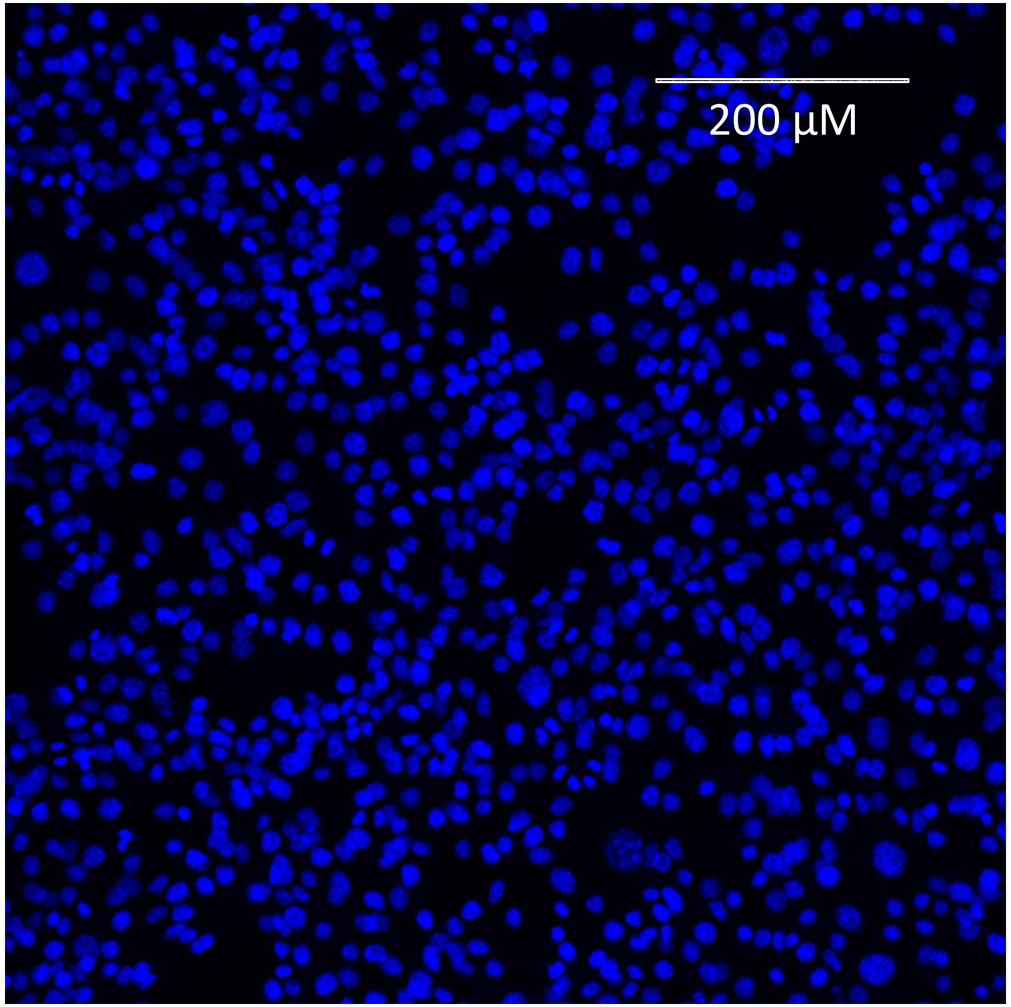

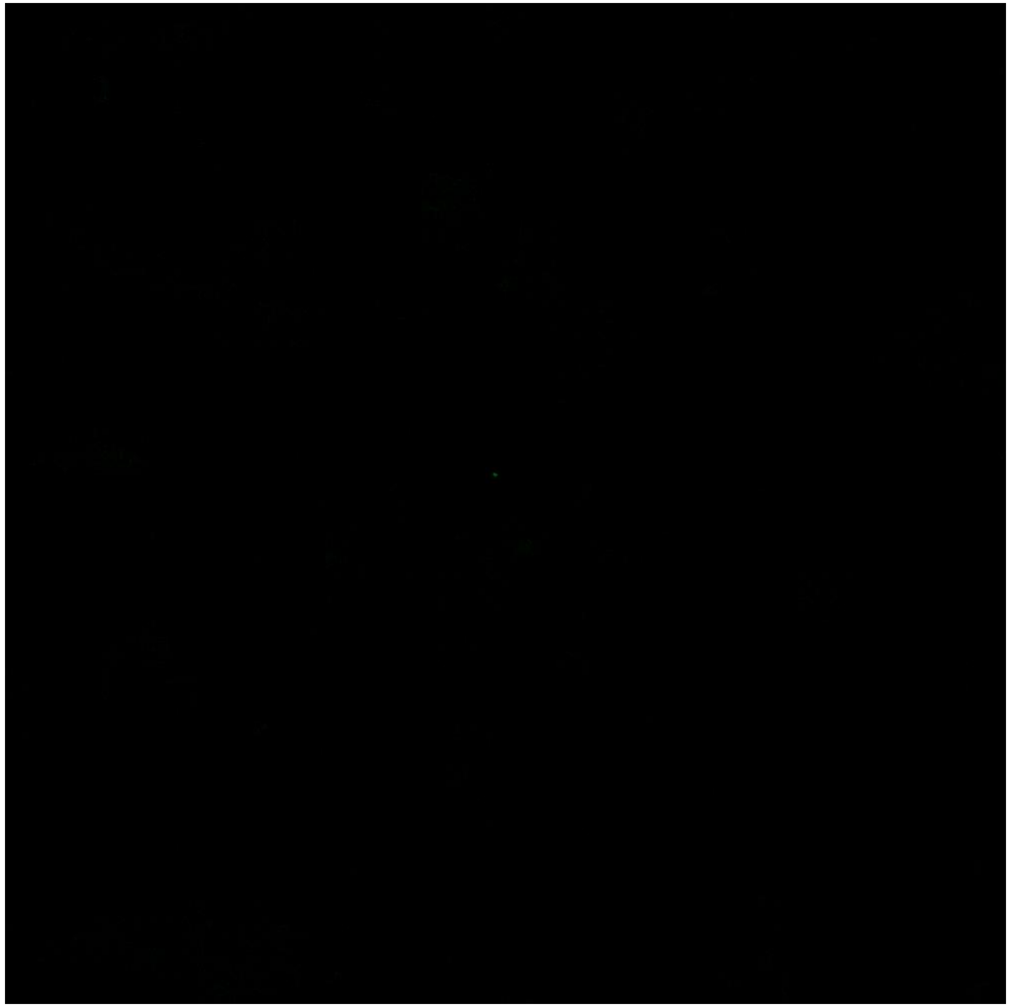

Control:

WT + DMSO (0.1%)

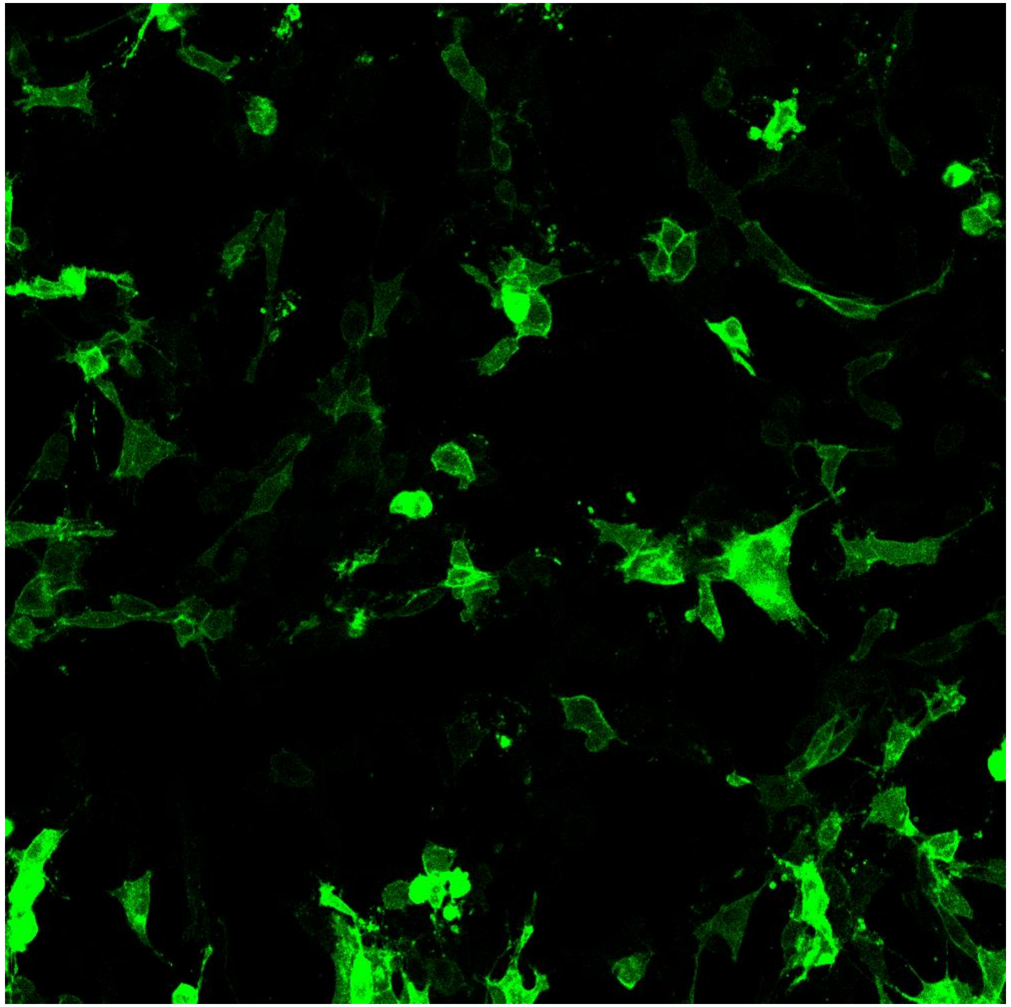

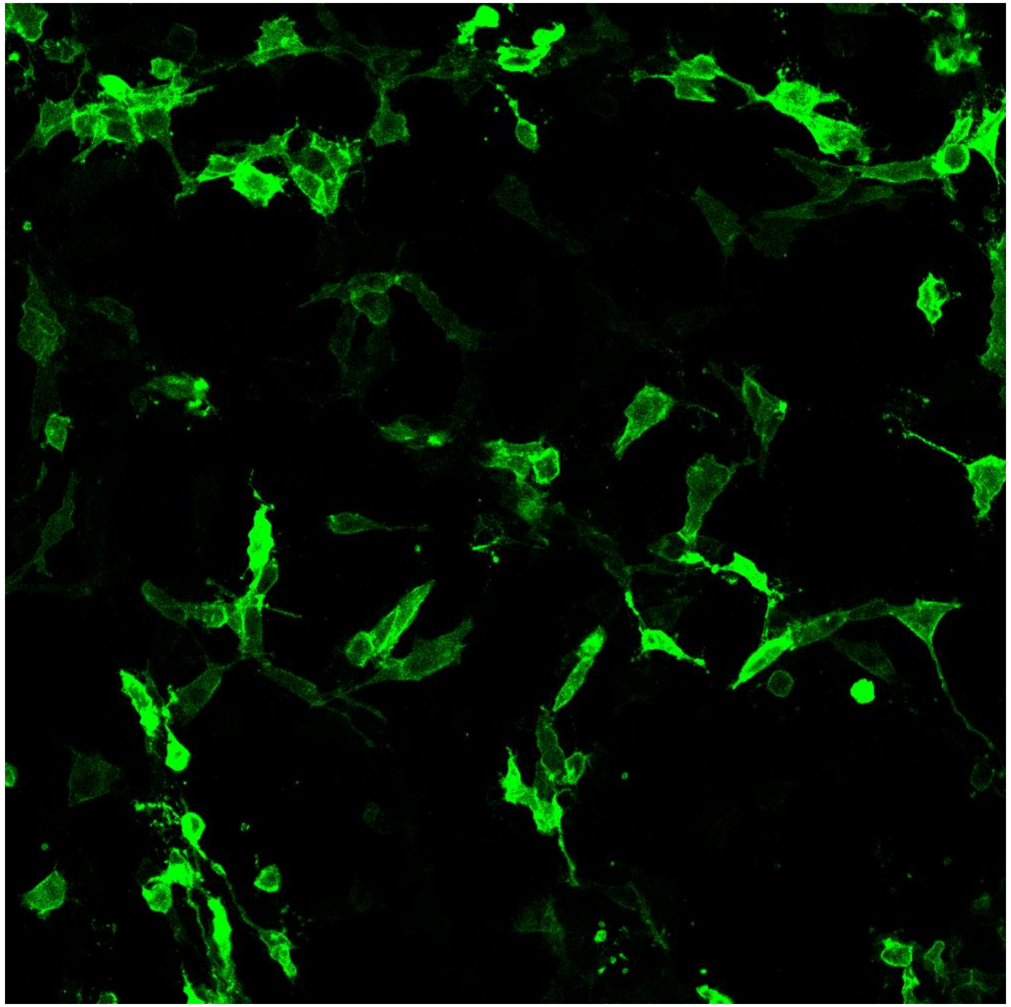

Control:

WT + DMSO (0.1%)

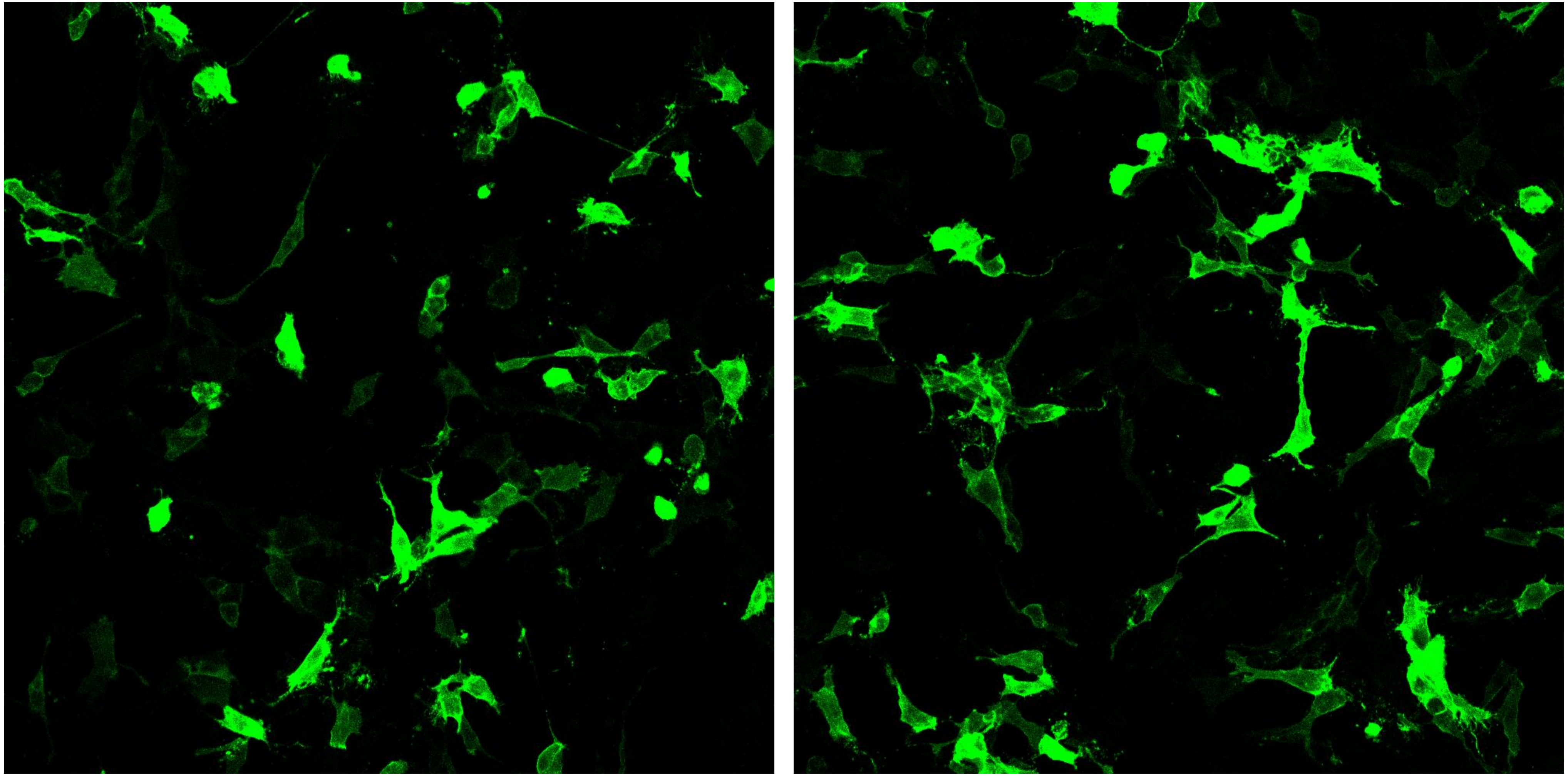

Control:

WT + DMSO (0.1%)

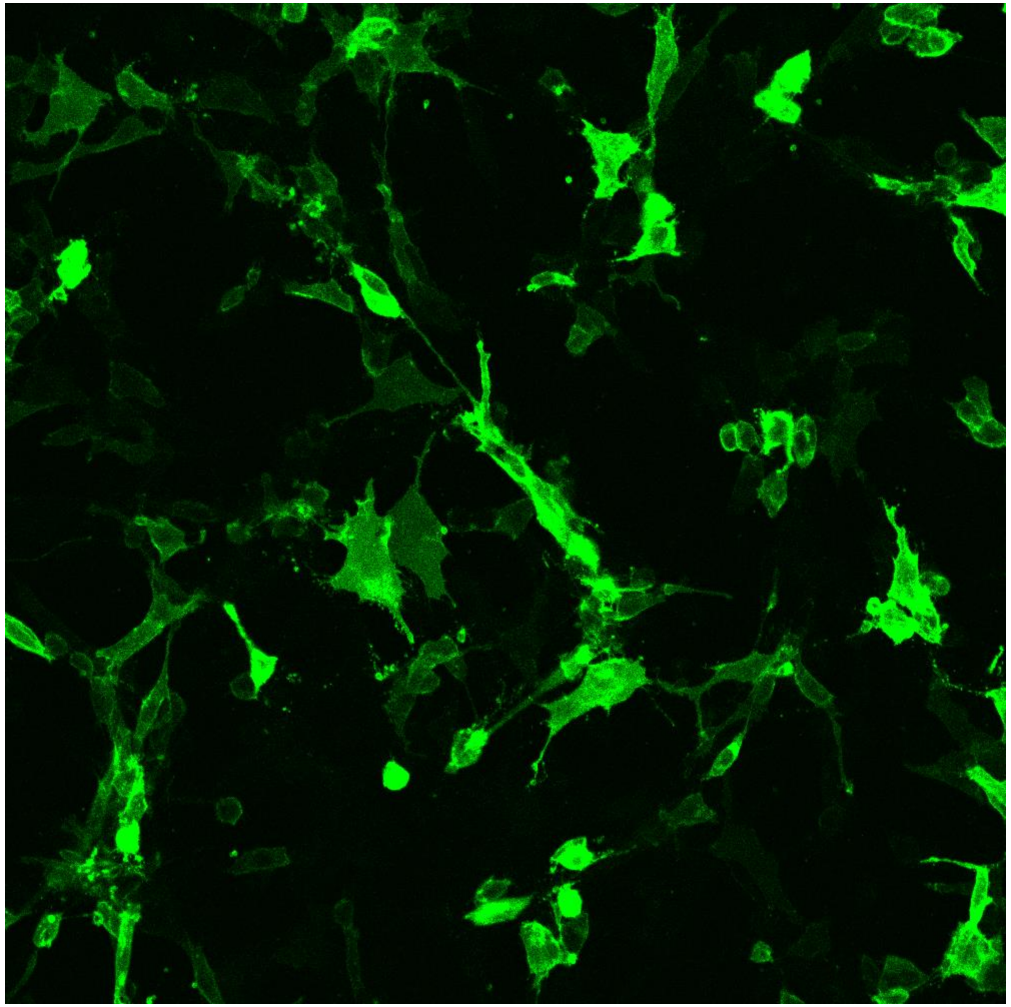

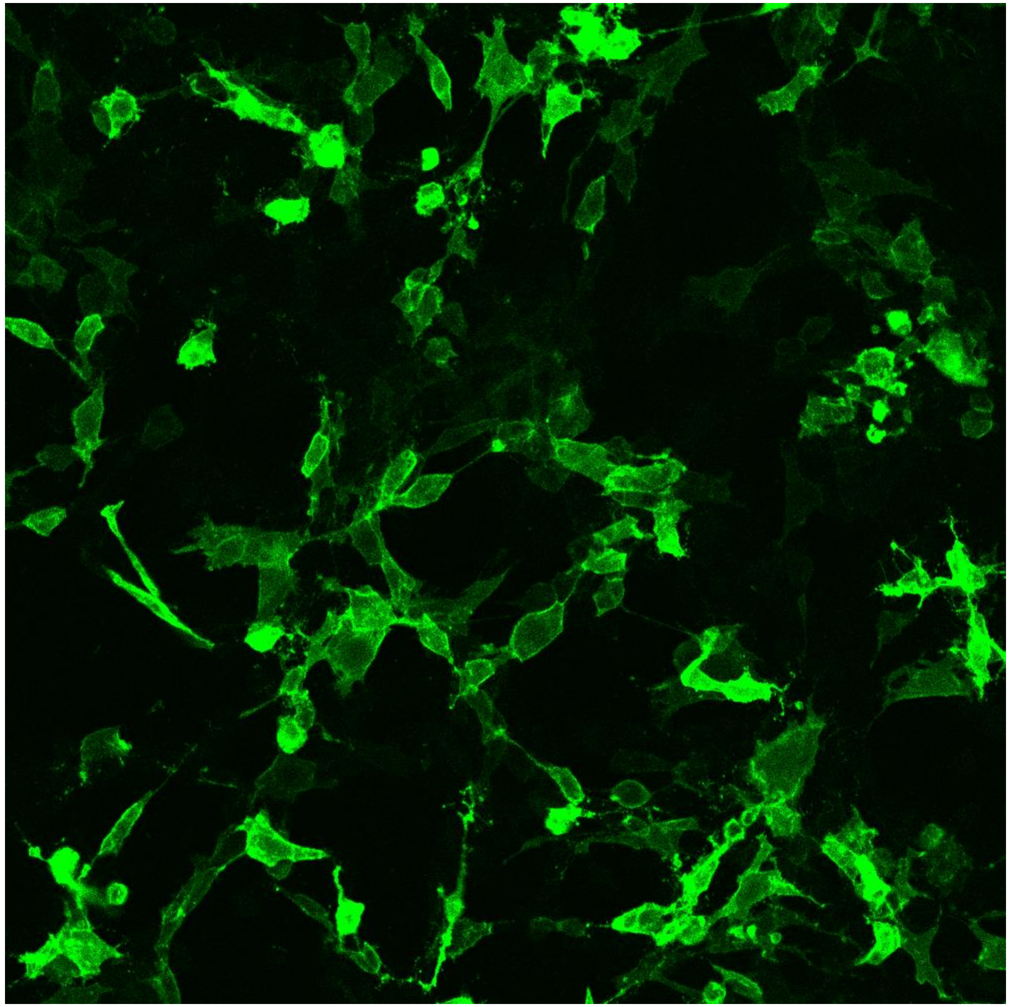

Control:

WT + DMSO (0.1%)

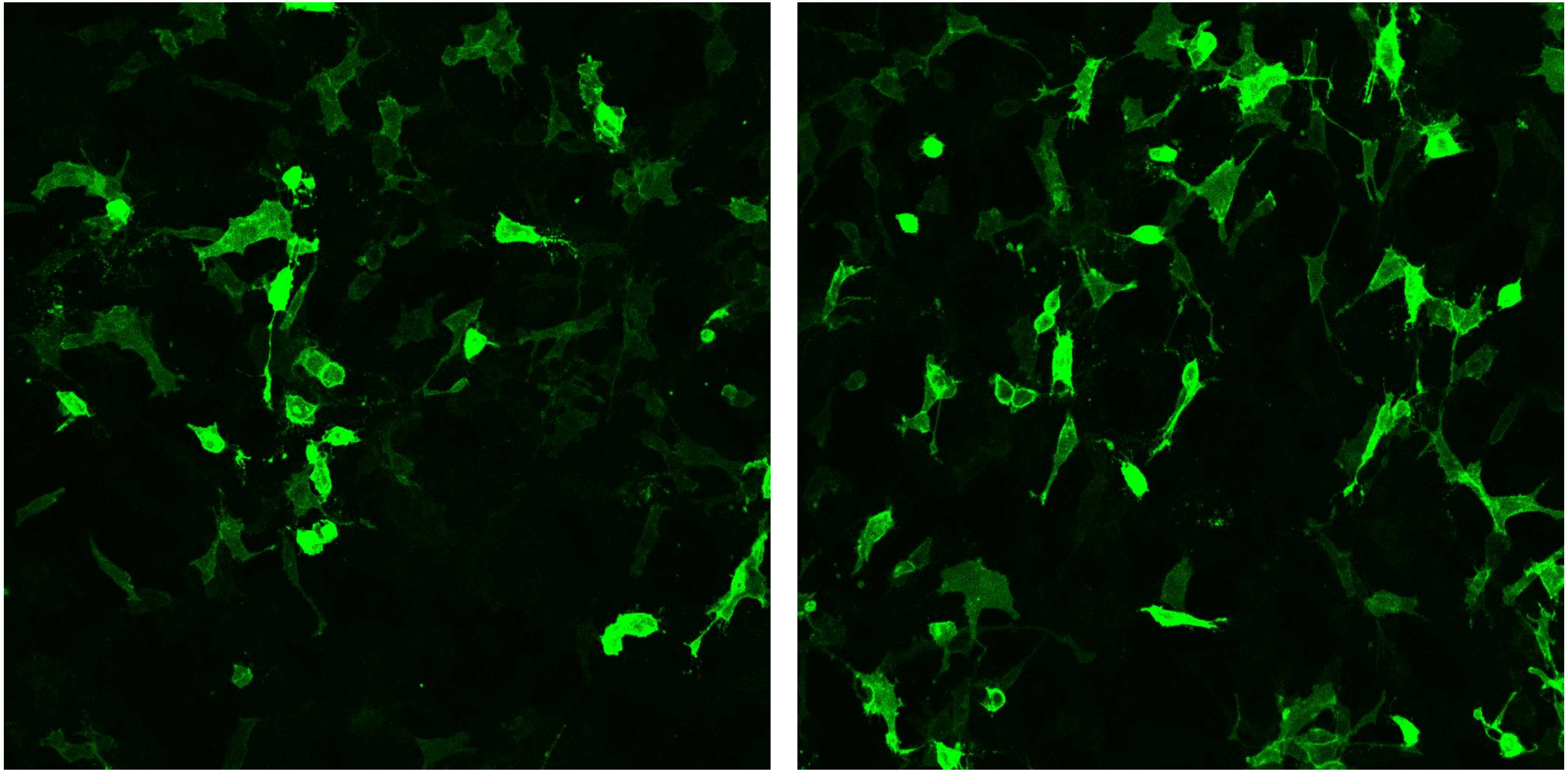

WT + 5 μM 9-*cis*-retinal

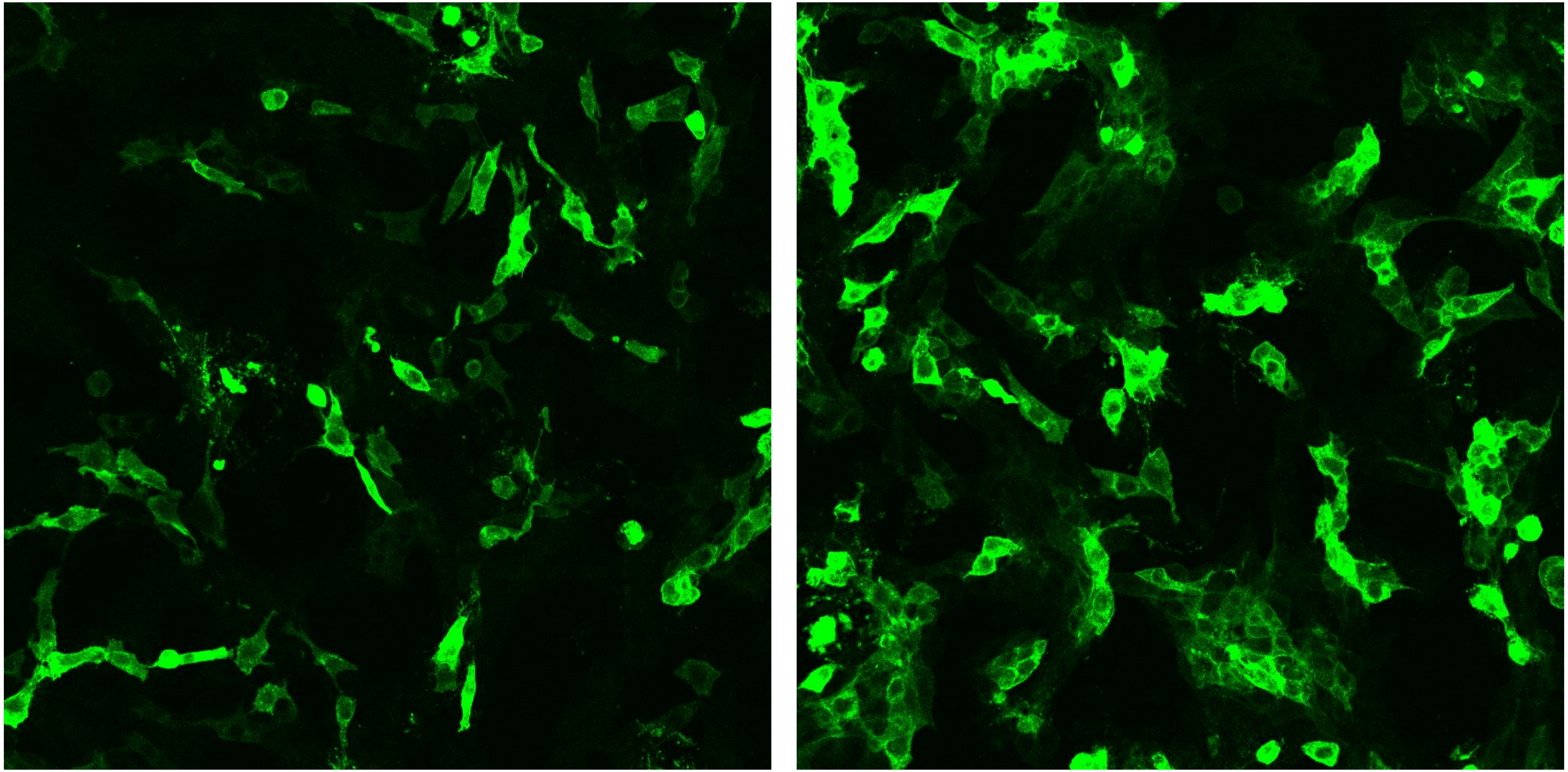

WT + 40 µM YC-001

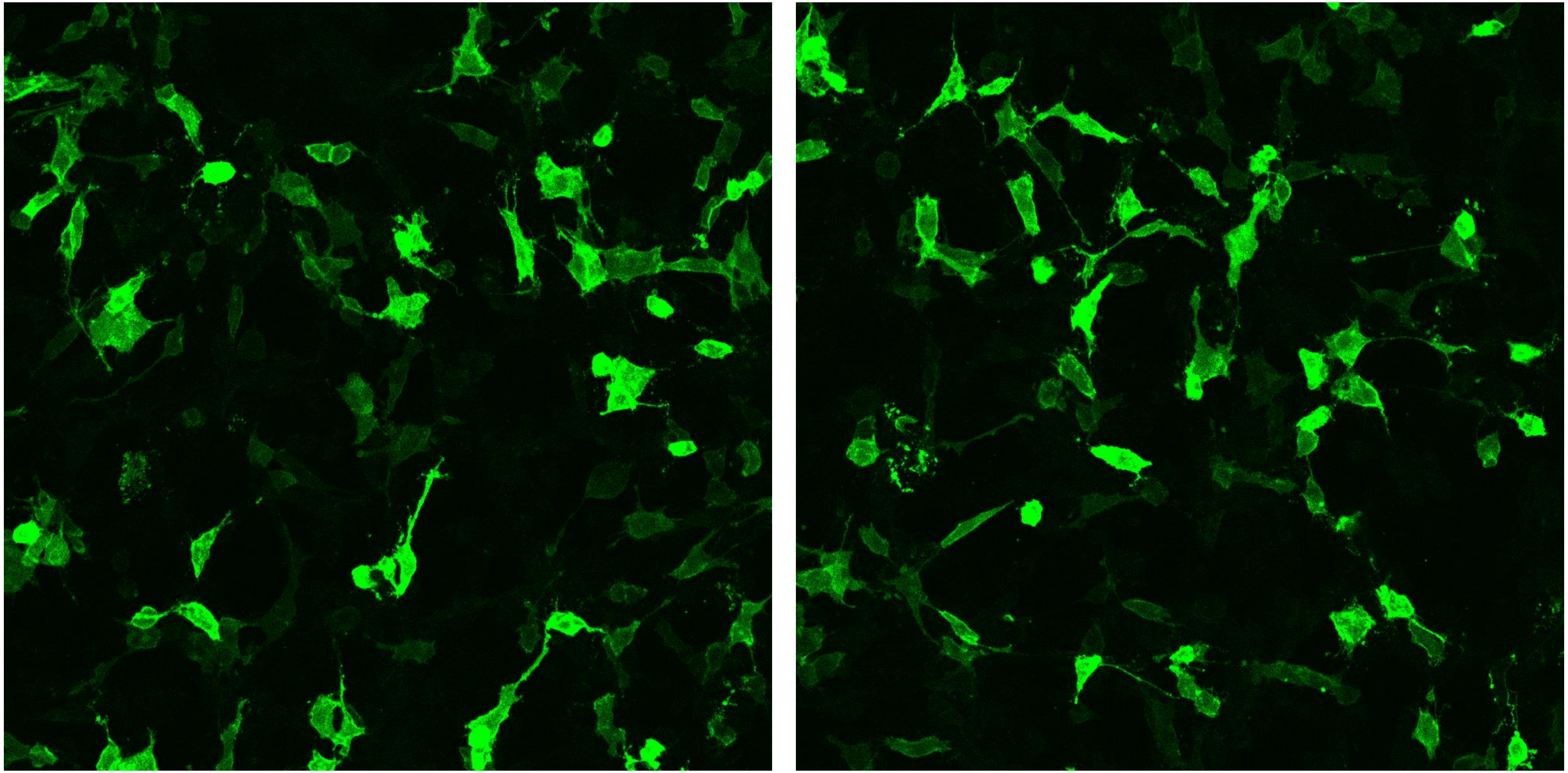

WT + 20 µM F5257-0462

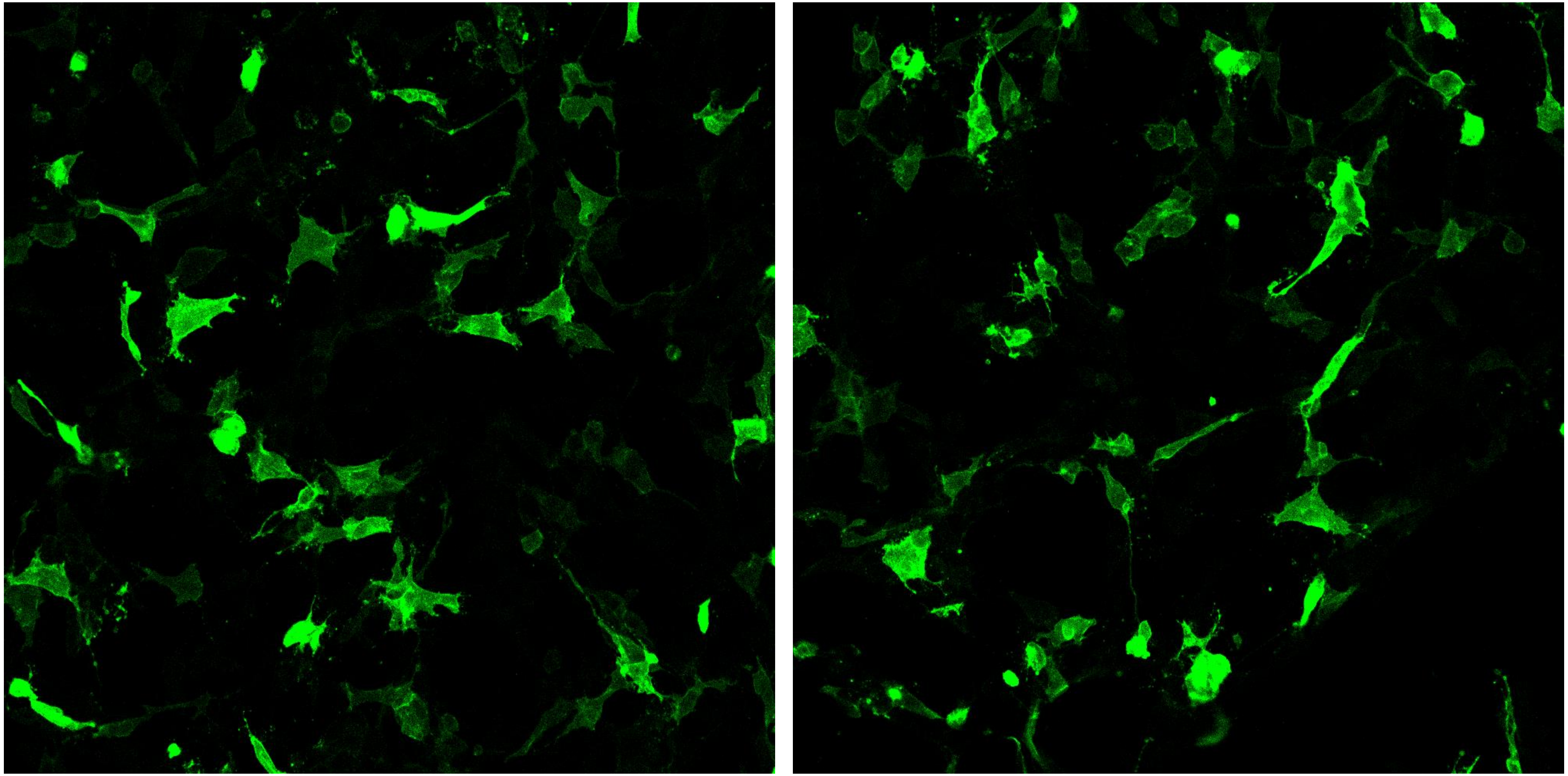

T4K + DMSO (0.1%)

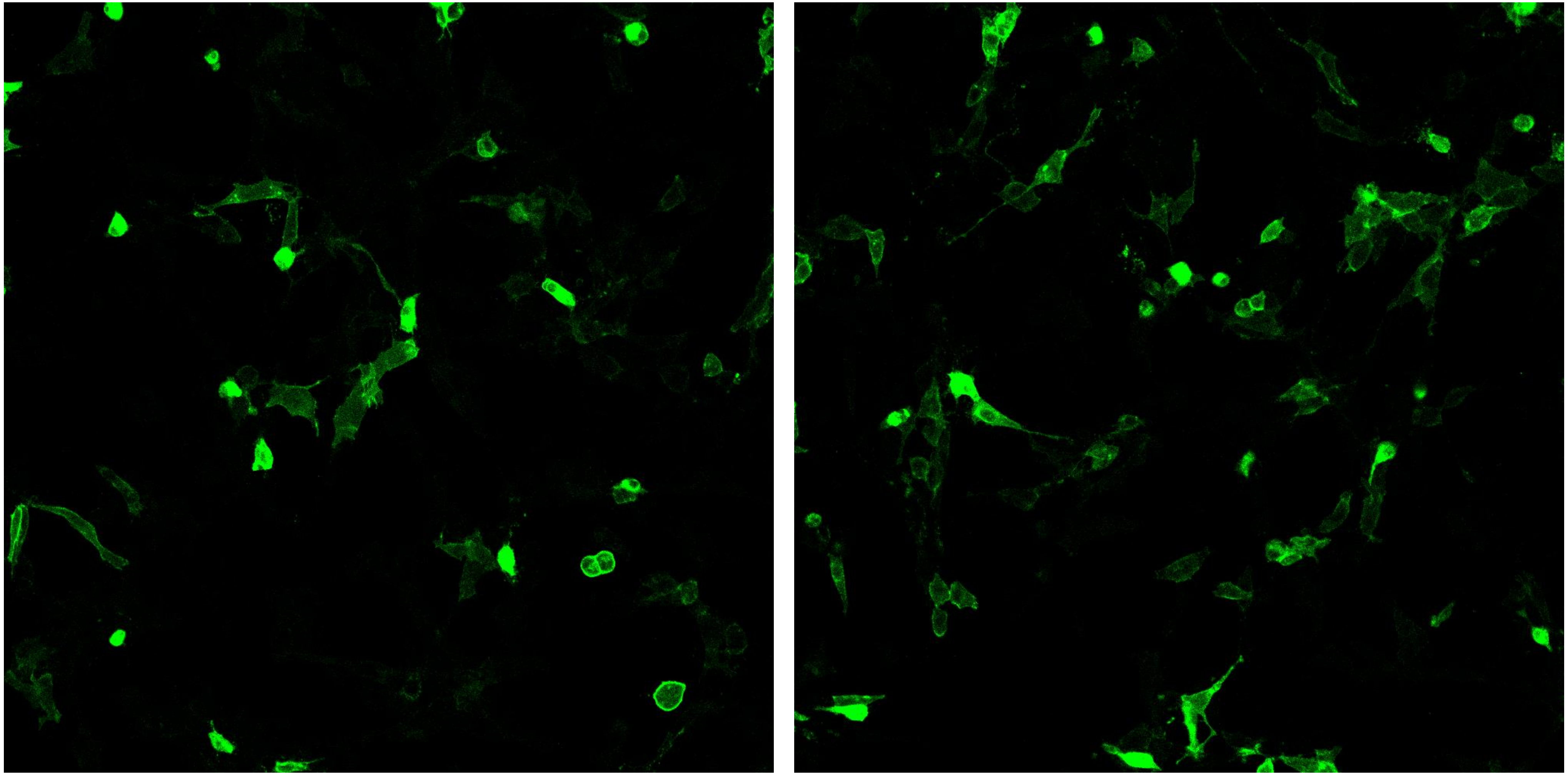

T4K + DMSO (0.1%)

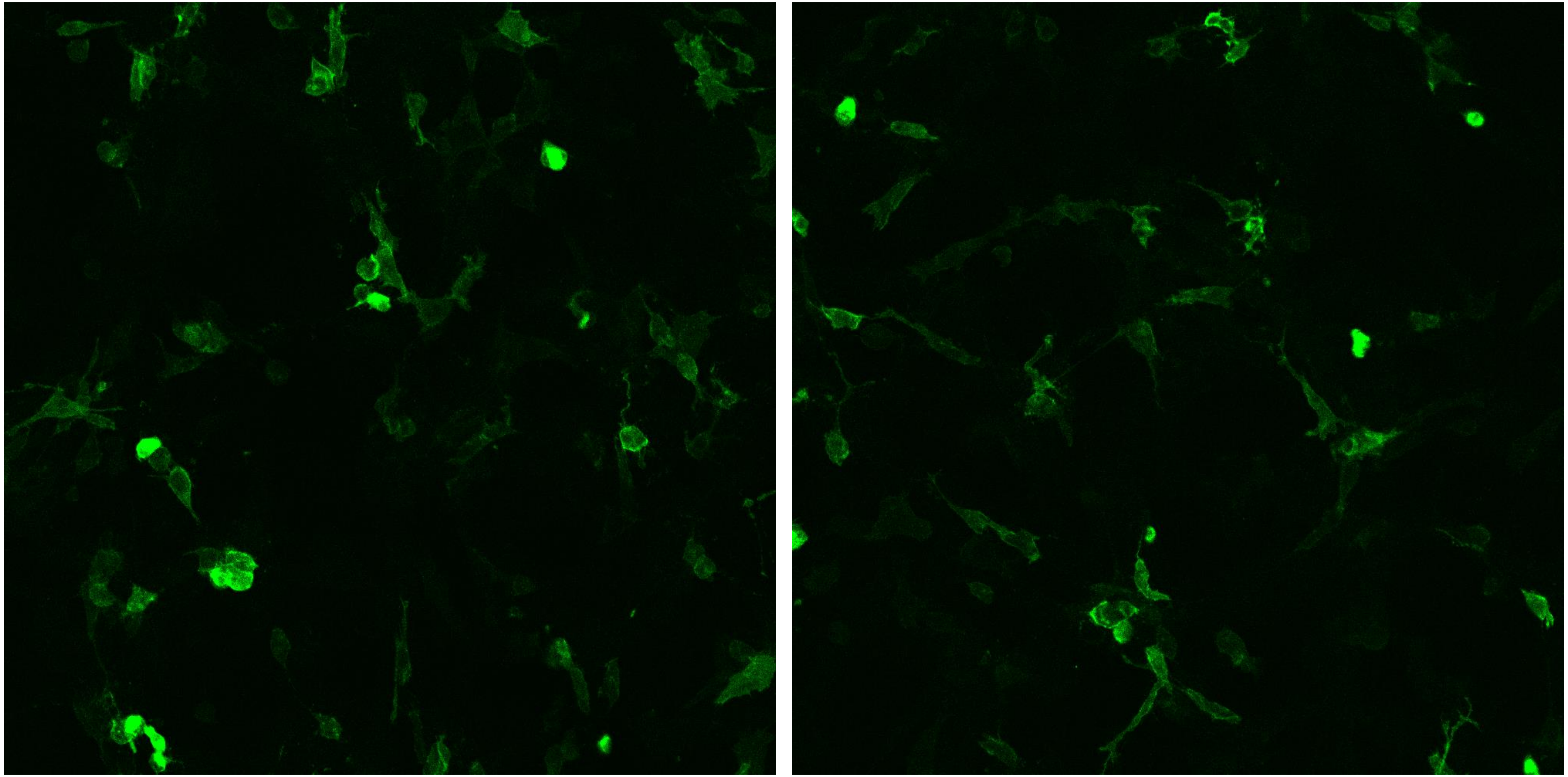

T4K + 5 µM 9-*cis*-retinal

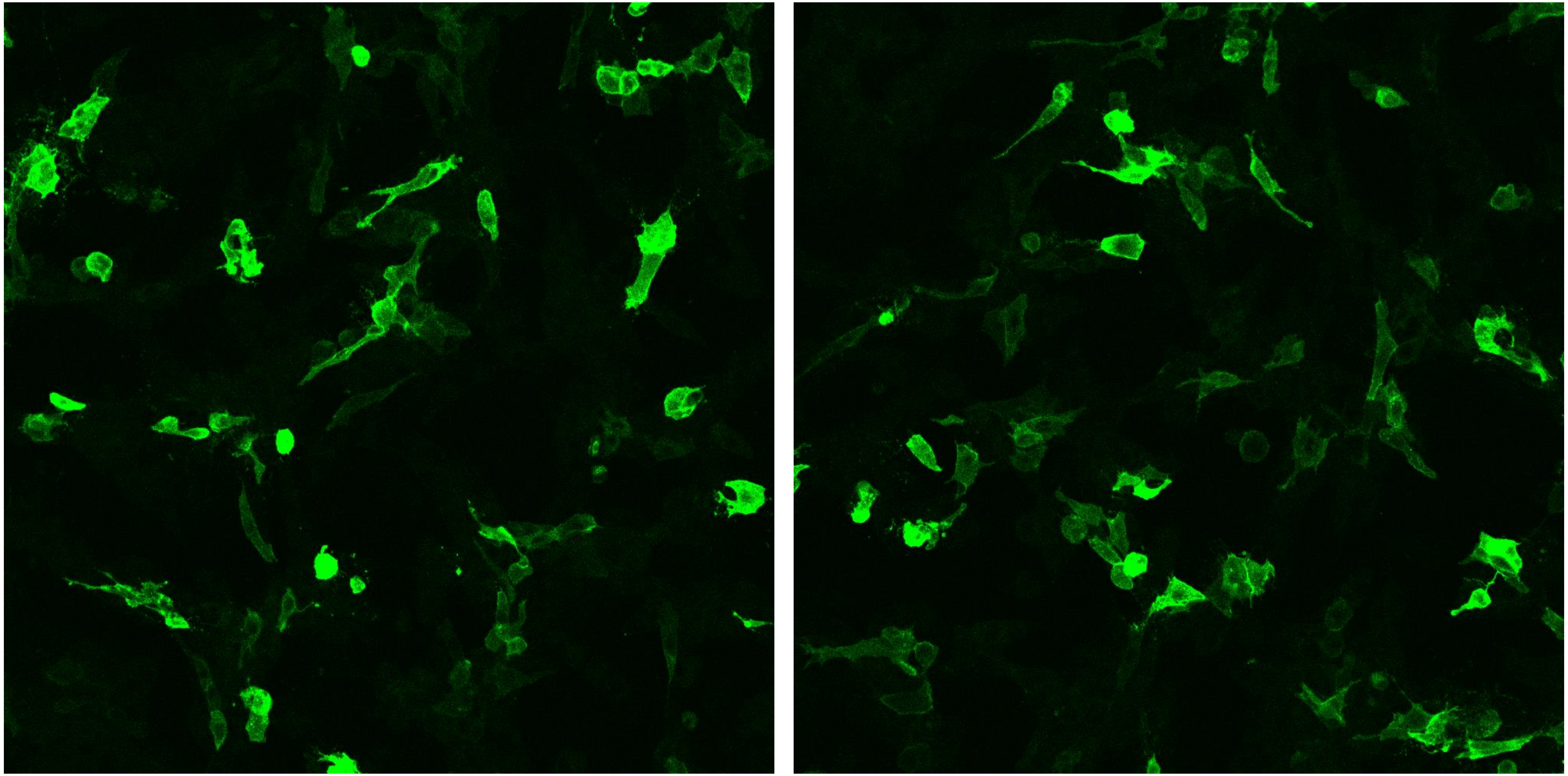

T4K + 40 μM YC-001

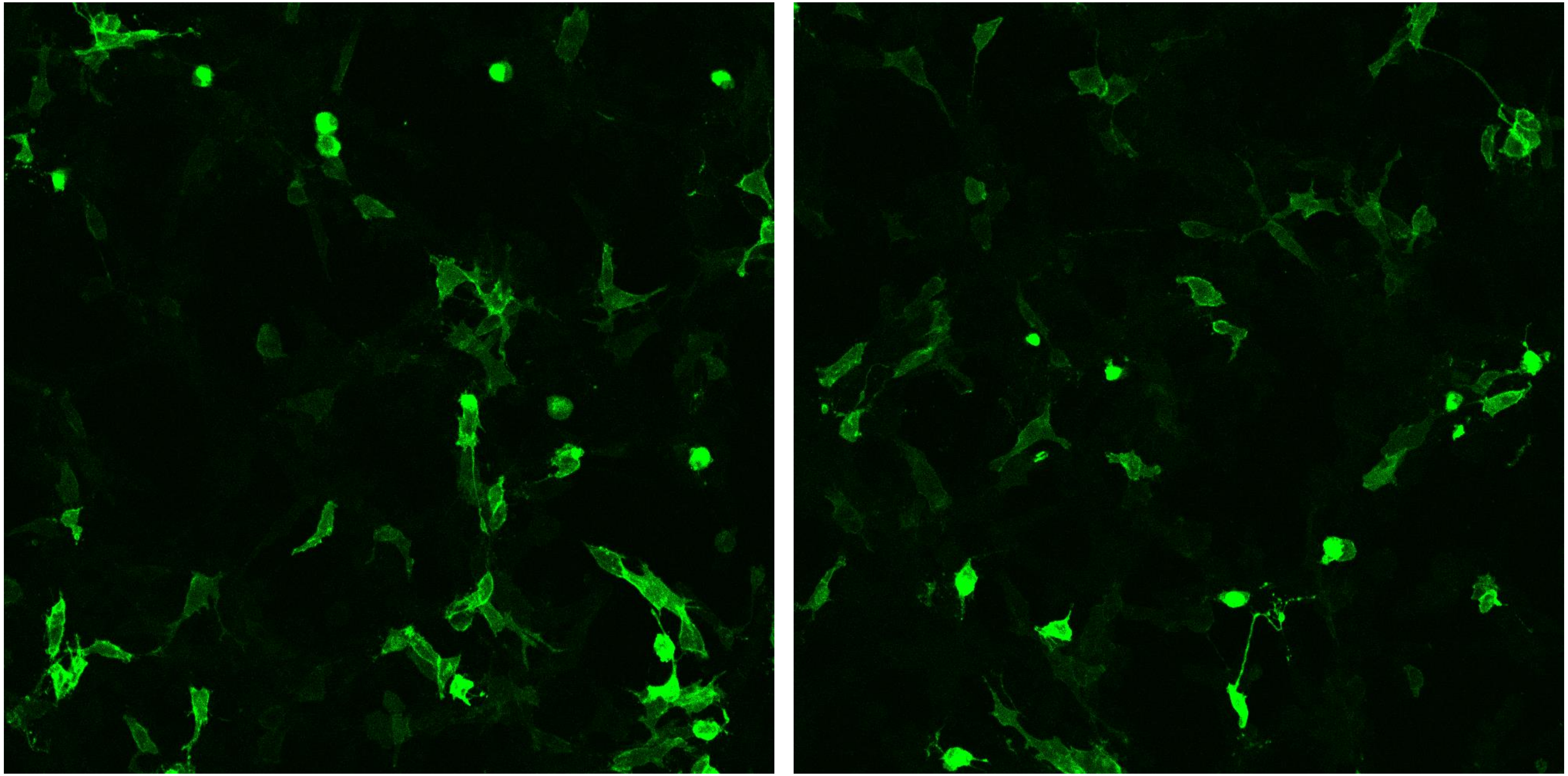

T4K + 20 μM F5257-0462

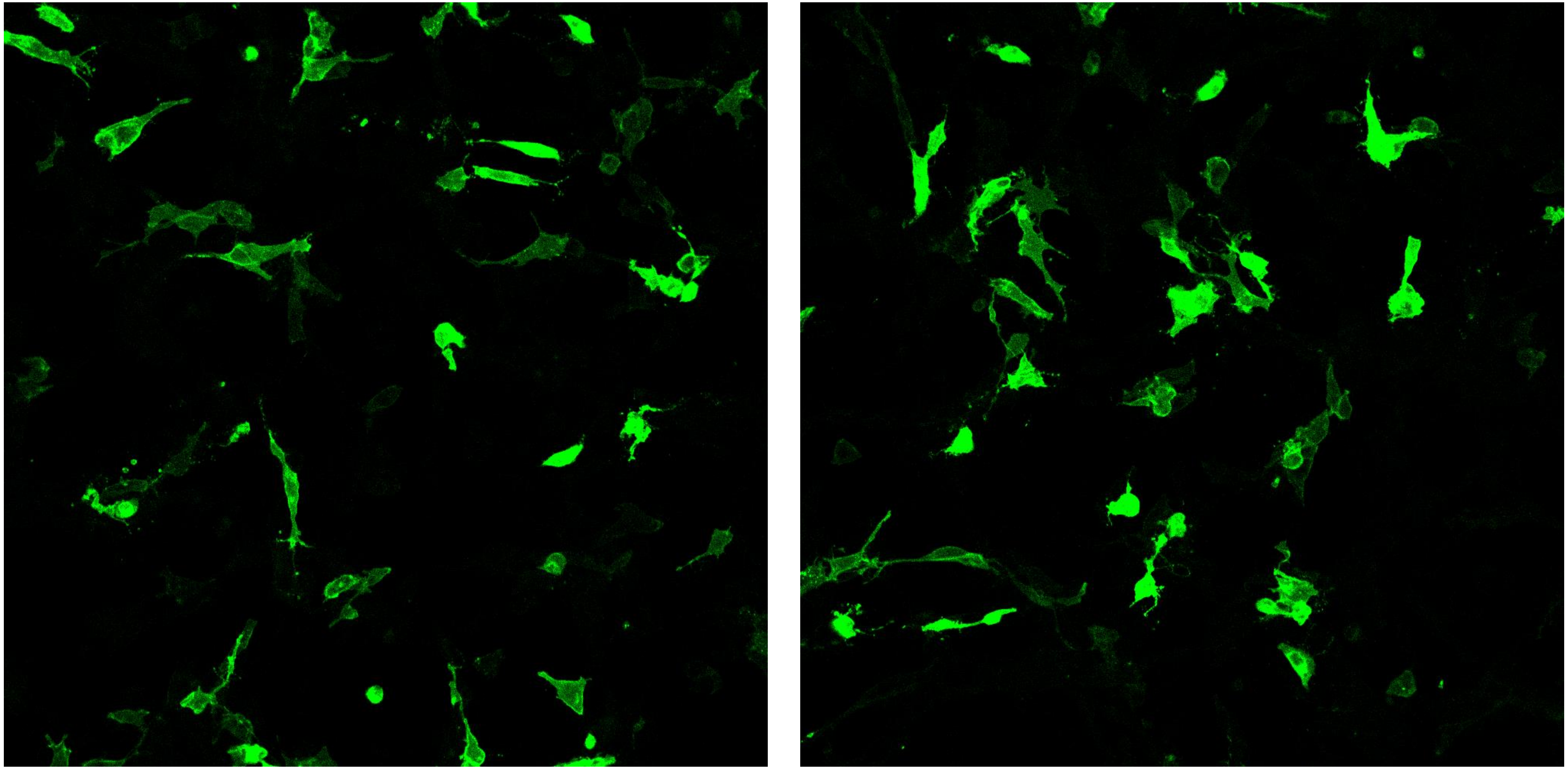

N15S + DMSO (0.1%)

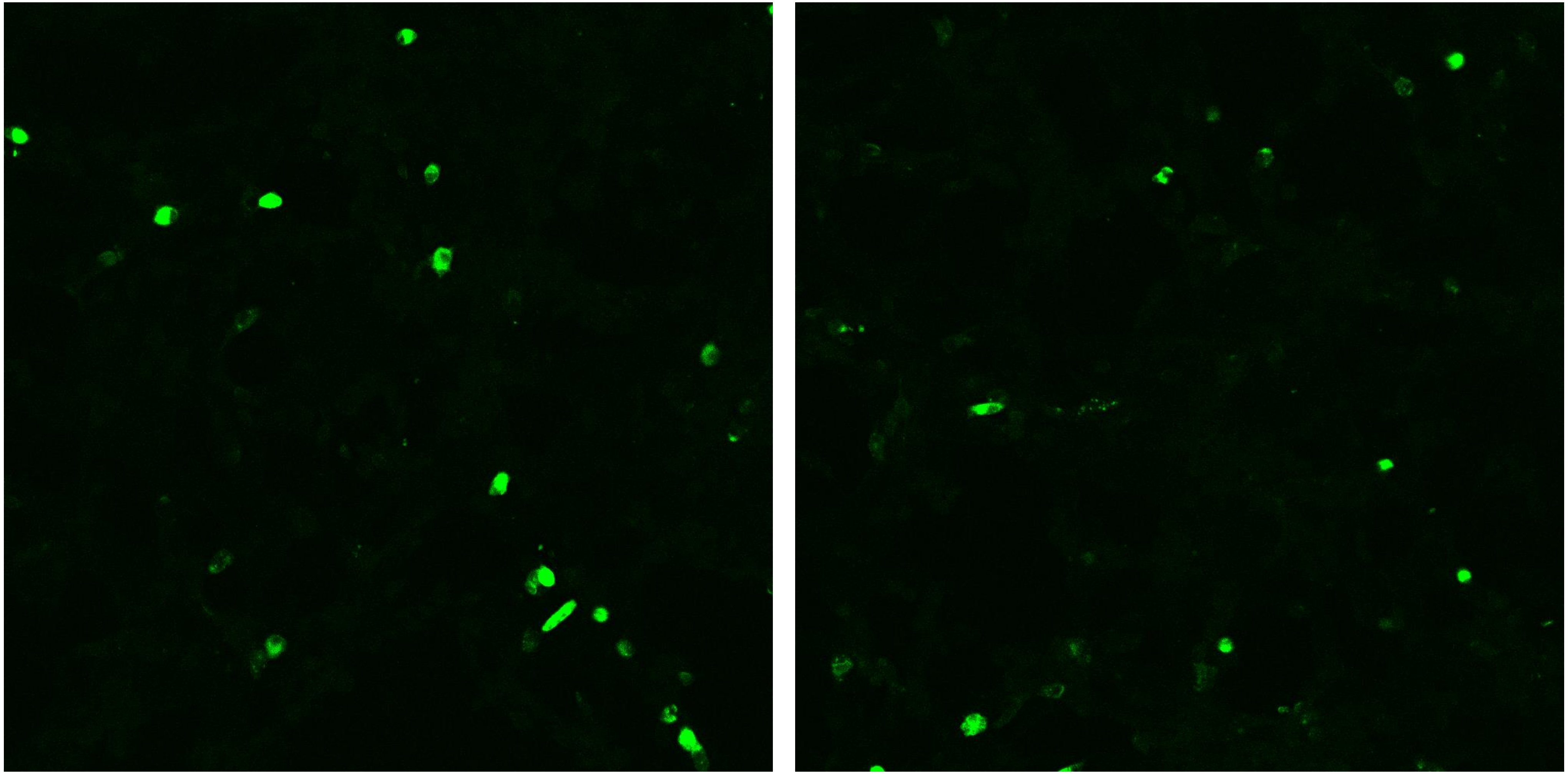

N15S + DMSO (0.1%)

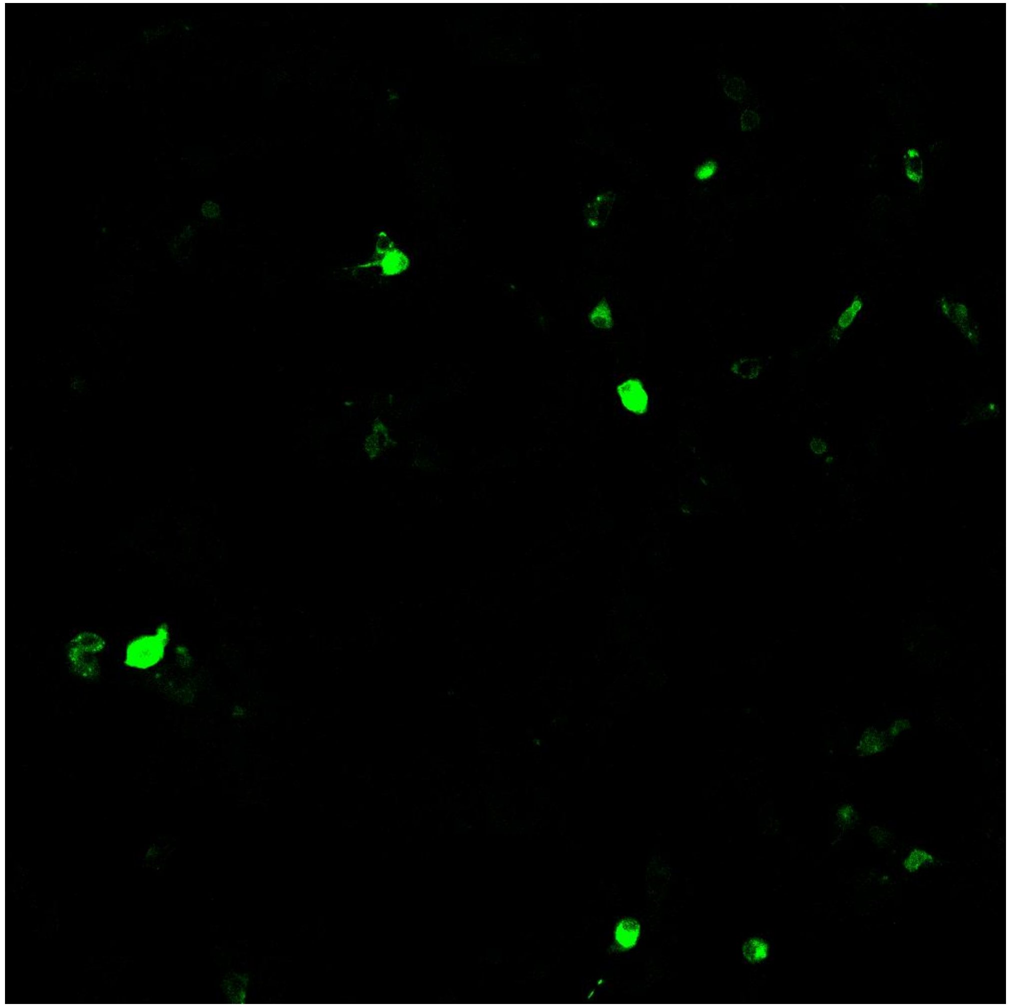

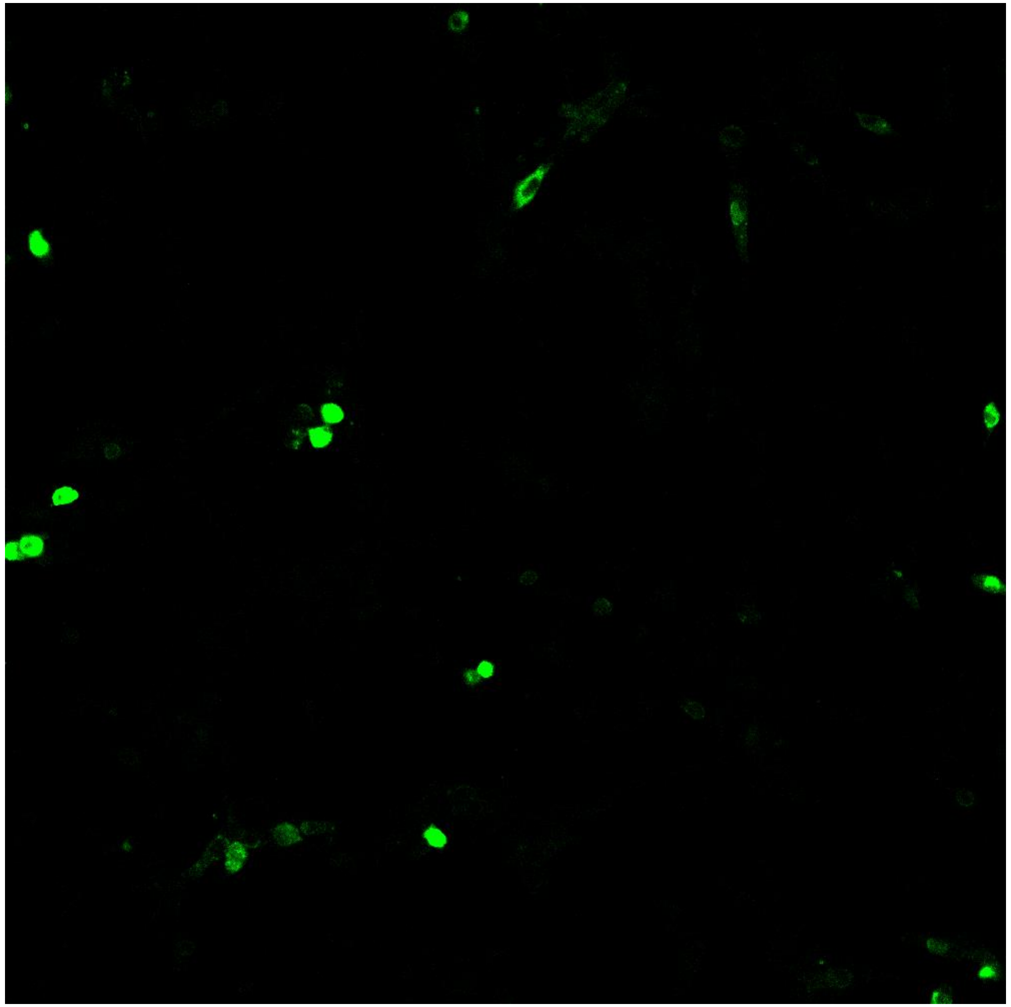

N15S + 5 µM 9-*cis*-retinal

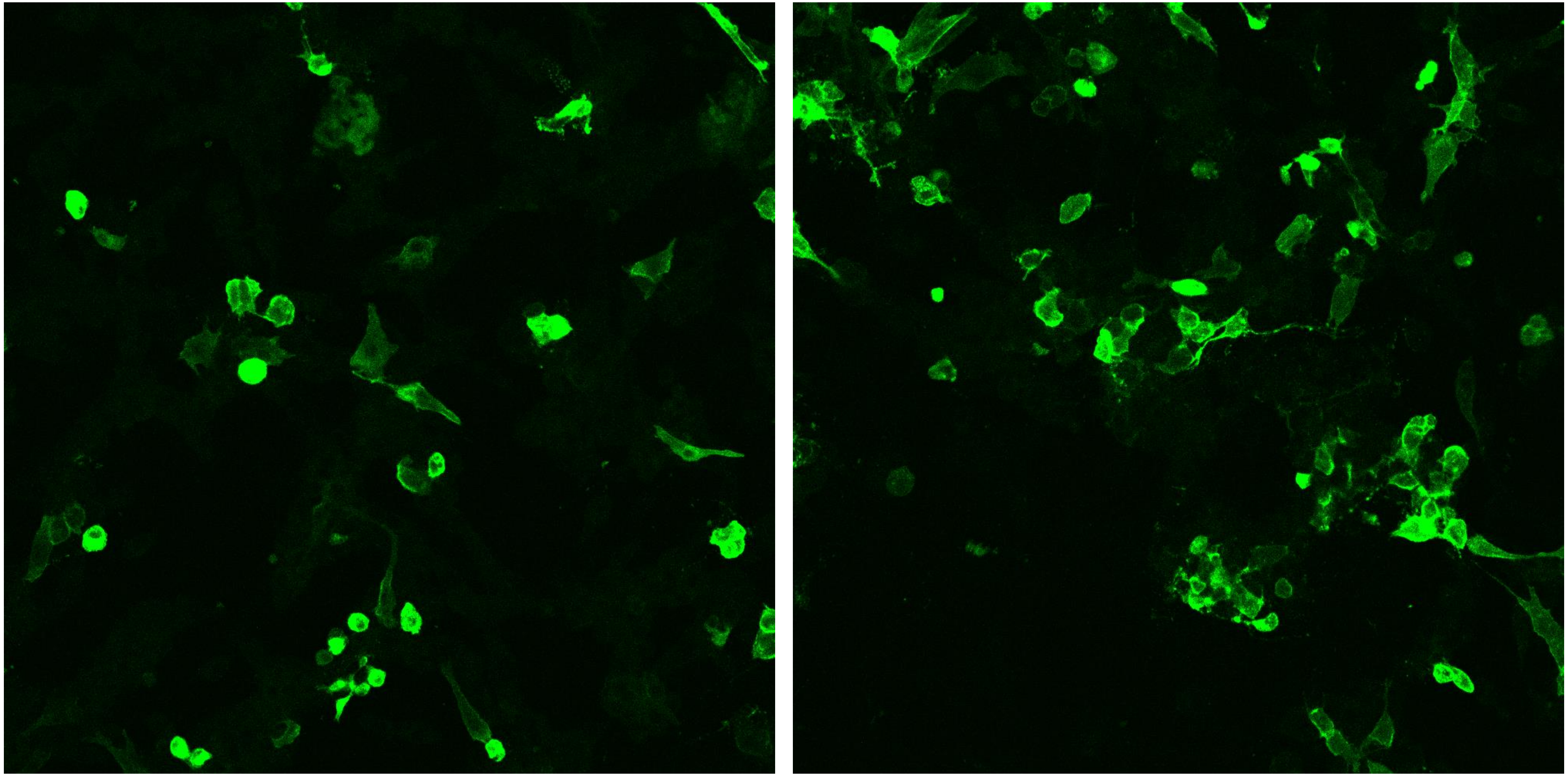

N15S + 40 μM YC-001

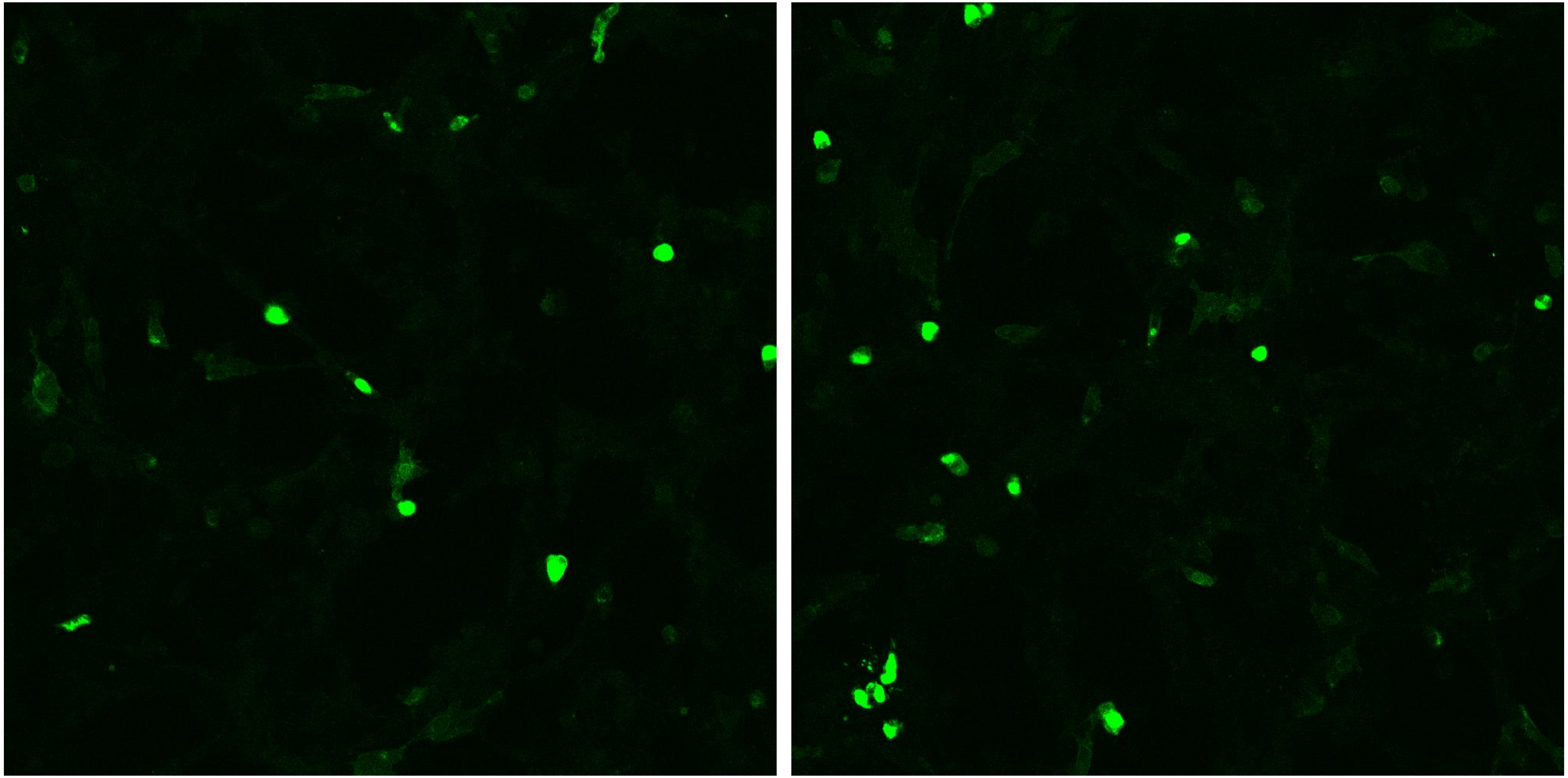

N15S + 20 µM F5257-0462

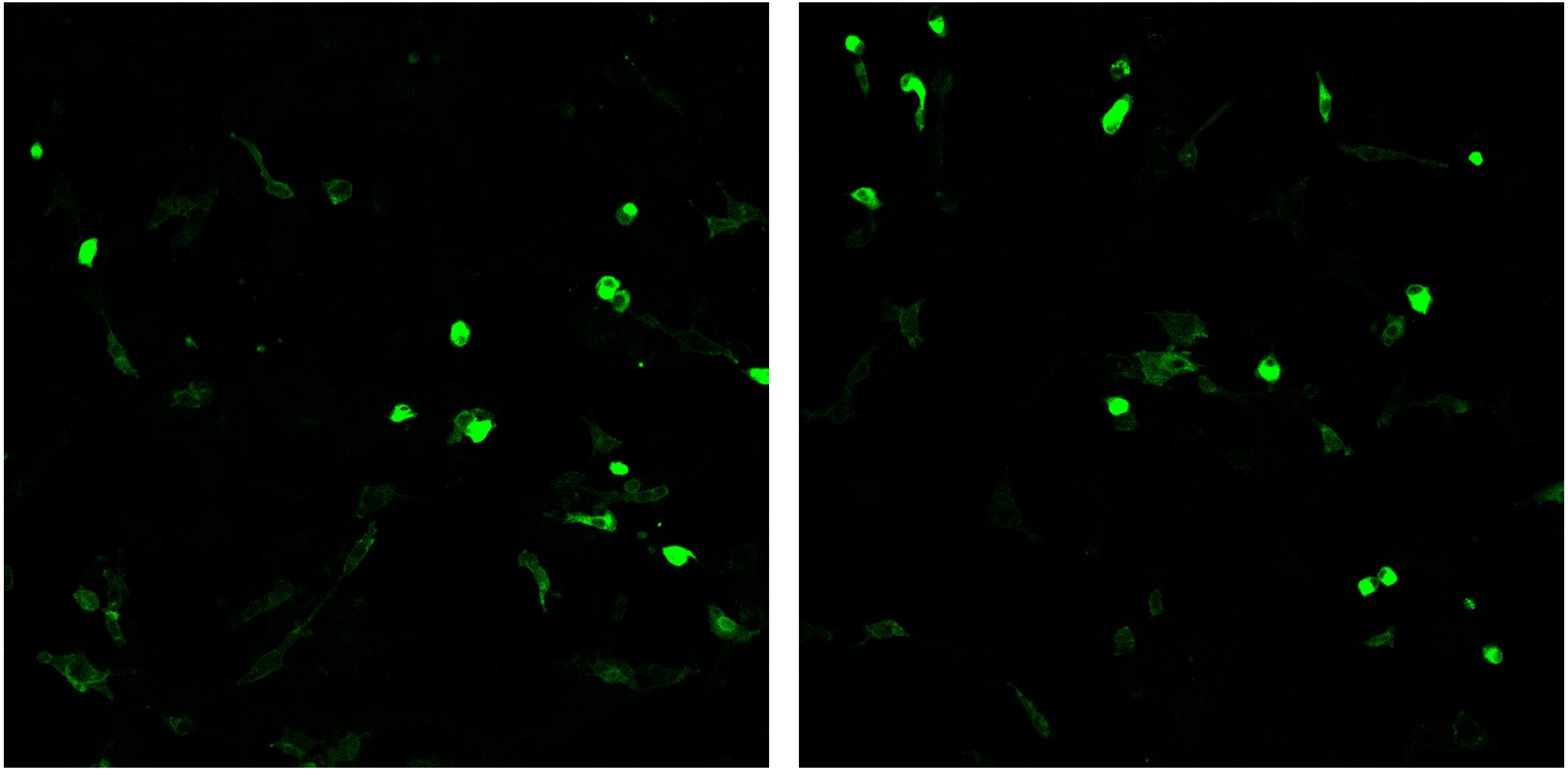

T17M + DMSO (0.1%)

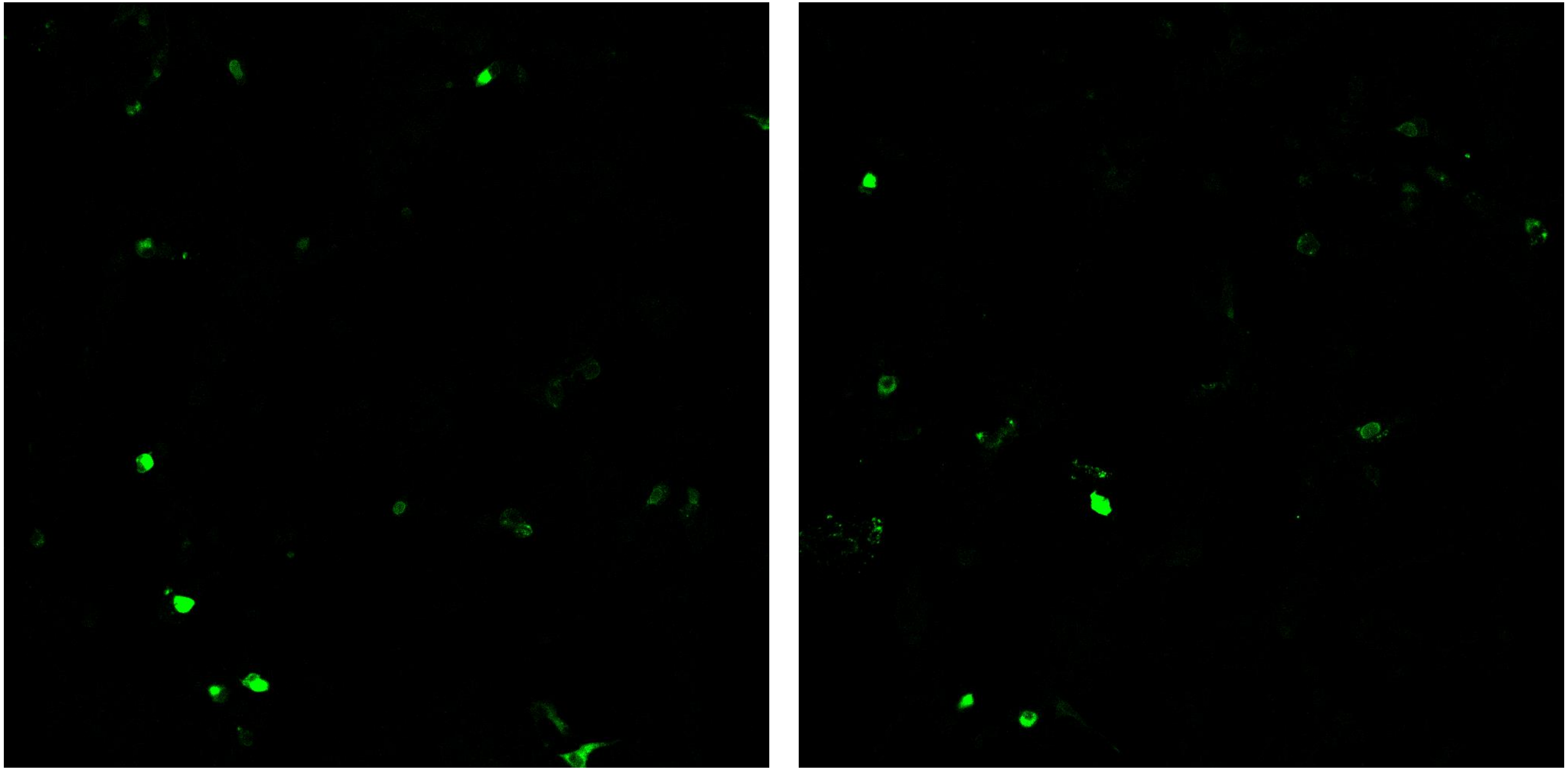

T17M + DMSO (0.1%)

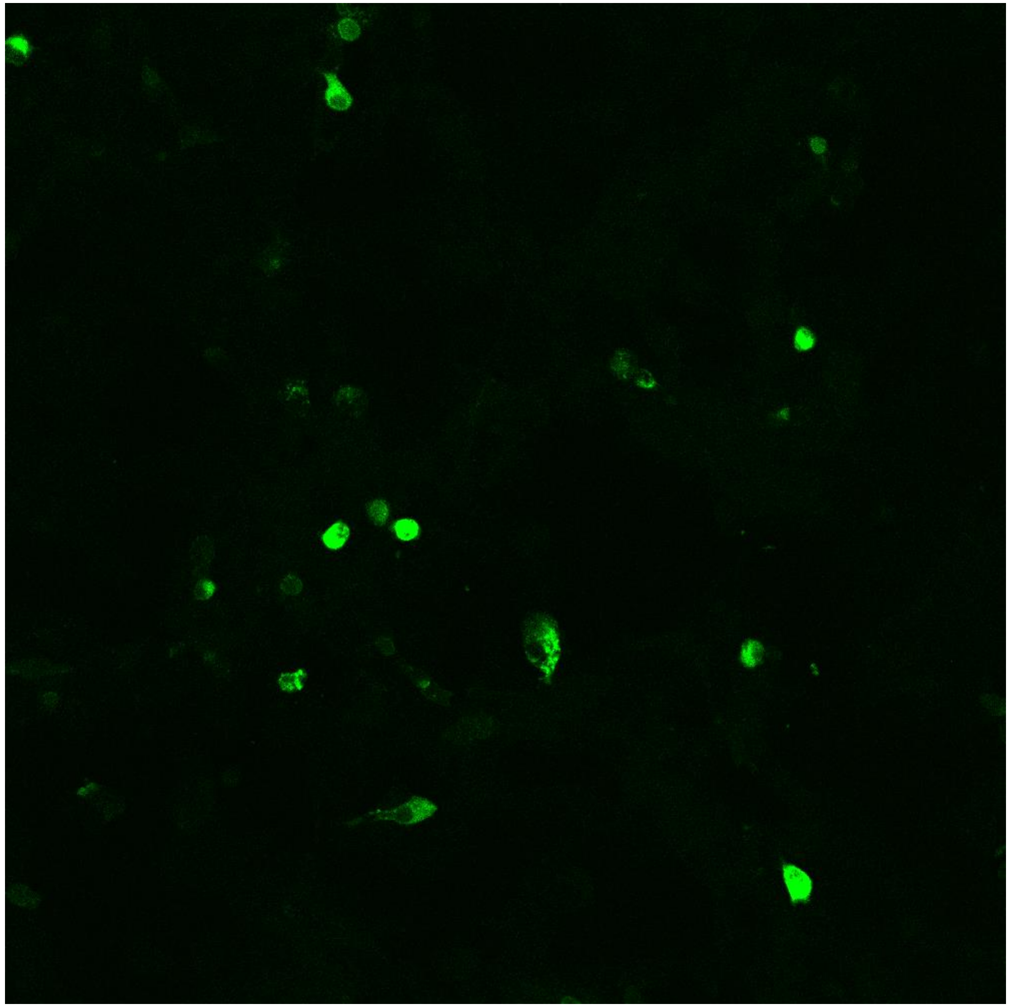

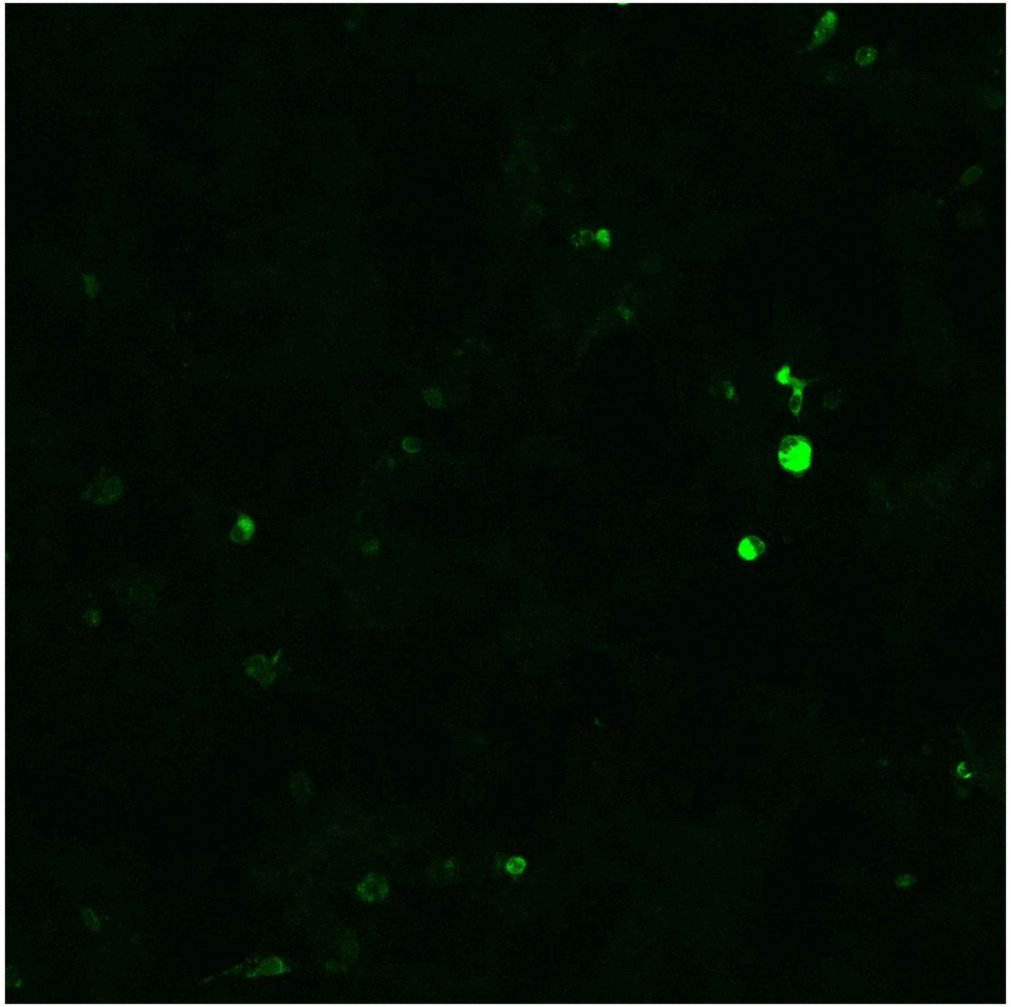

T17M + 5 µM 9-*cis*-retinal

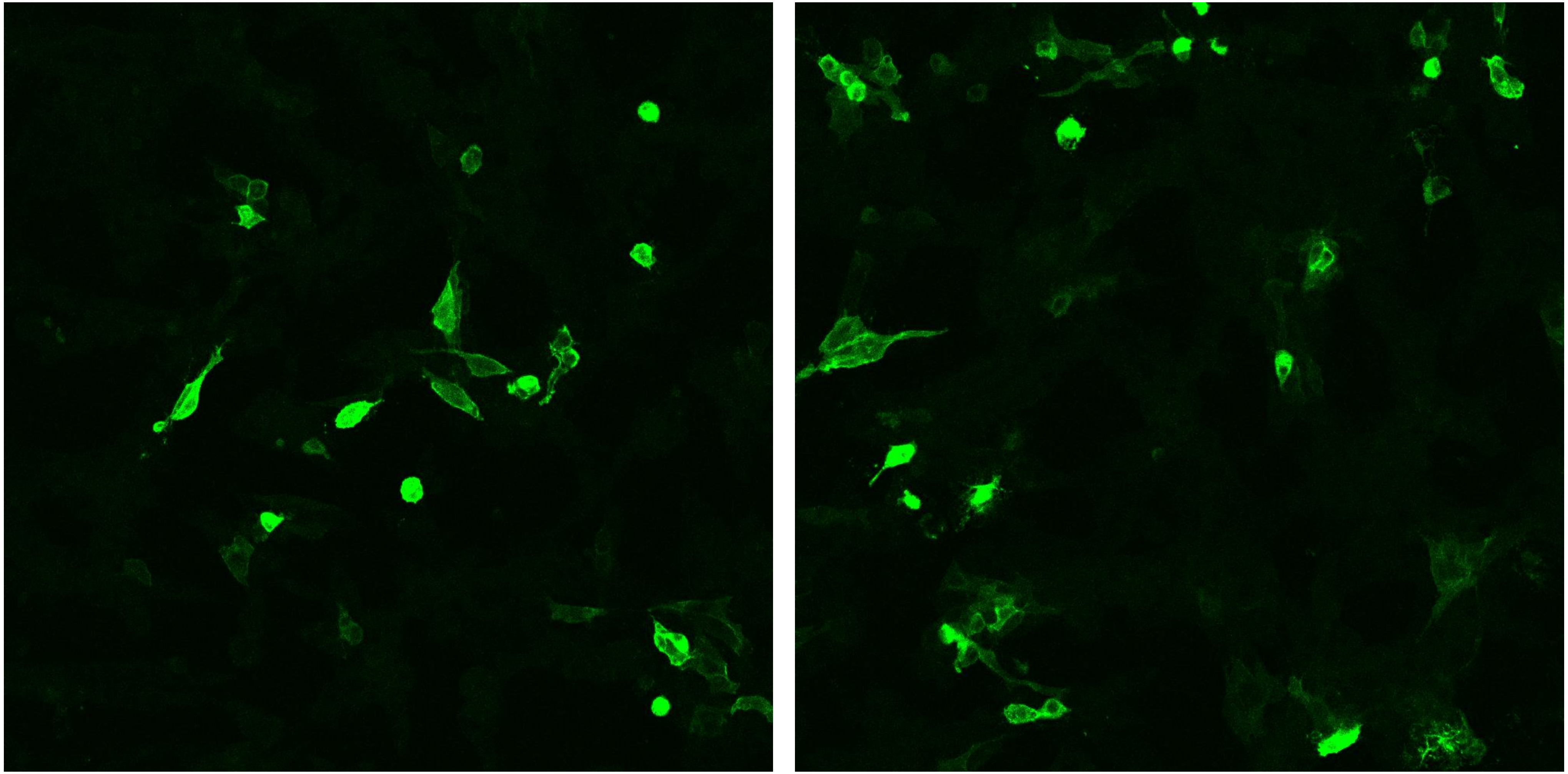

T17M + 40 μM YC-001

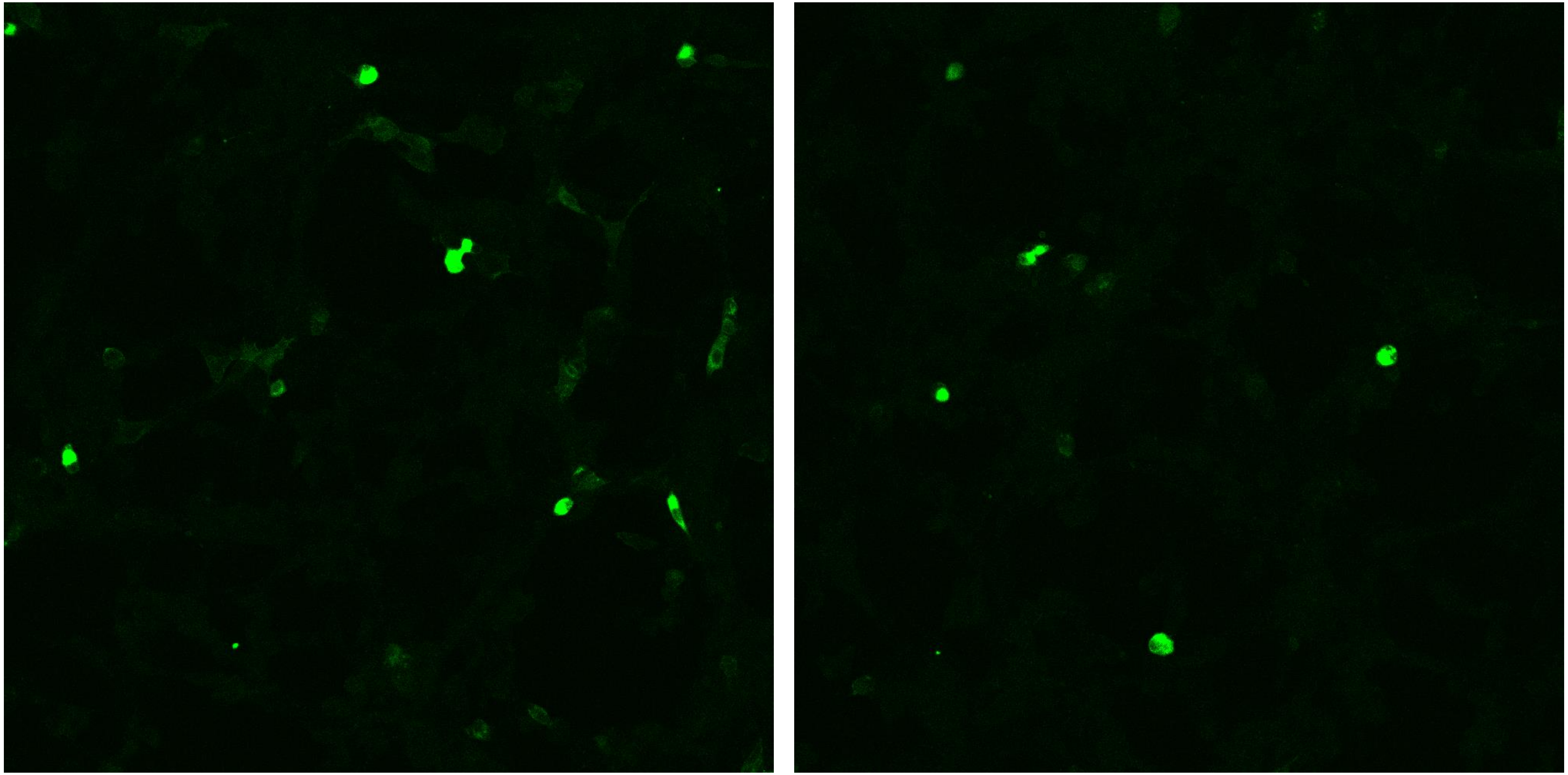

T17M + 20 μM F5257-0462

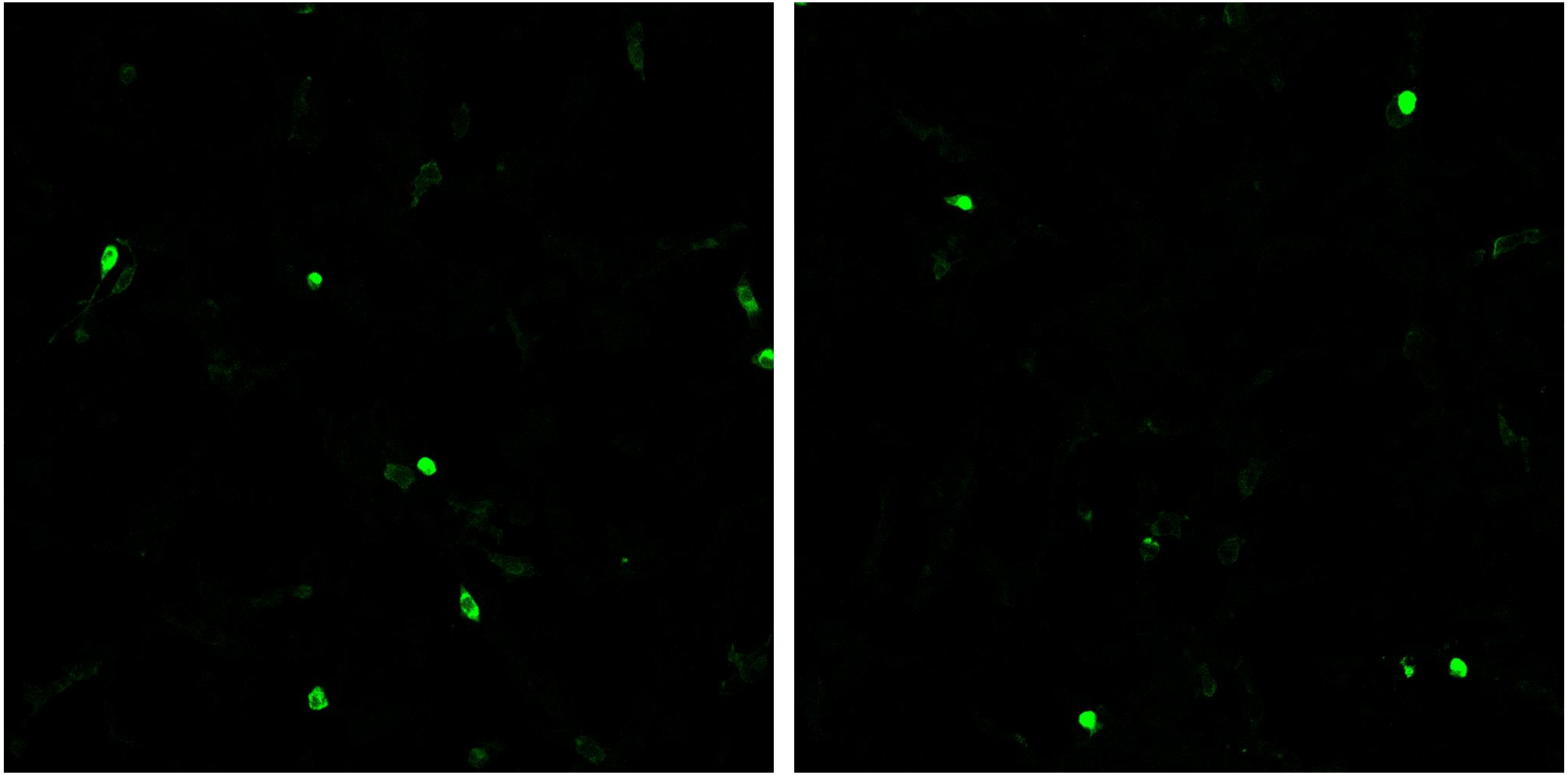

P23H + DMSO (0.1%)

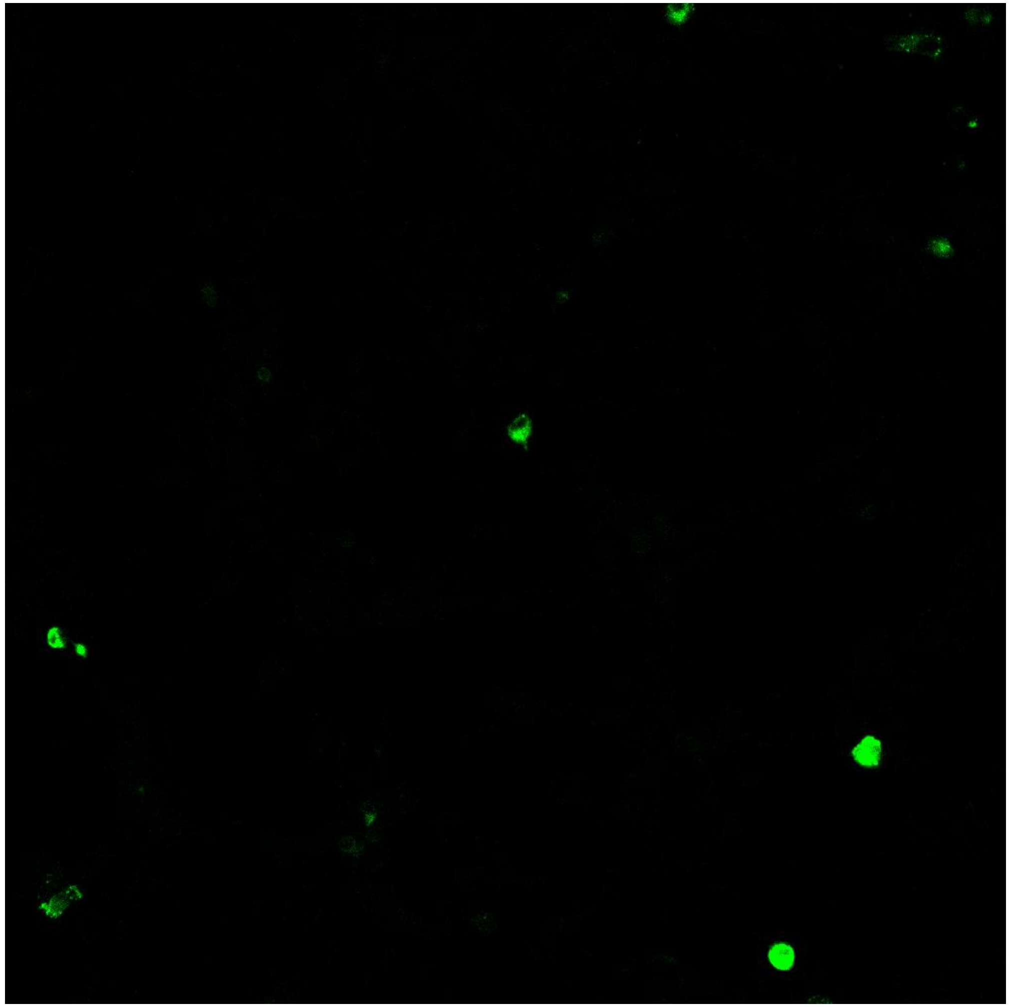

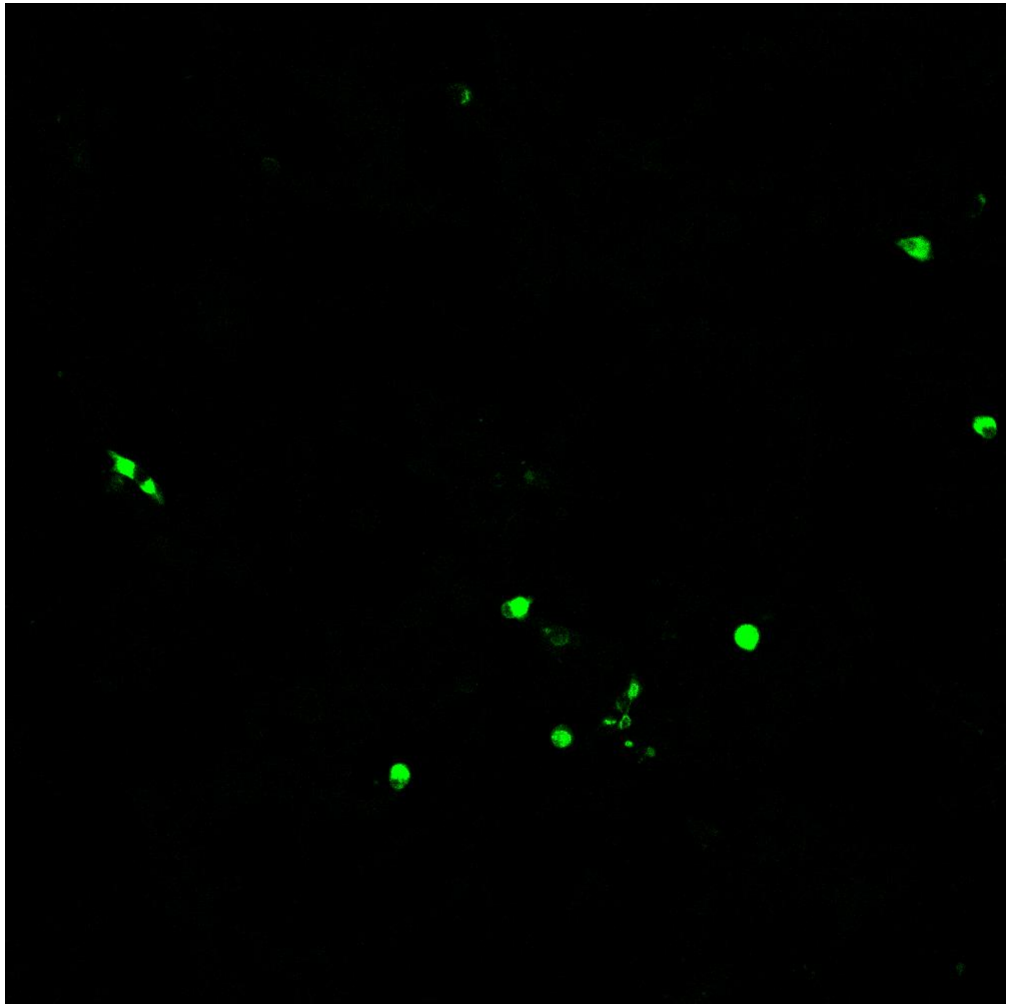

P23H + DMSO (0.1%)

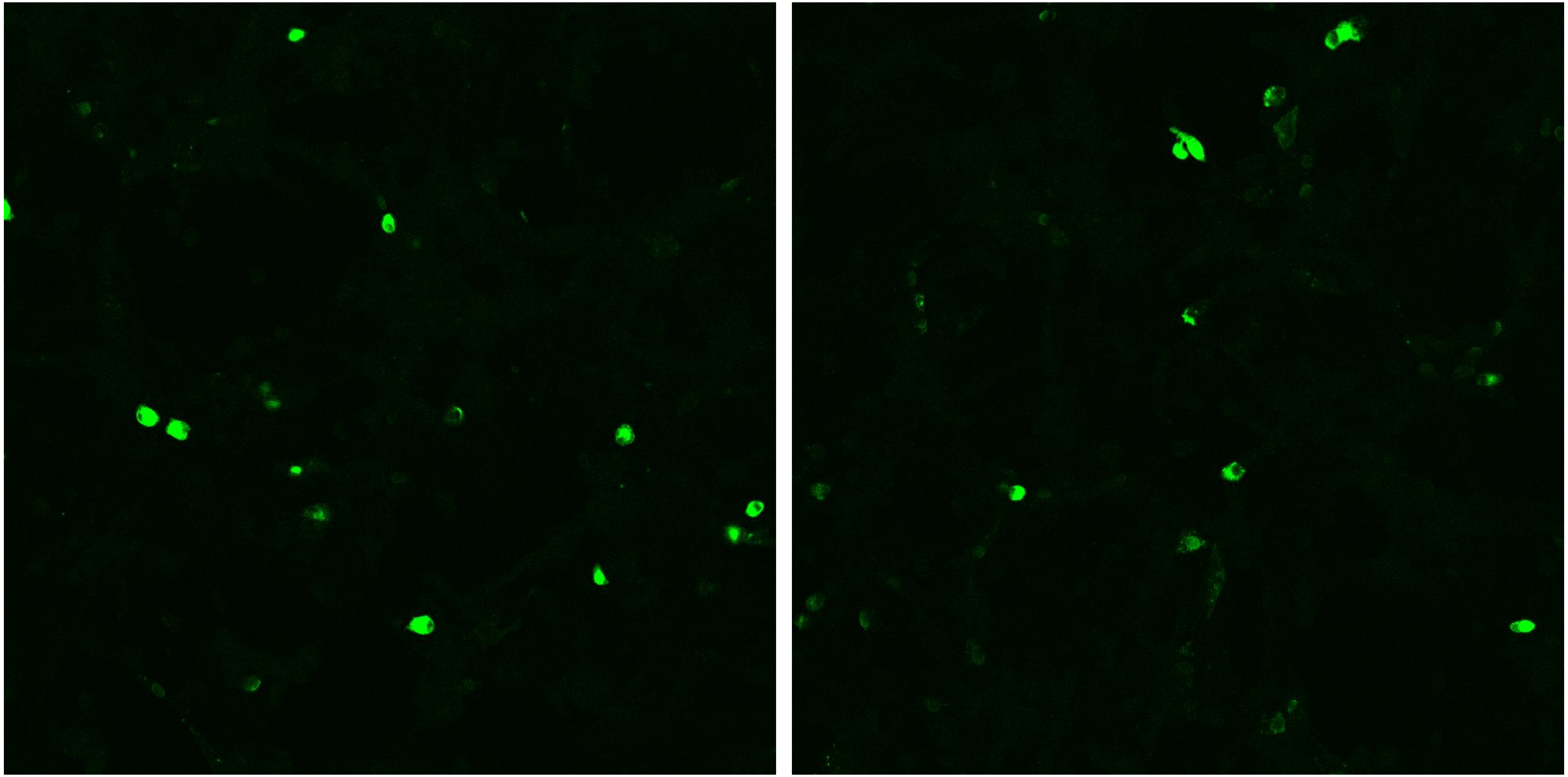

P23H + 5 μM 9-*cis*-retinal

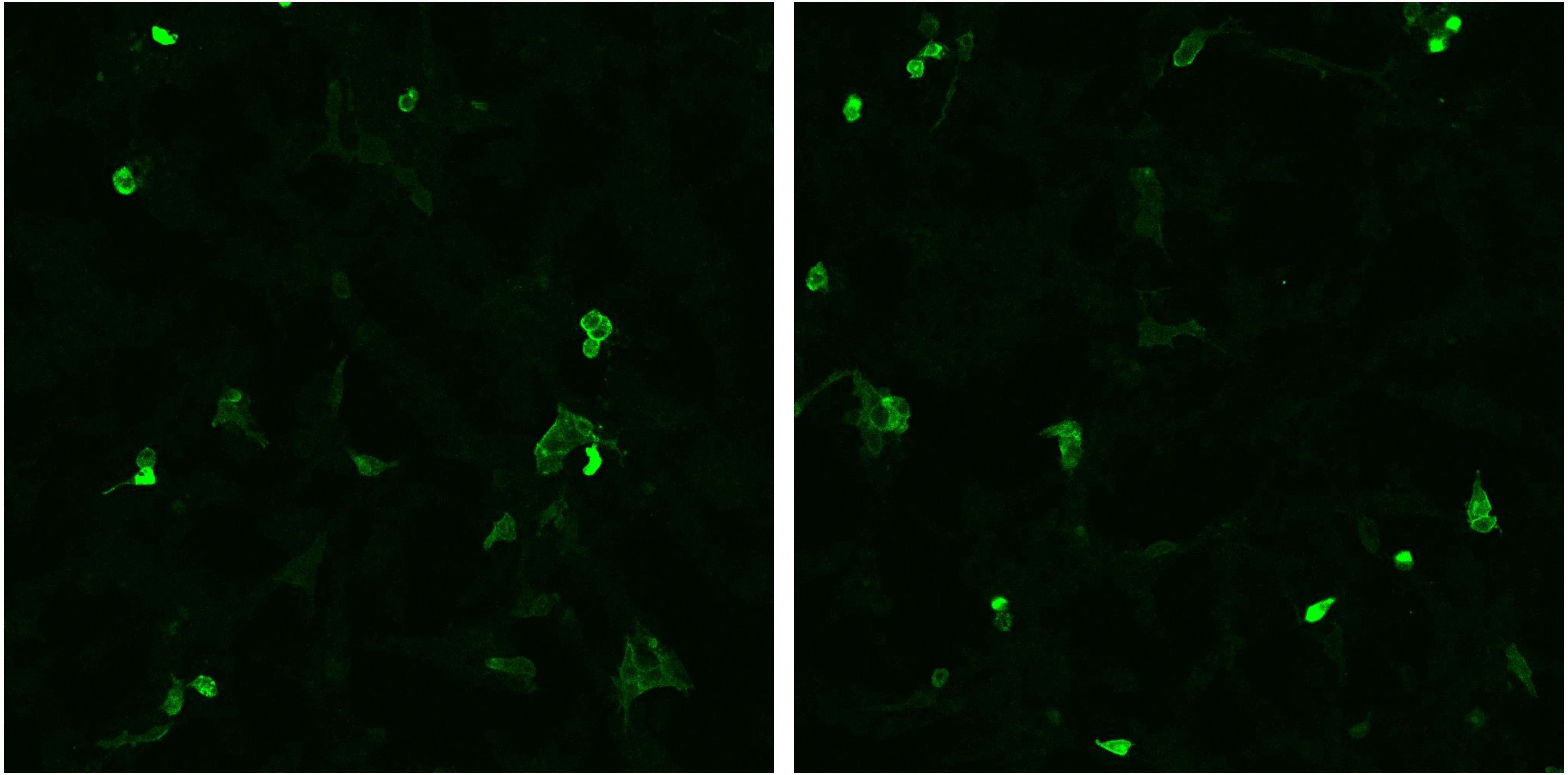

P23H + 40 μM YC-001

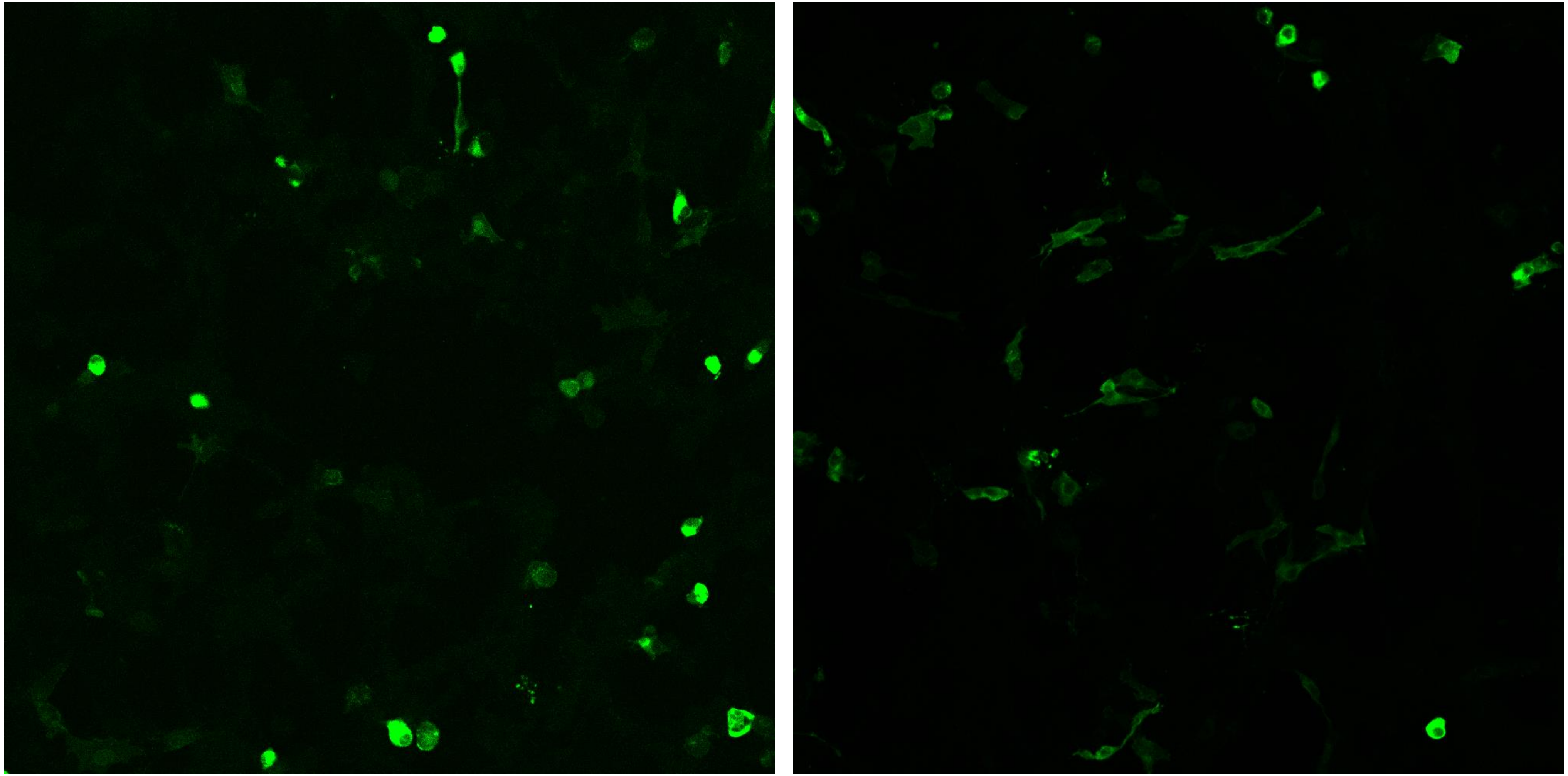

P23H + 20 μM F5257-0462

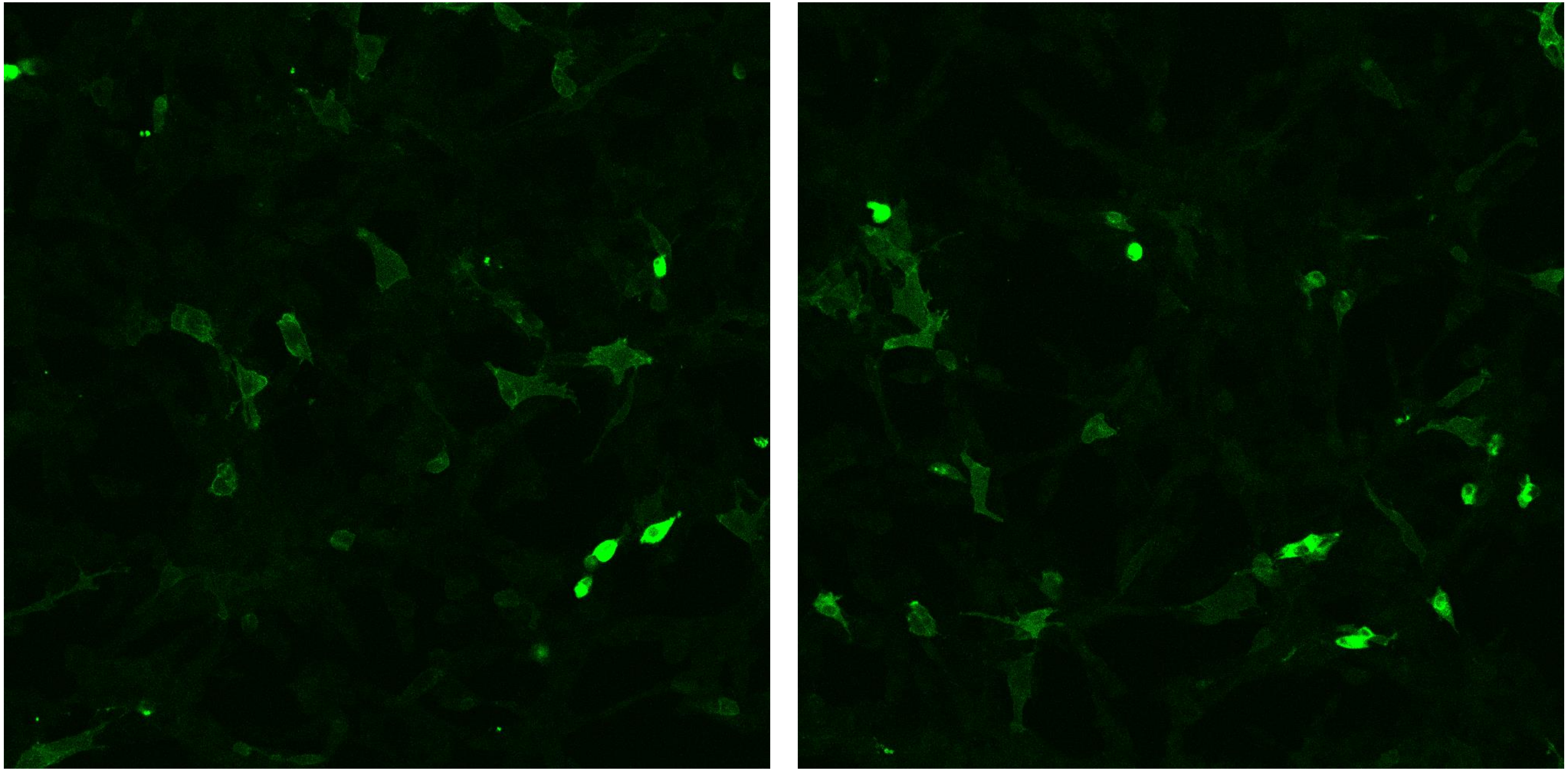

P23L + DMSO (0.1%)

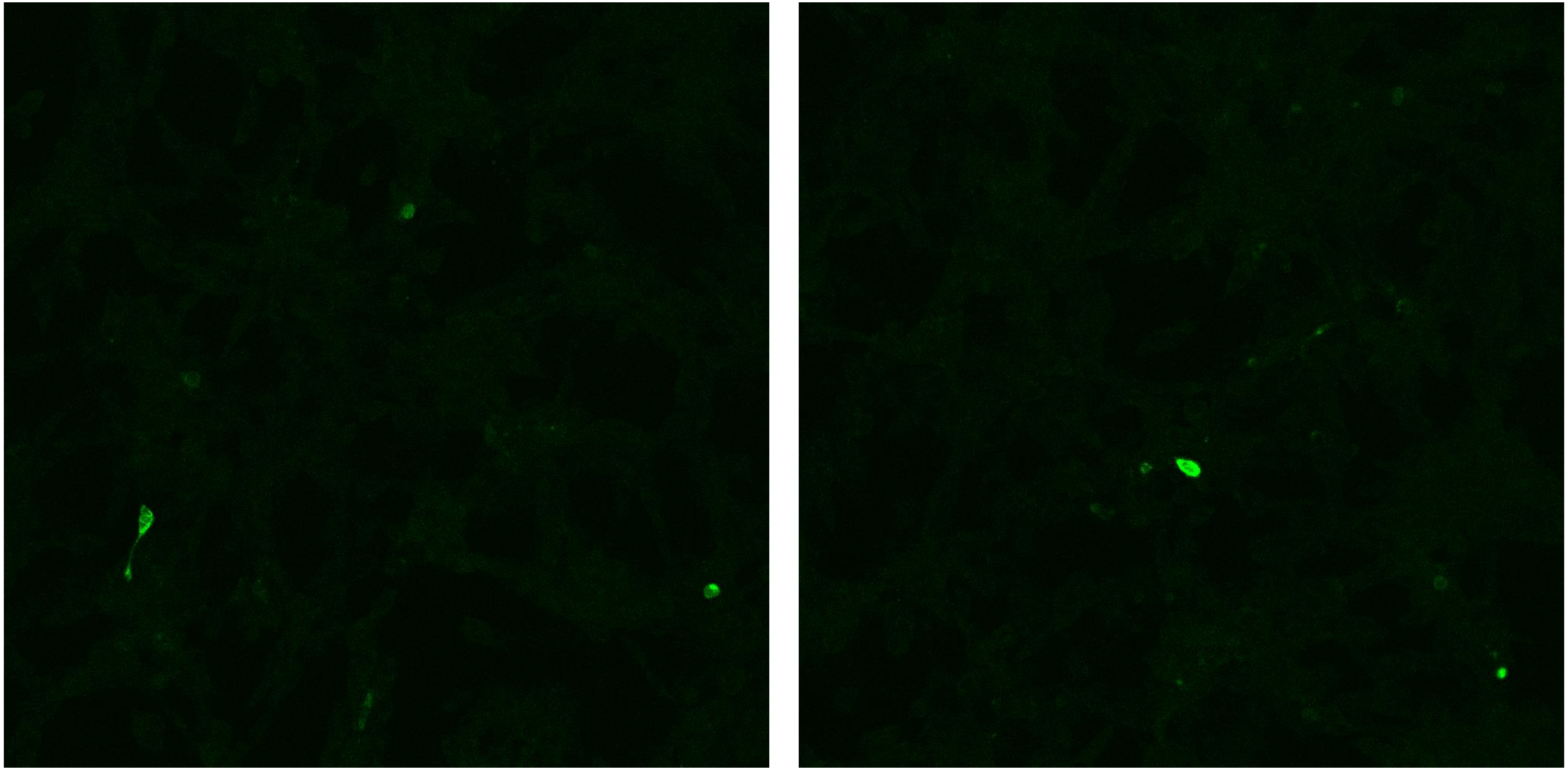

P23L + DMSO (0.1%)

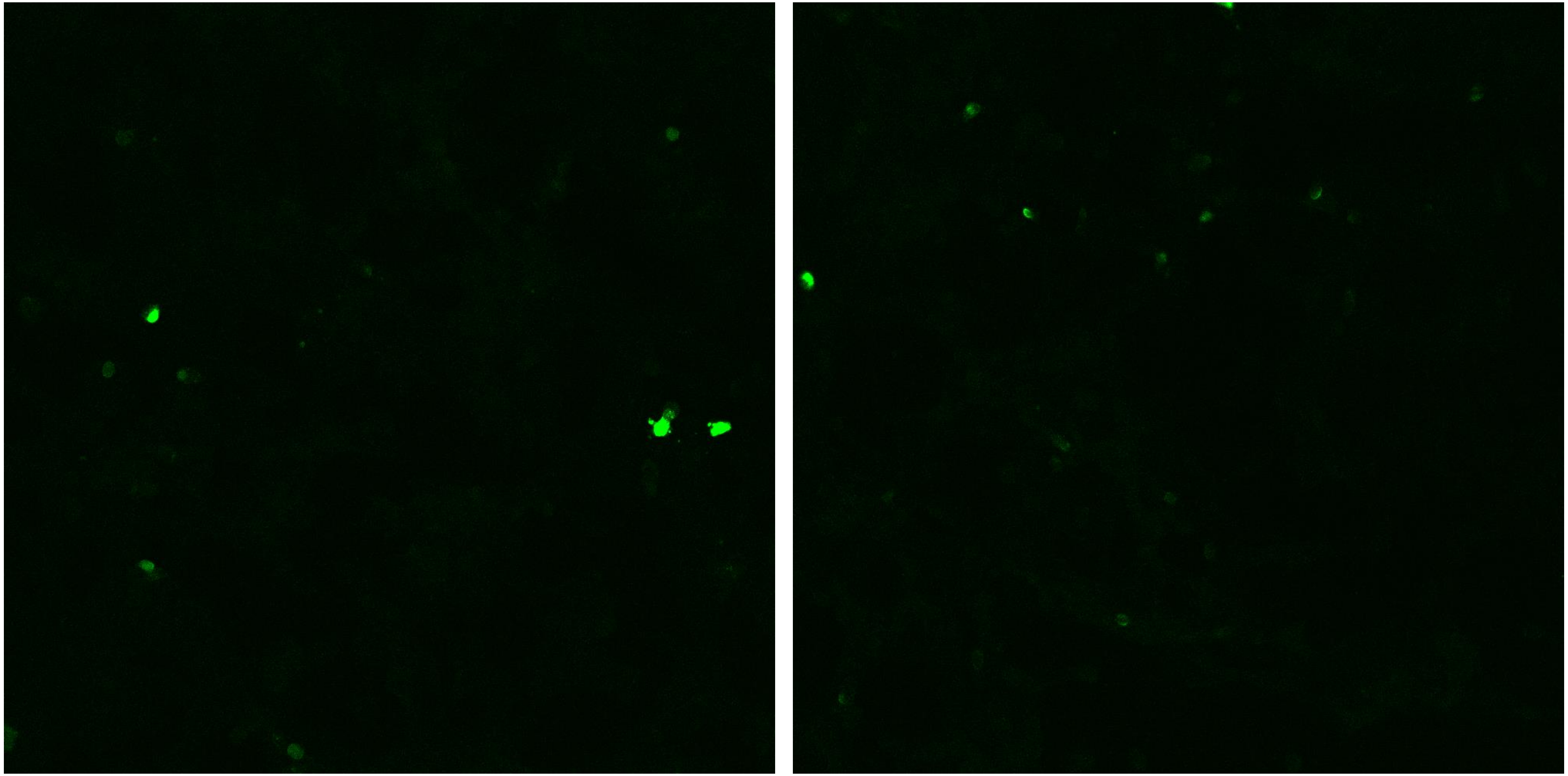

P23L + 5 μM 9-*cis*-retinal

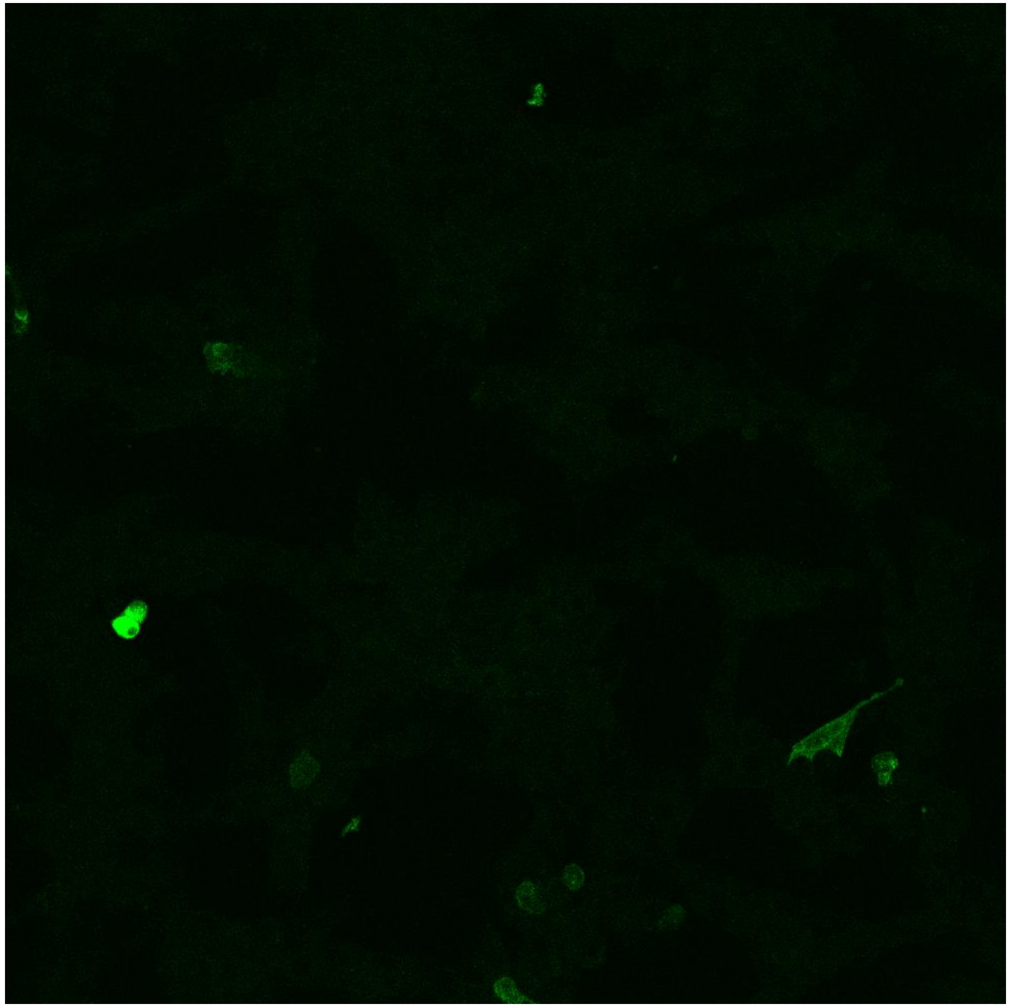

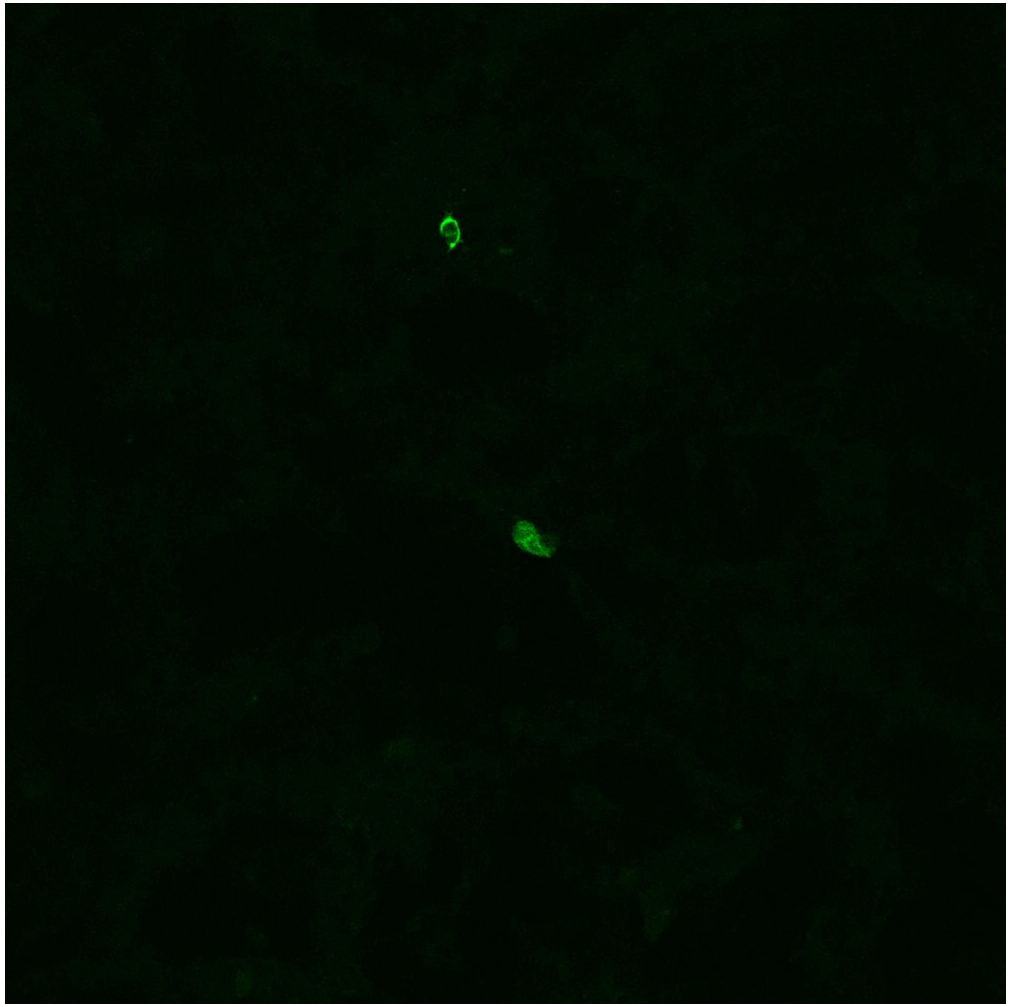

P23L + 40 µM YC-001

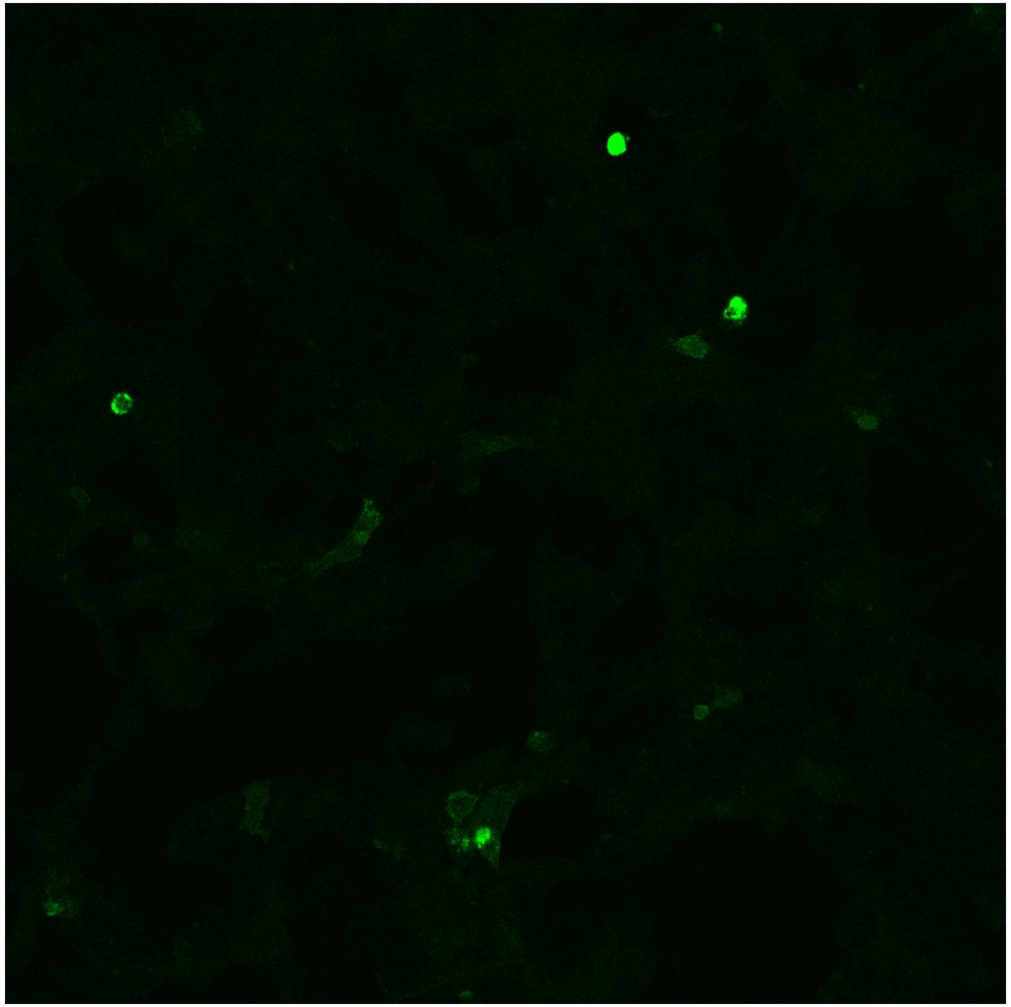

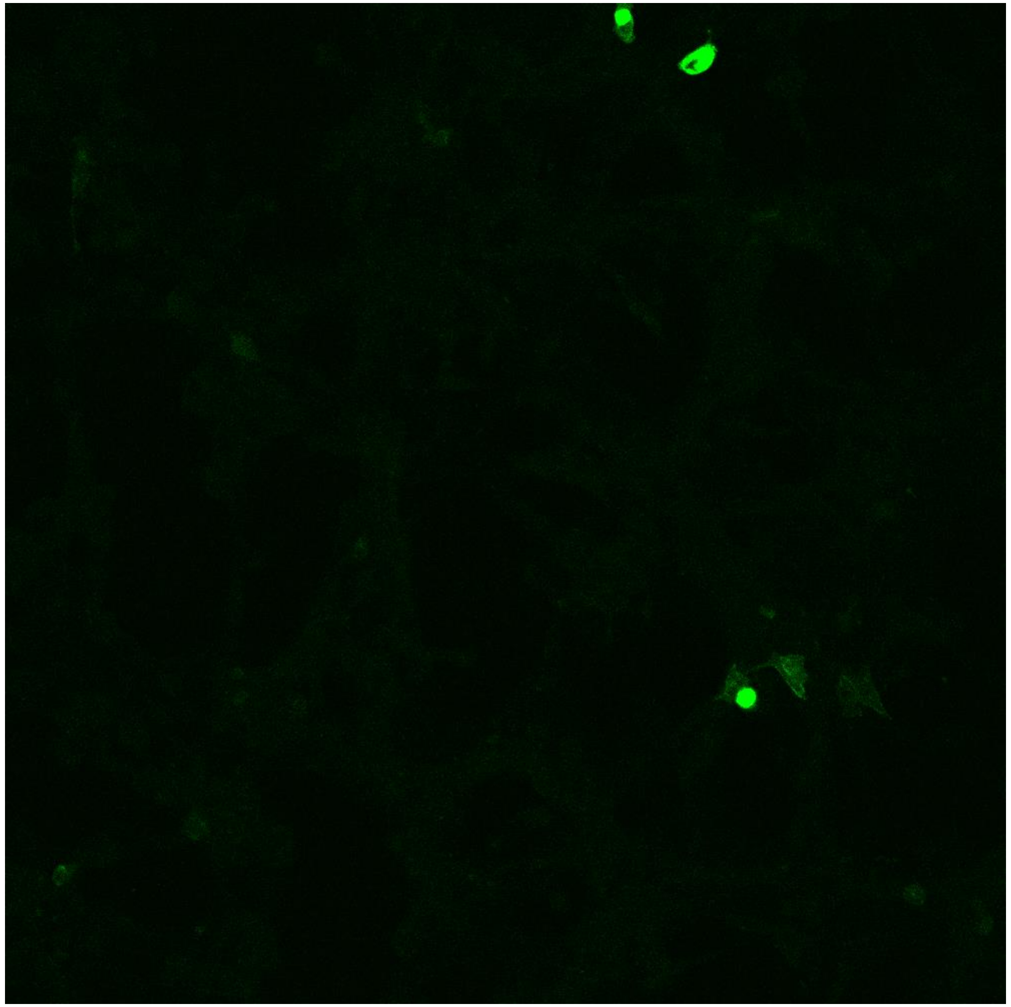

P23L + 20 µM F5257-0462

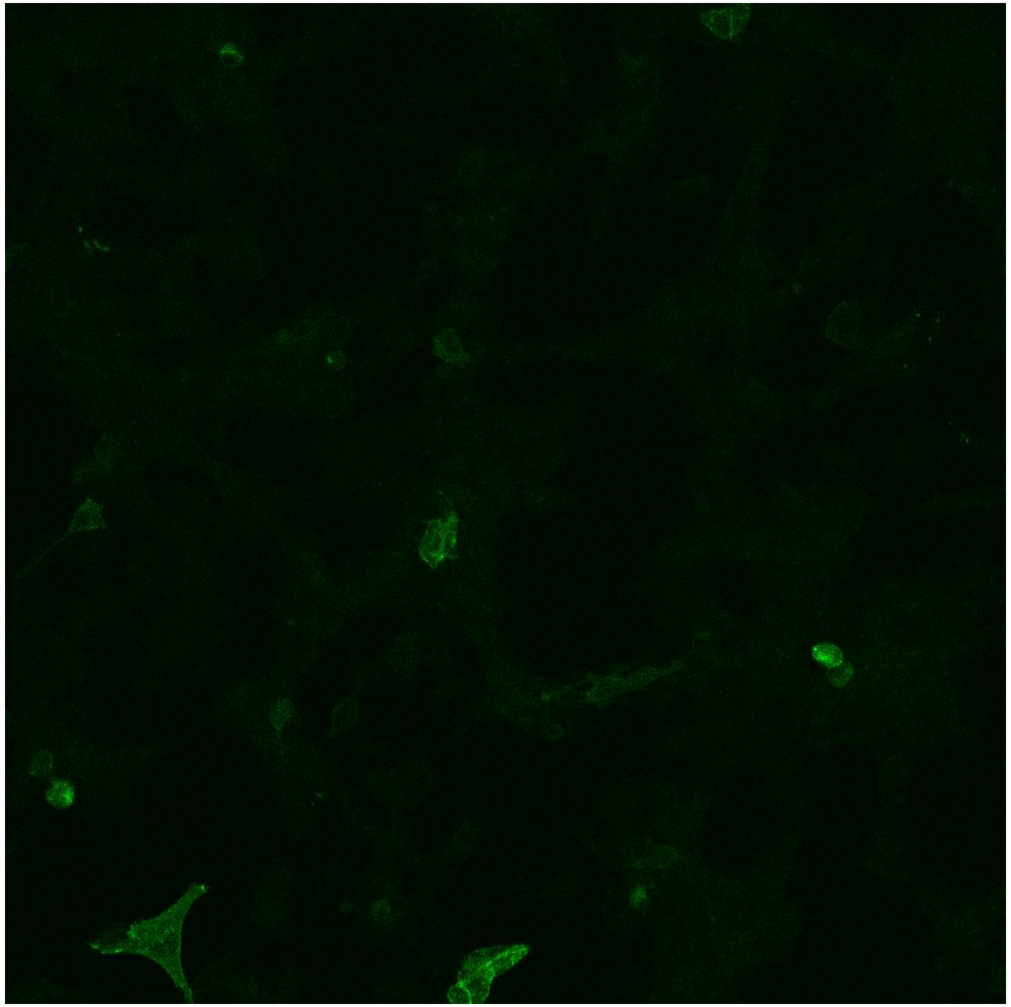

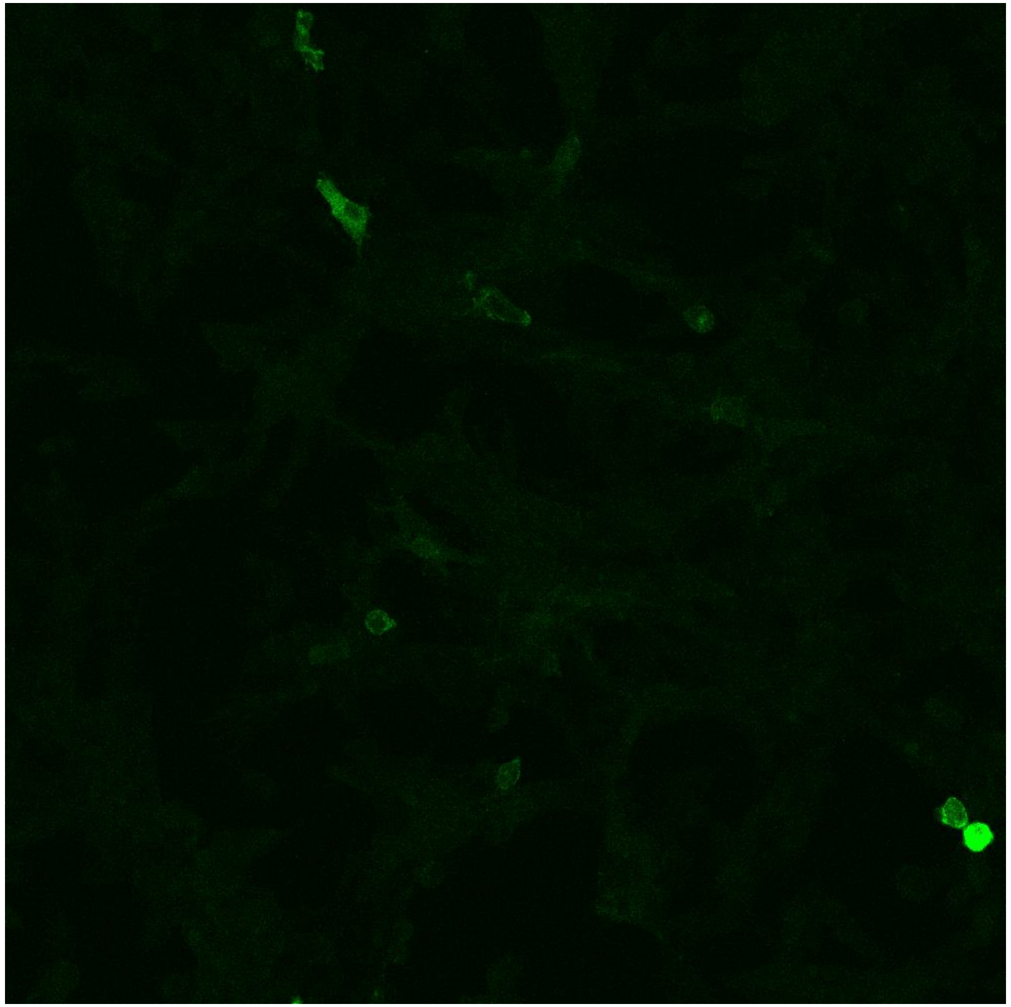

Q28H + DMSO (0.1%)

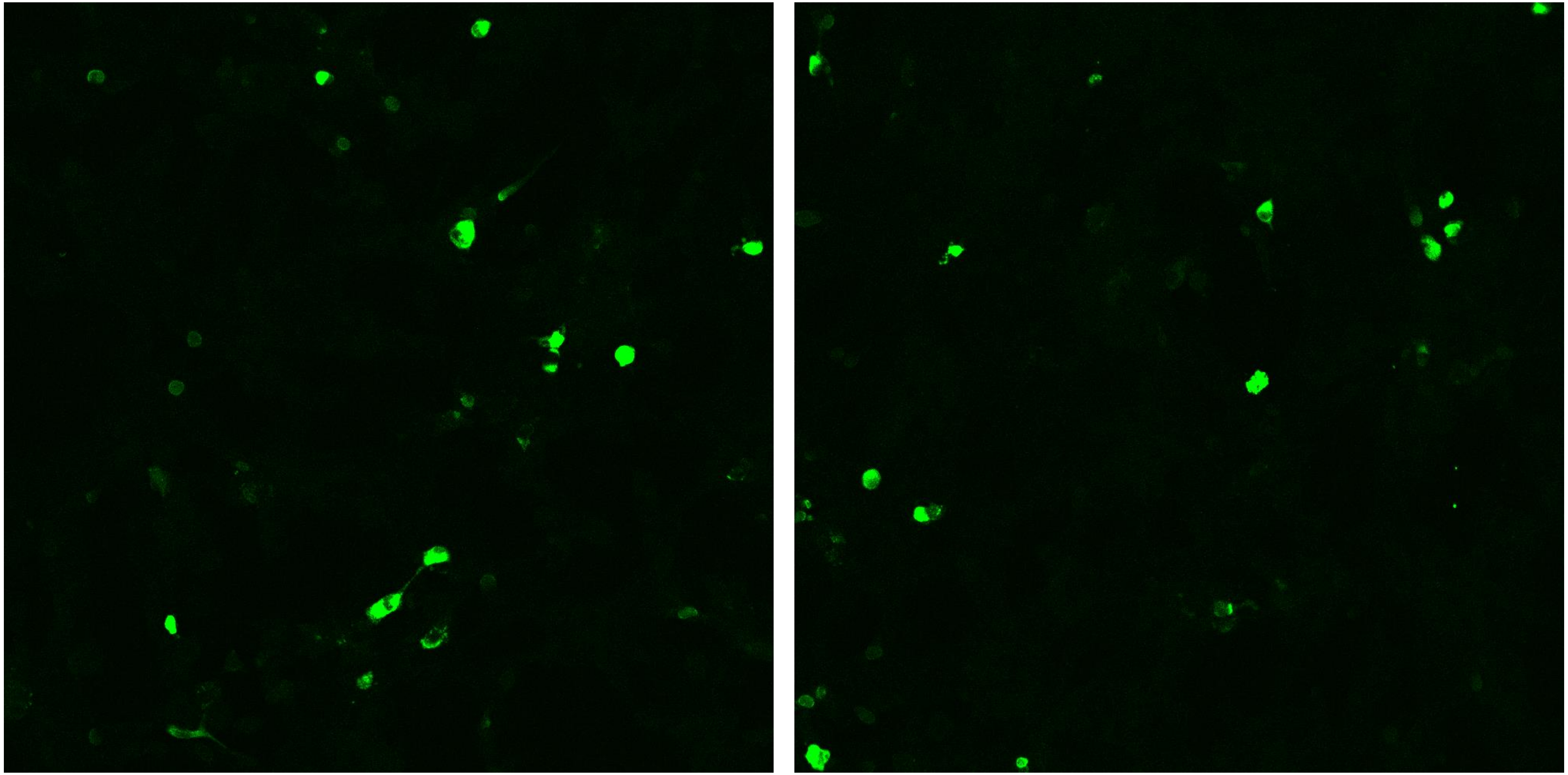

Q28H + DMSO (0.1%)

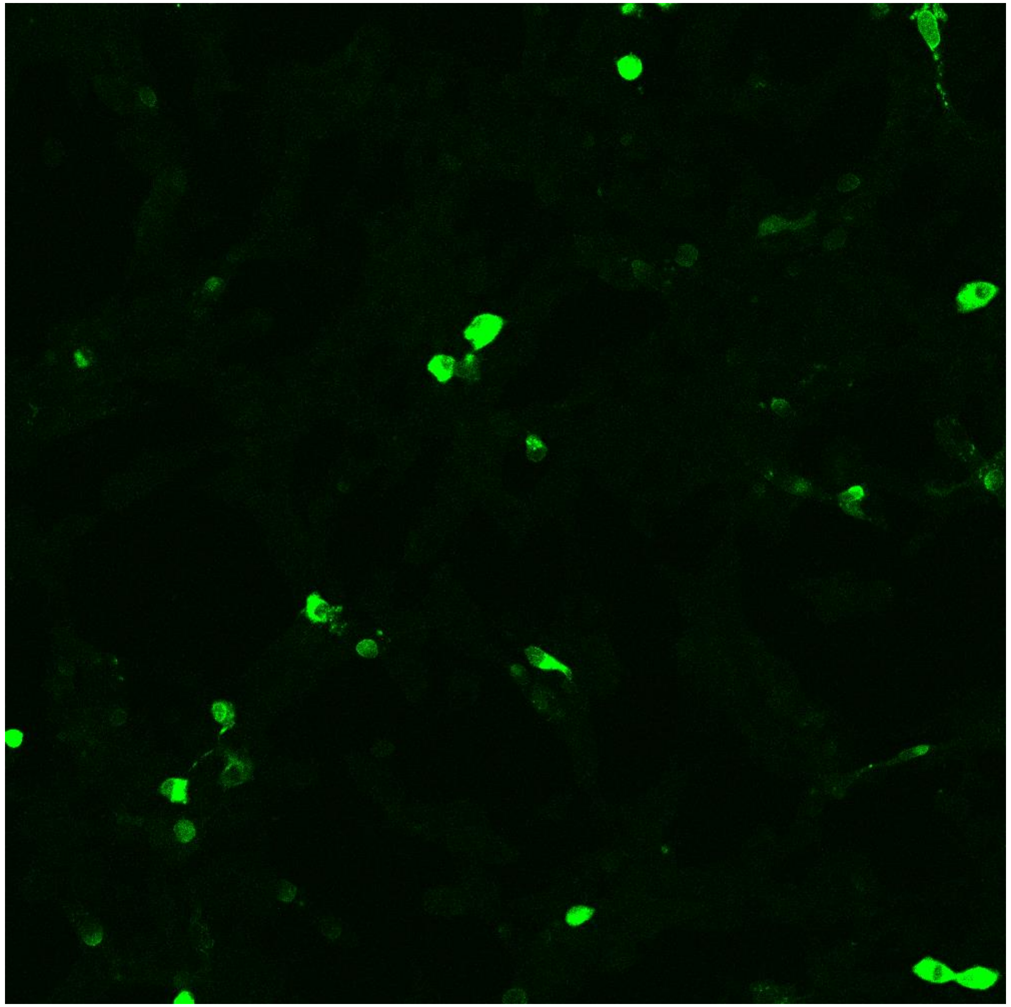

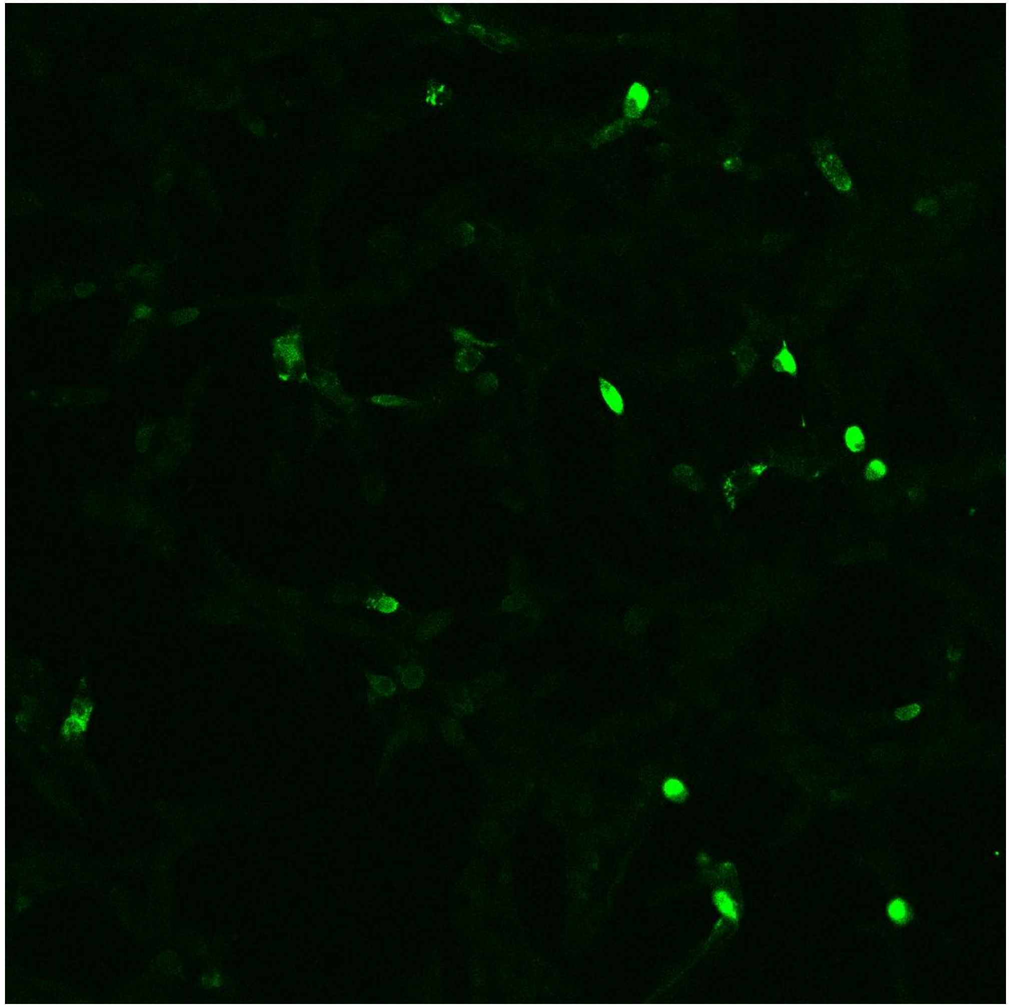

Q28H + 5 μM 9-*cis*-retinal

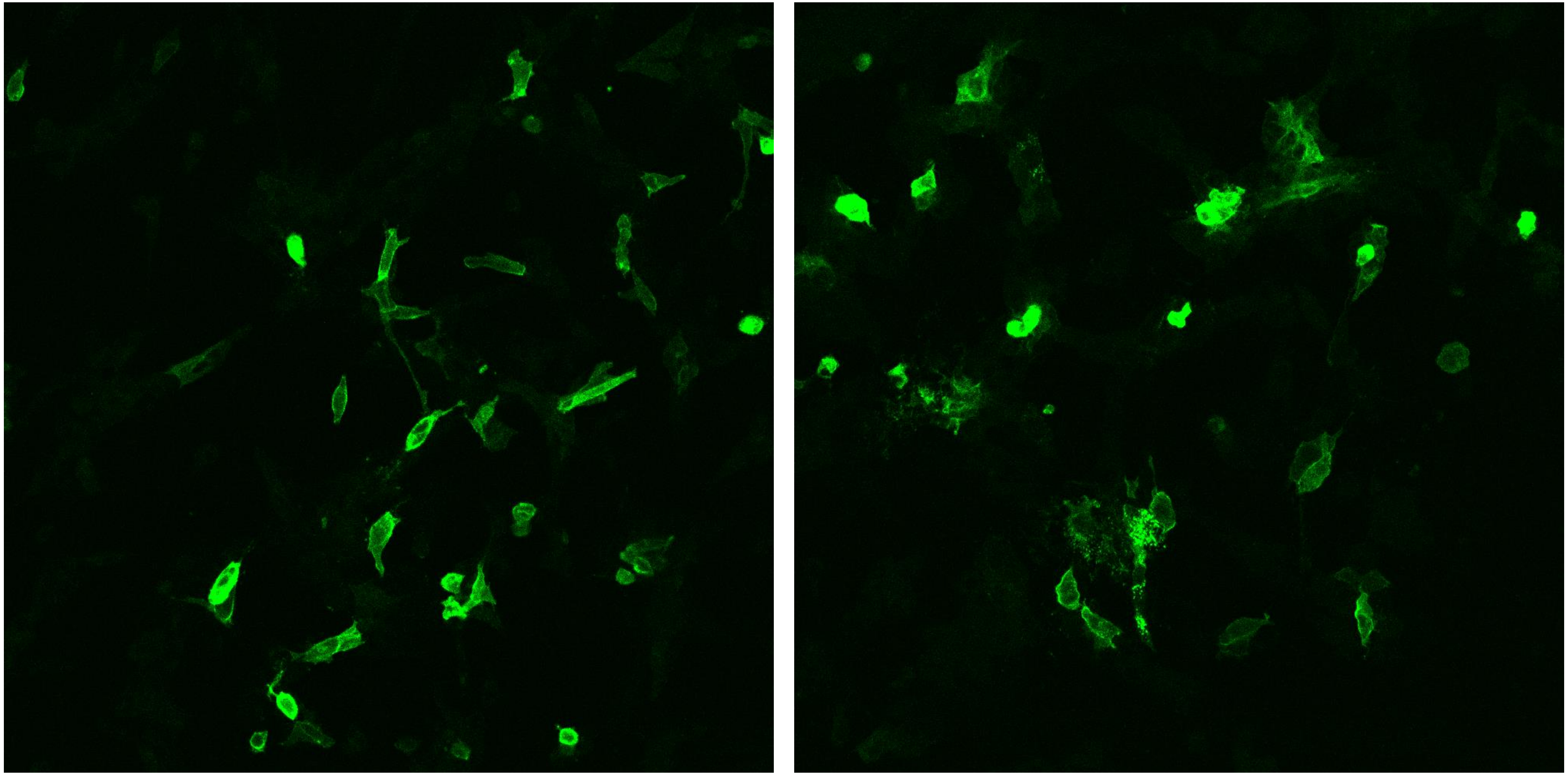

Q28H + 40 μM YC-001

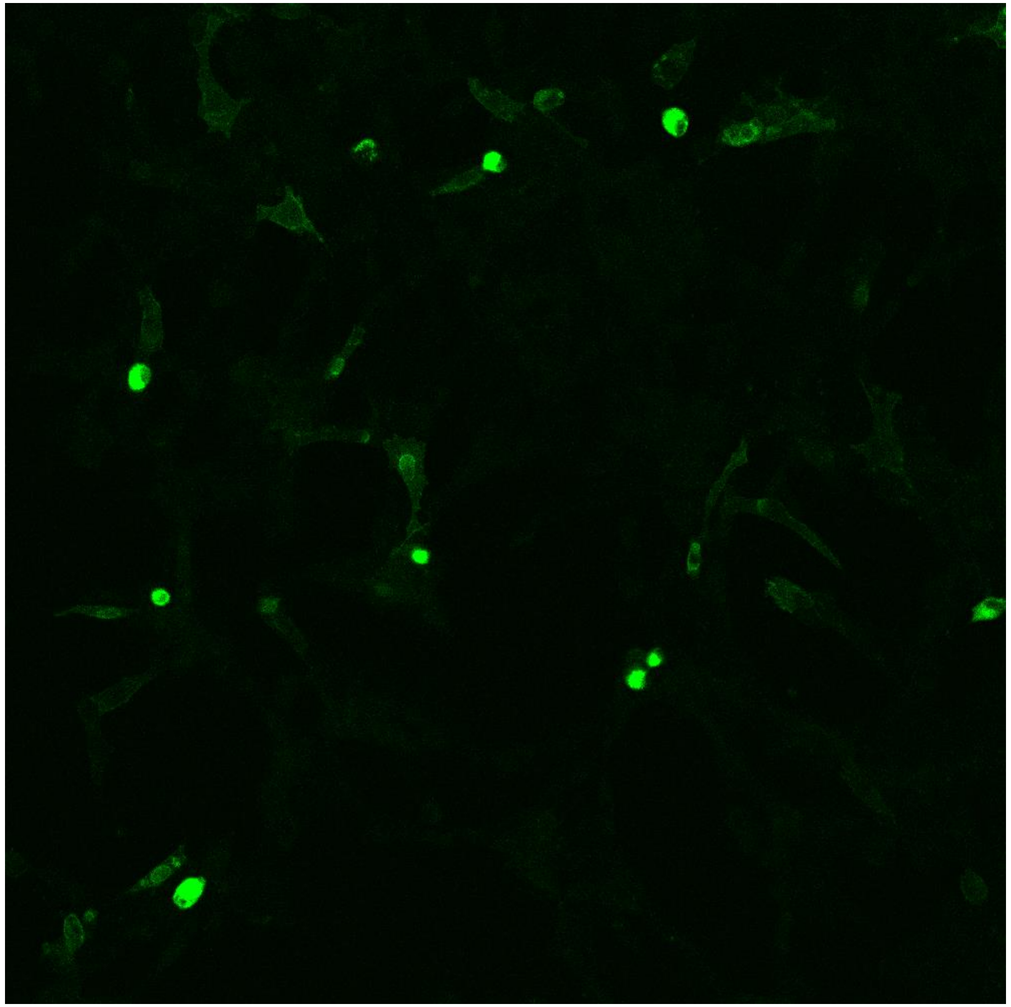

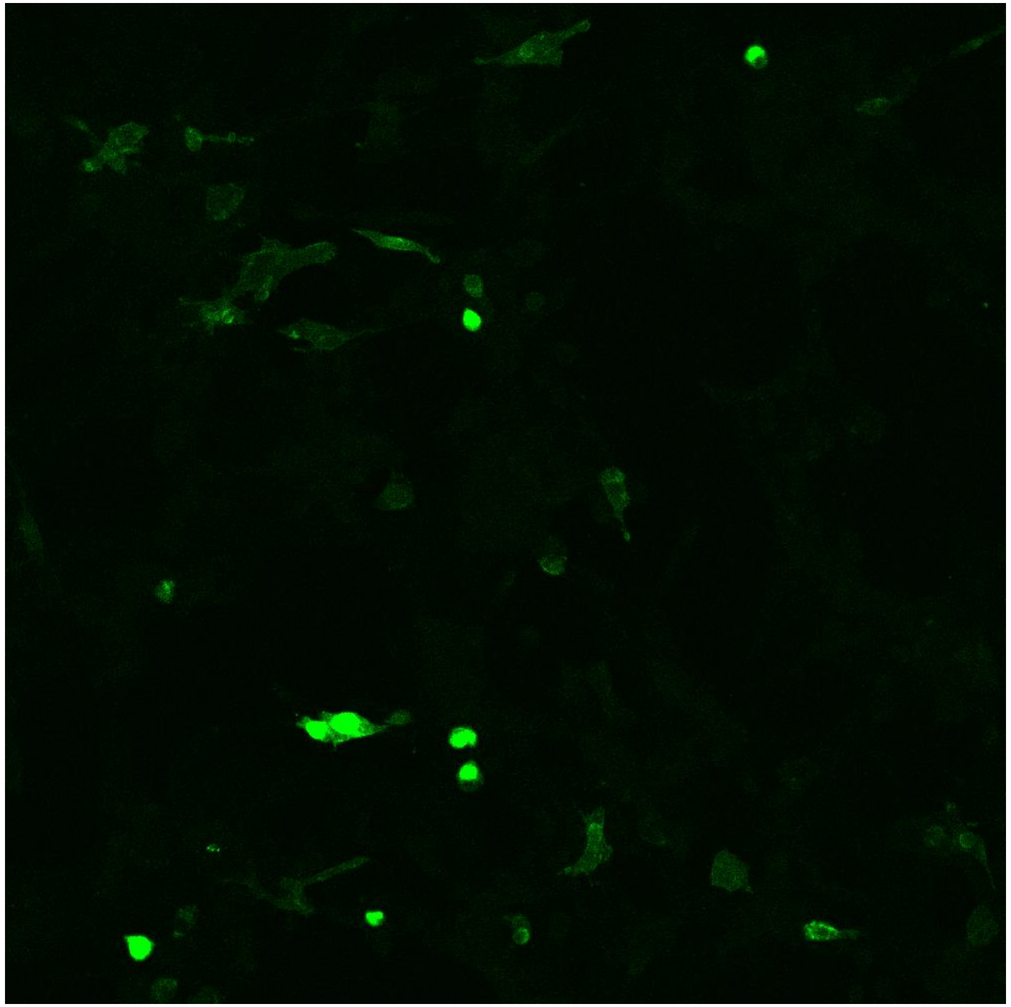

Q28H + 20 μM F5257-0462

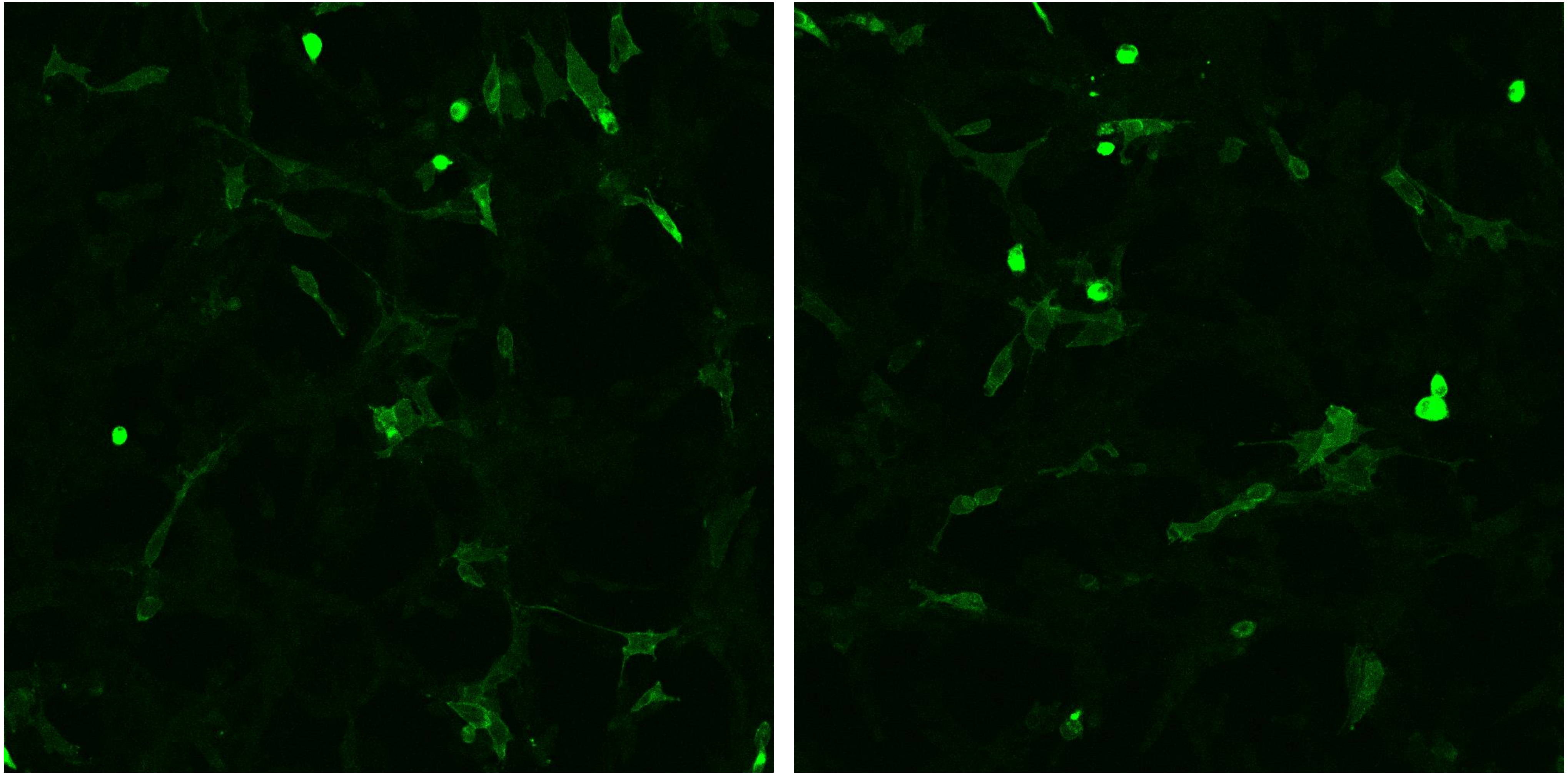

P53R + DMSO (0.1%)

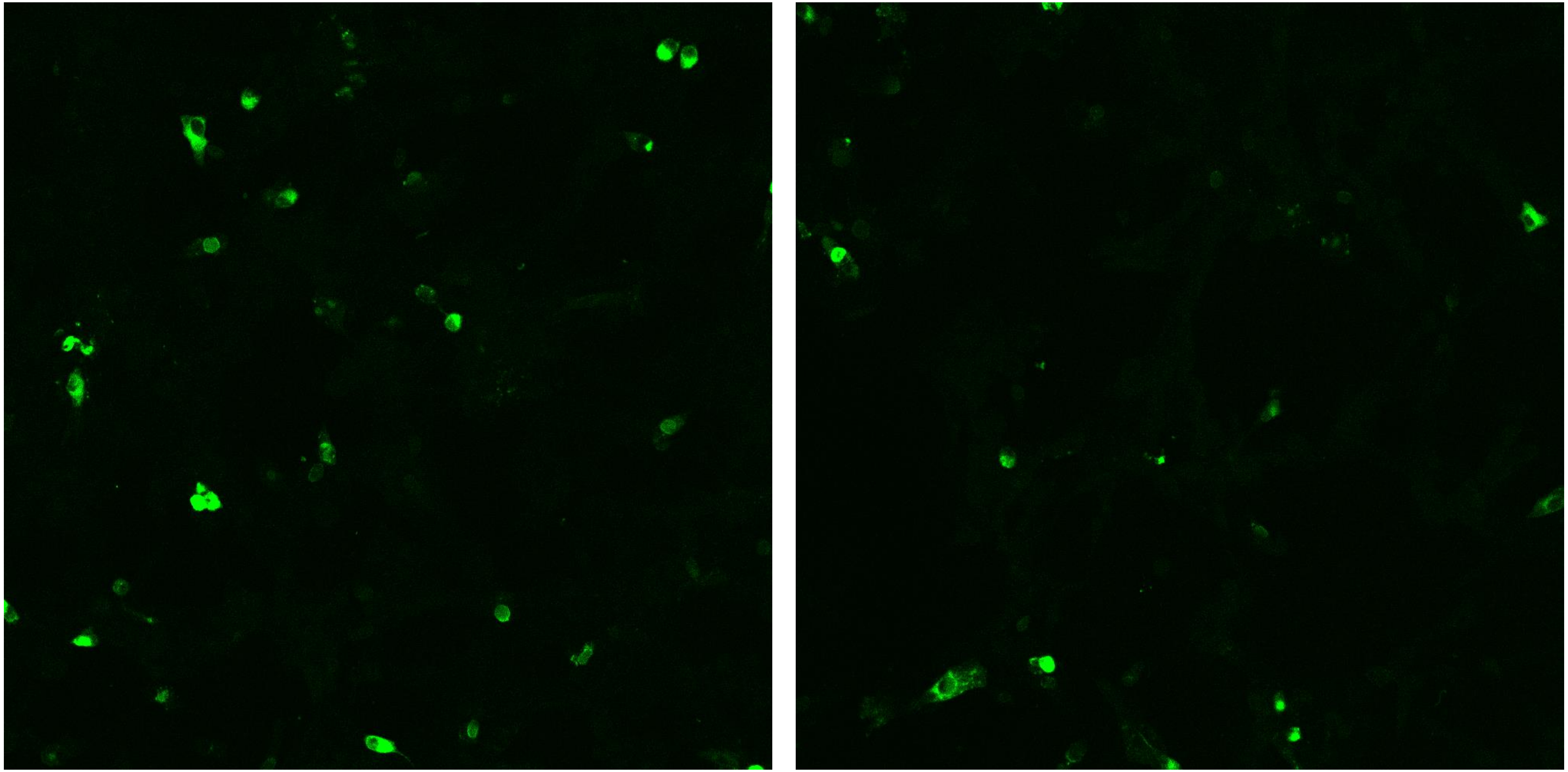

P53R + DMSO (0.1%)

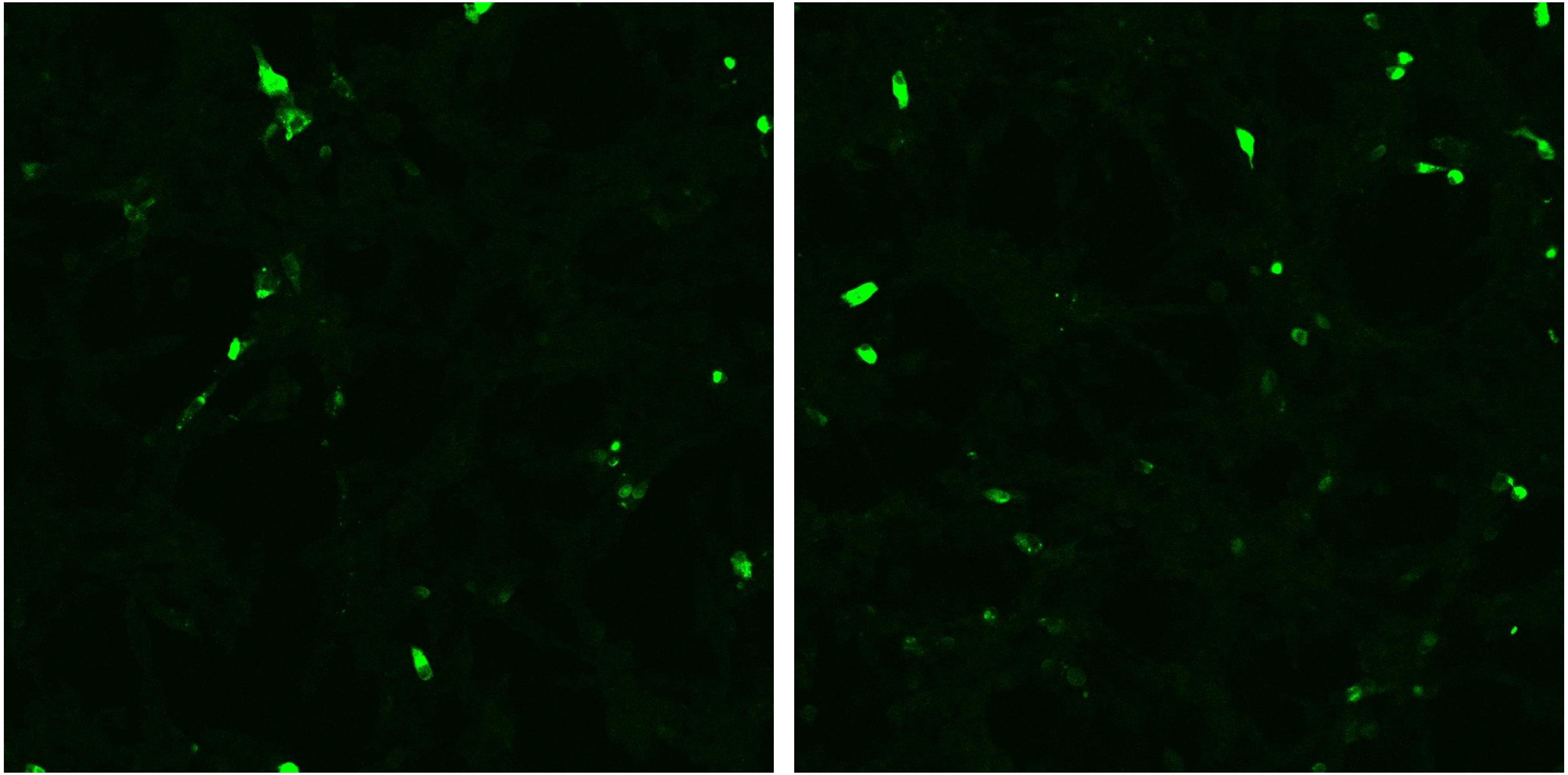

P53R + 5 µM 9-*cis*-retinal

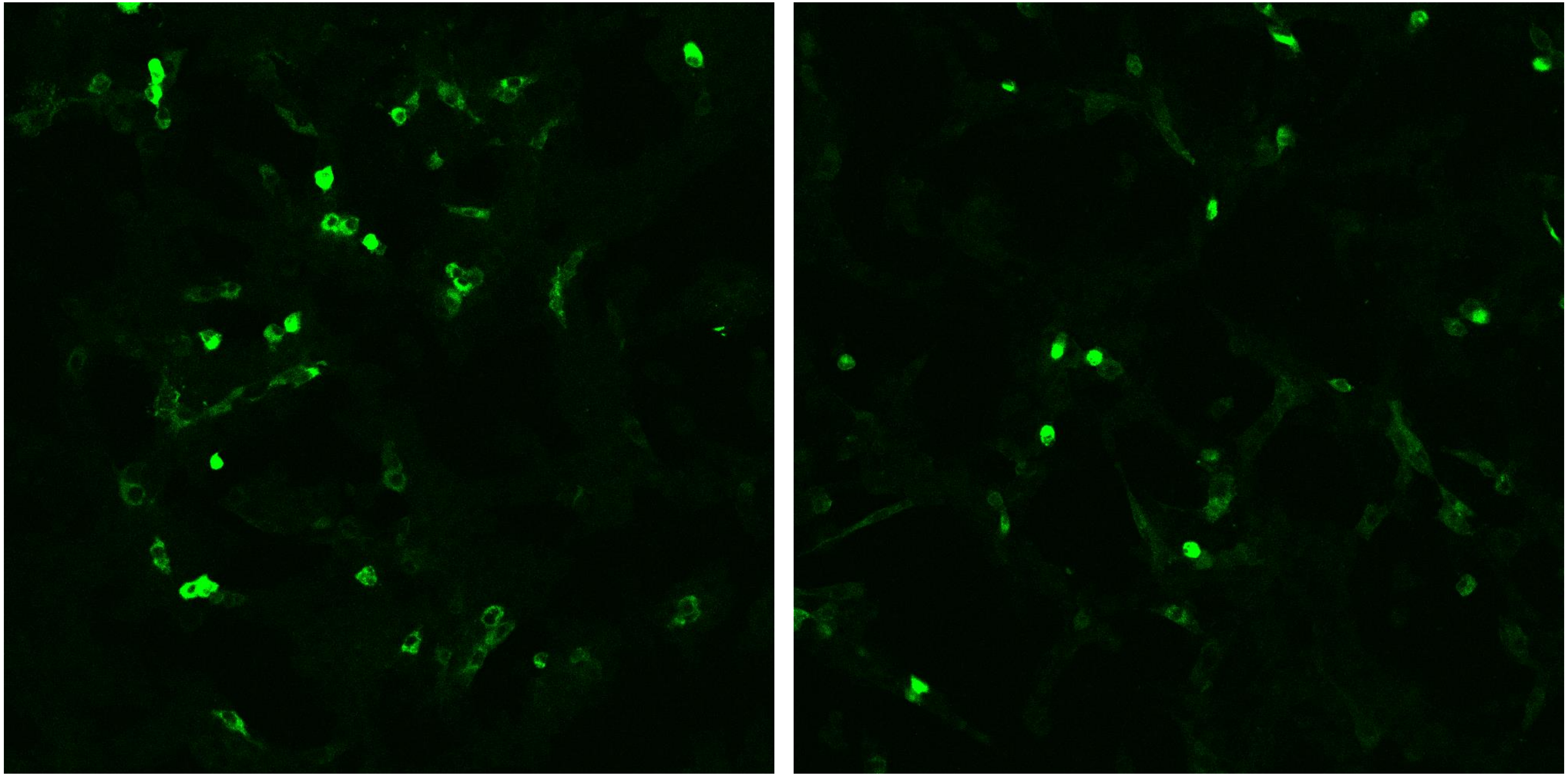

P53R + 40 μM YC-001

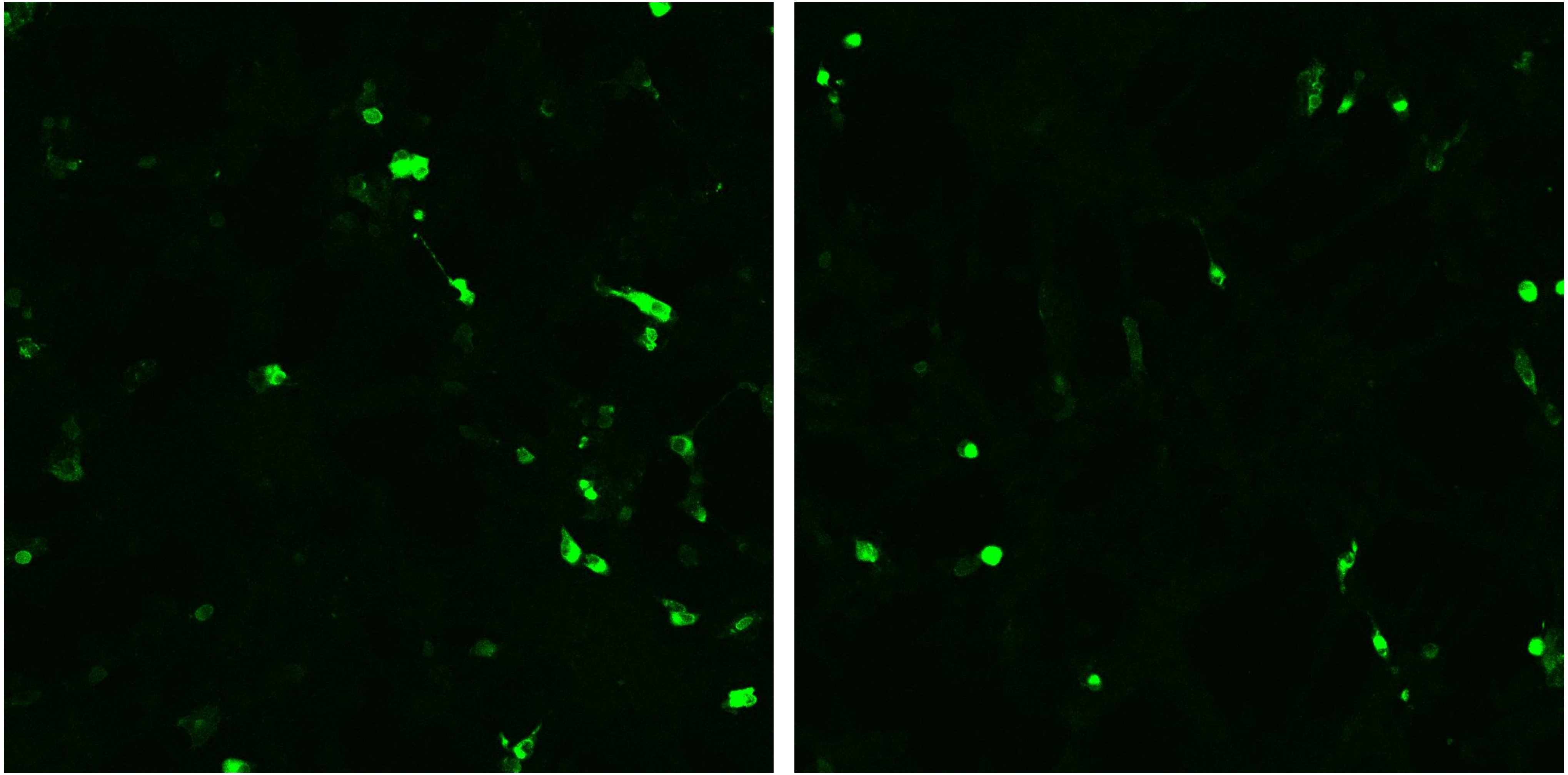

P53R + 20 μM F5257-0462

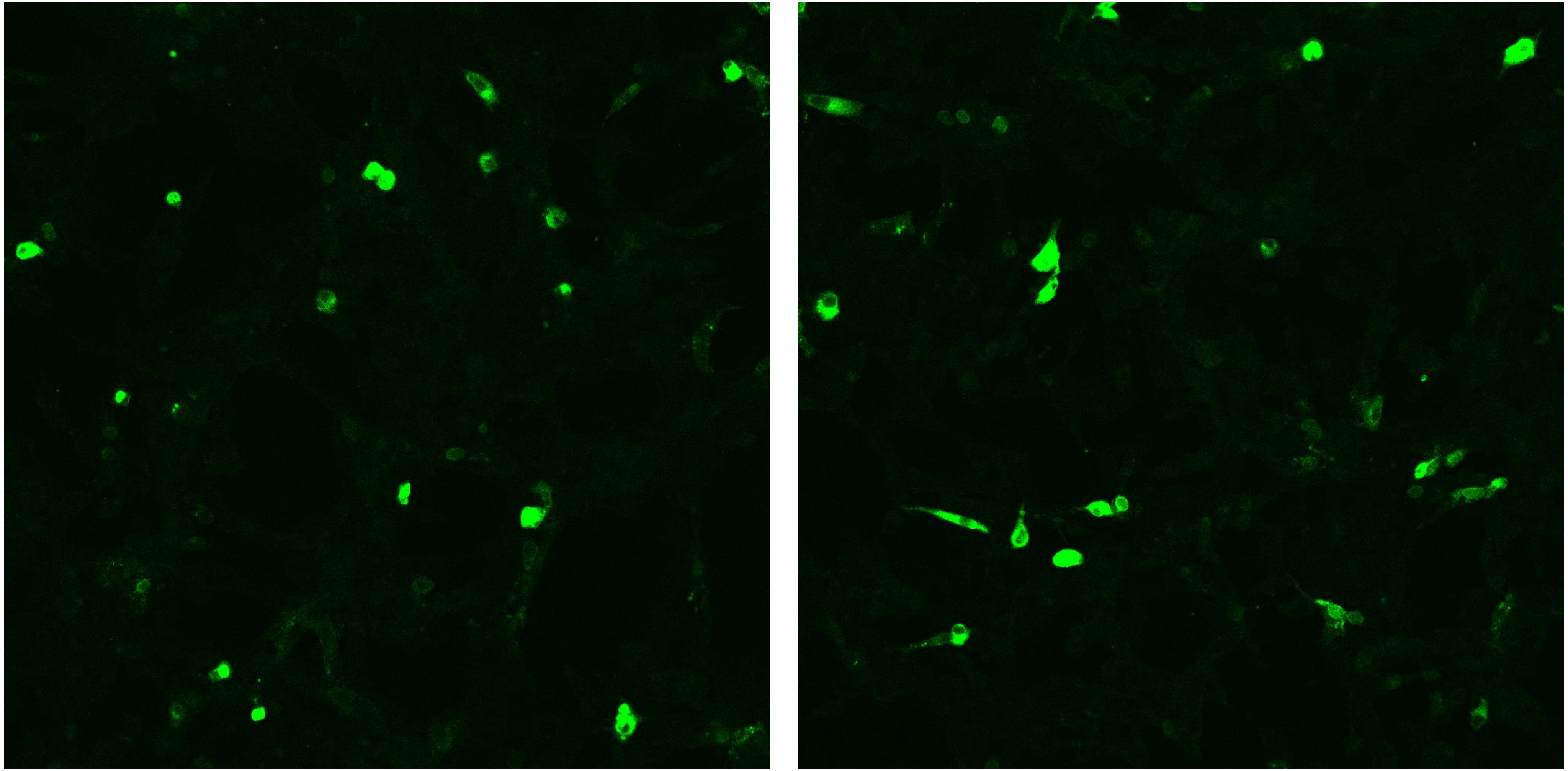

G106R + DMSO (0.1%)

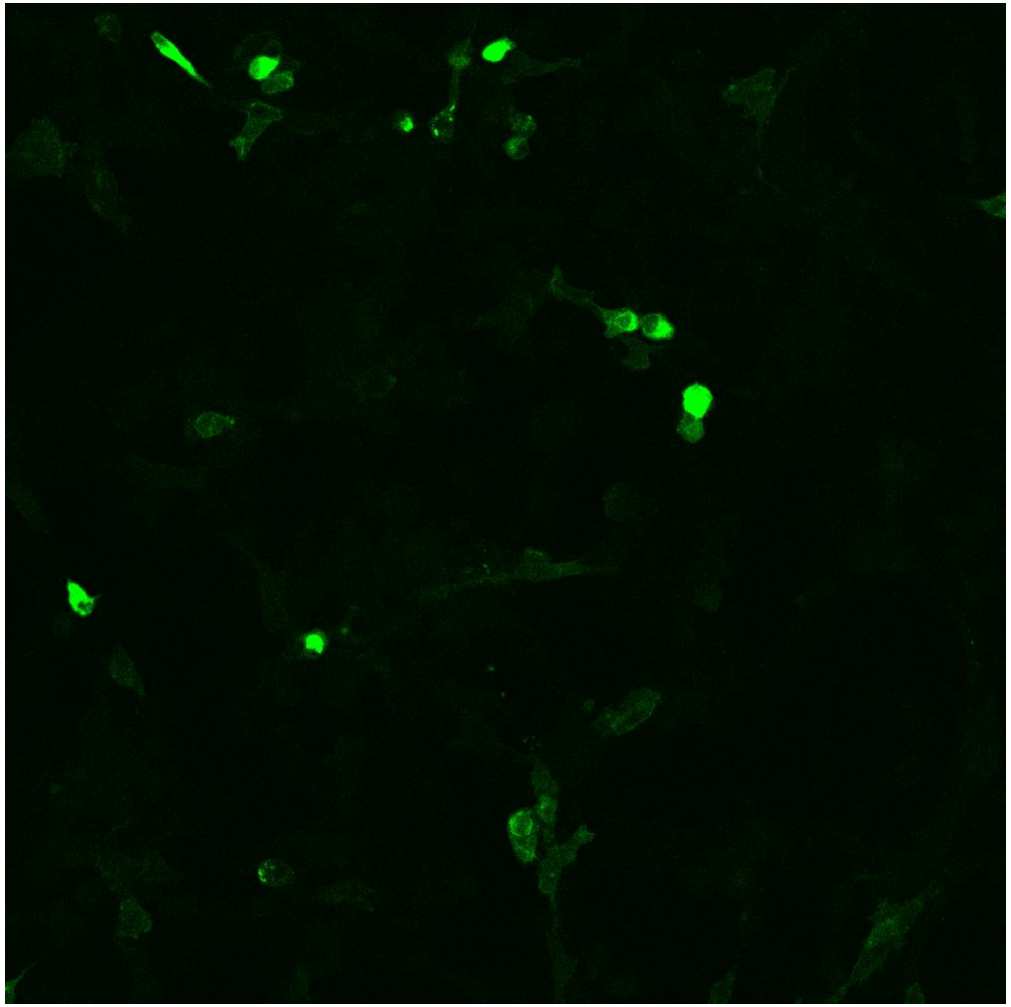

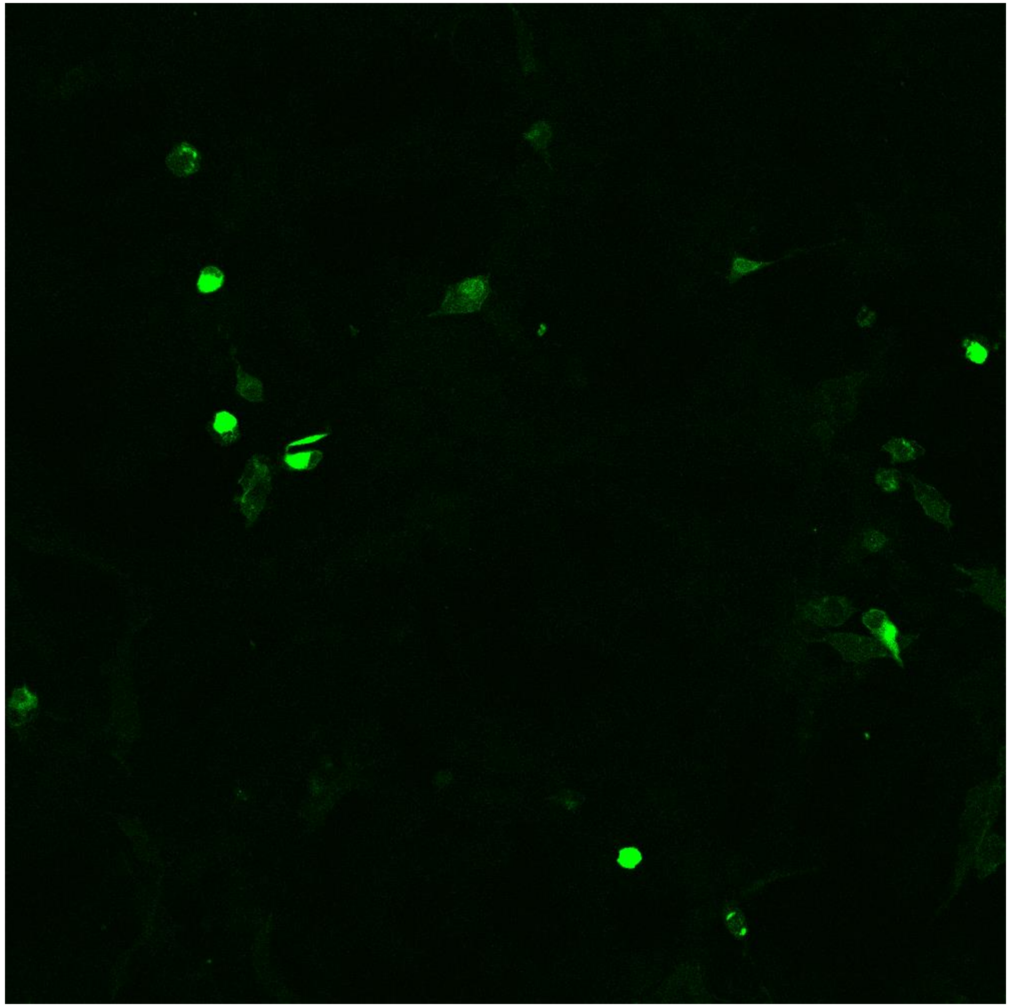

G106R + DMSO (0.1%)

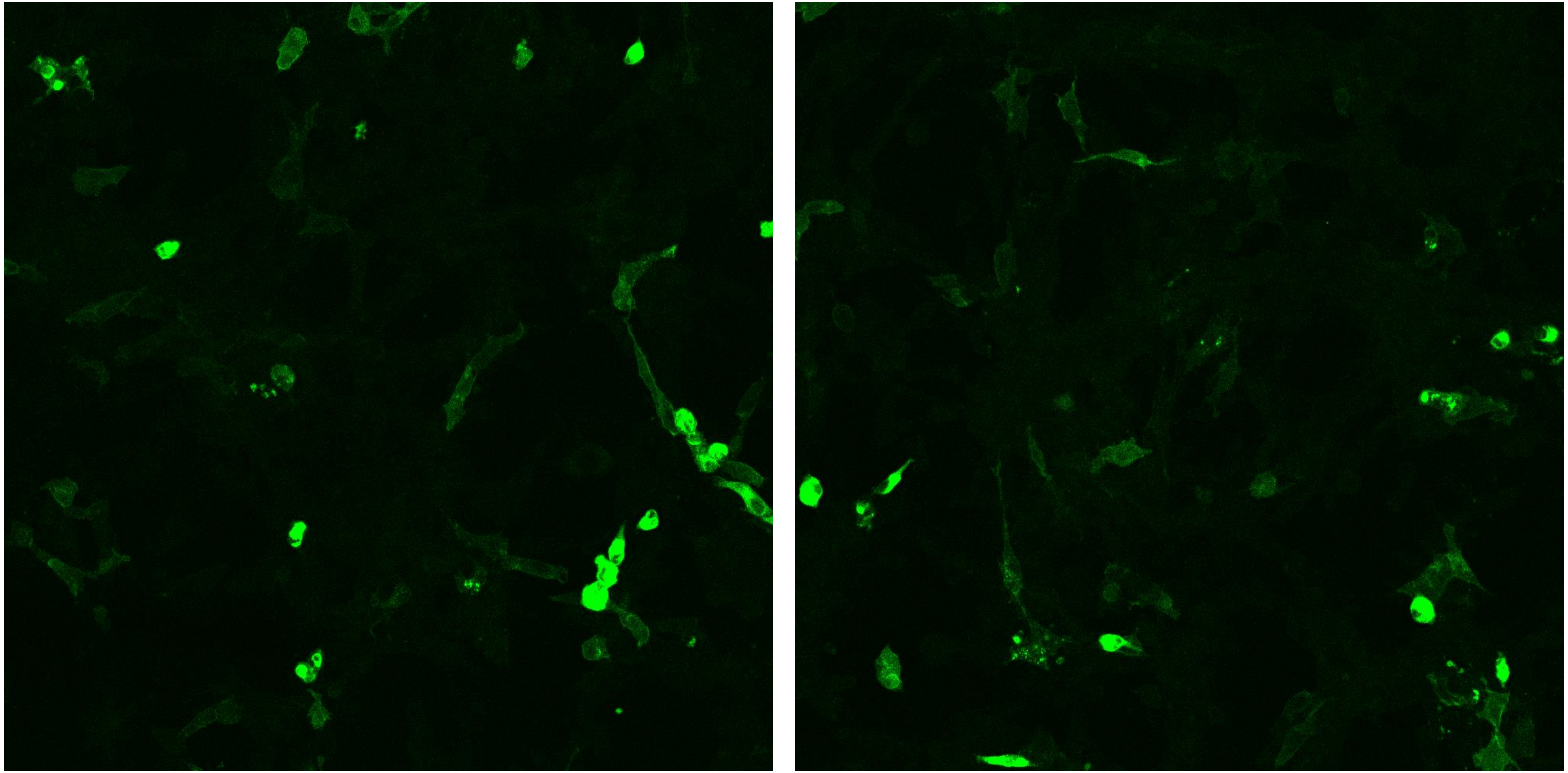

G106R + 5 μM 9-*cis*-retinal

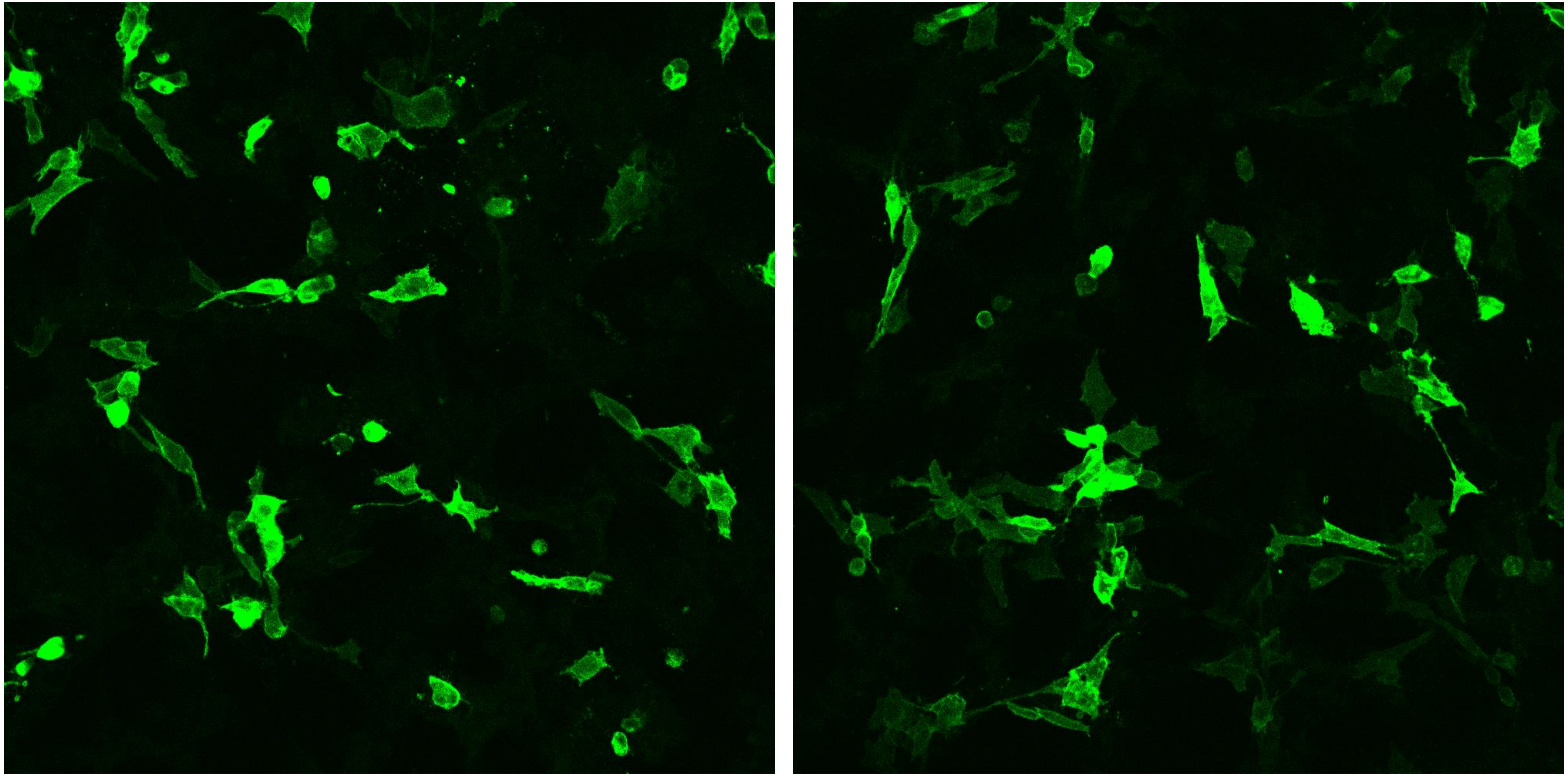

G106R + 40 μM YC-001

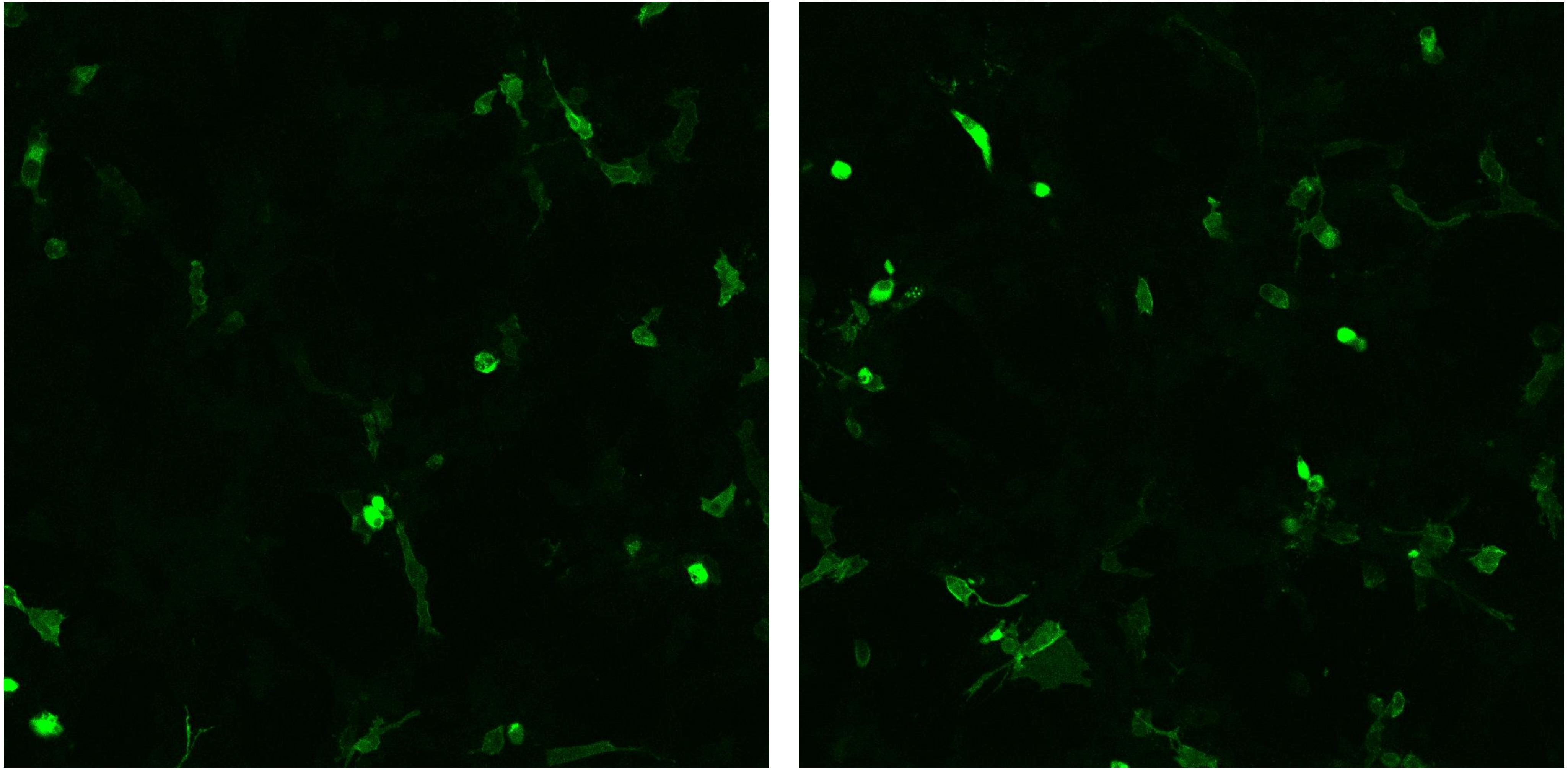

G106R + 20 μM F5257-0462

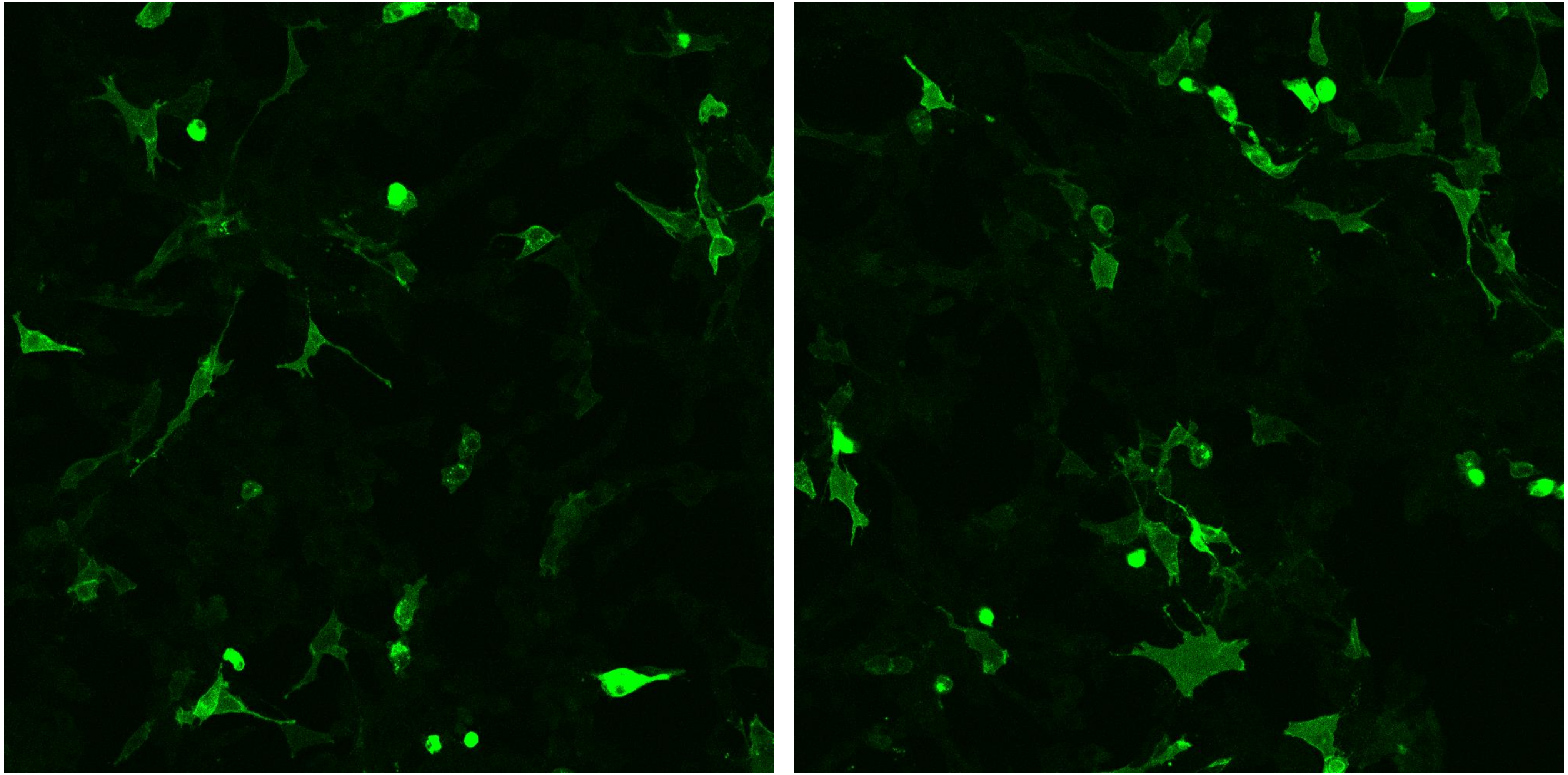

G106W + DMSO (0.1%)

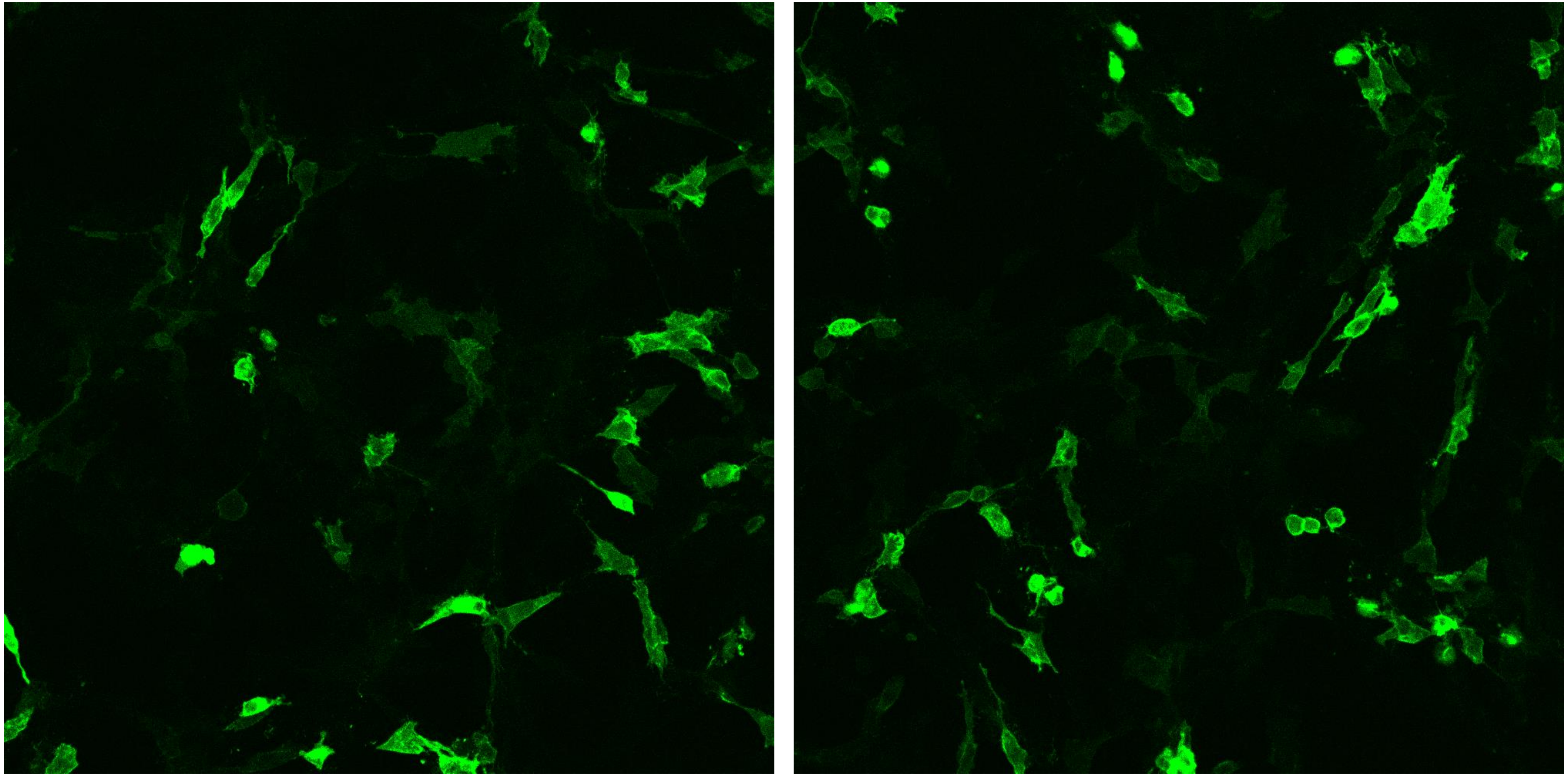

G106W + DMSO (0.1%)

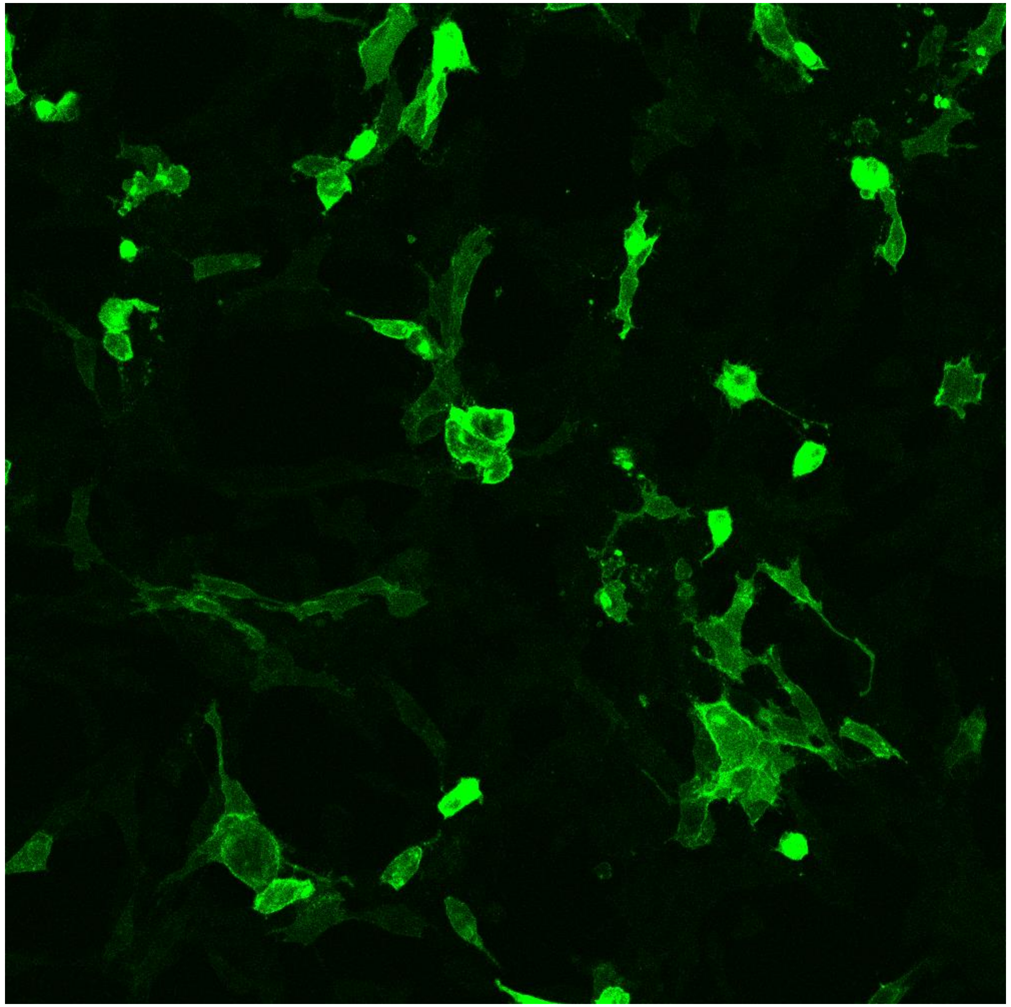

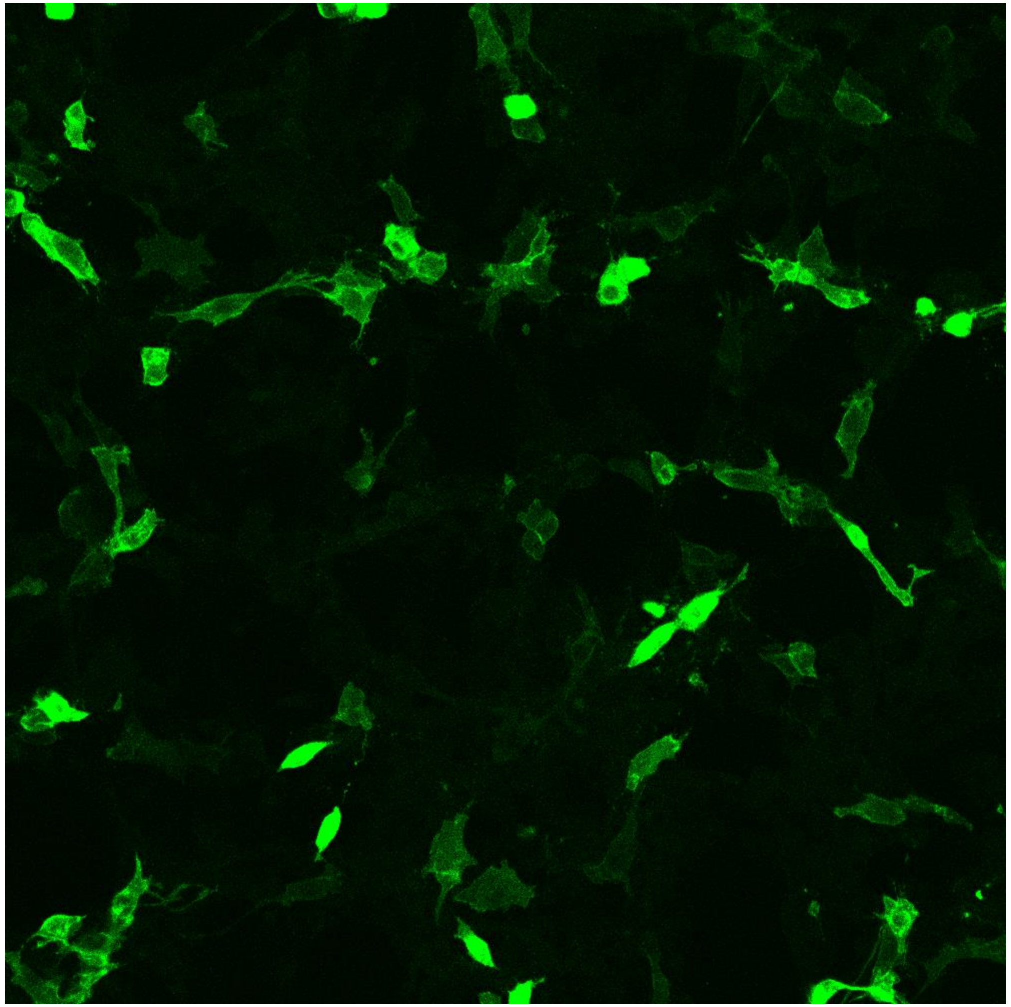

G106W + 5 µM 9-*cis*-retinal

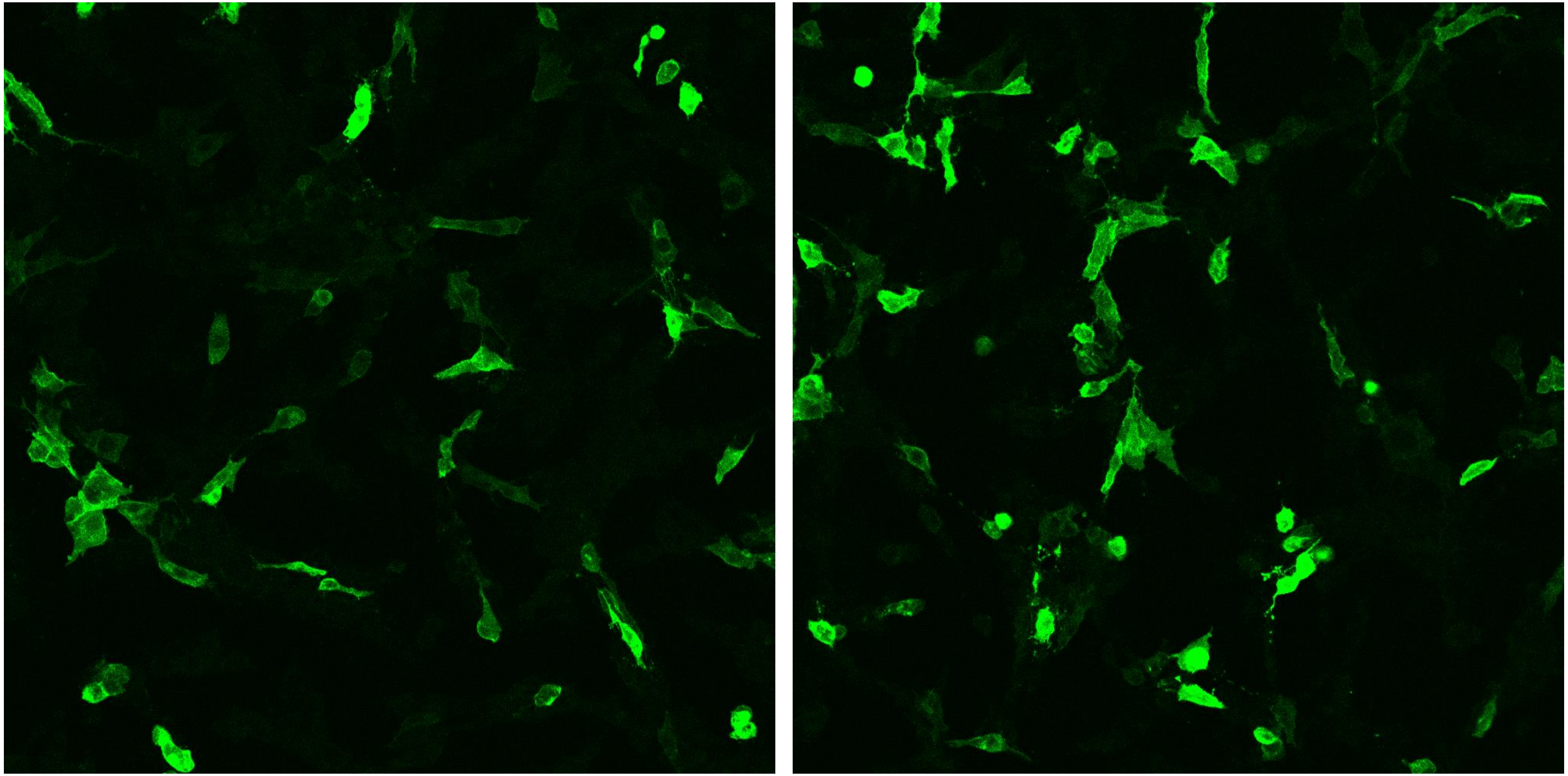

G106W + 40 μM YC-001

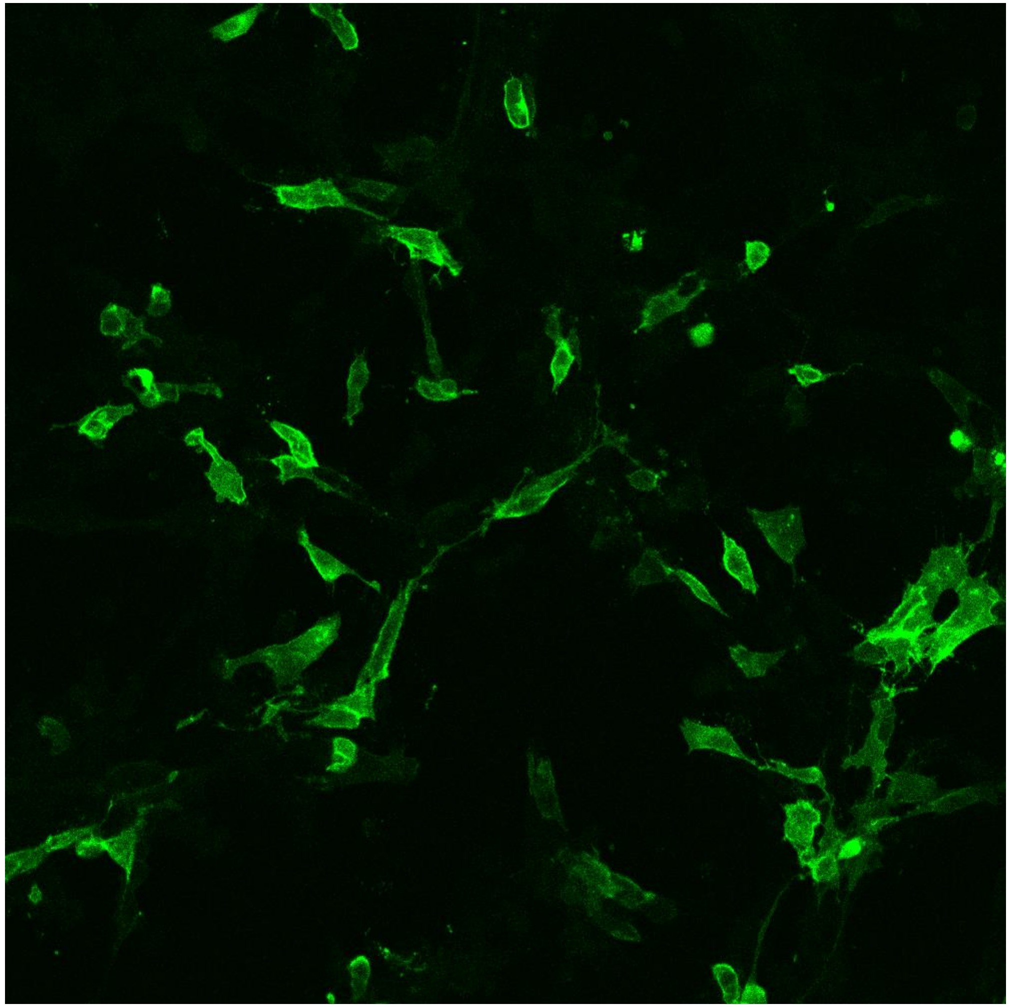

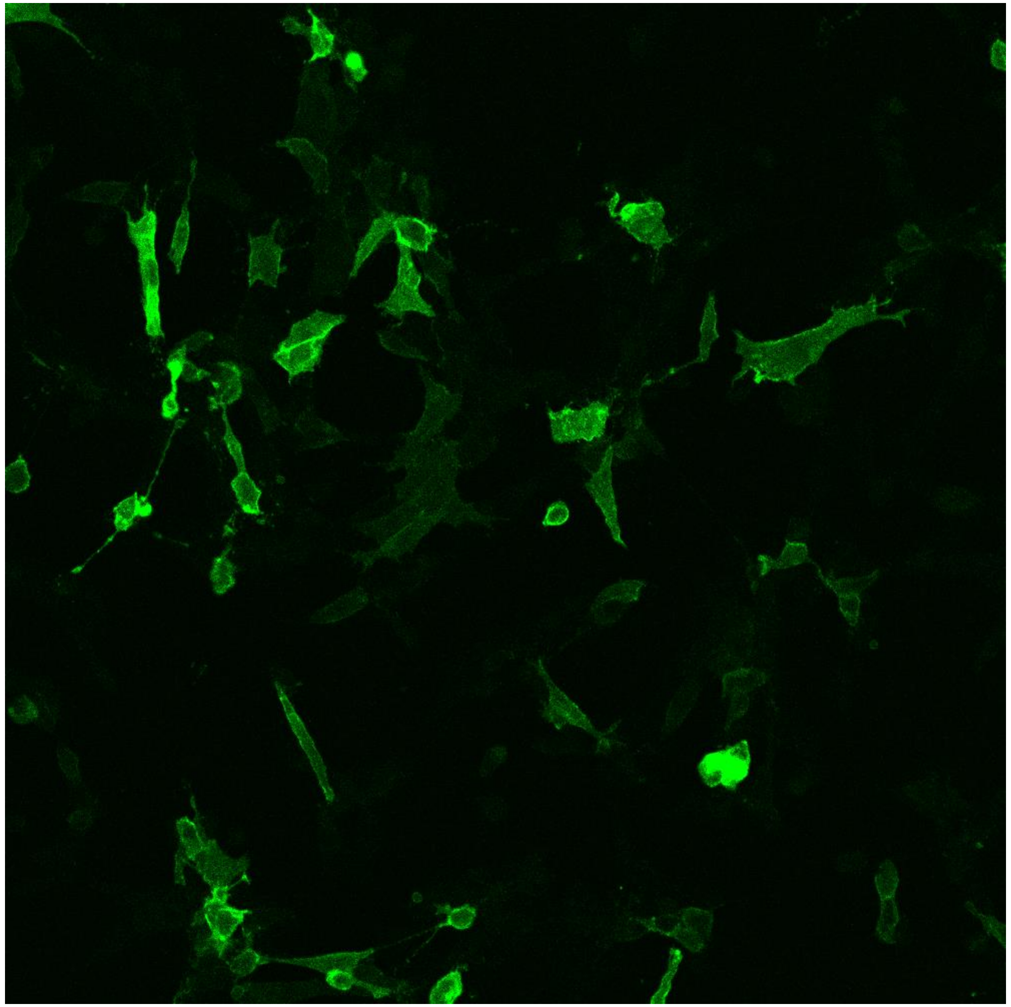

G106W + 20 µM F5257-0462

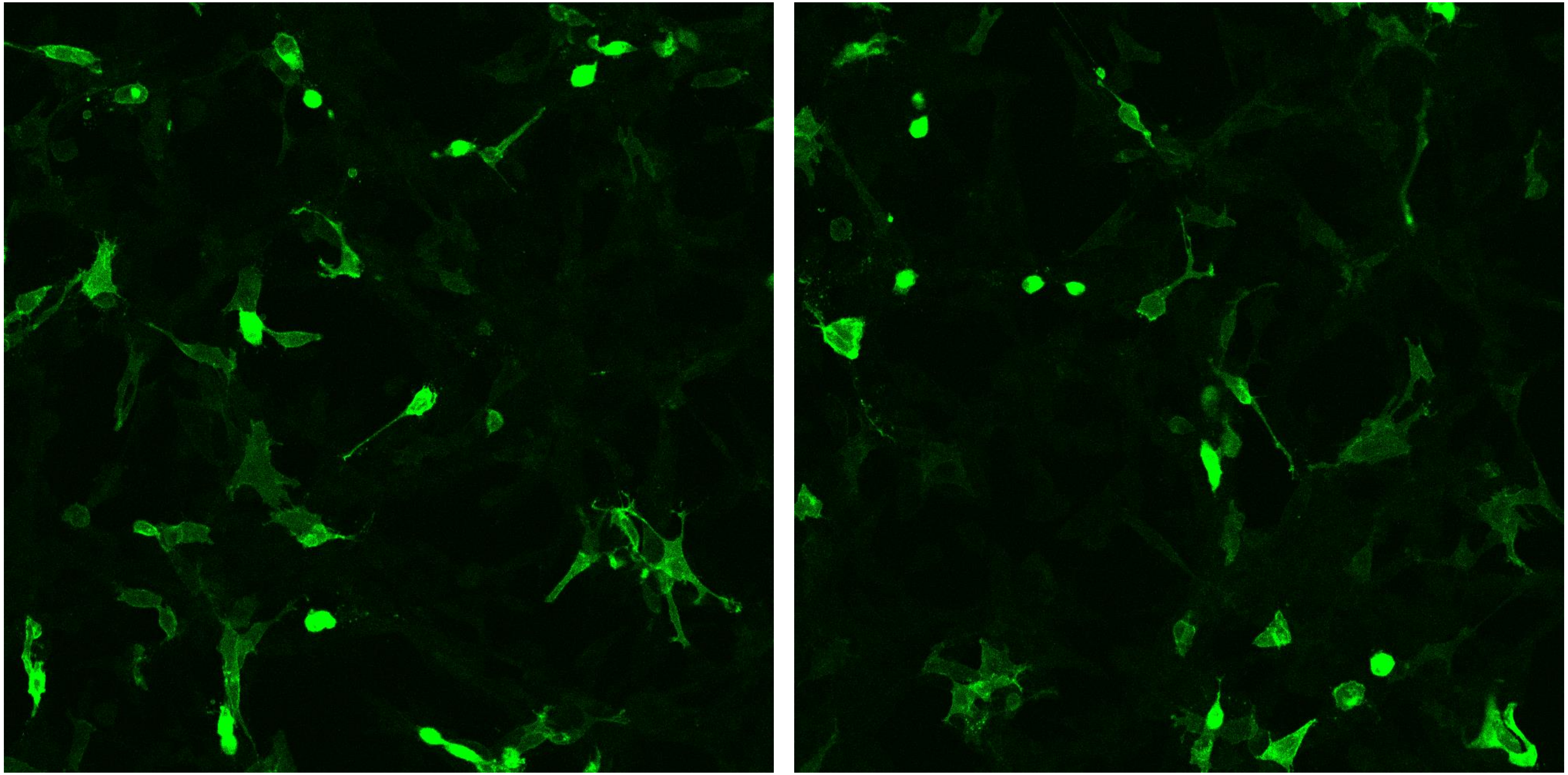

G109R + DMSO (0.1%)

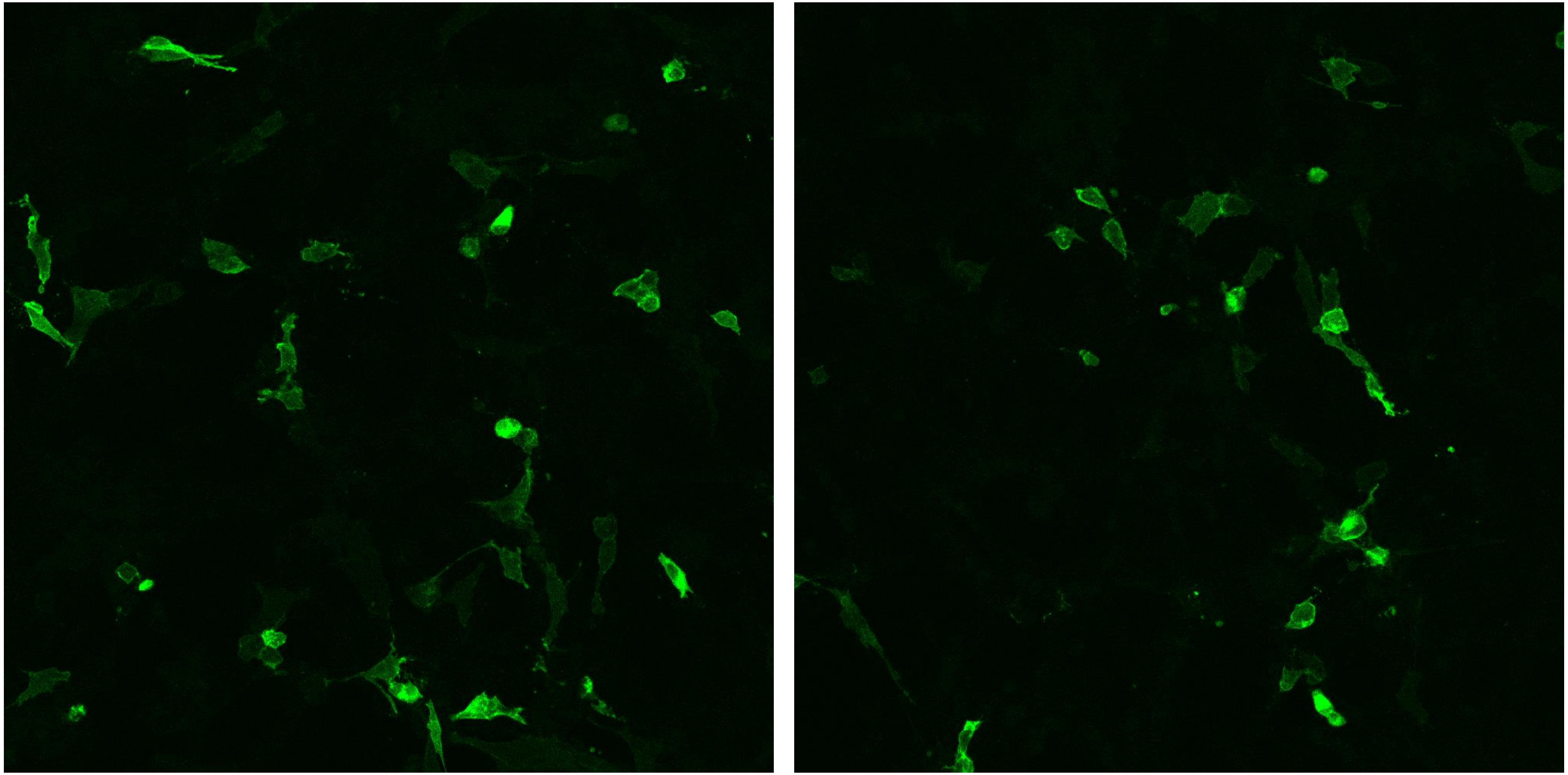

G109R + DMSO (0.1%)

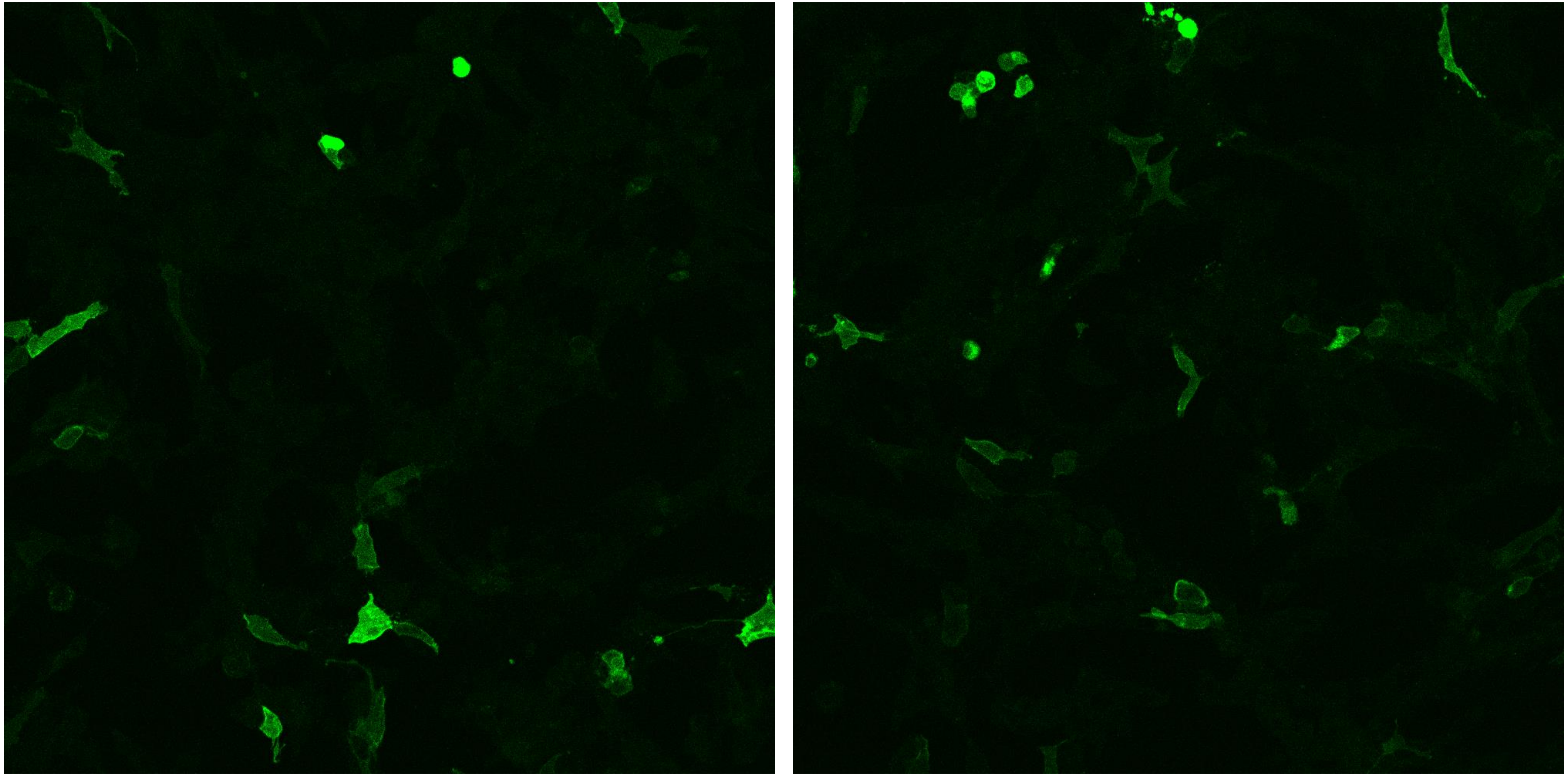

G109R + 5 µM 9-*cis*-retinal

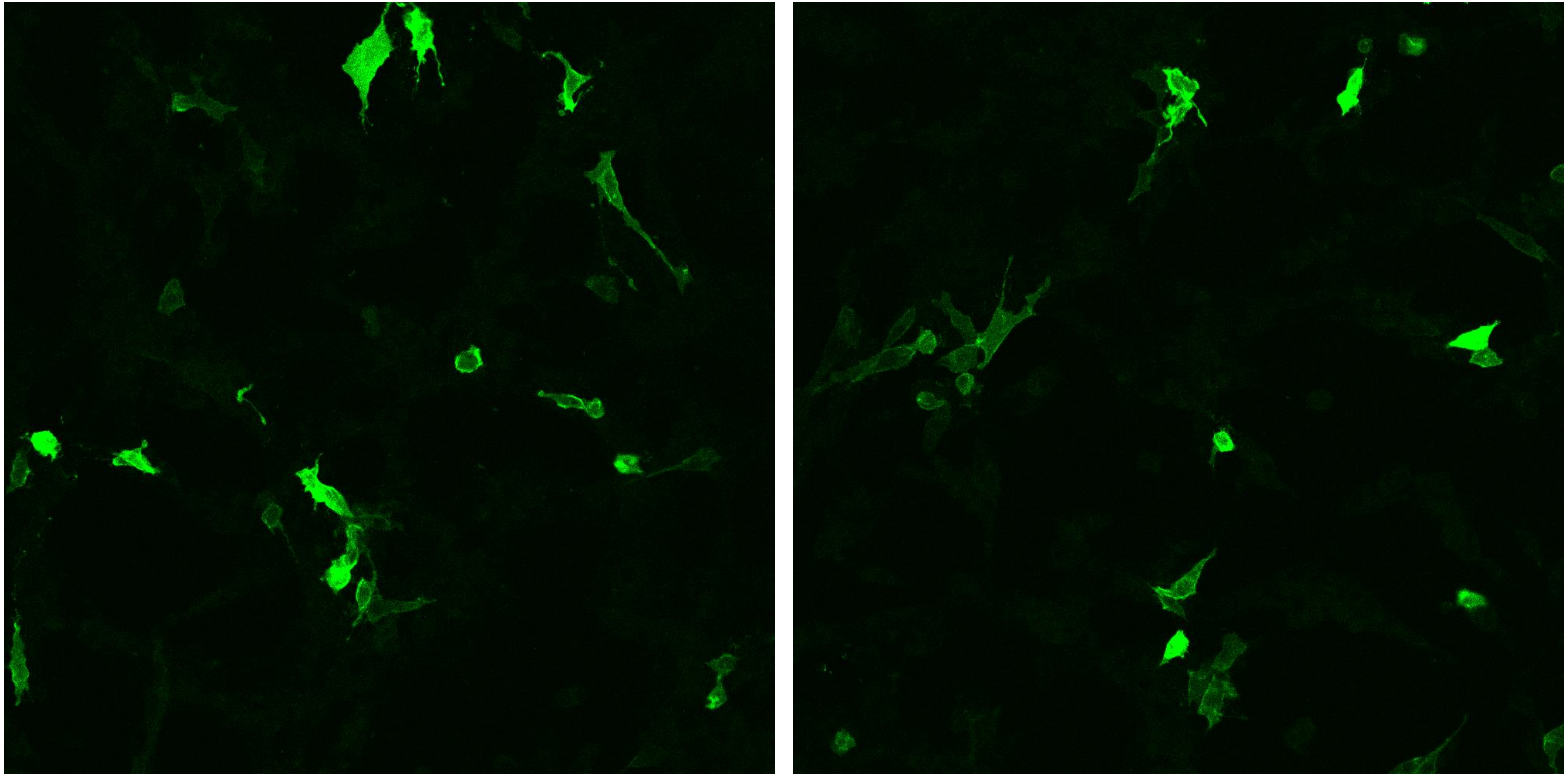

G109R + 40 μM YC-001

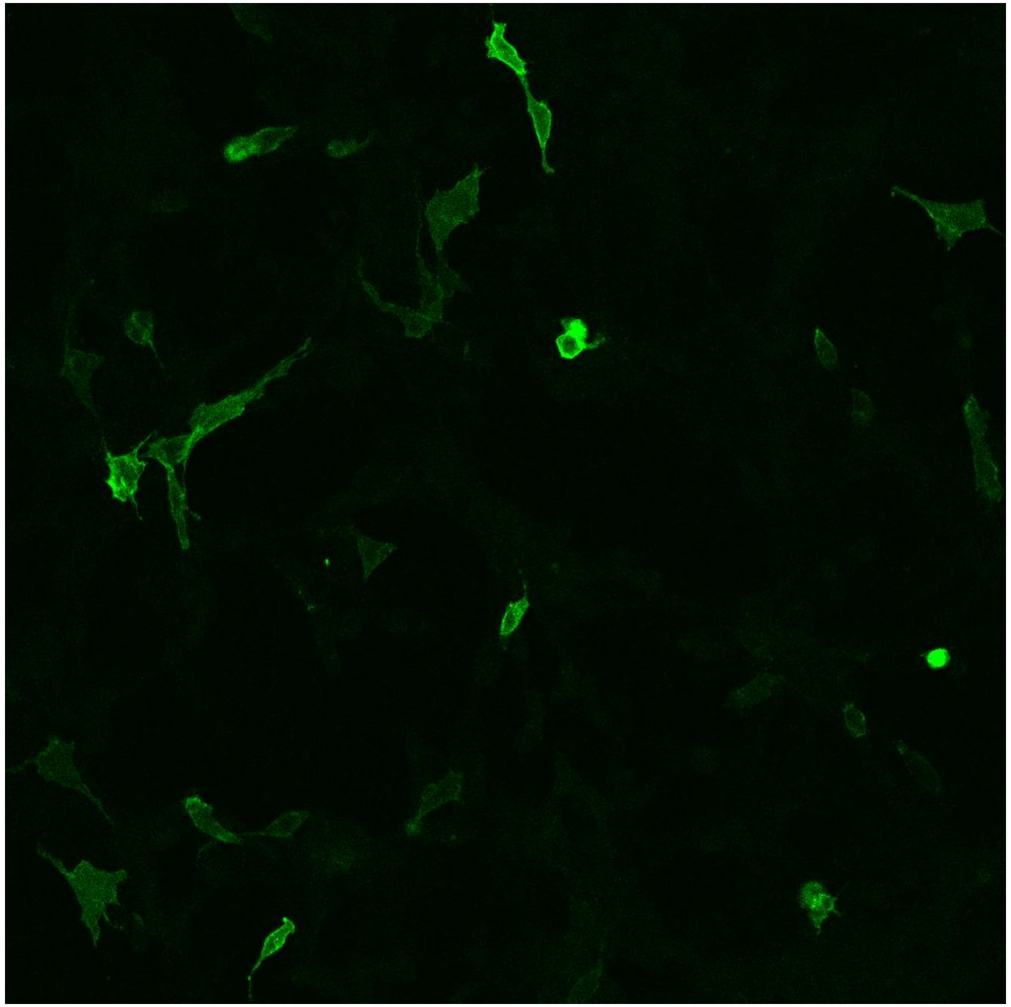

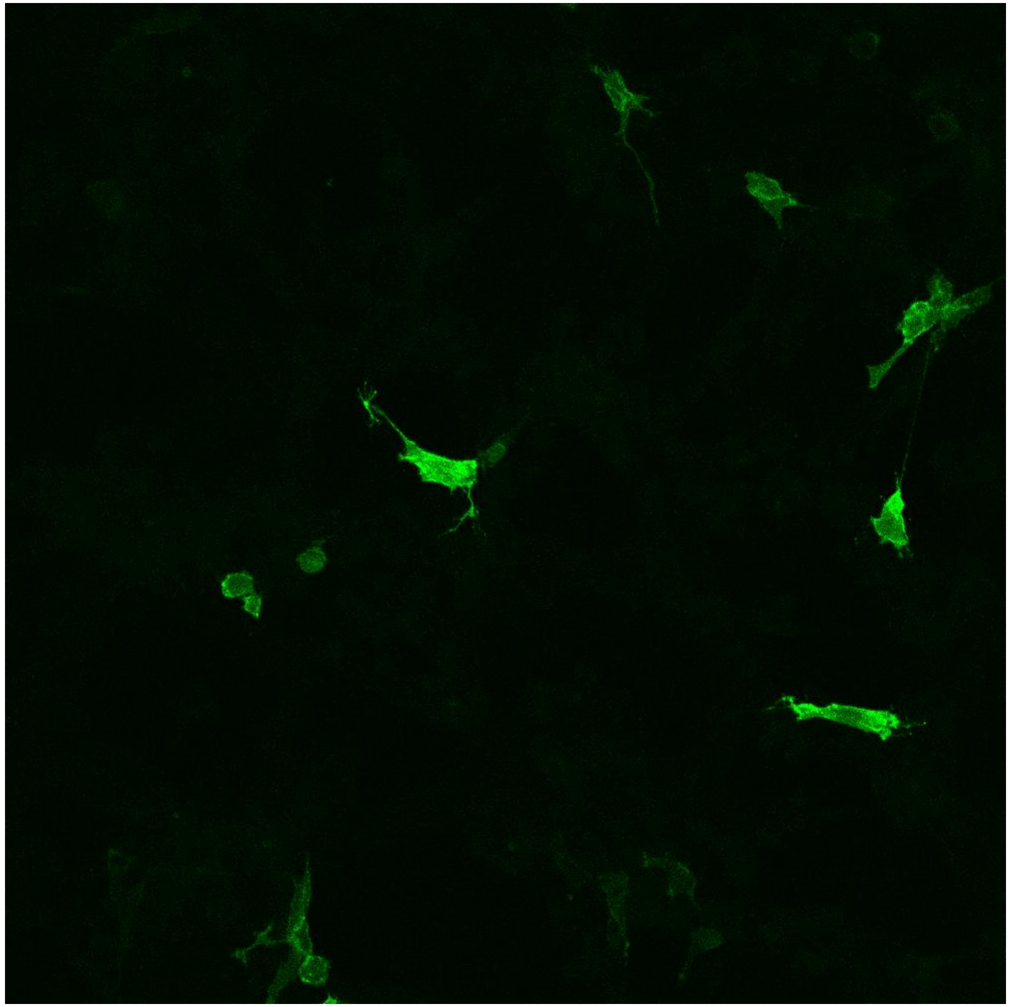

G109R + 20 µM F5257-0462

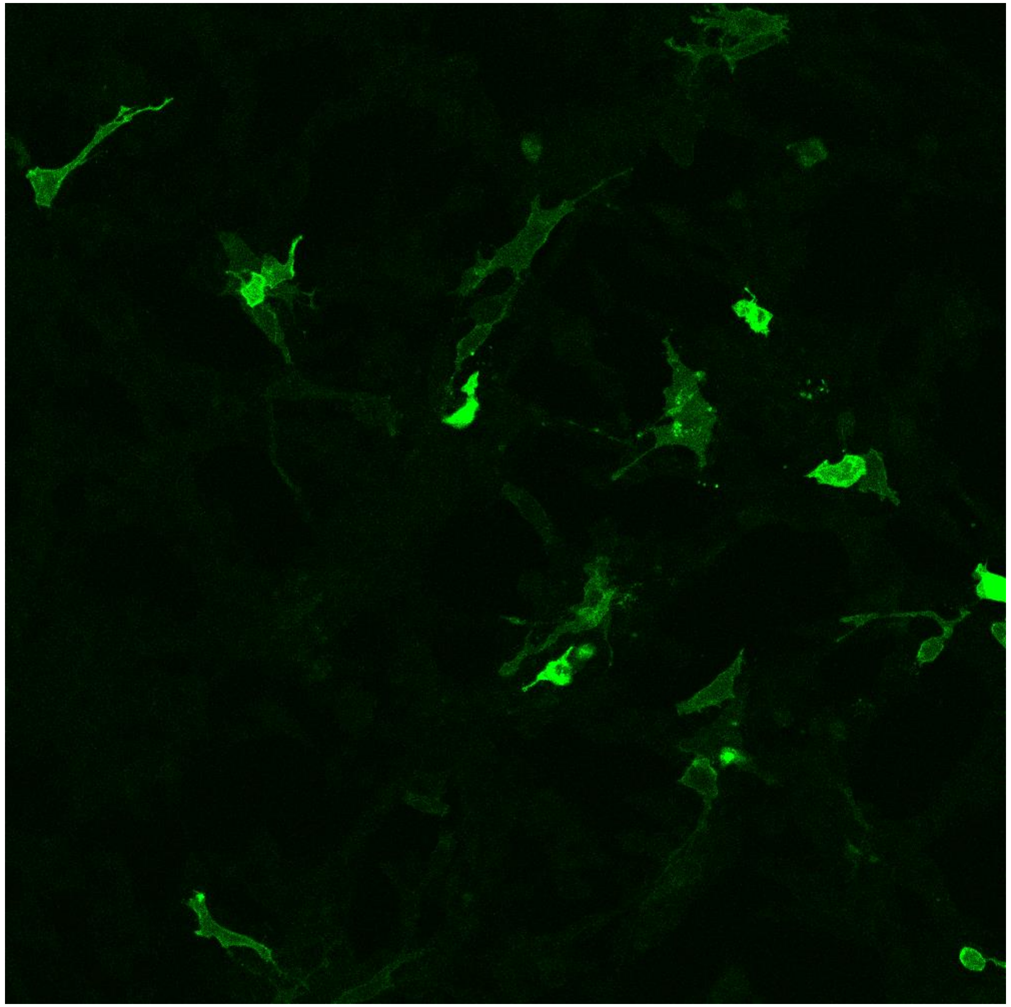

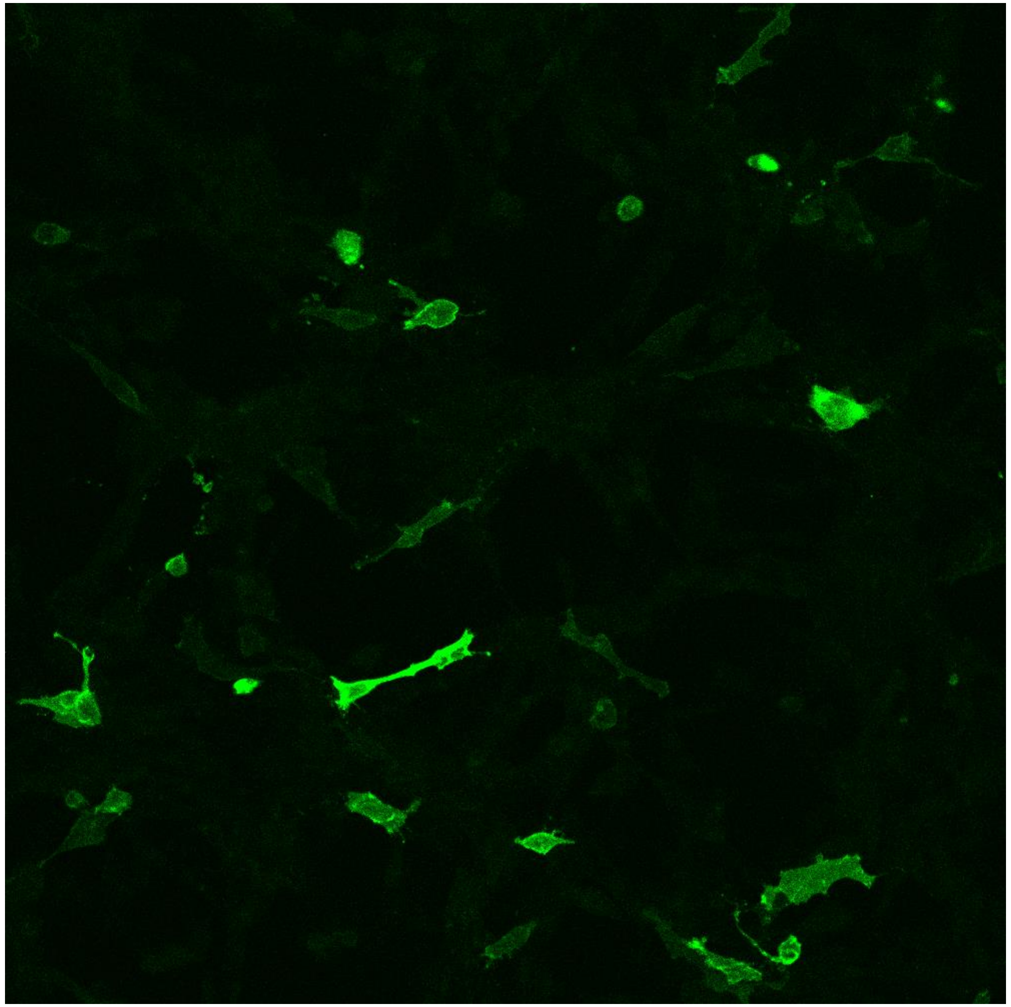

C110Y + DMSO (0.1%)

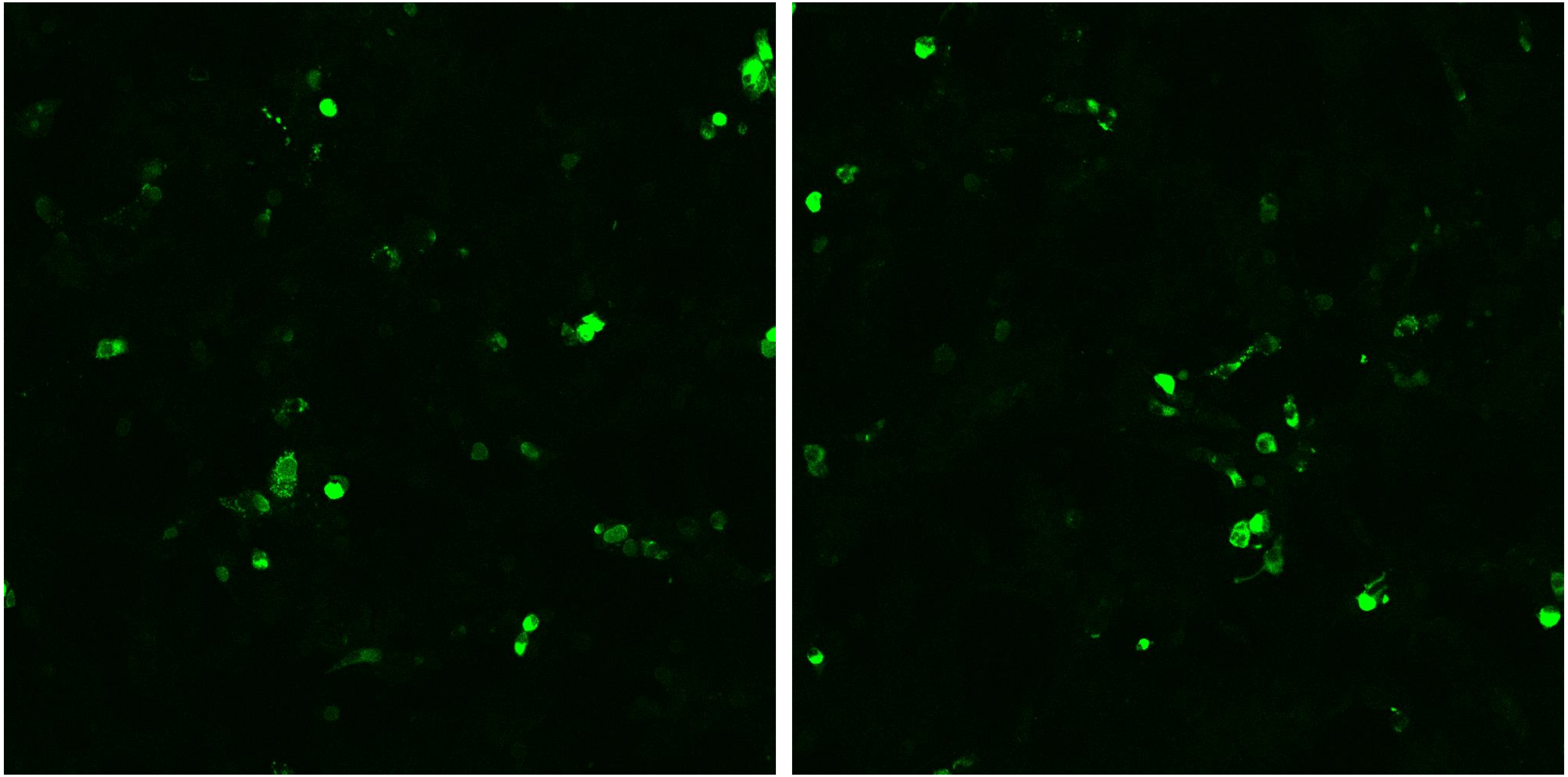

C110Y + DMSO (0.1%)

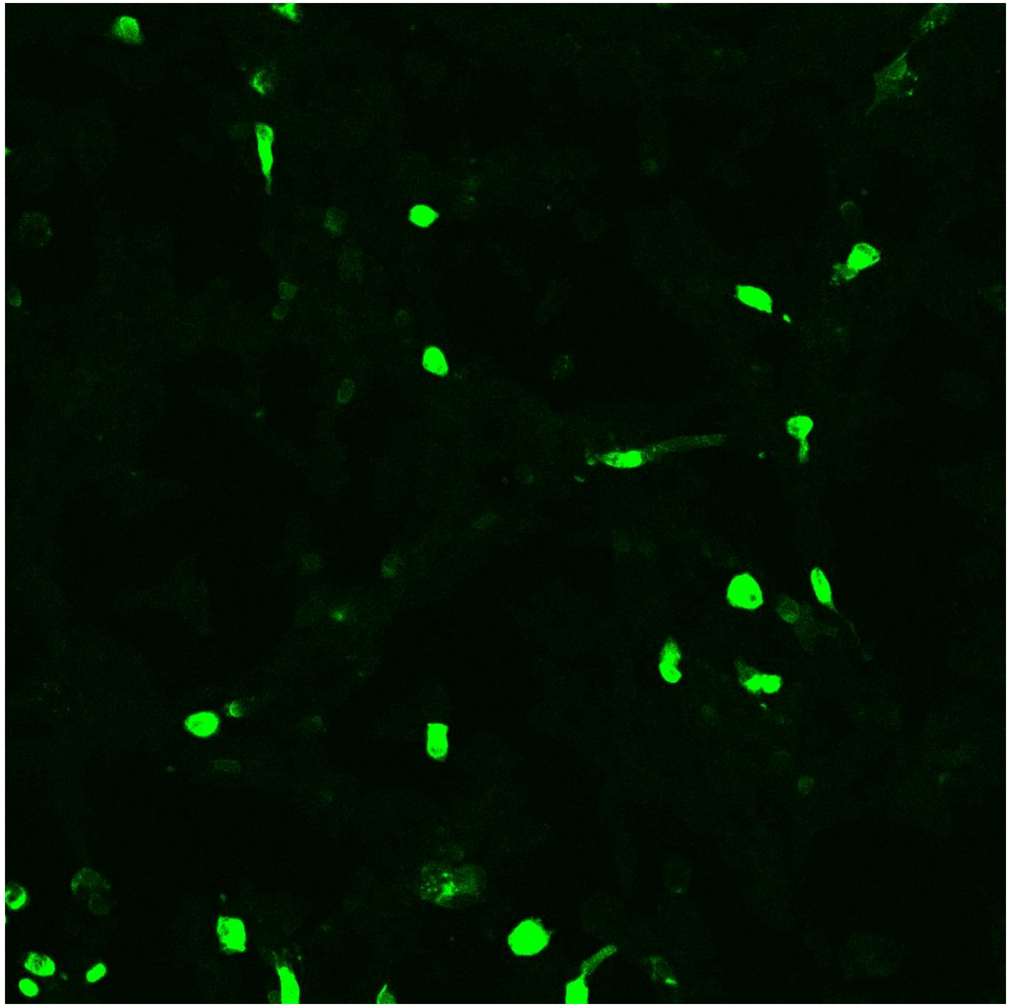

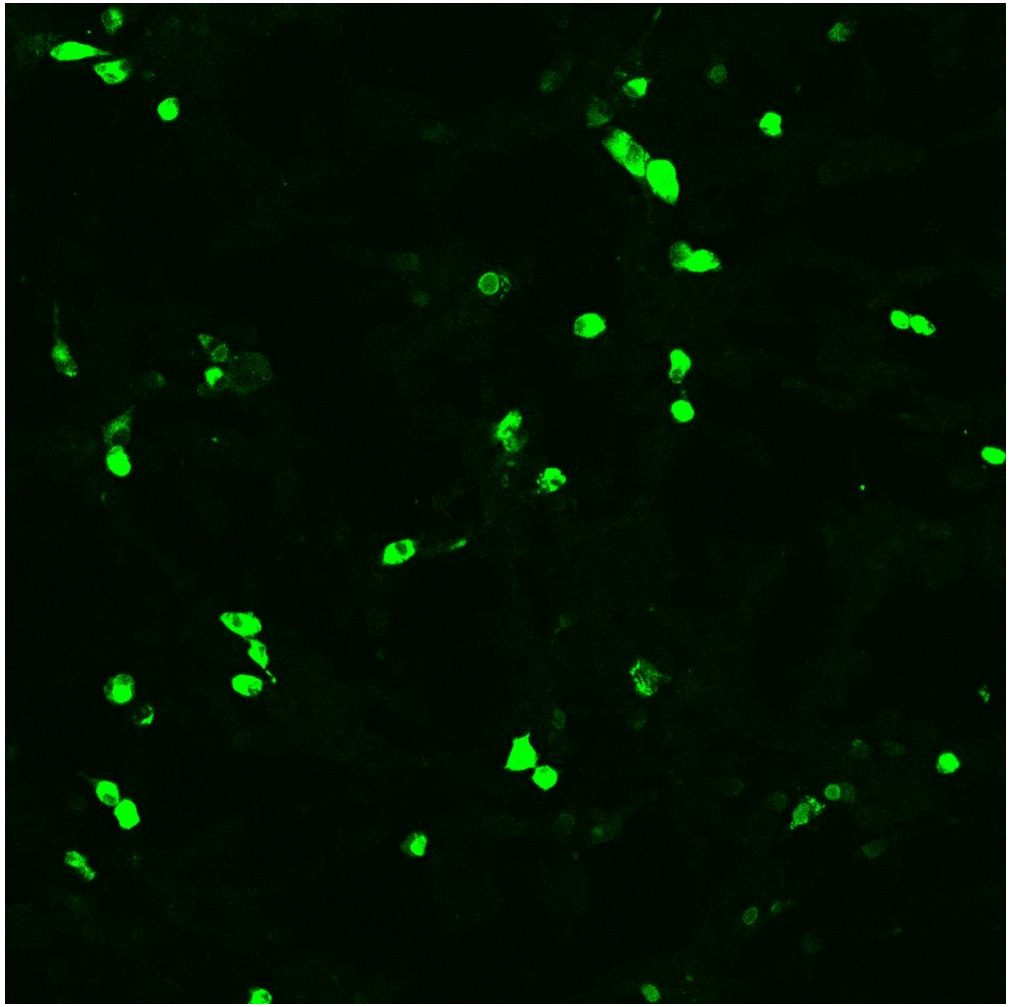

C110Y + 5 μM 9-*cis*-retinal

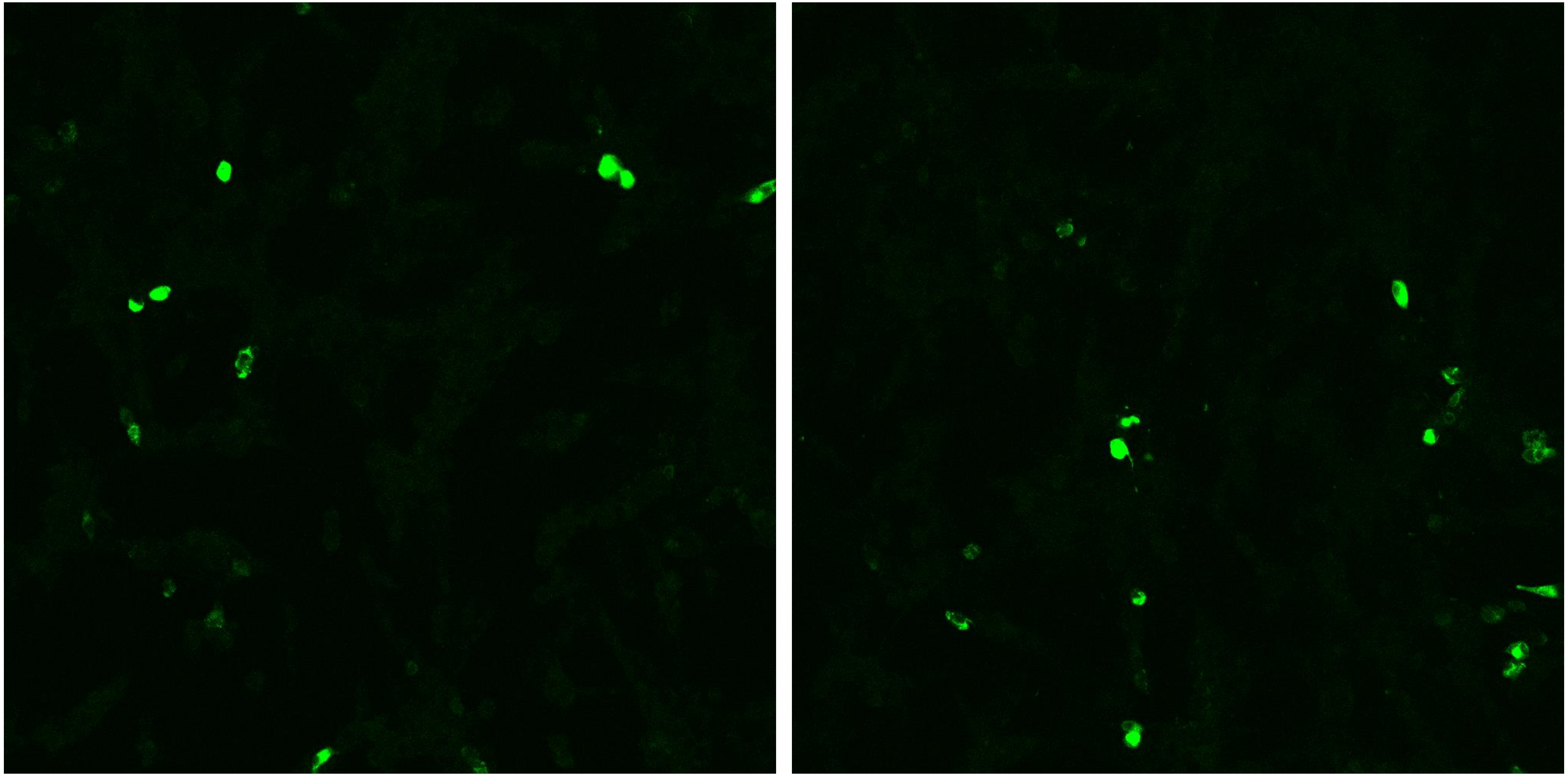

C110Y + 40 μM YC-001

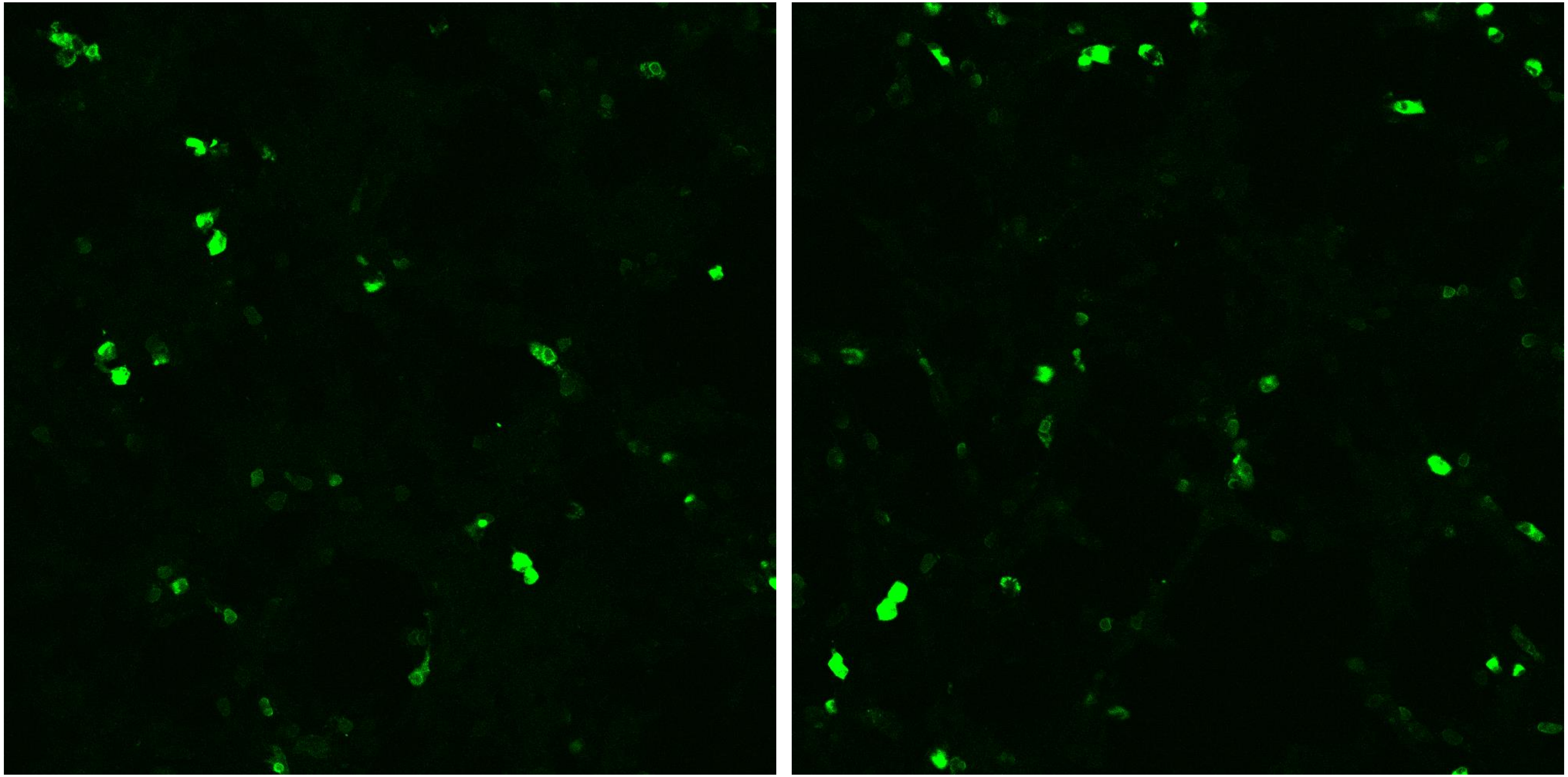

C110Y + 20 μM F5257-0462

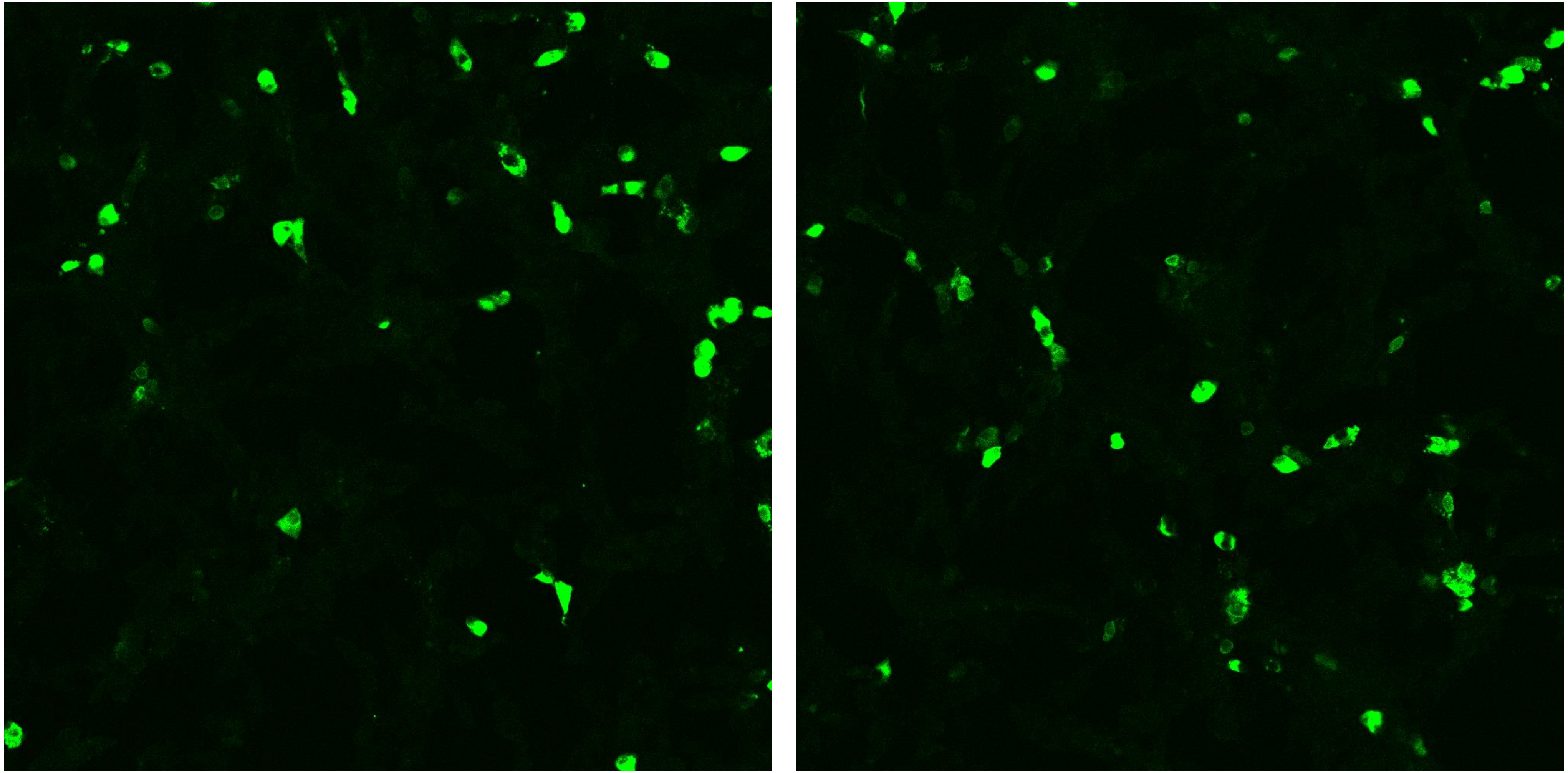

Y178N + DMSO (0.1%)

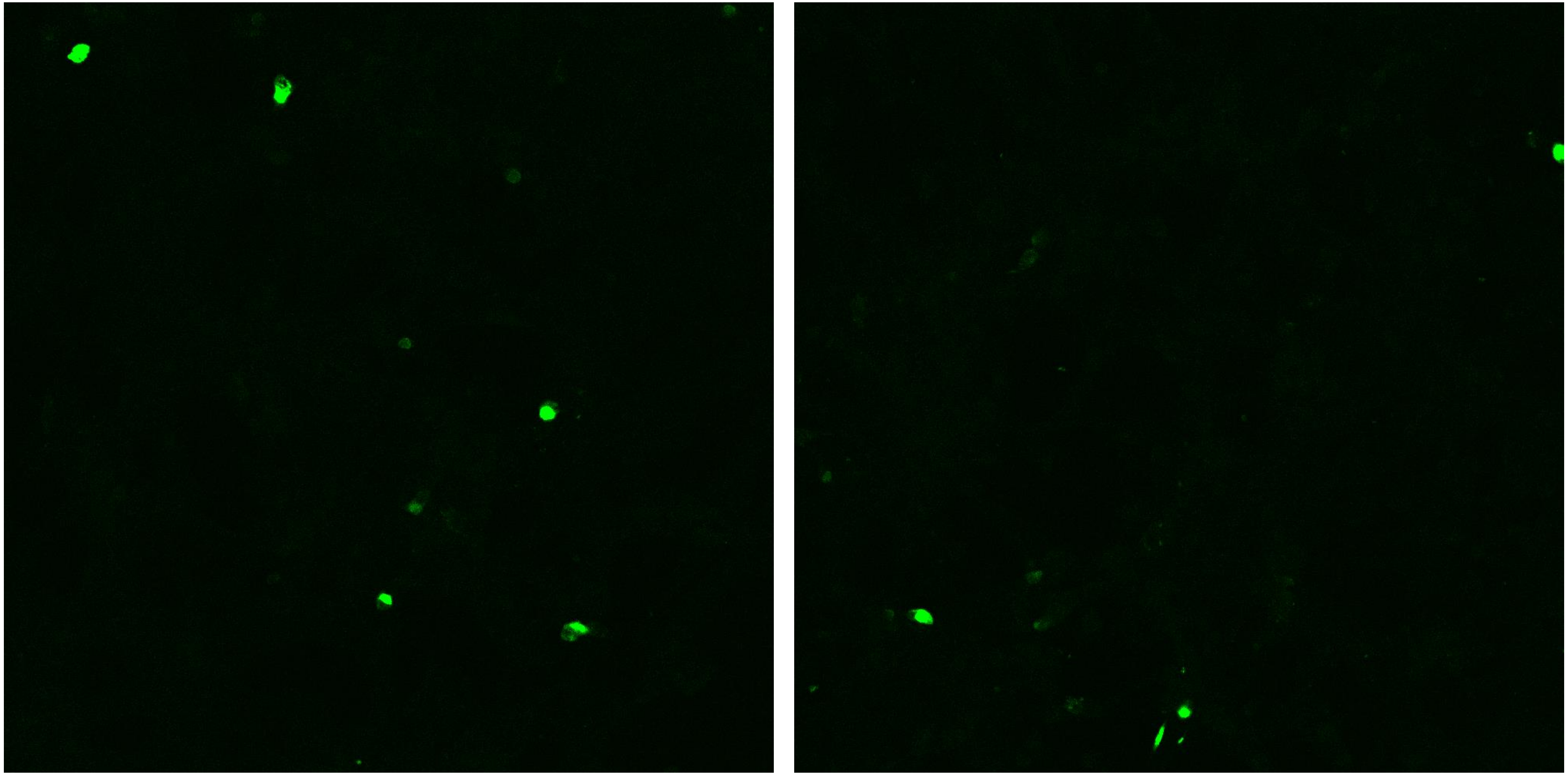

Y178N + DMSO (0.1%)

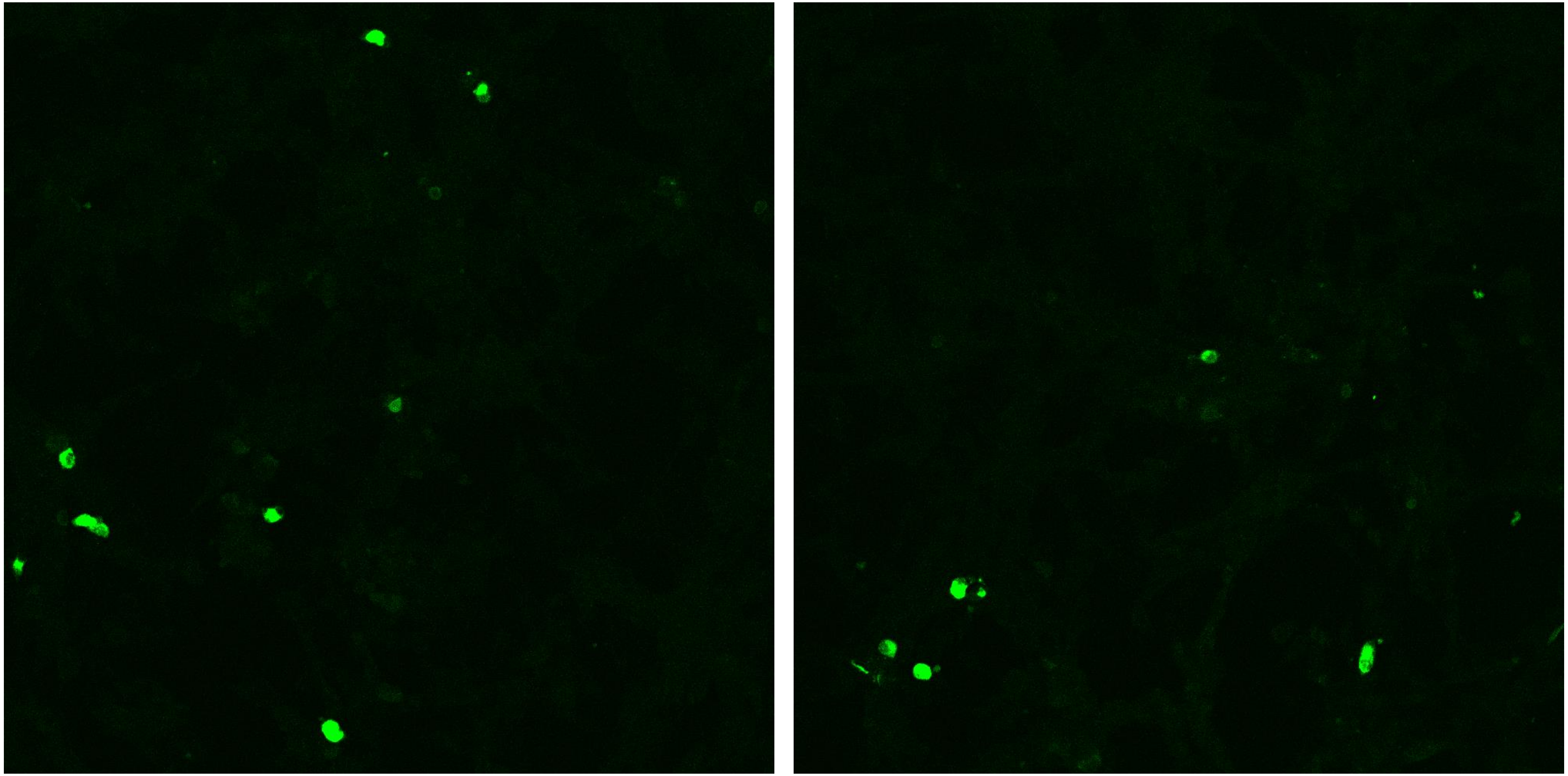

Y178N + 5 µM 9-*cis*-retinal

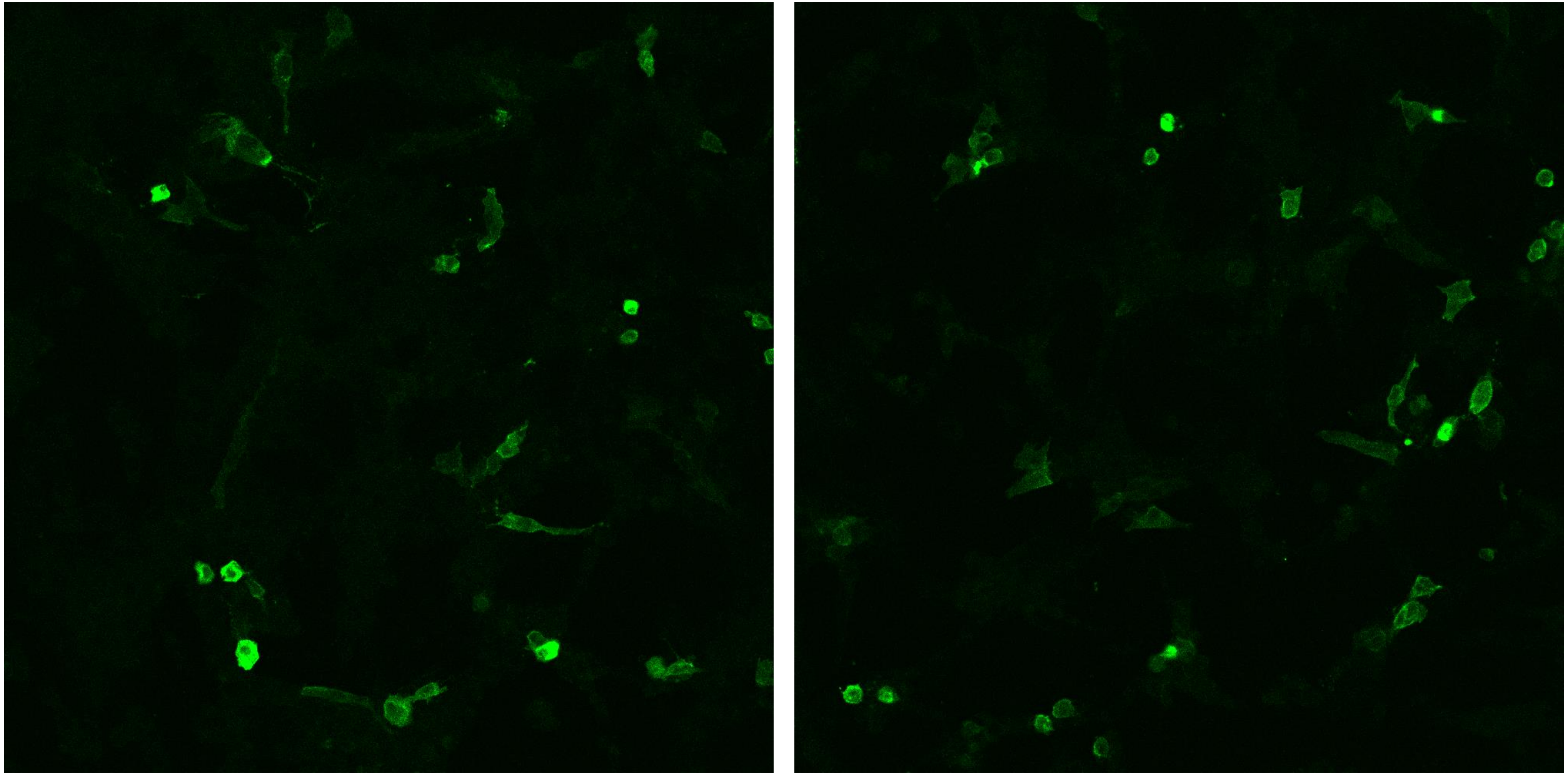

Y178N + 40 μM YC-001

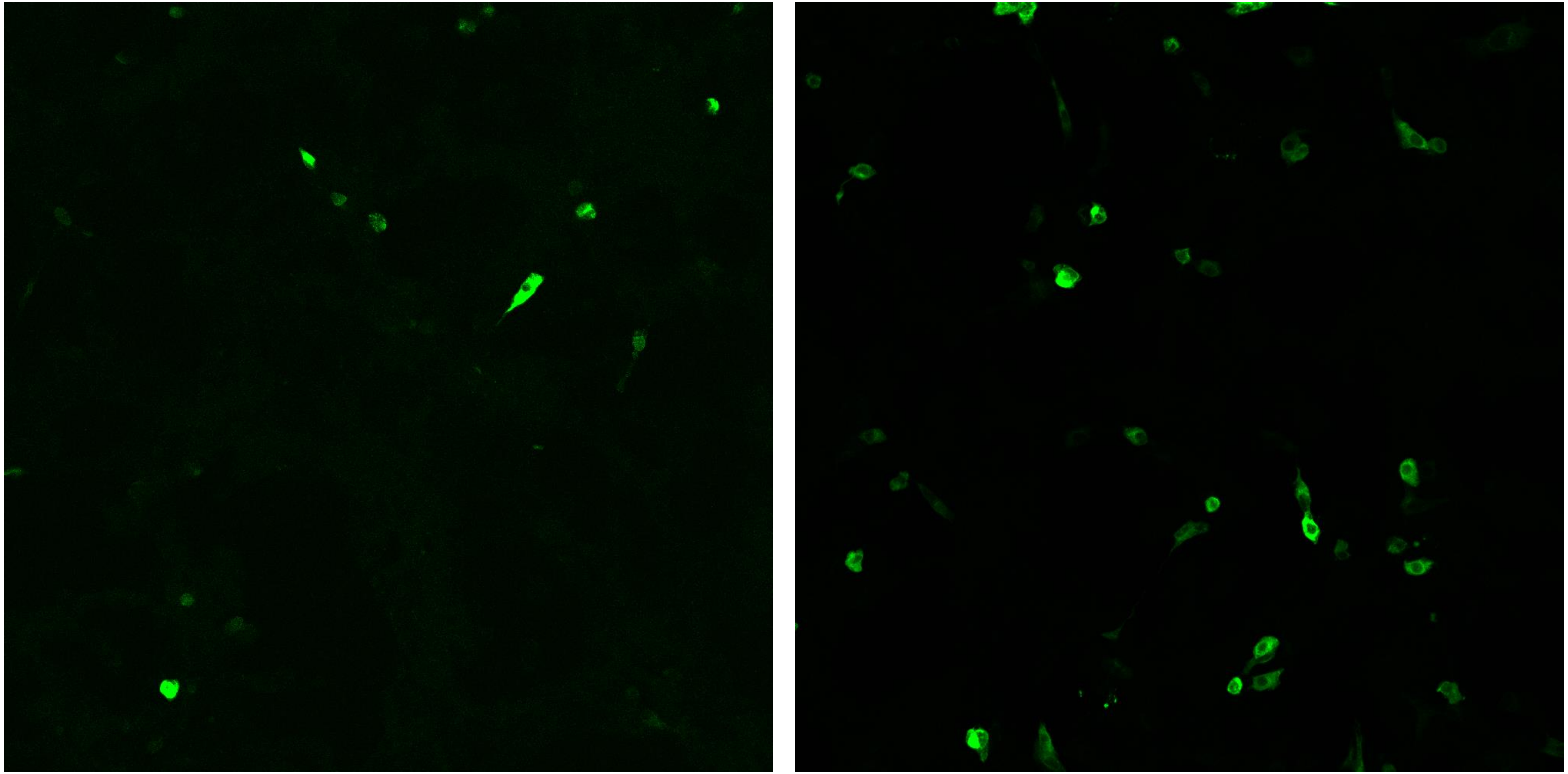

Y178N + 20 μM F5257-0462

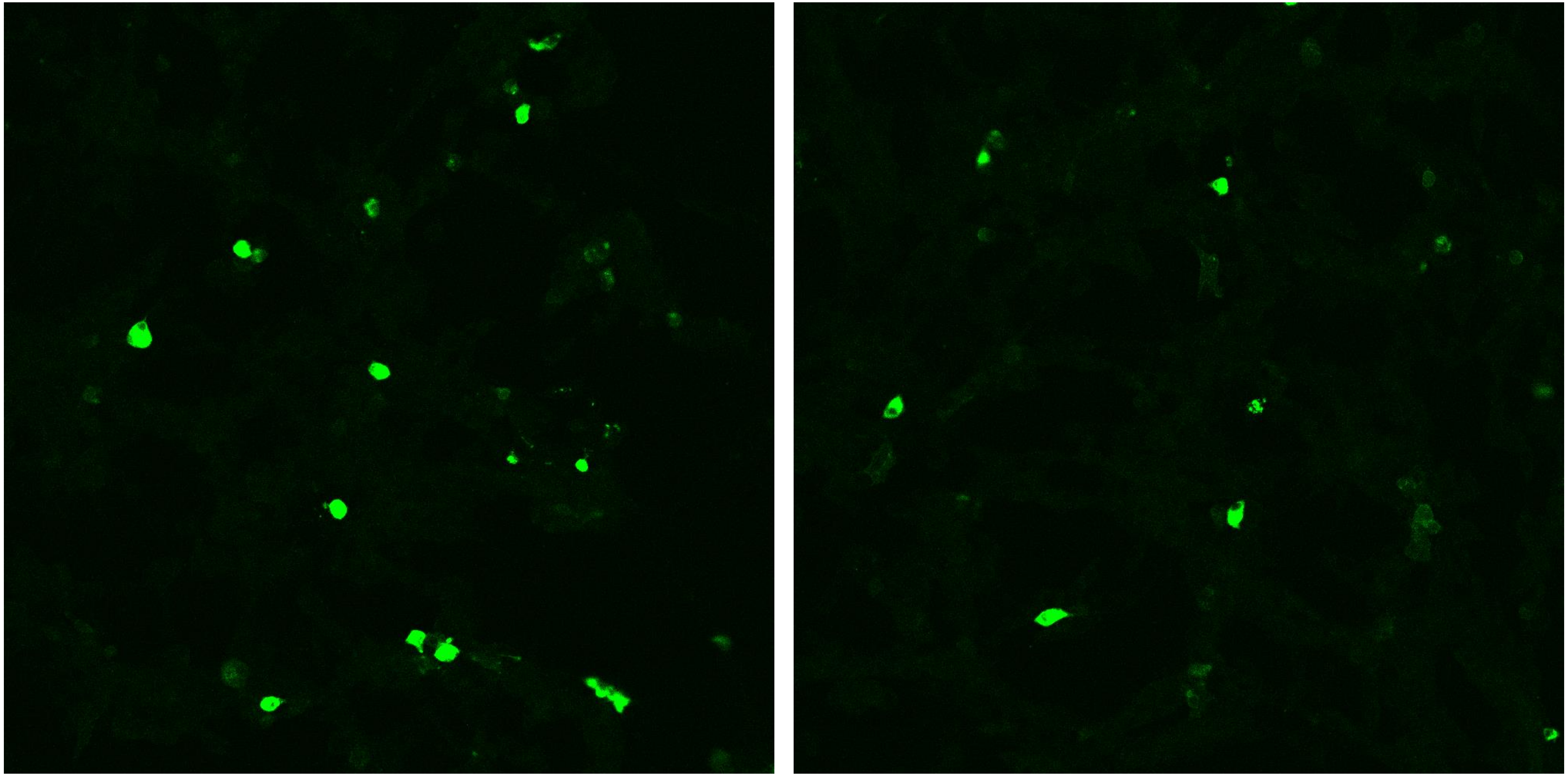

Y178C + DMSO (0.1%)

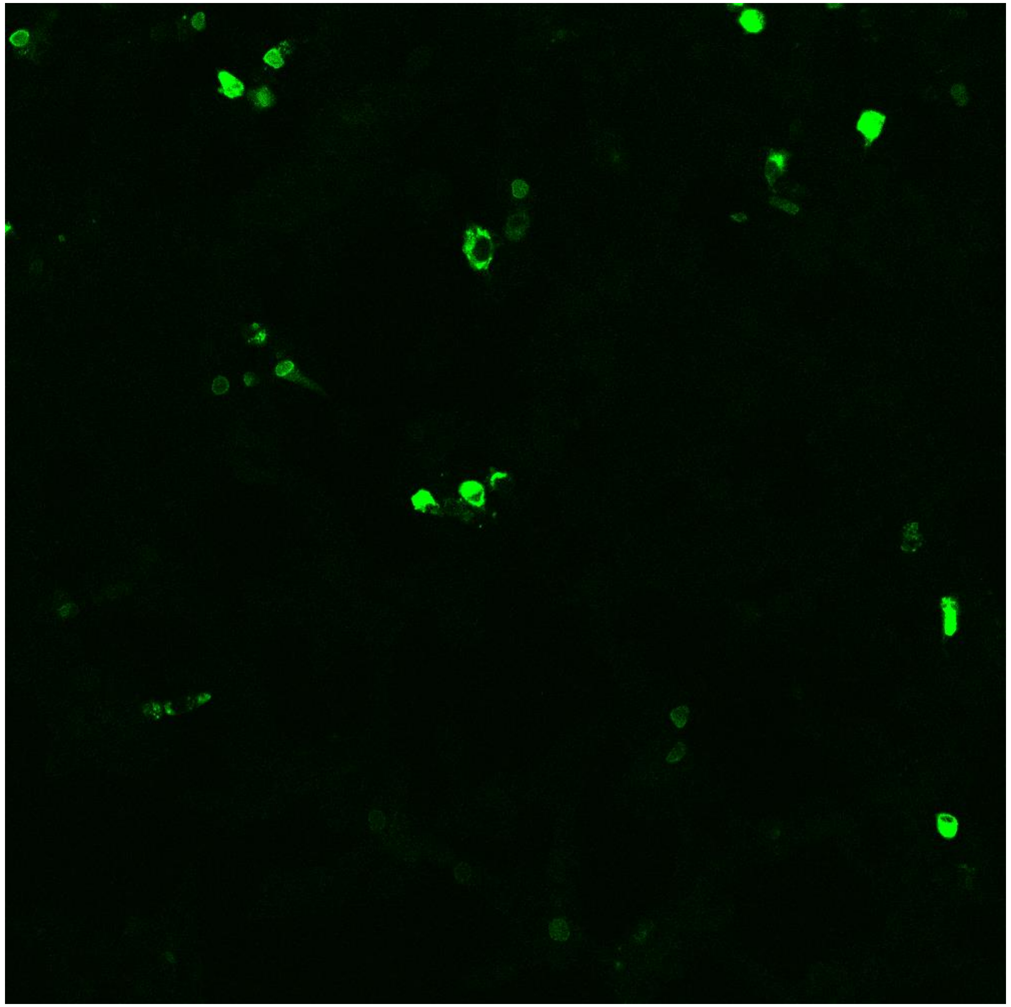

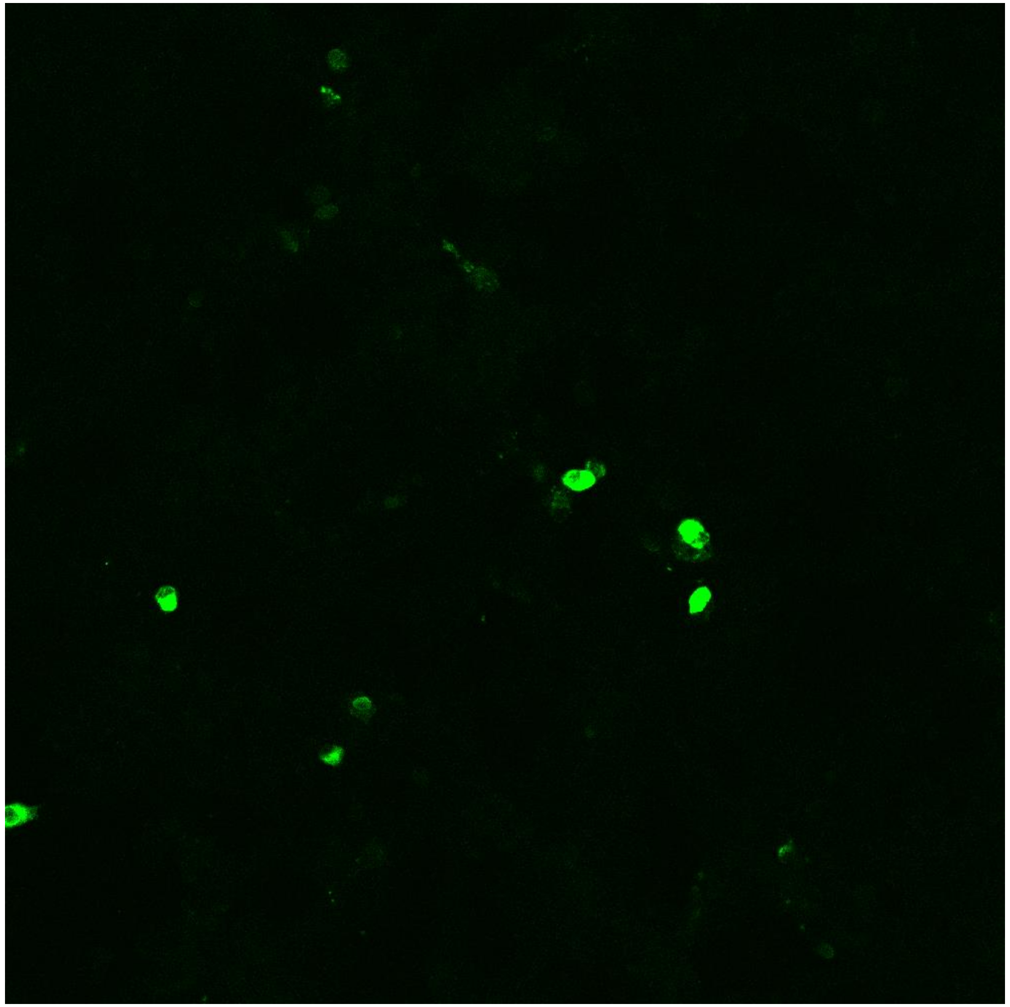

Y178C + DMSO (0.1%)

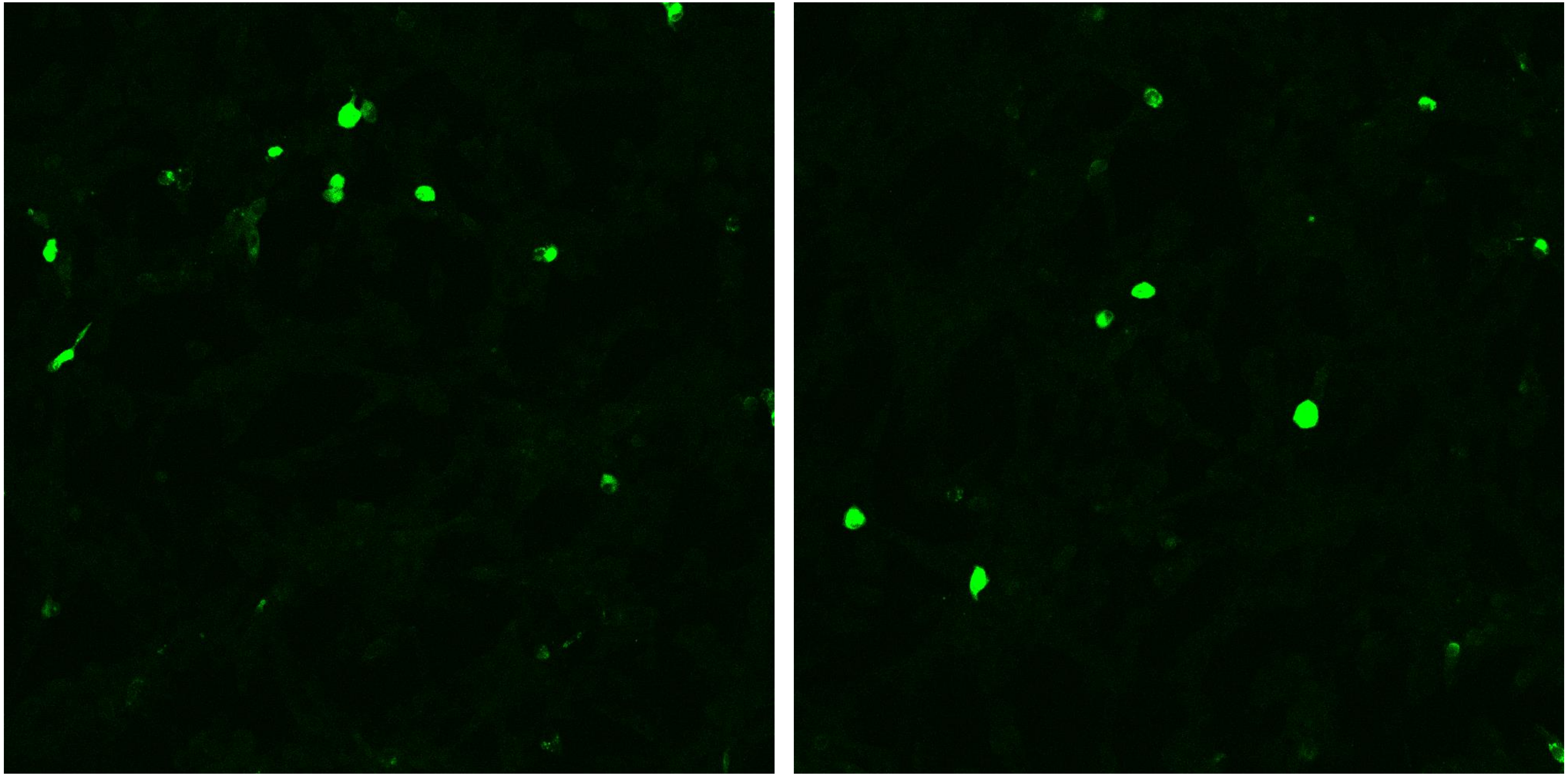

Y178C + 5 μM 9-*cis*-retinal

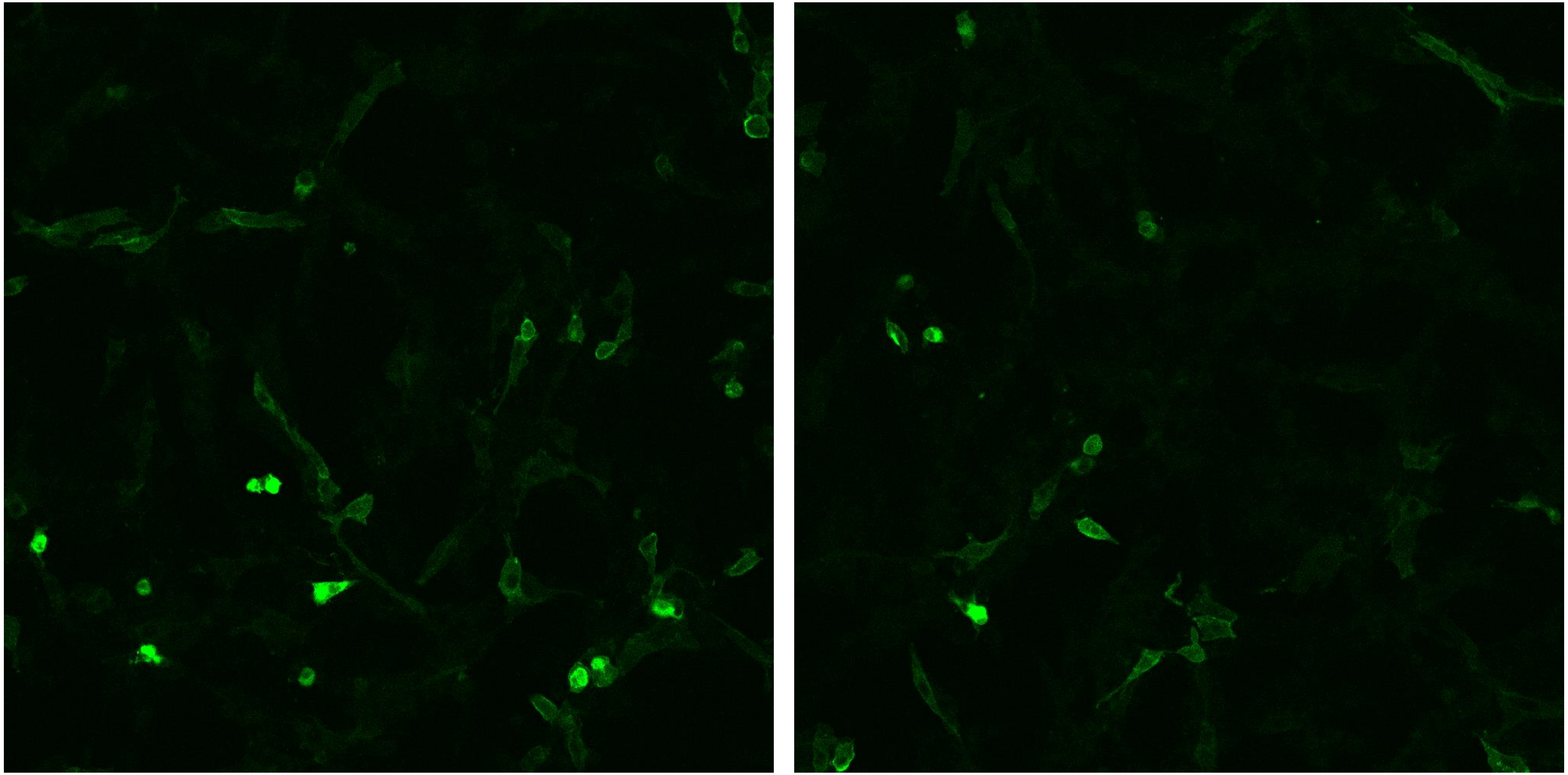

Y178C + 40 µM YC-001

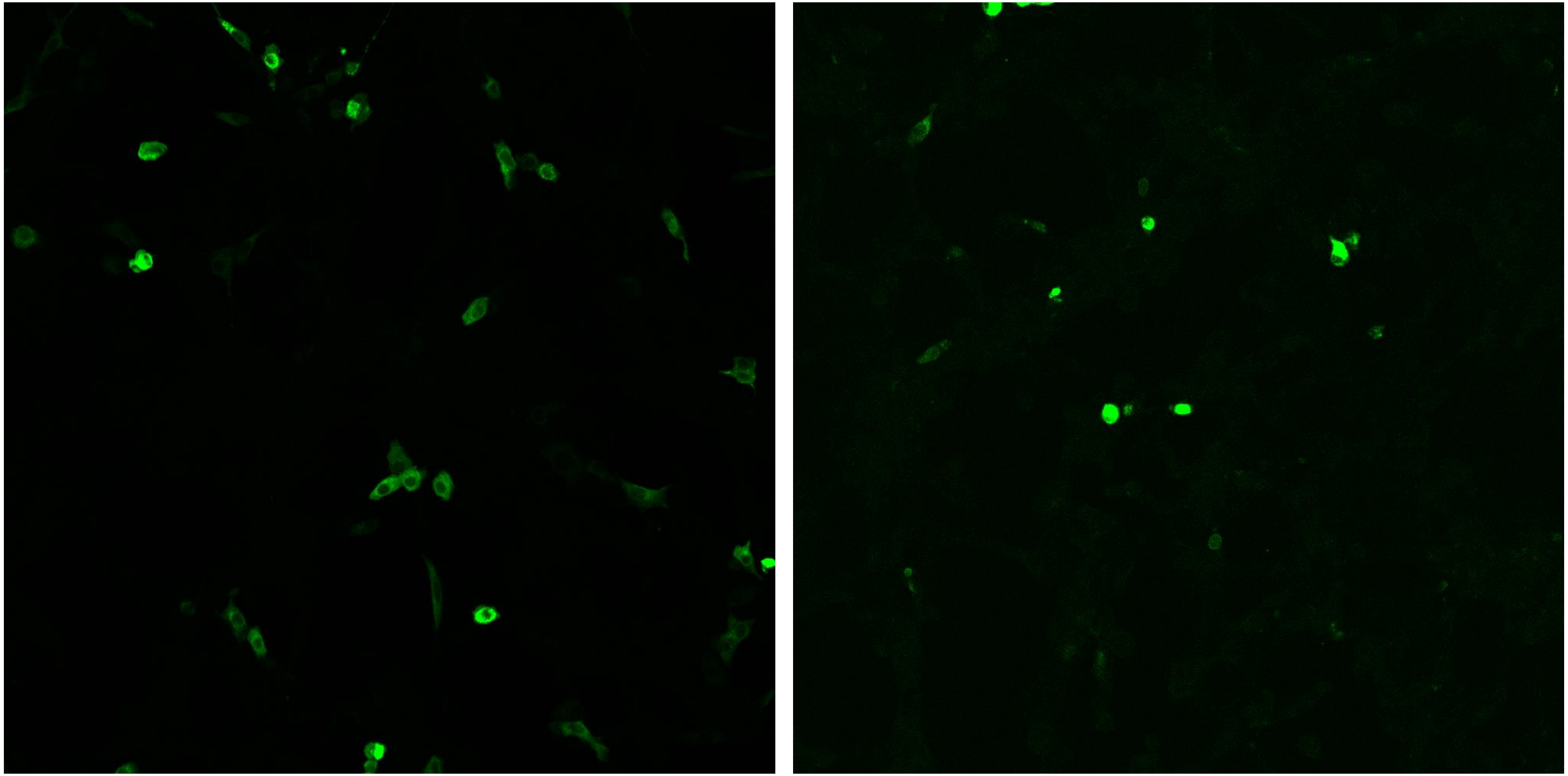

Y178C + 20 μM F5257-0462

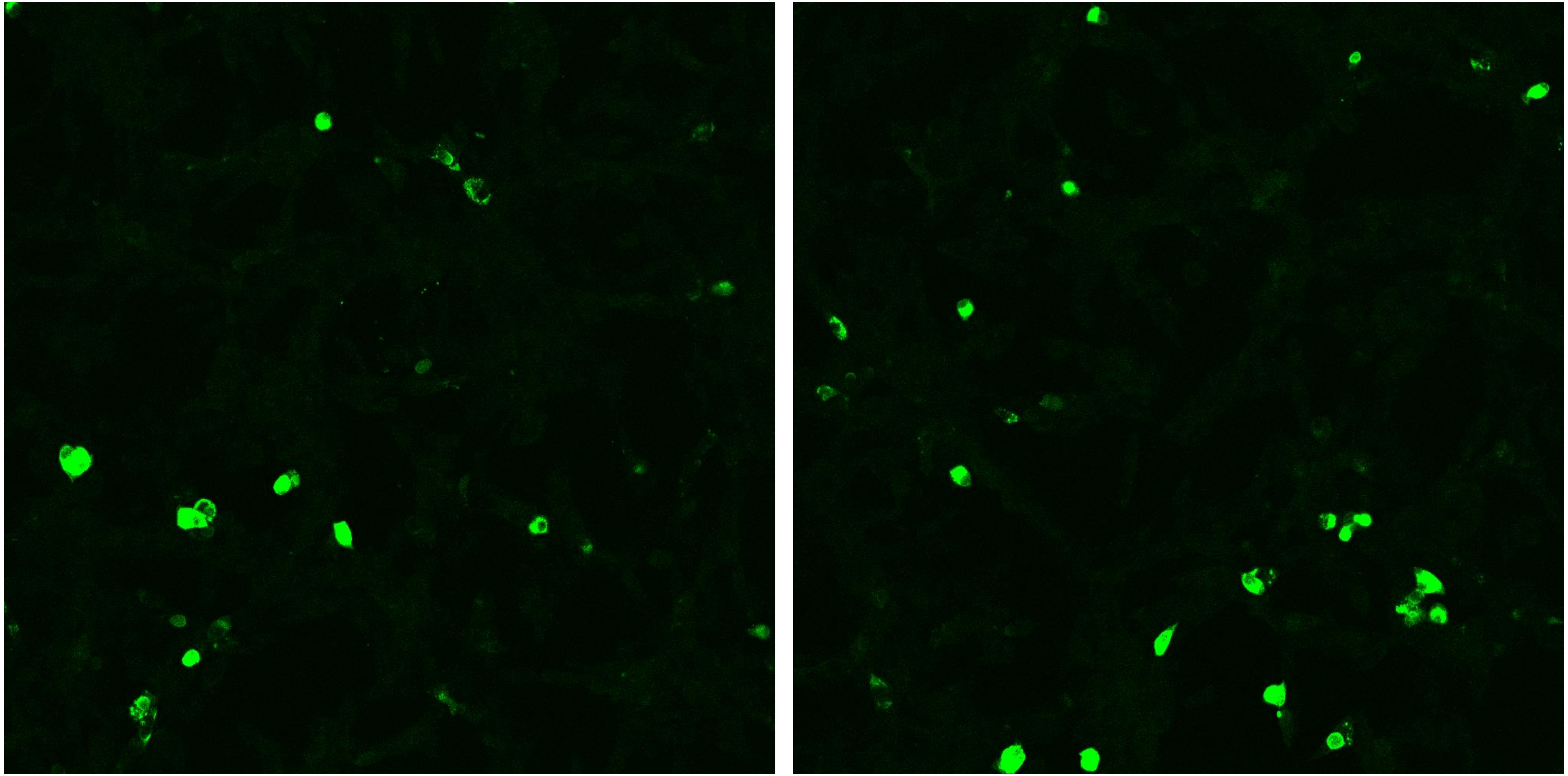

P180A + DMSO (0.1%)

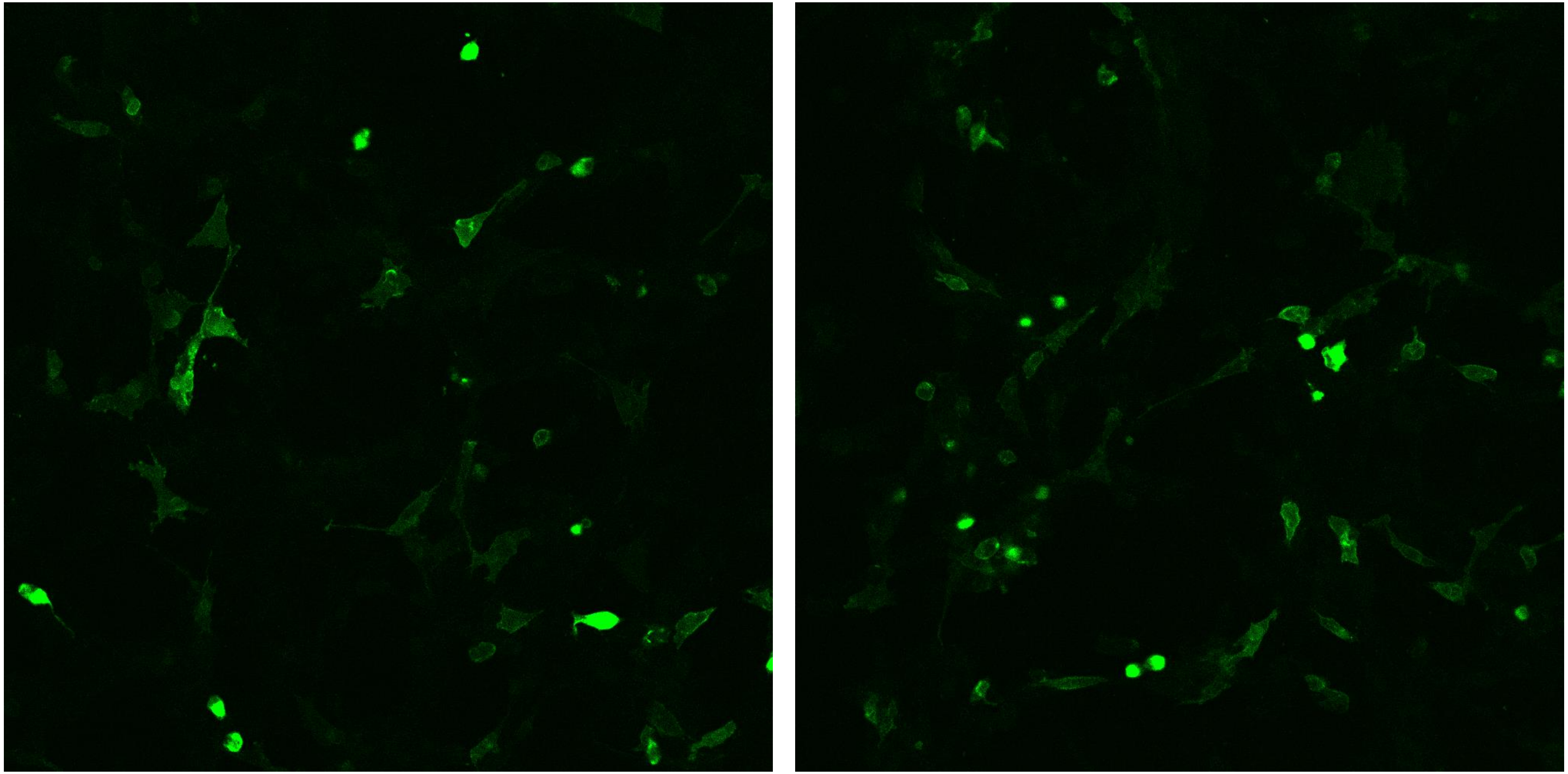

P180A + DMSO (0.1%)

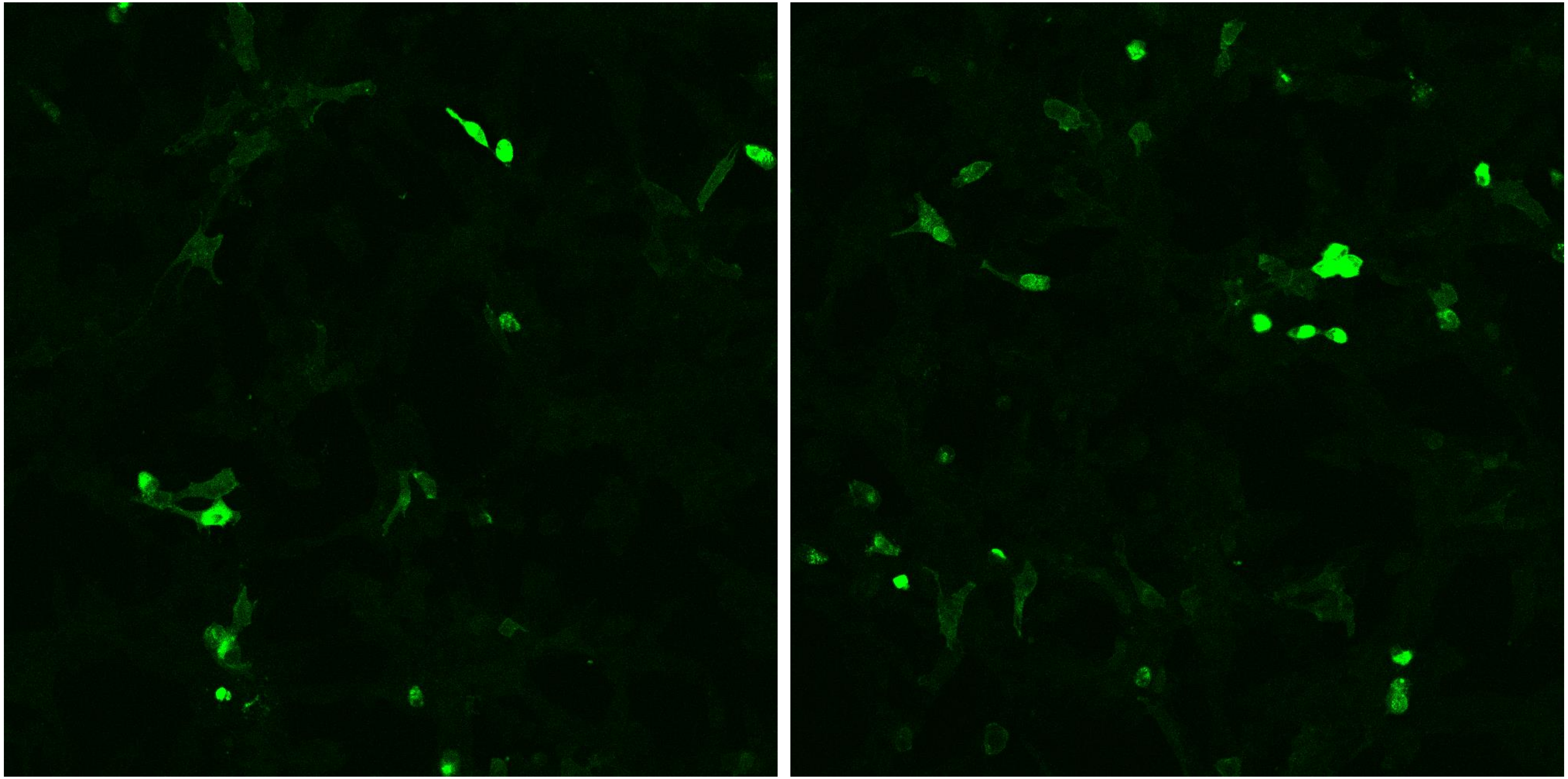

P180A + 5 μM 9-*cis*-retinal

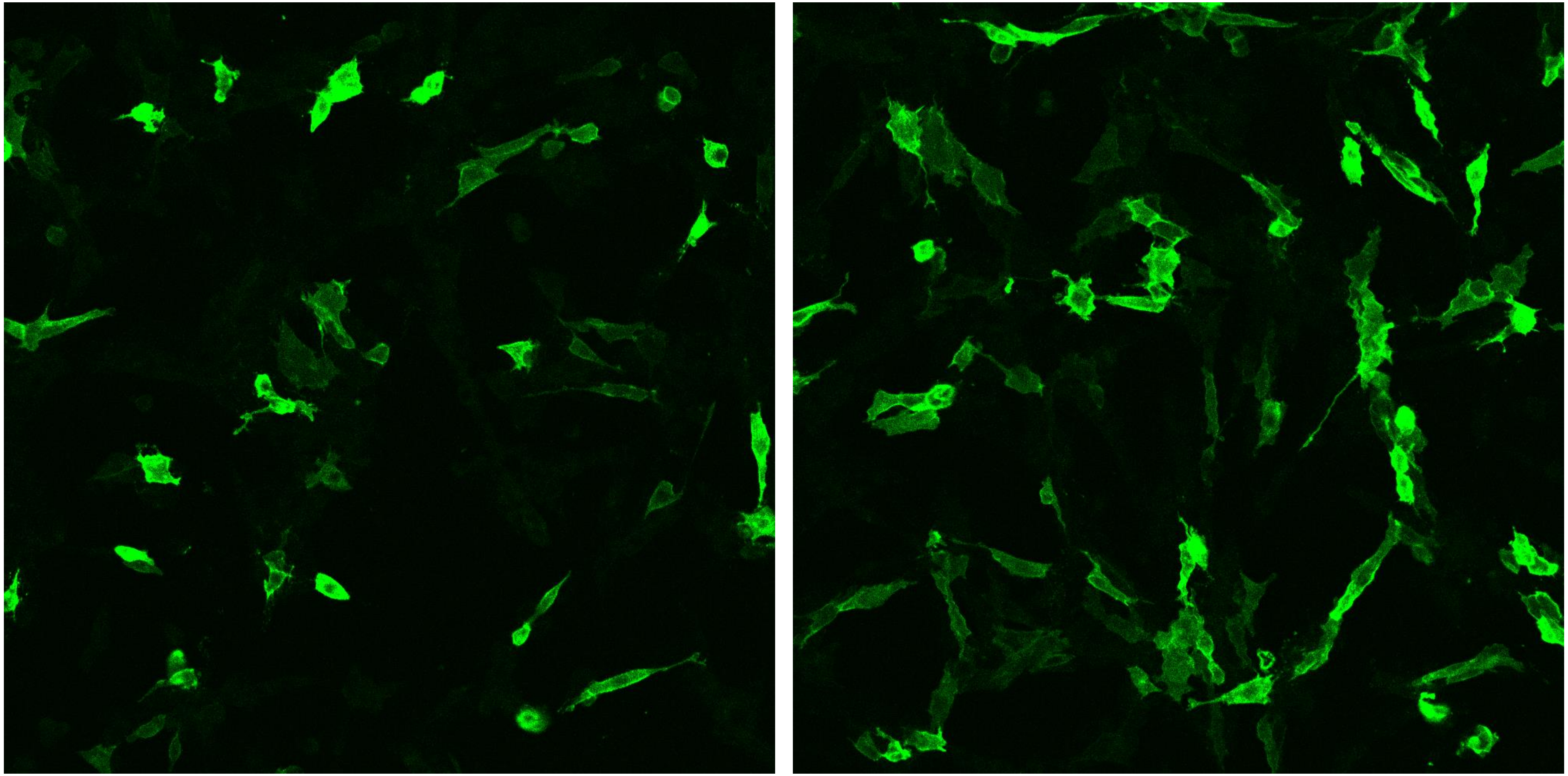

P180A + 40 μM YC-001

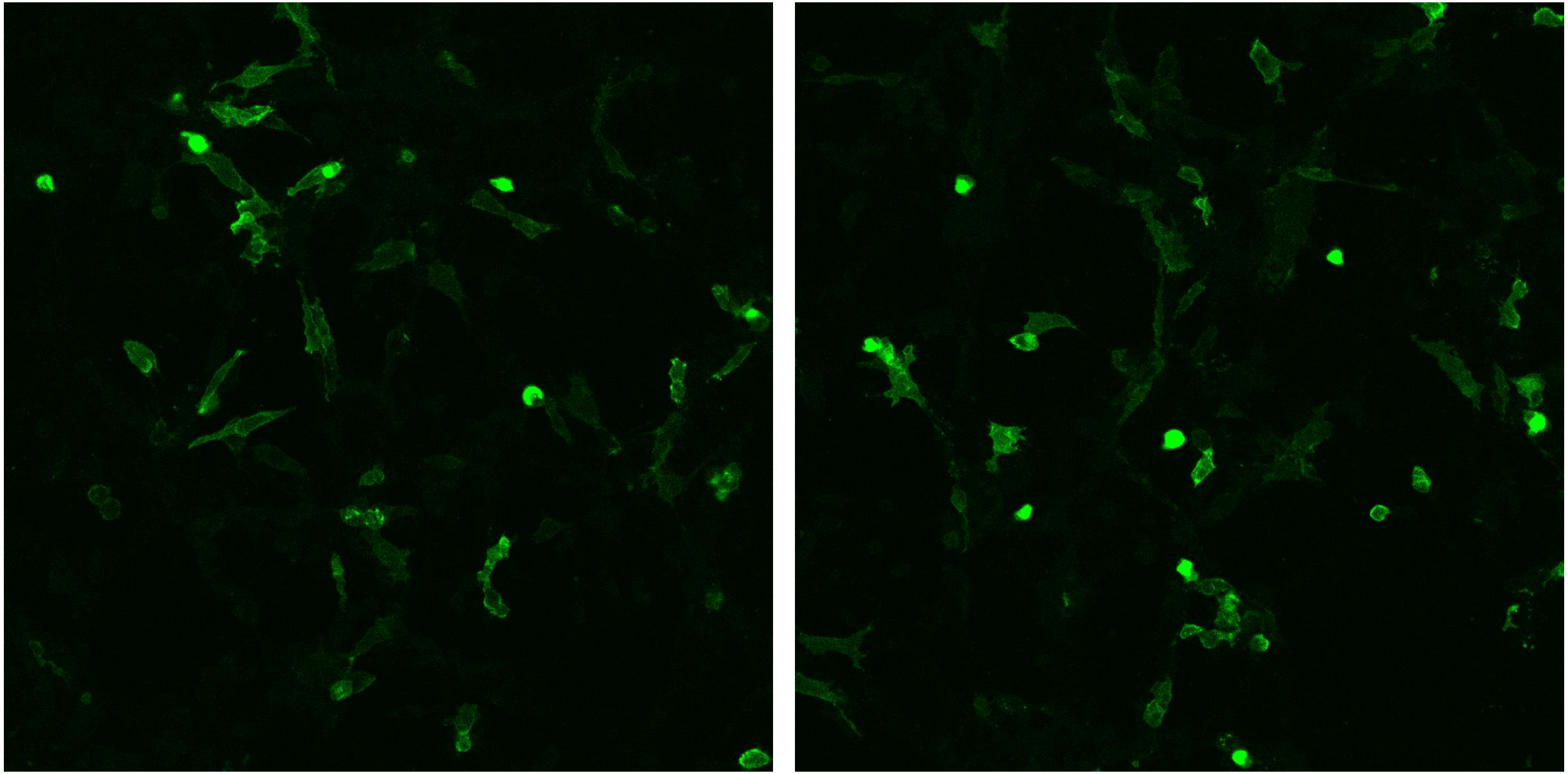

P180A + 20 μM F5257-0462

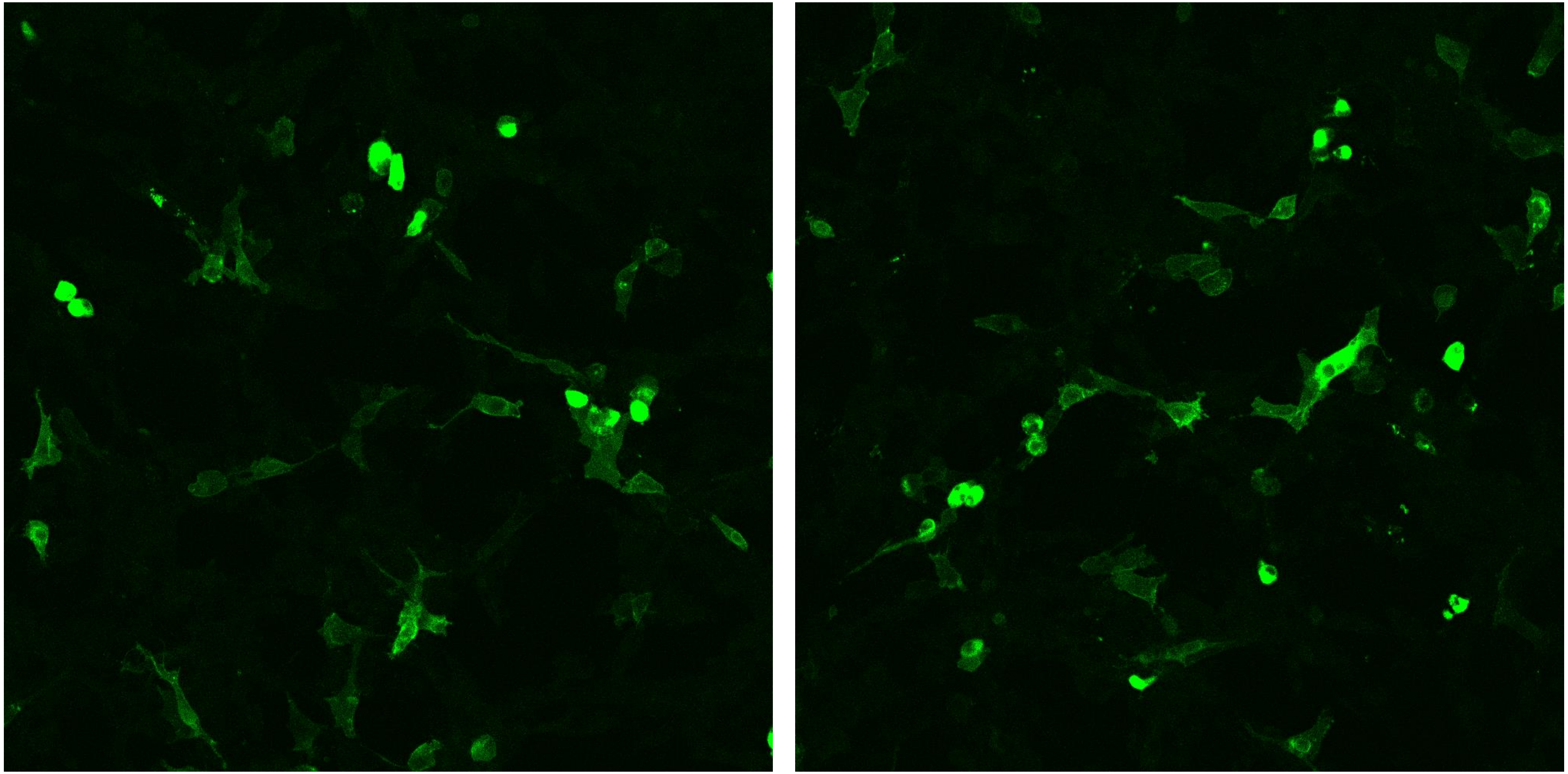

E181K + DMSO (0.1%)

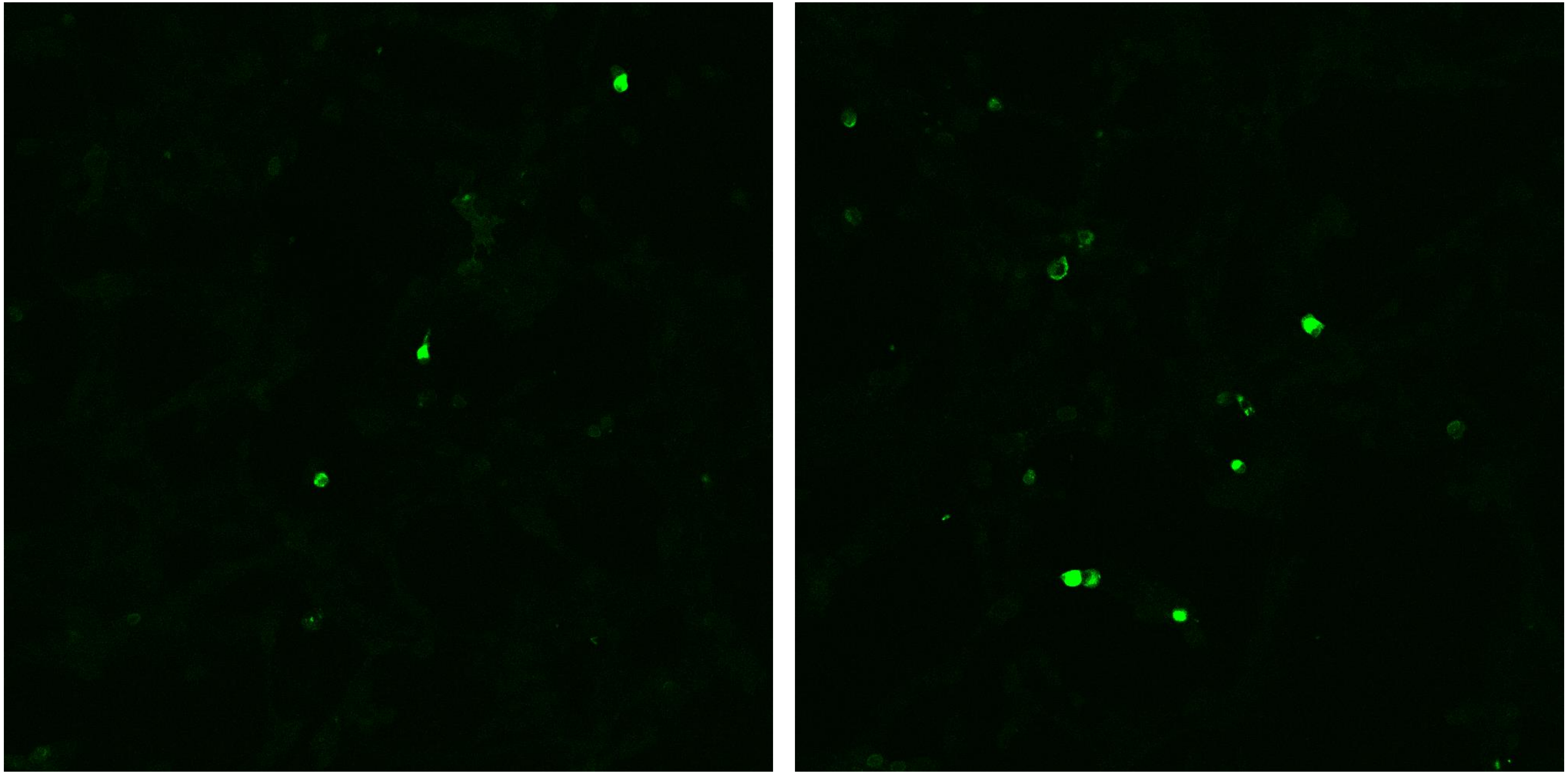

E181K + DMSO (0.1%)

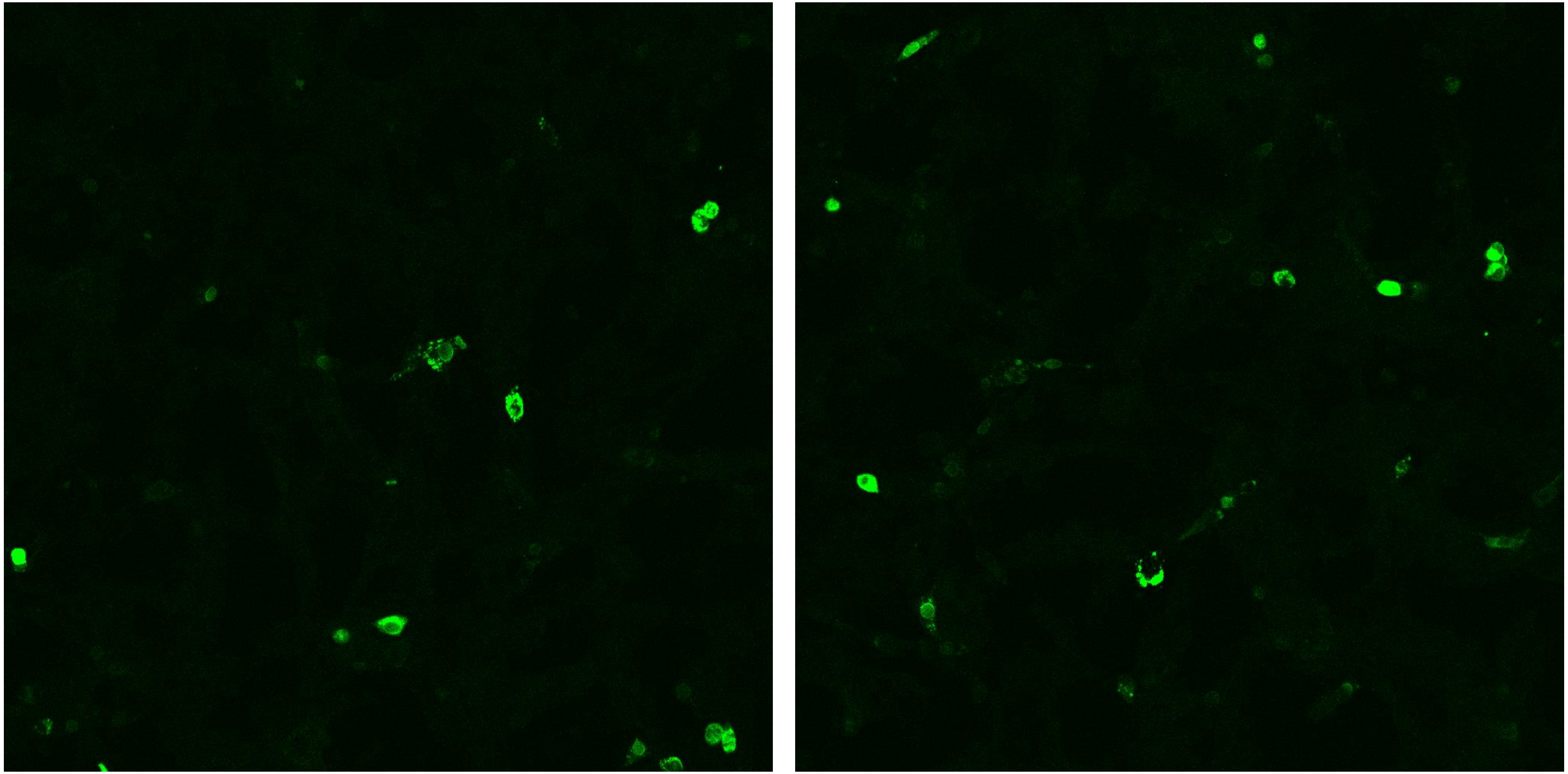

E181K + 5 μM 9-*cis*-retinal

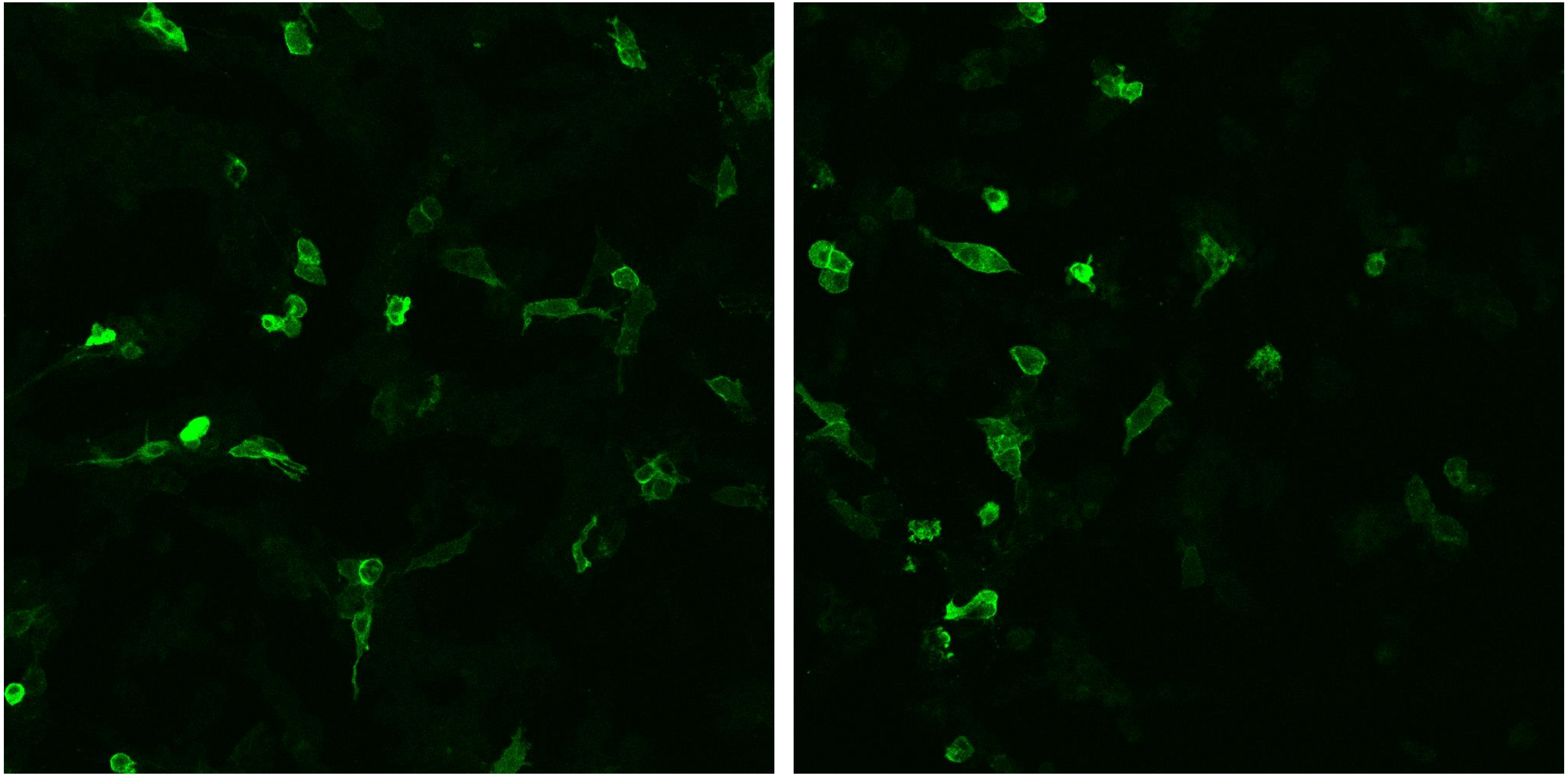

E181K + 40 μM YC-001

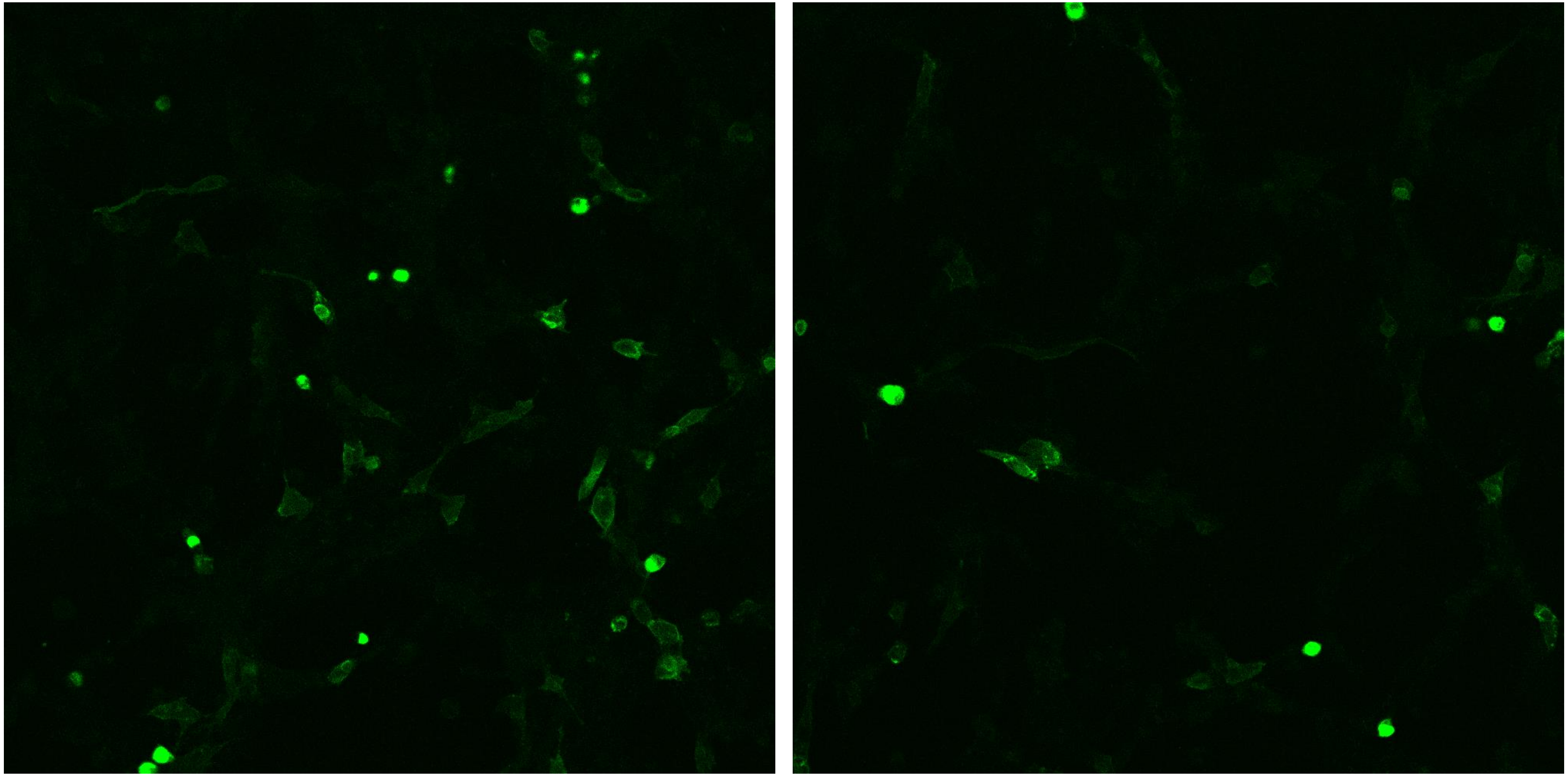

E181K + 20 μM F5257-0462

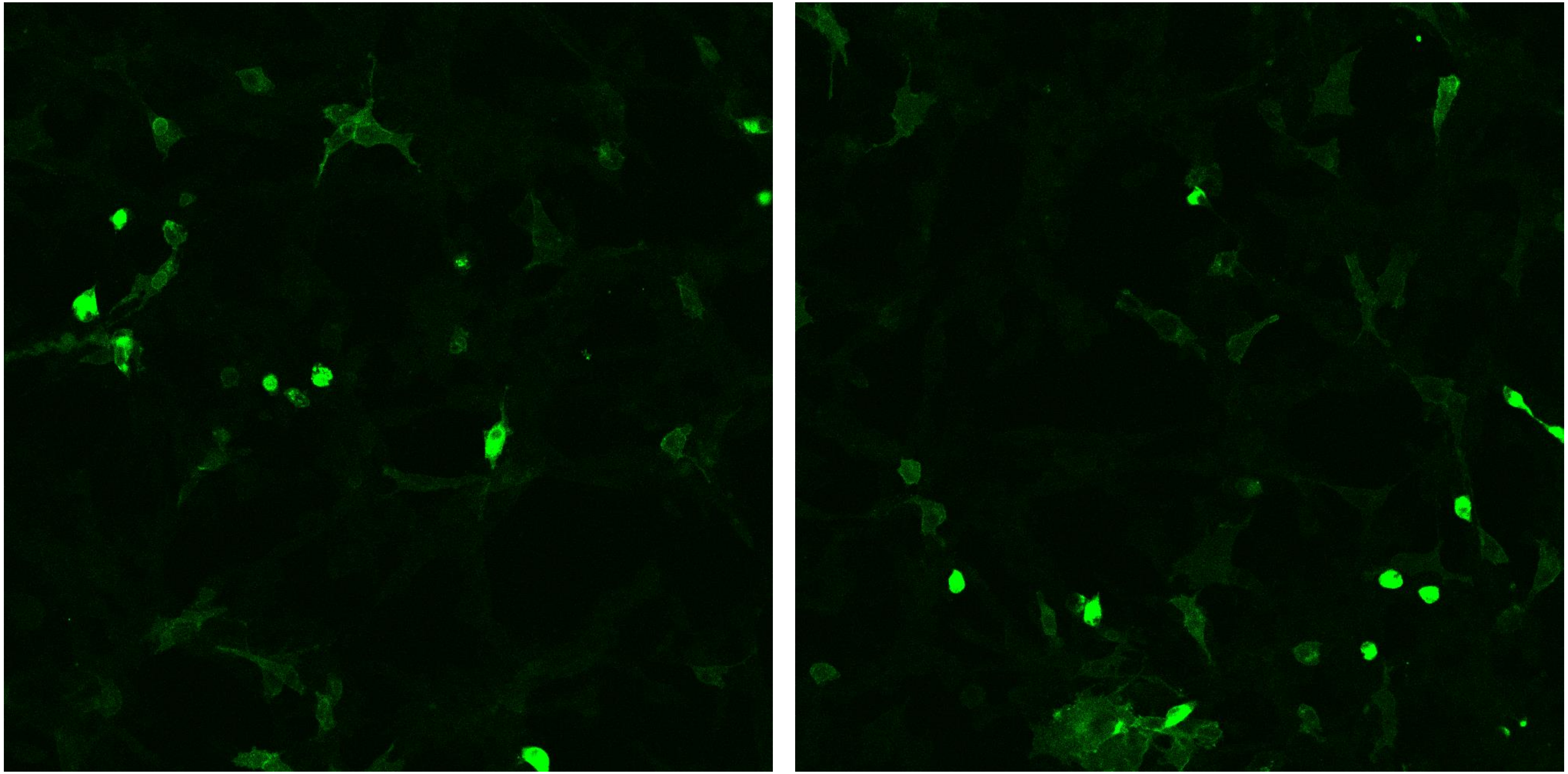

G182S + DMSO (0.1%)

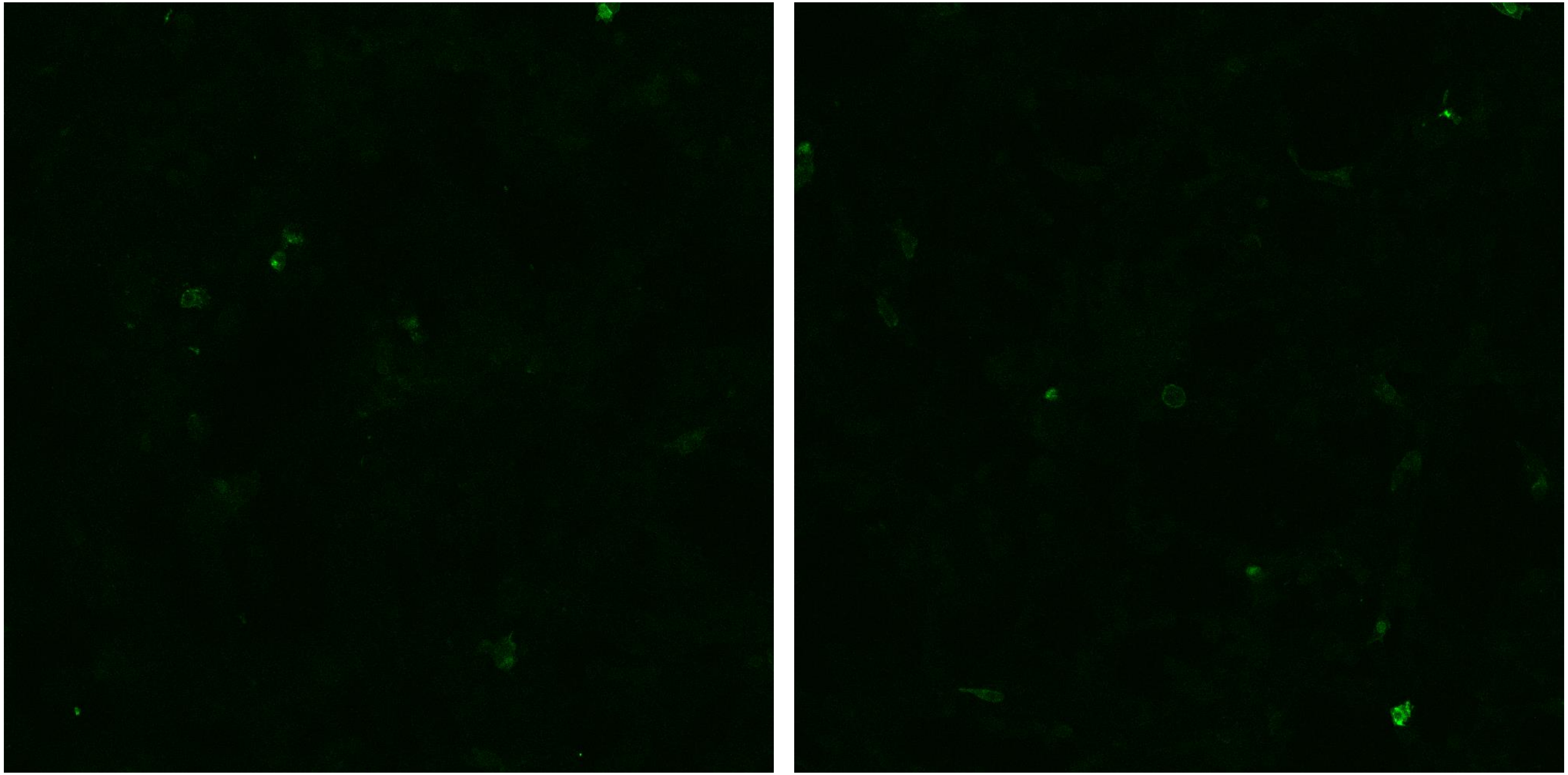

G182S + DMSO (0.1%)

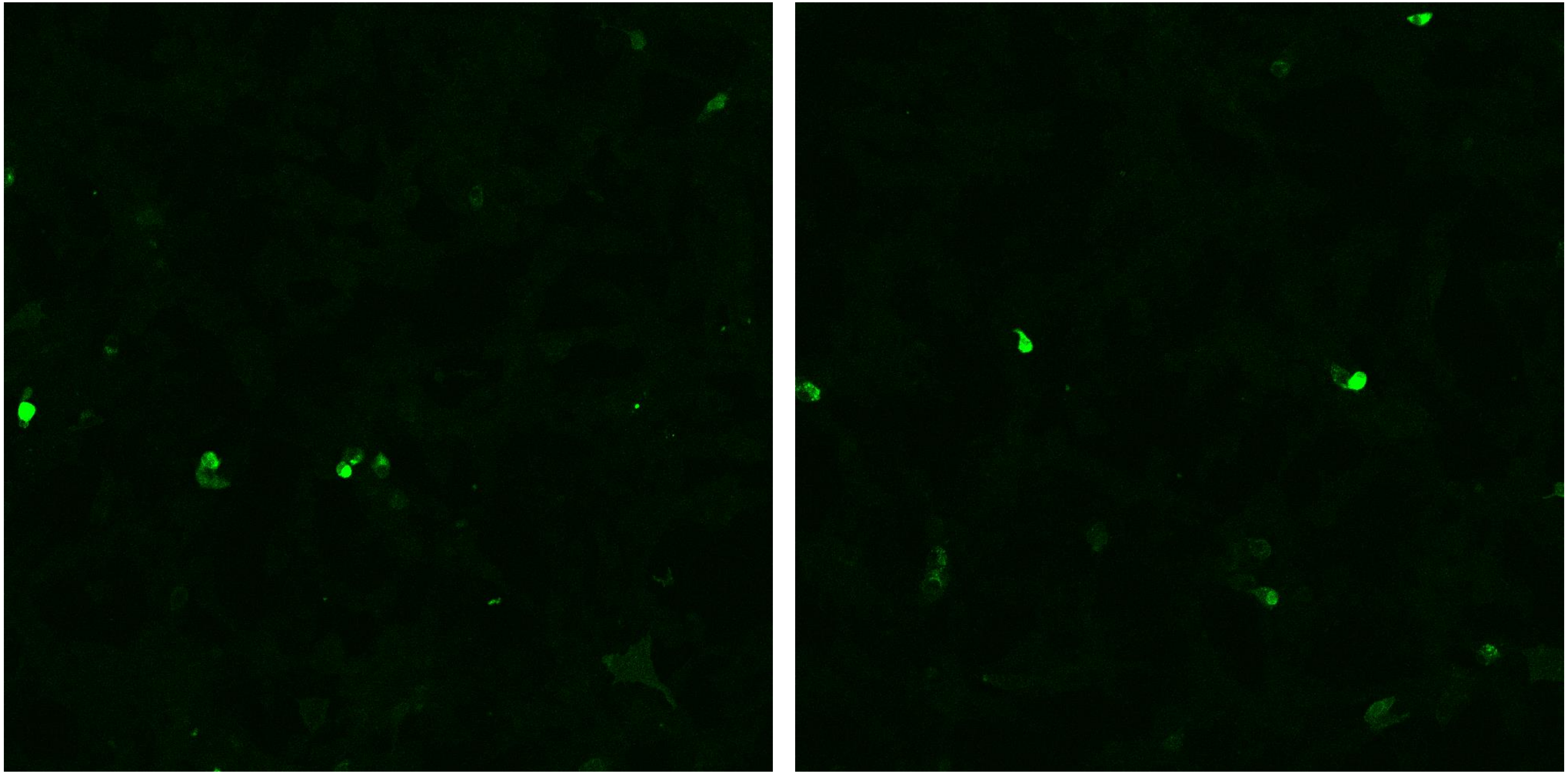

G182S + 5 μM 9-*cis*-retinal

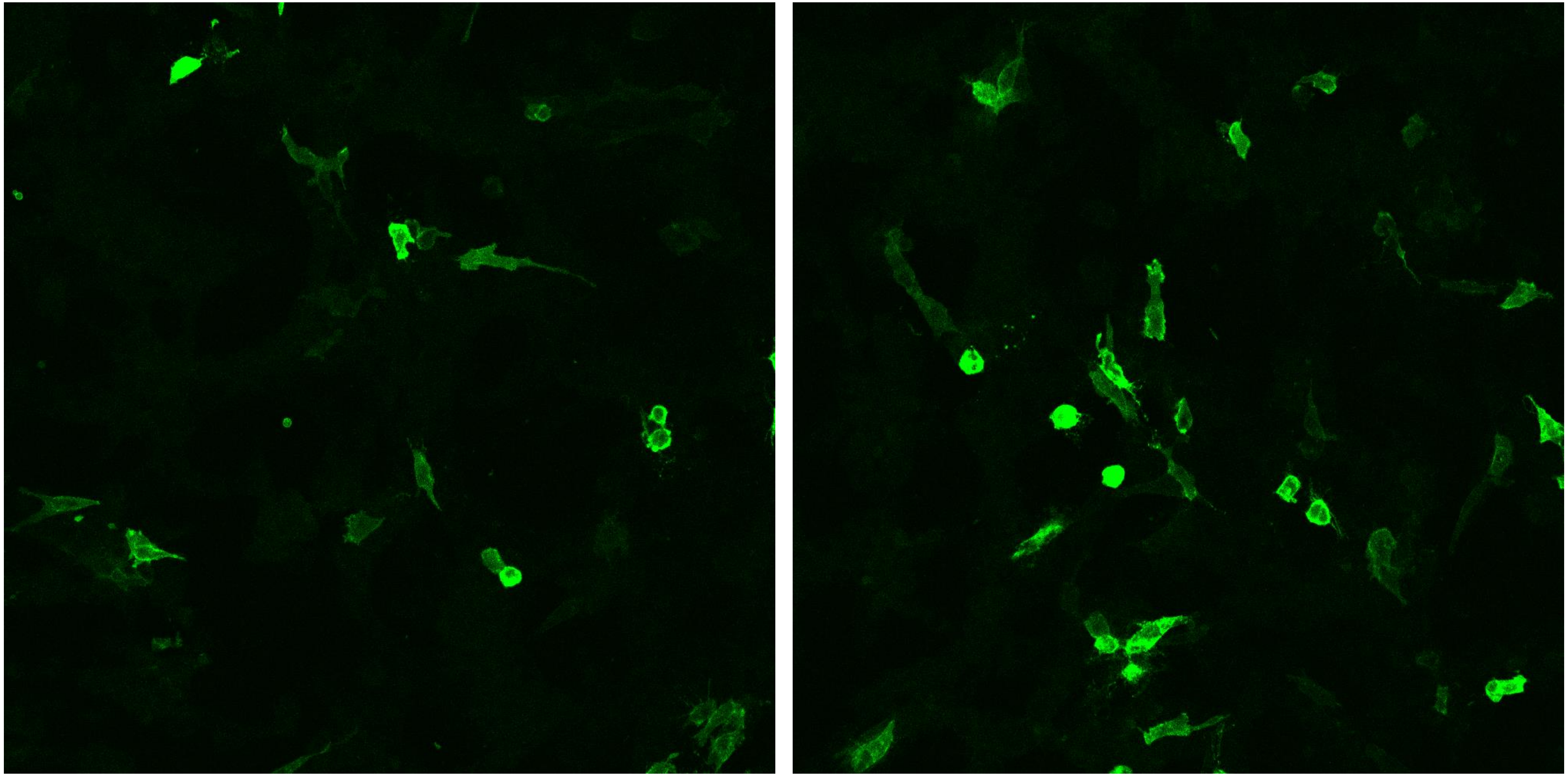

G182S + 40 μM YC-001

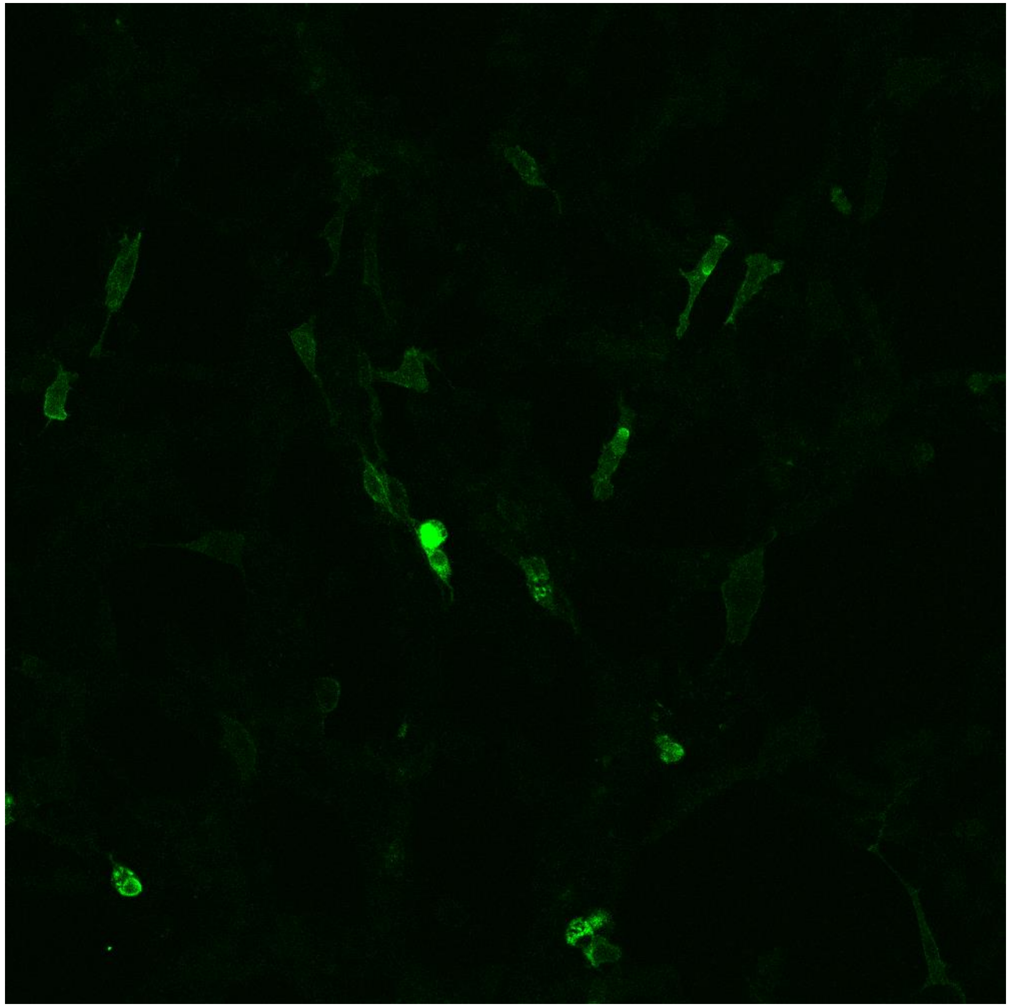

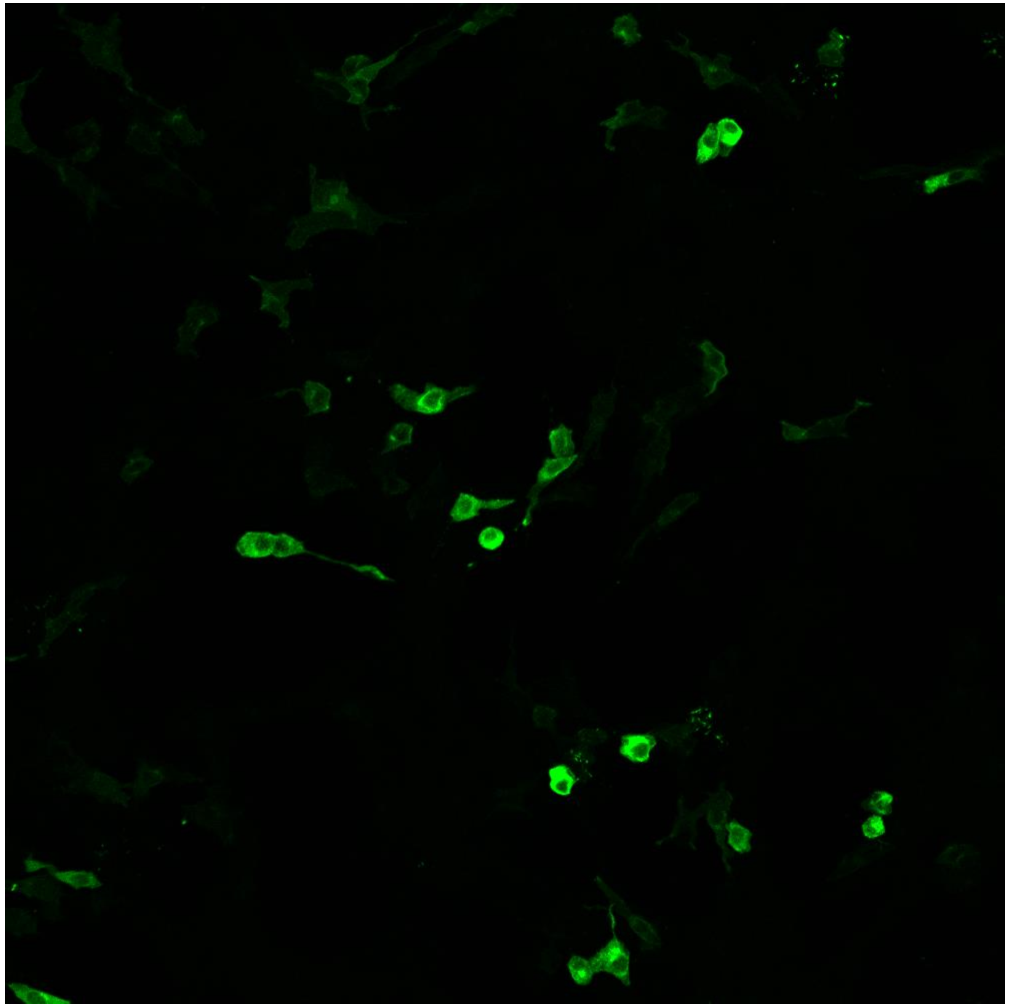

G182S + 20 μM F5257-0462

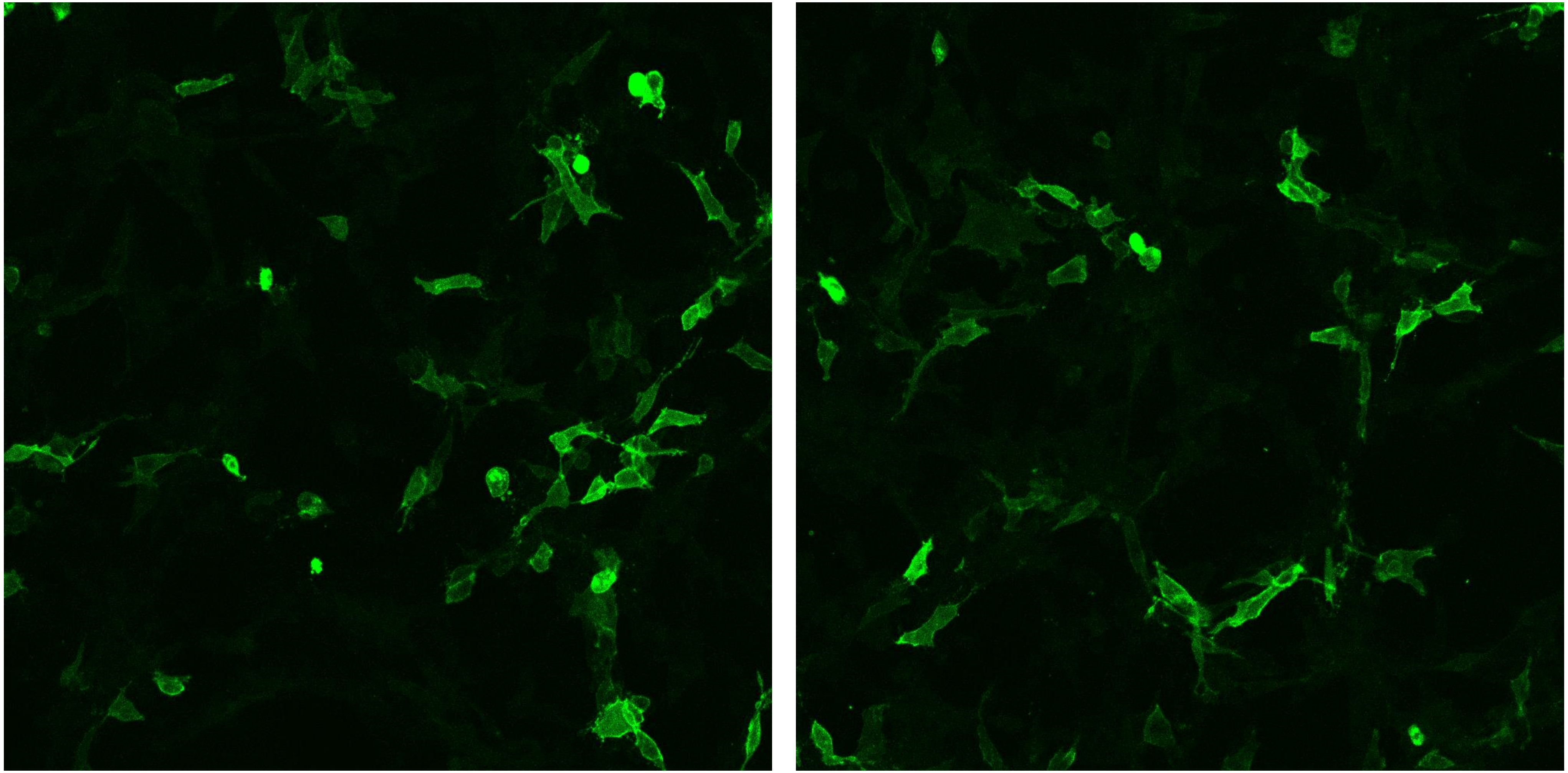

Q184P + DMSO (0.1%)

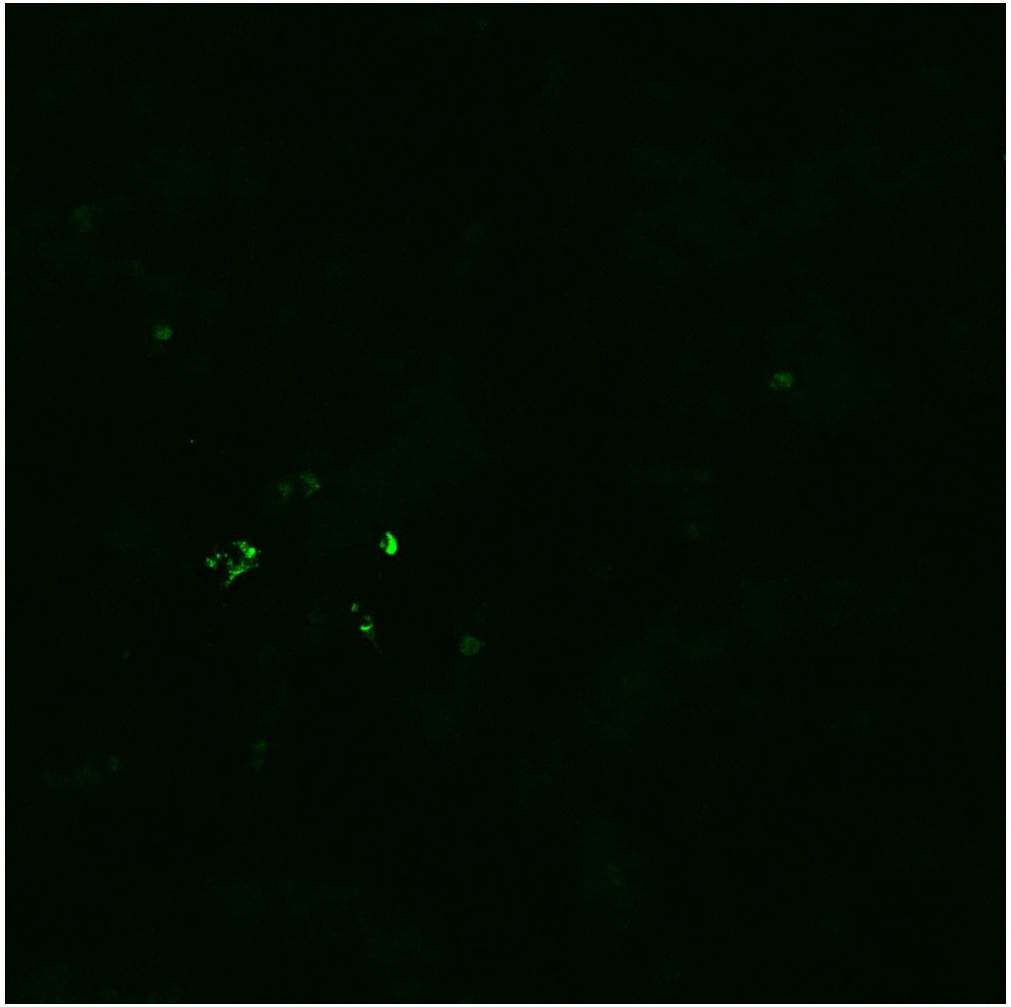

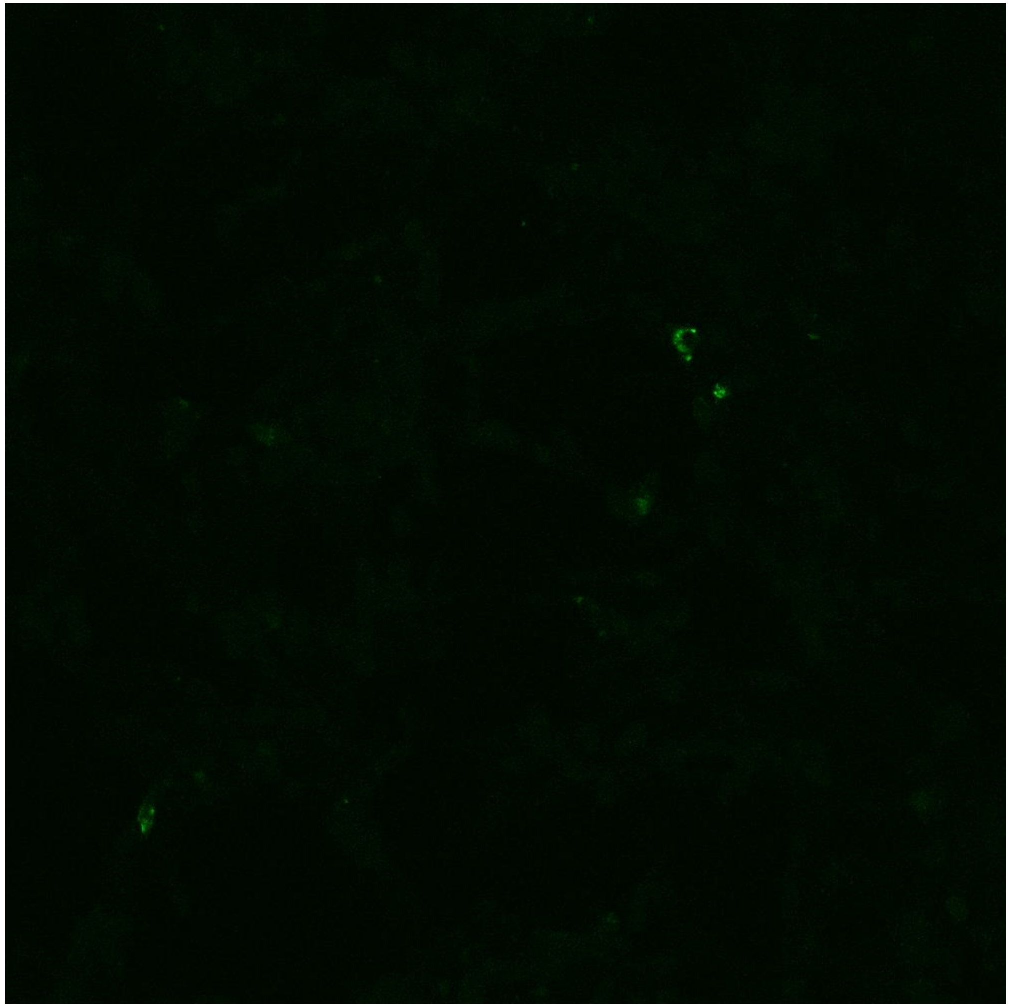

Q184P + DMSO (0.1%)

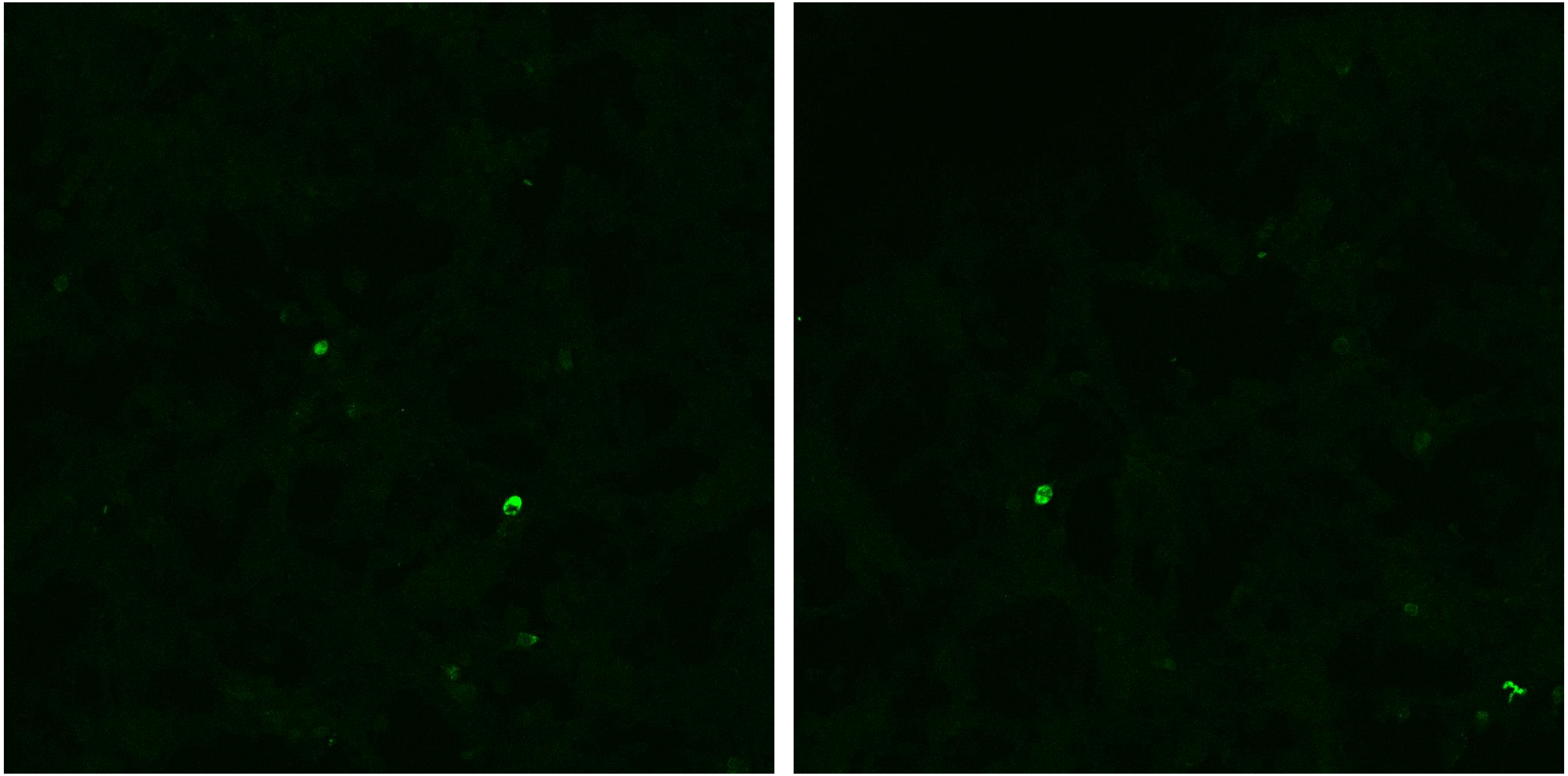

Q184P + 5 μM 9-*cis*-retinal

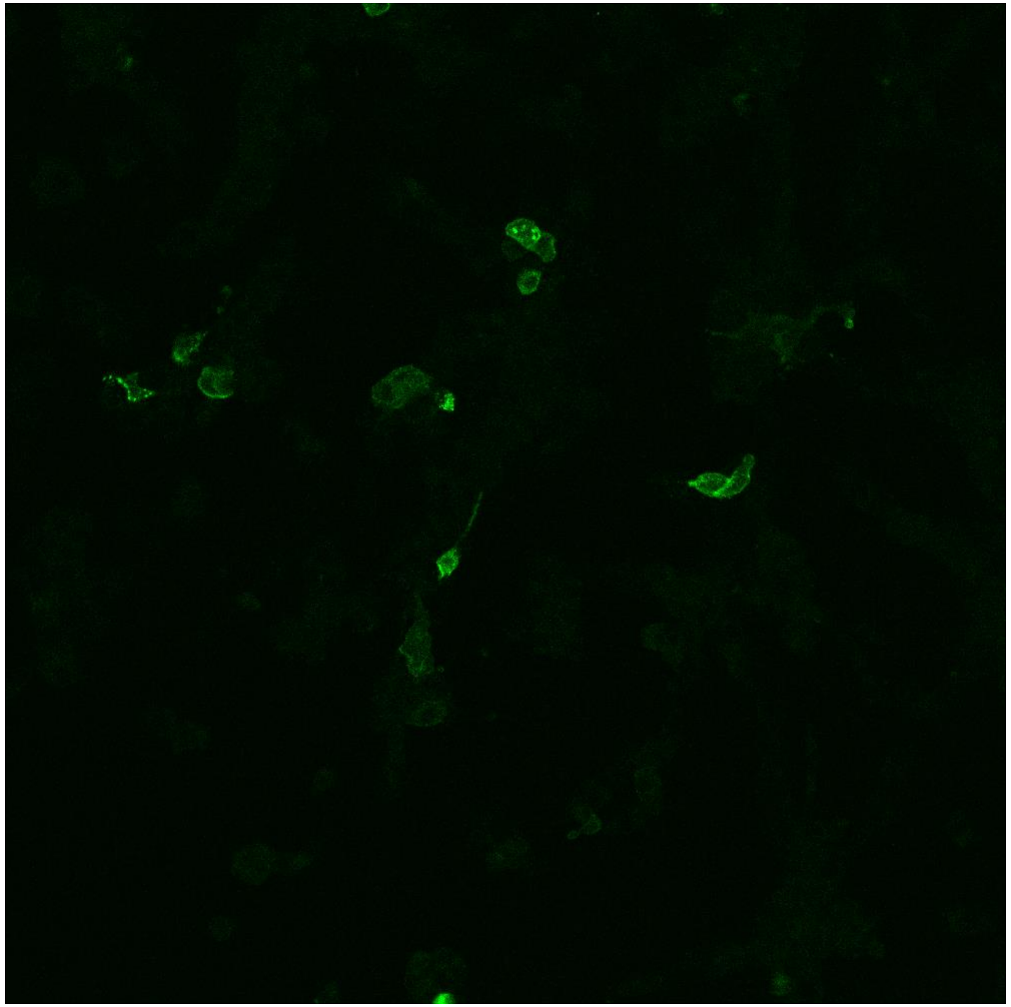

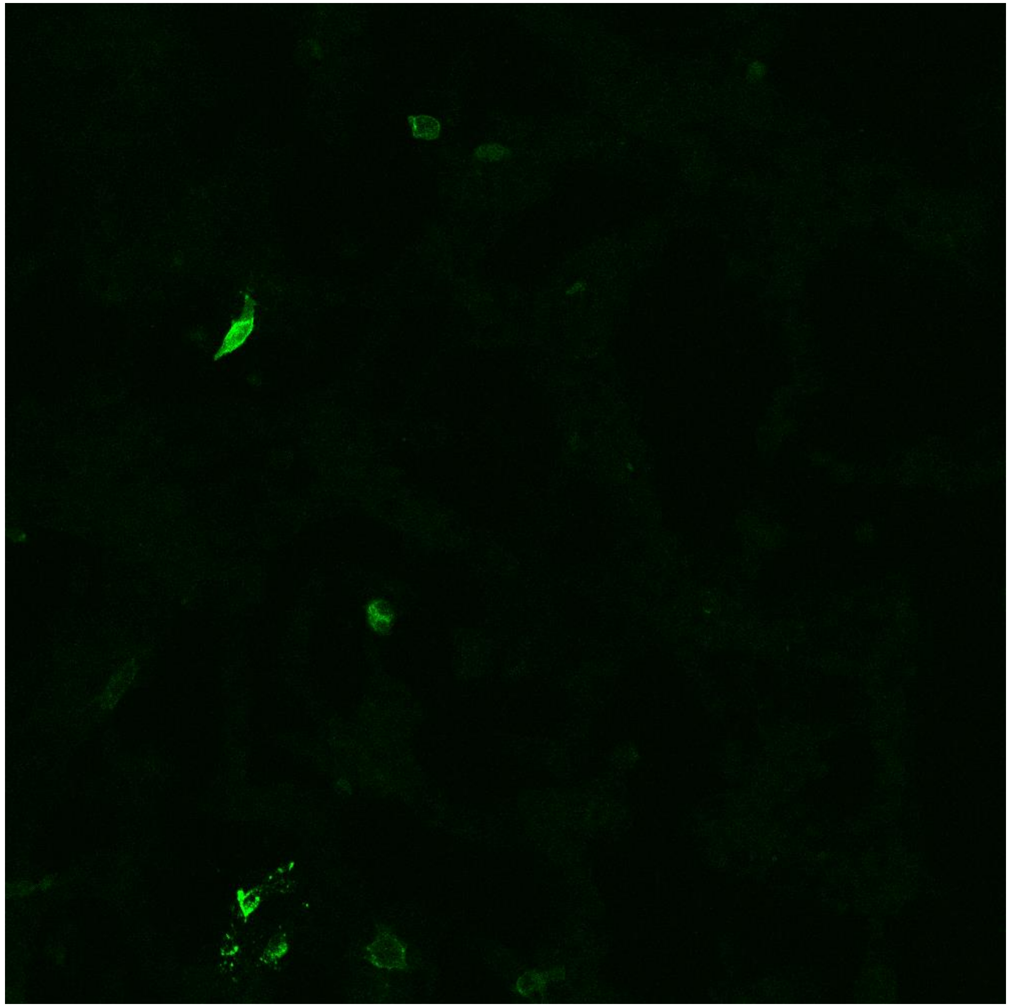

Q184P + 40 µM YC-001

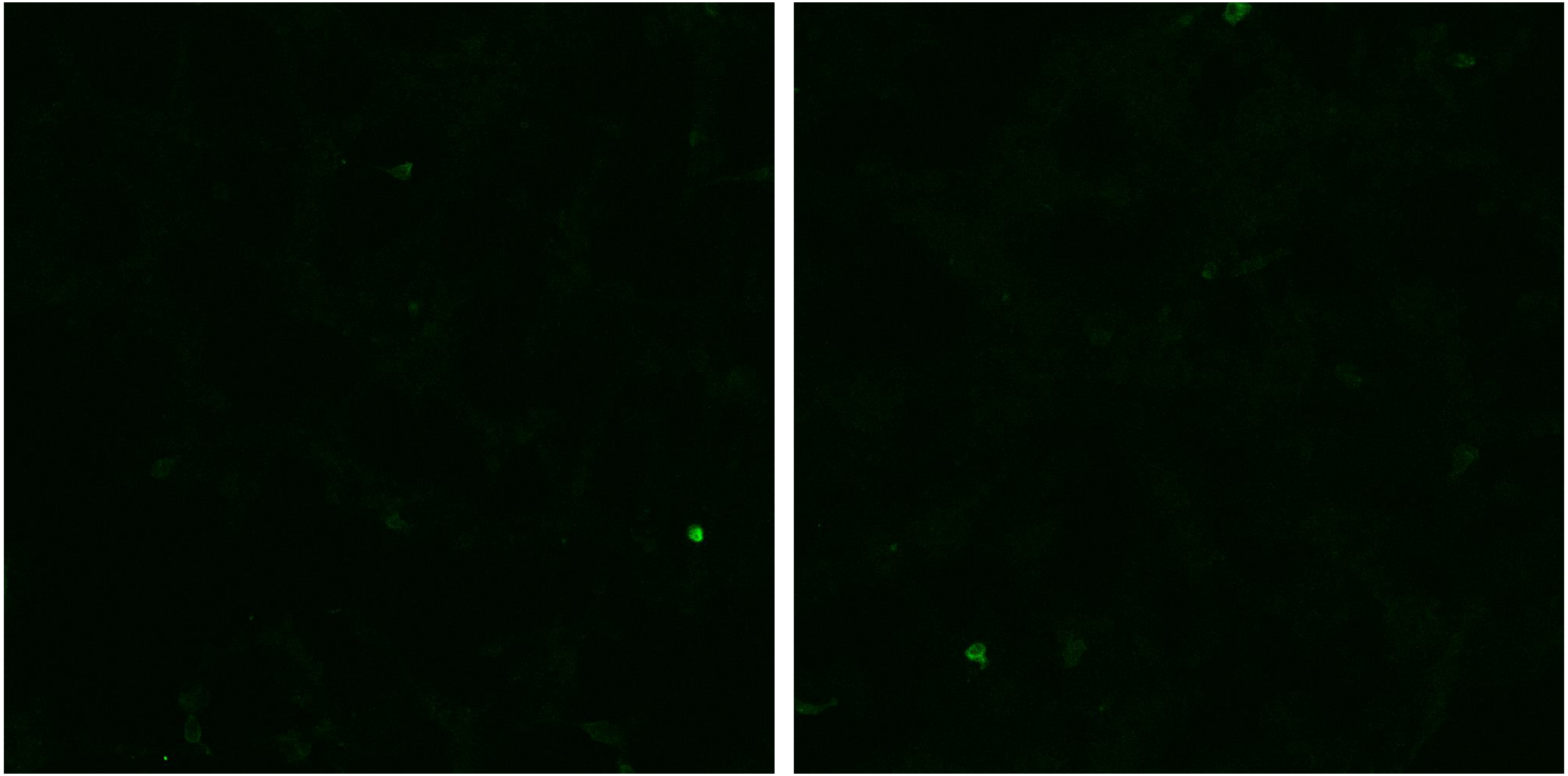

Q184P + 20 μM F5257-0462

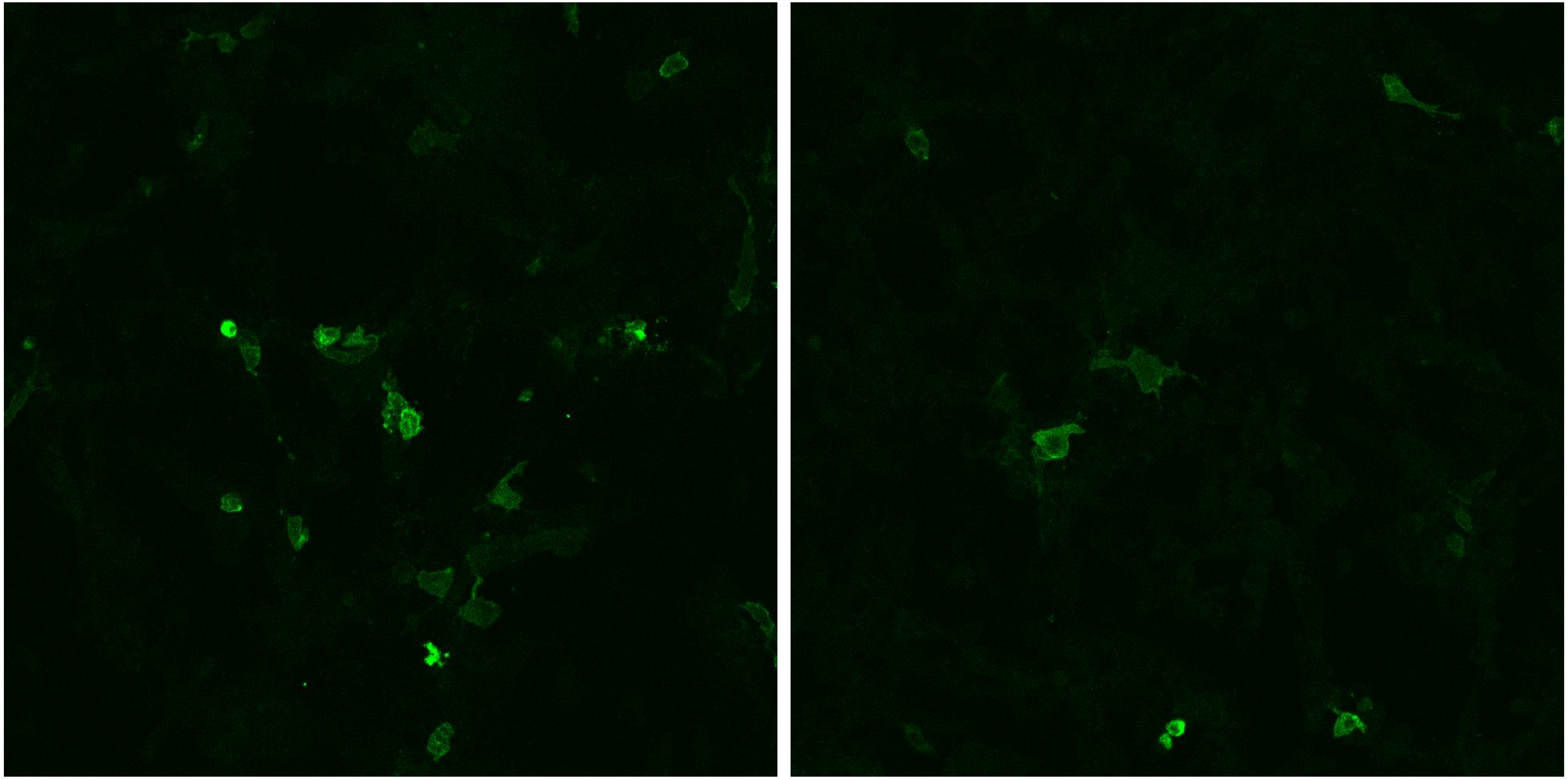

S186P + DMSO (0.1%)

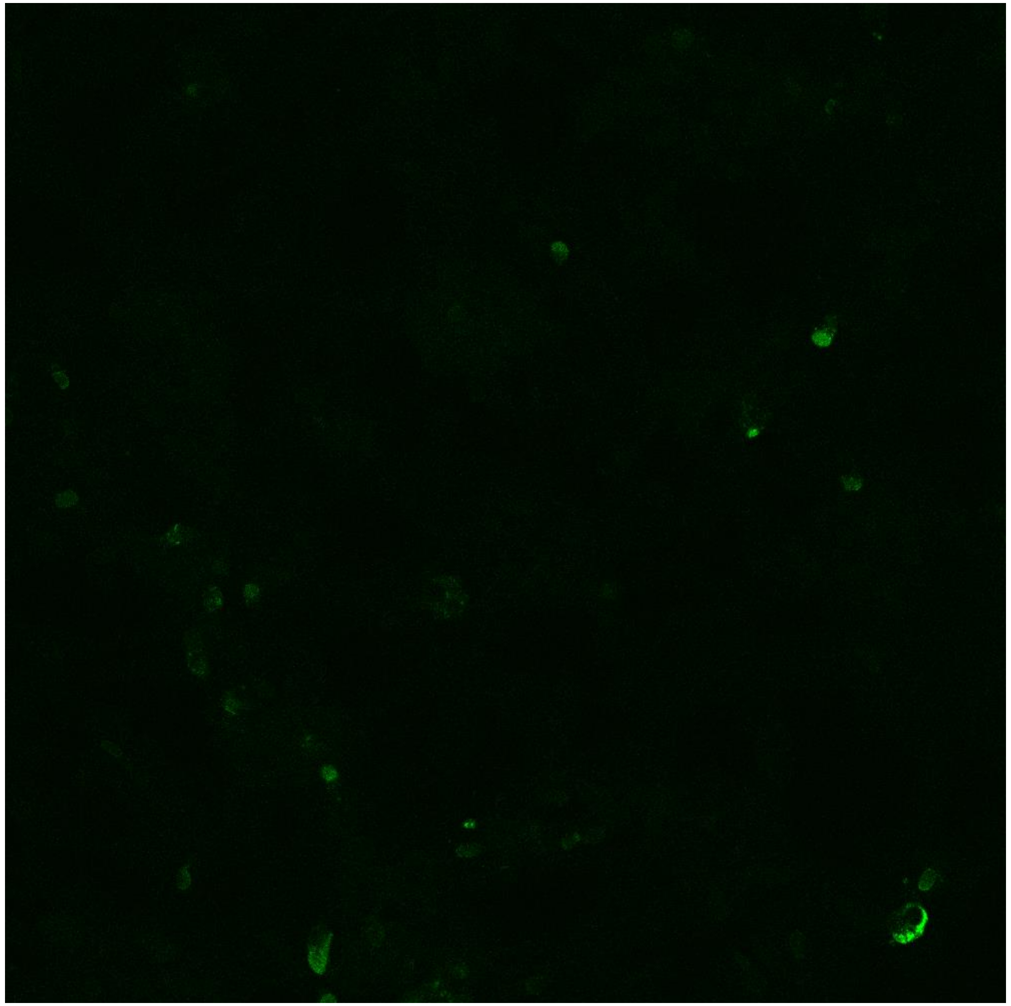

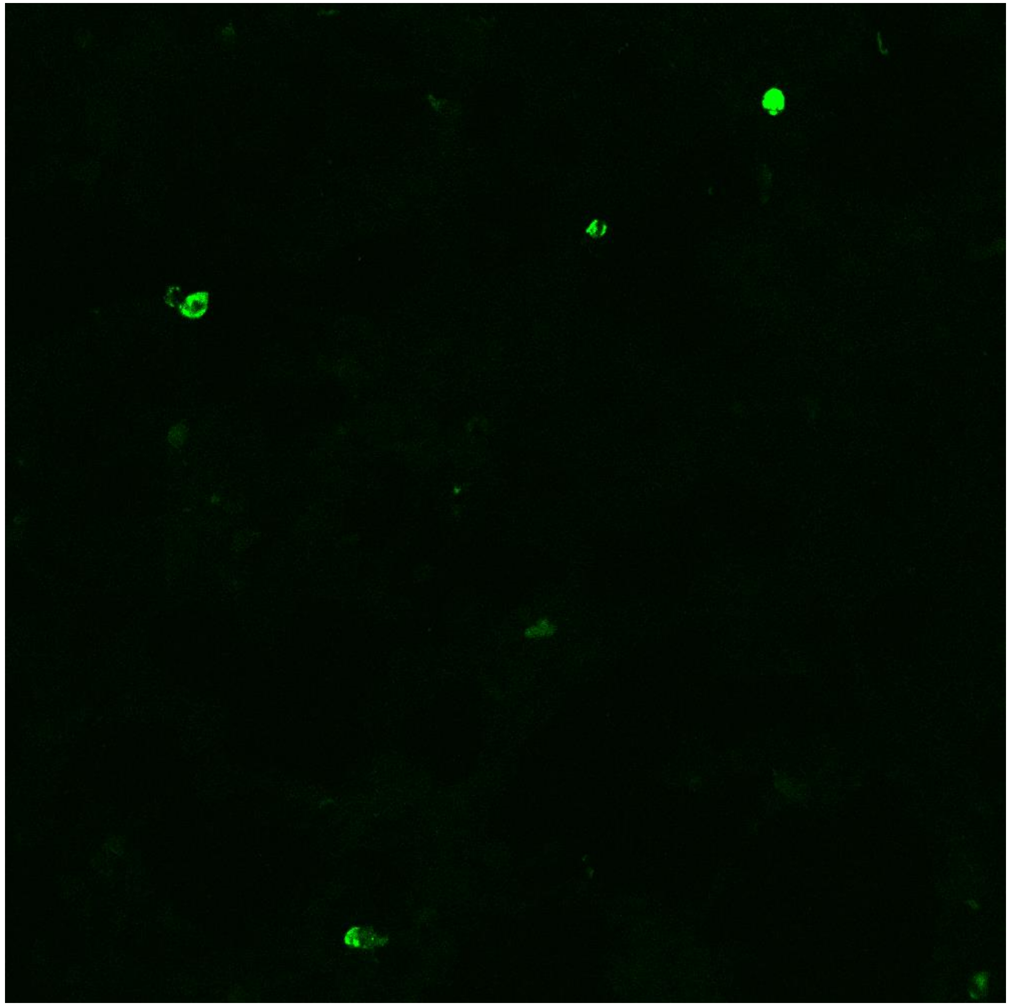

S186P + DMSO (0.1%)

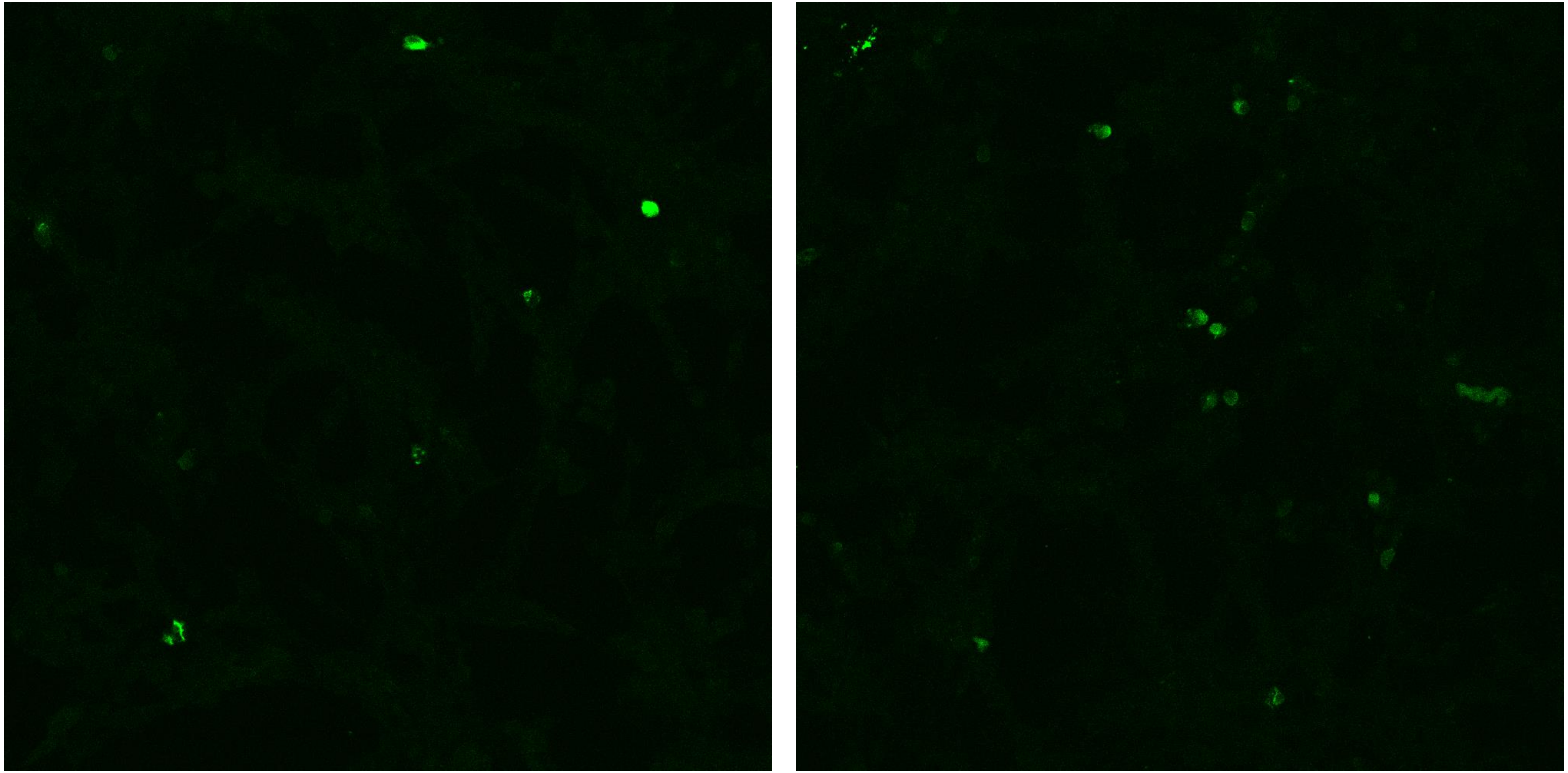

S186P + 5 µM 9-*cis*-retinal

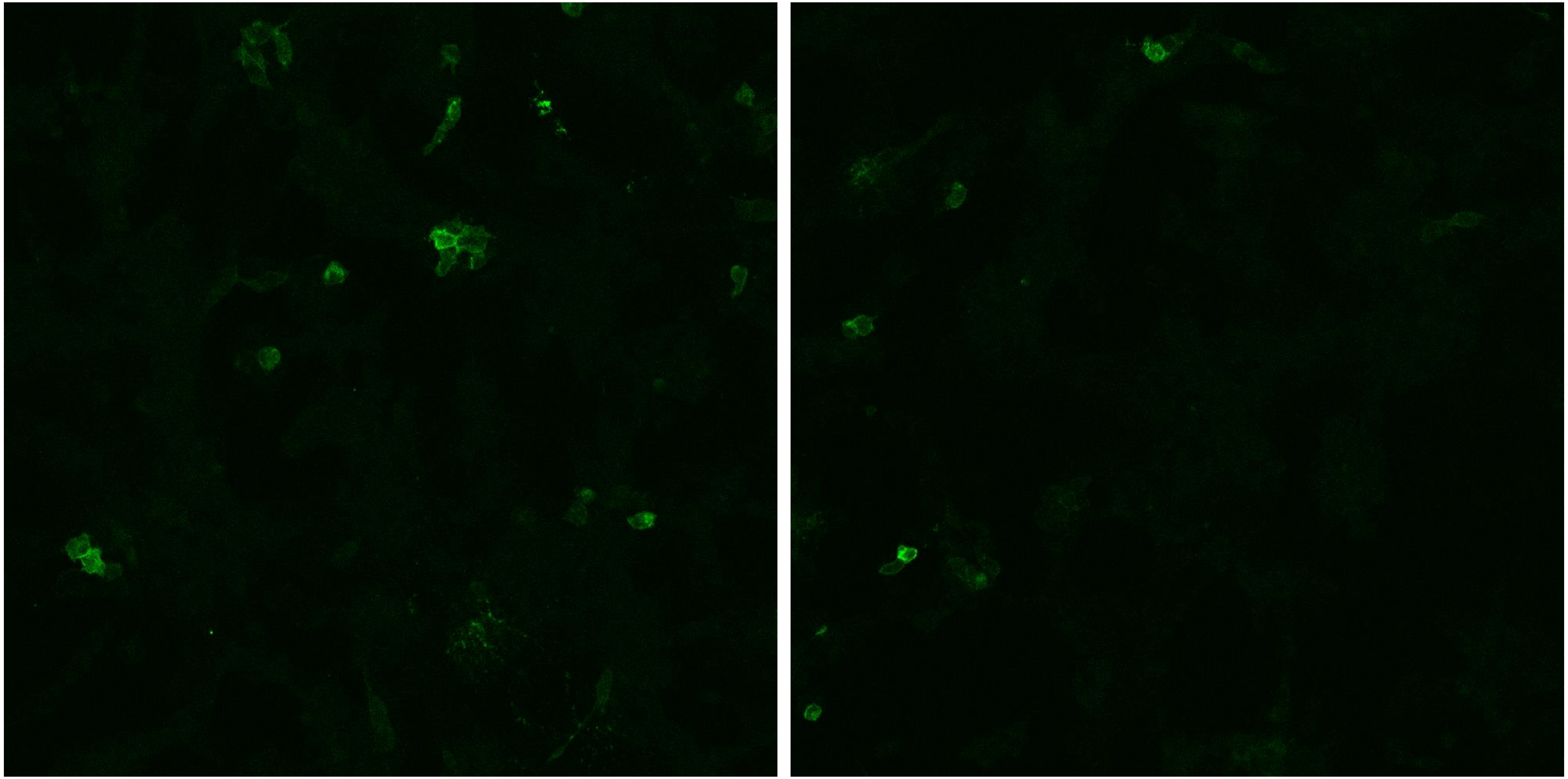

S186P + 40 μM YC-001

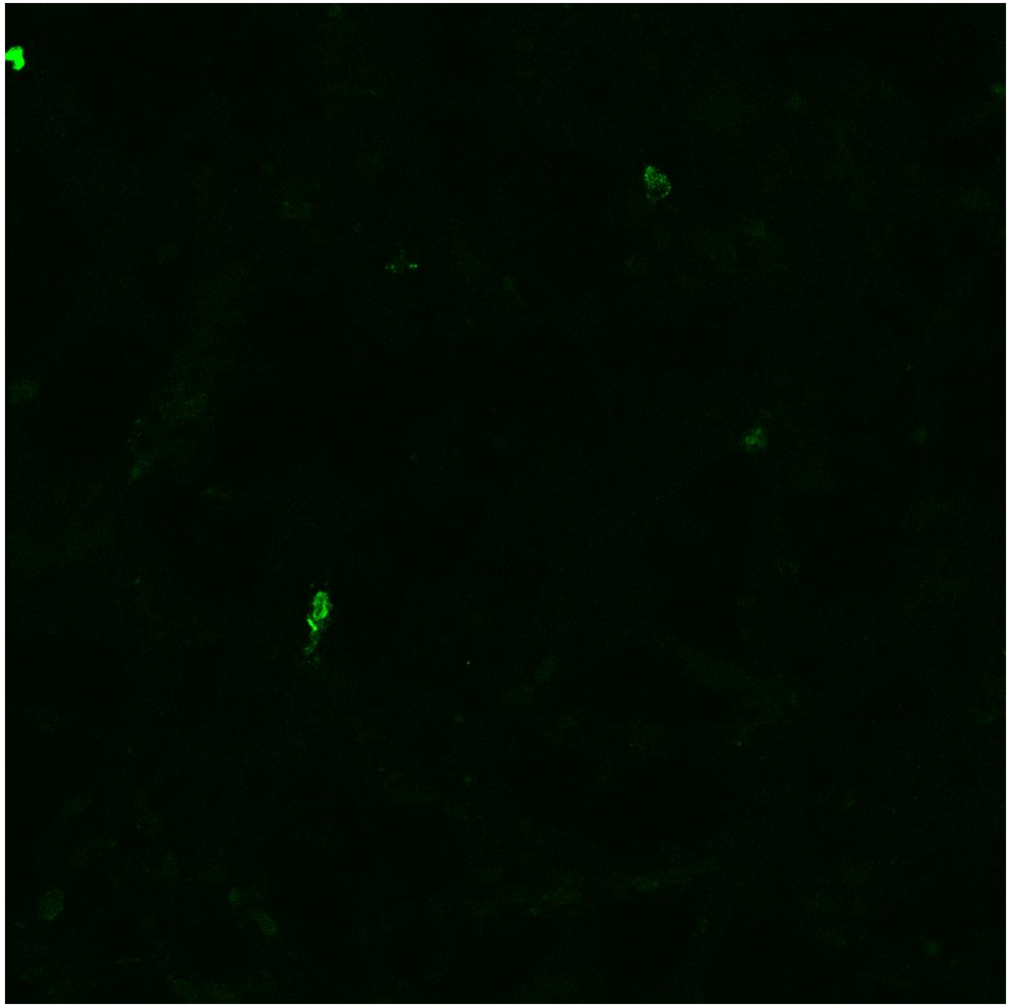

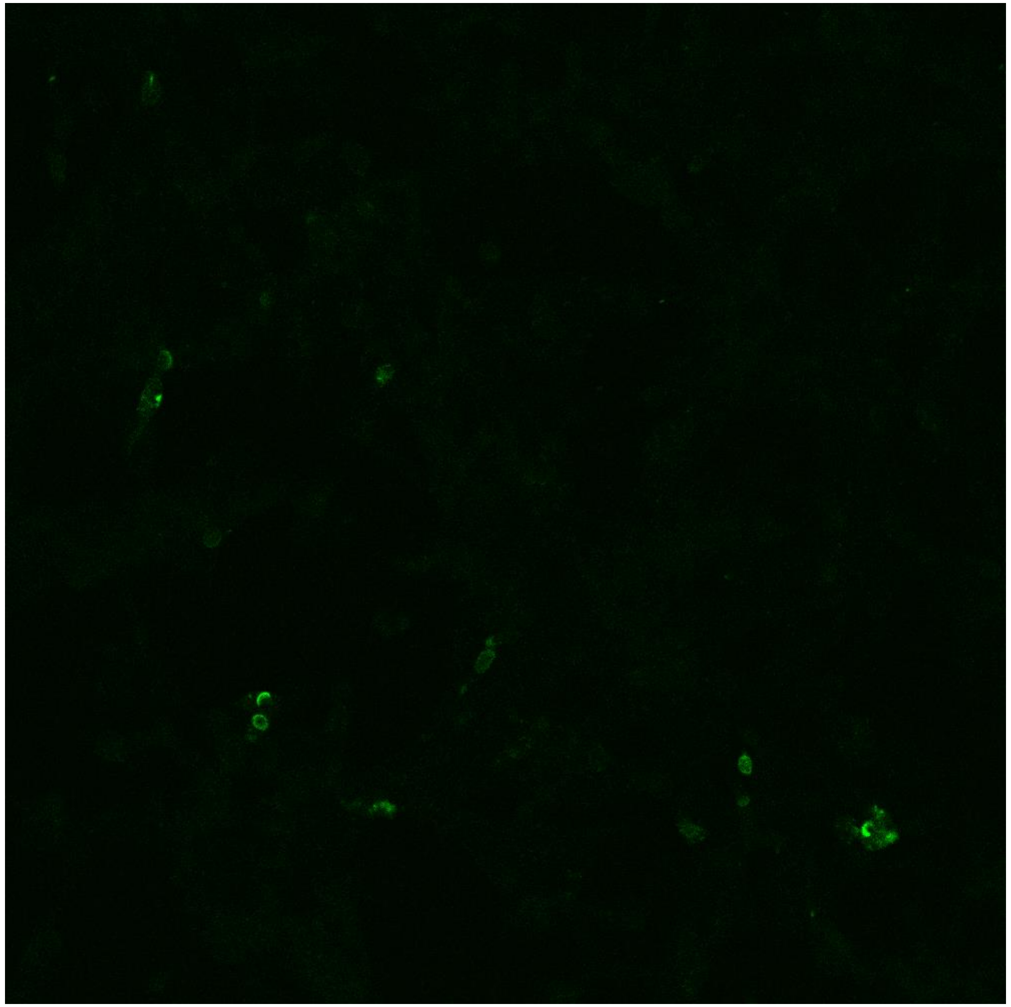

S186P + 20 μM F5257-0462

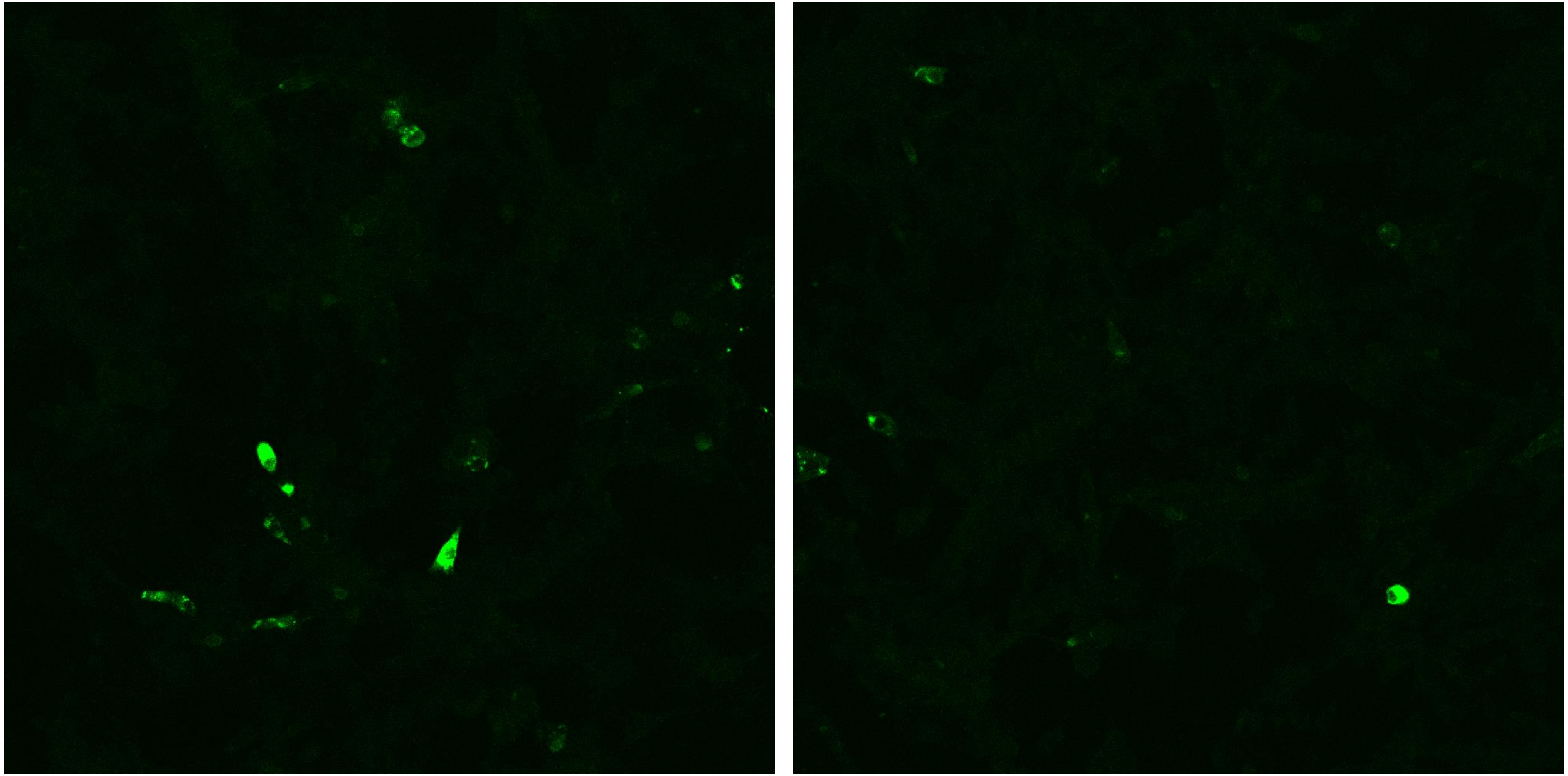

S186W + DMSO (0.1%)

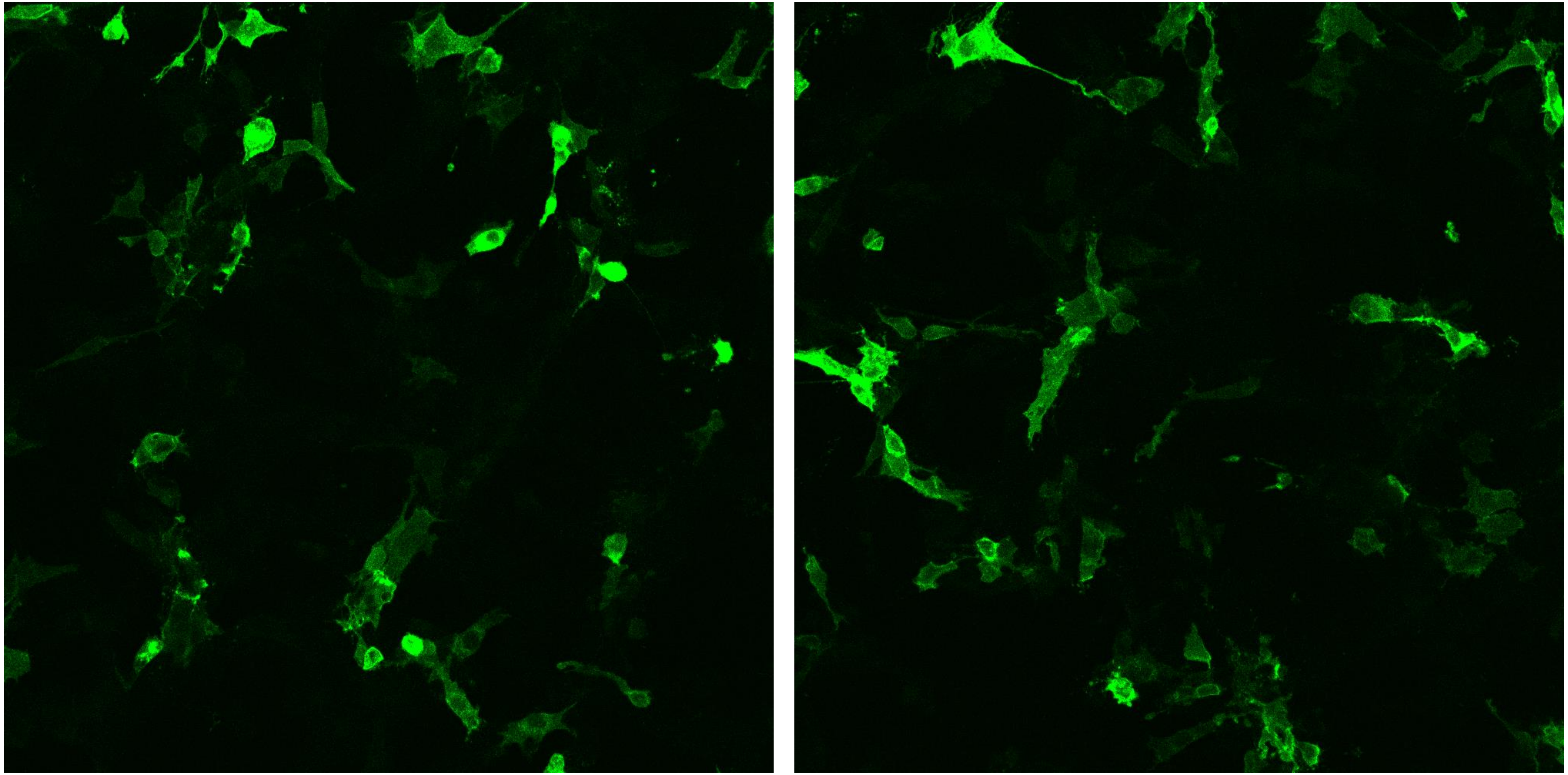

S186W + DMSO (0.1%)

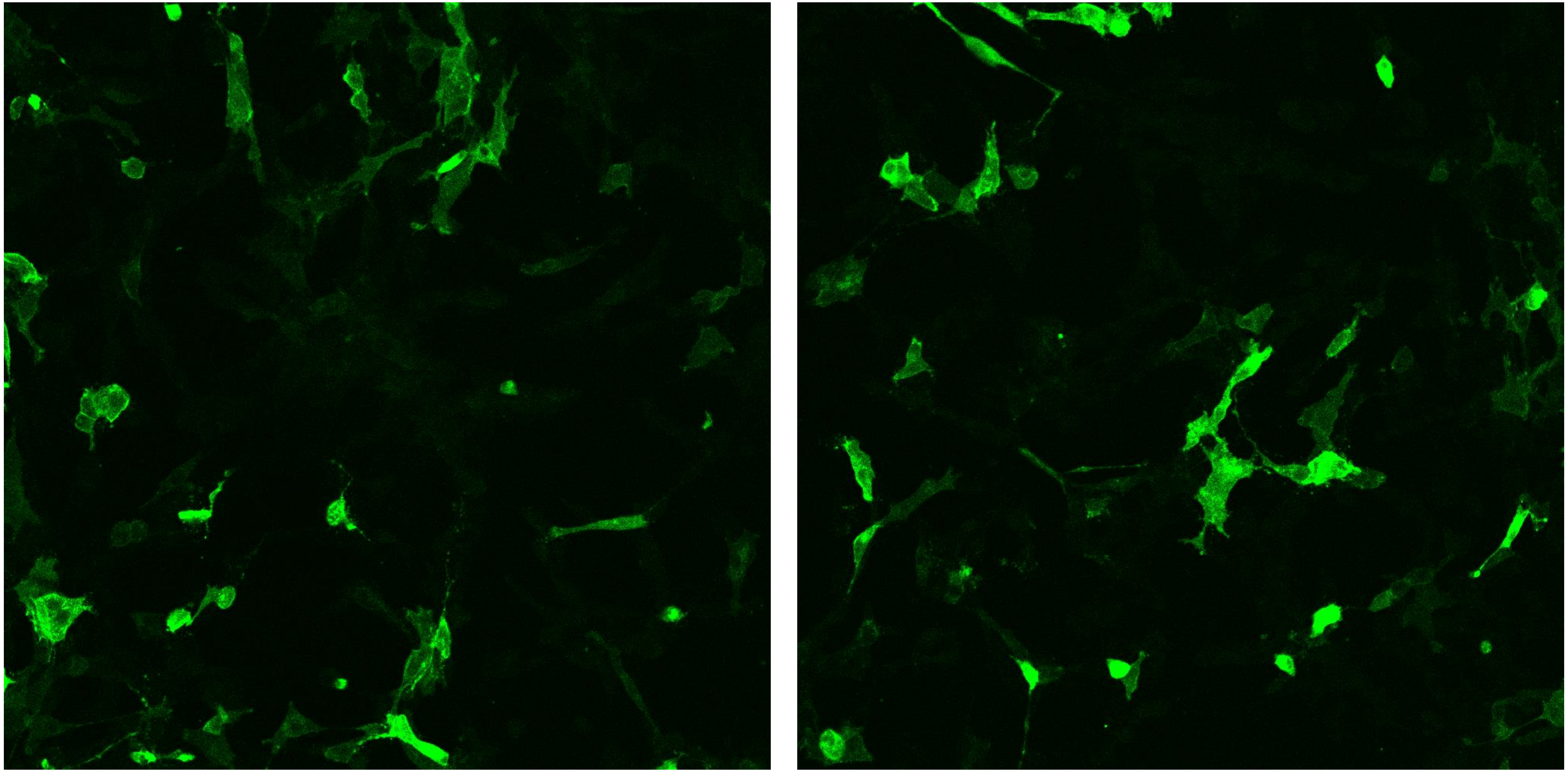

S186W + 5 µM 9-*cis*-retinal

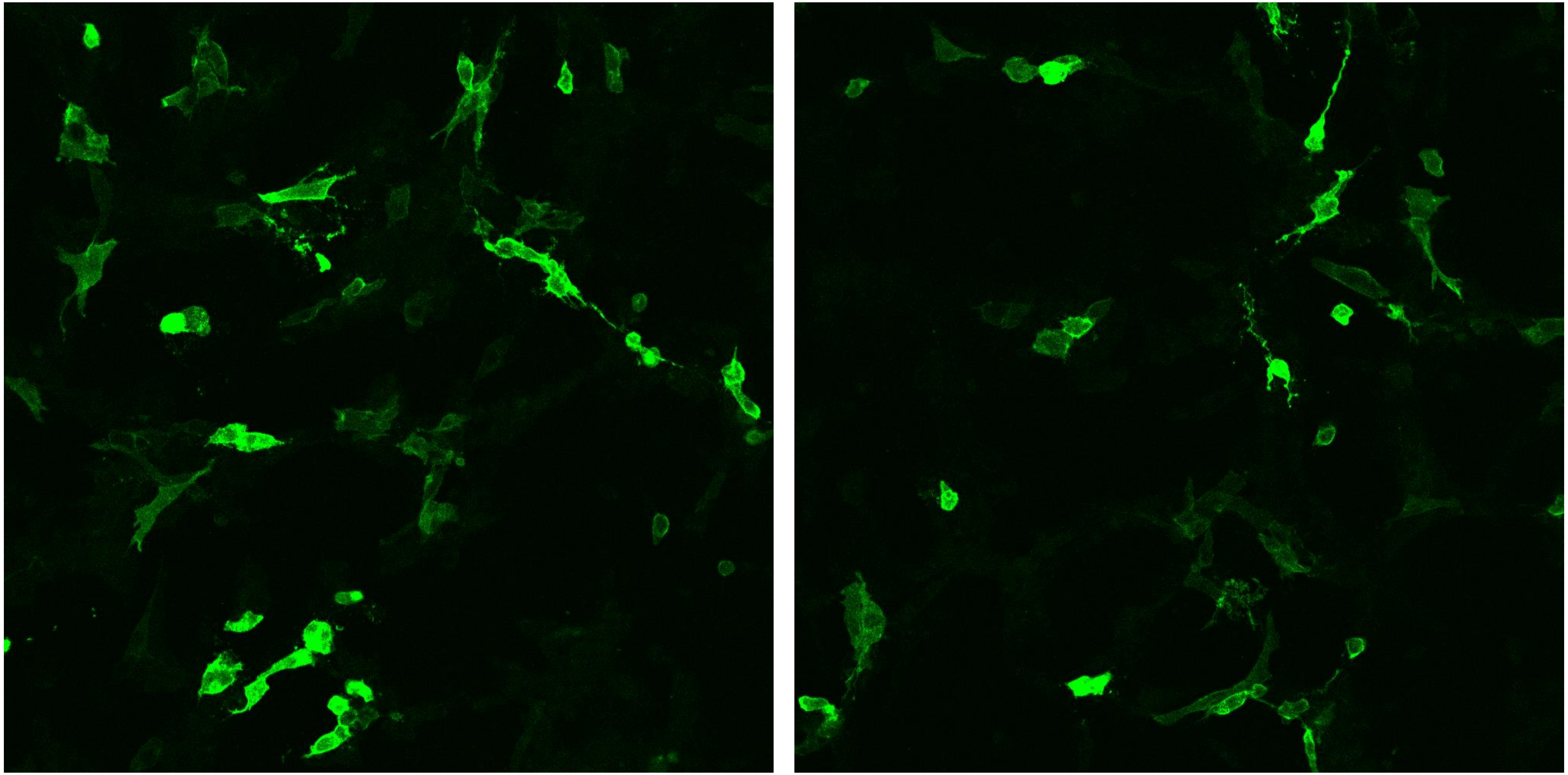

S186W + 40 µM YC-001

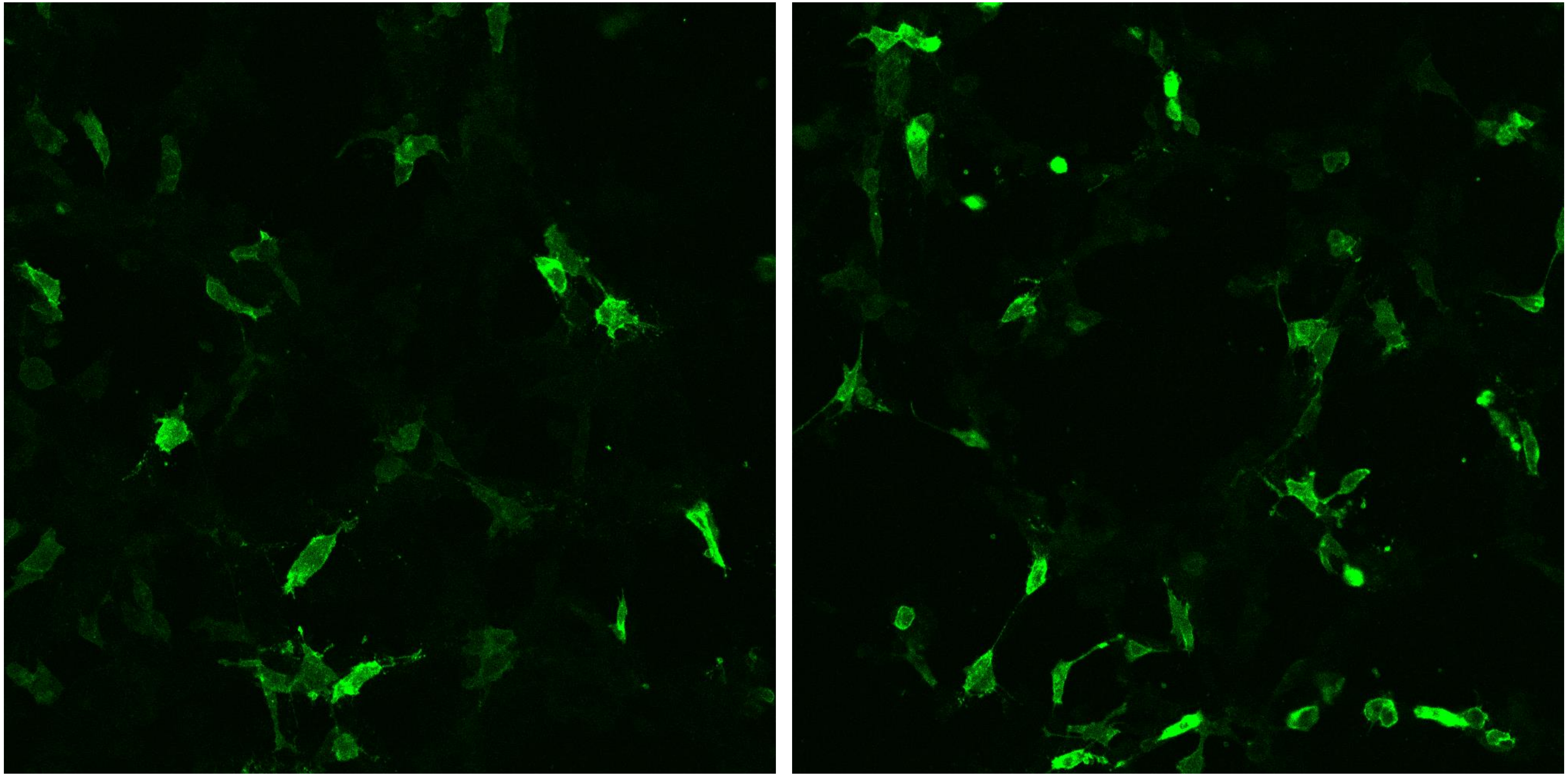

S186W + 20 μM F5257-0462

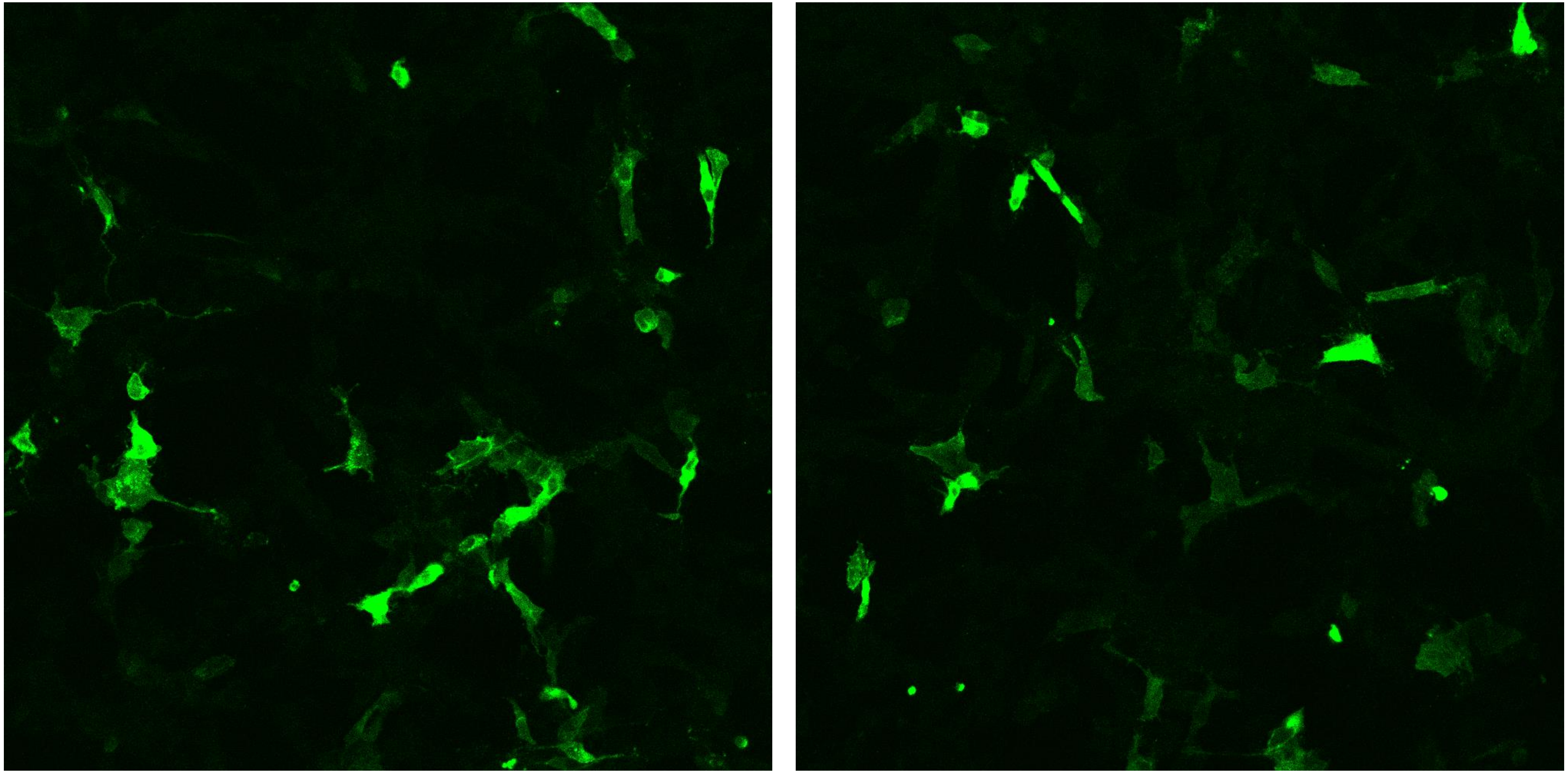

C187Y + DMSO (0.1%)

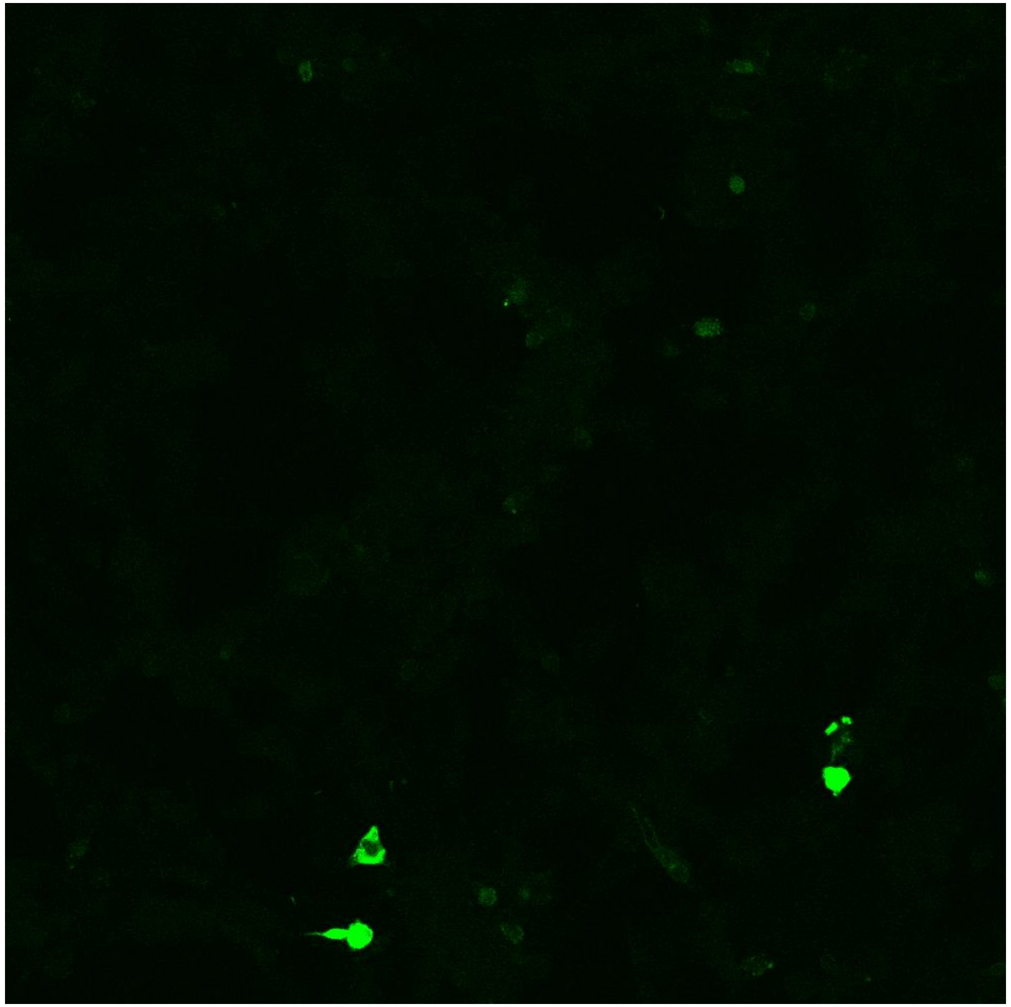

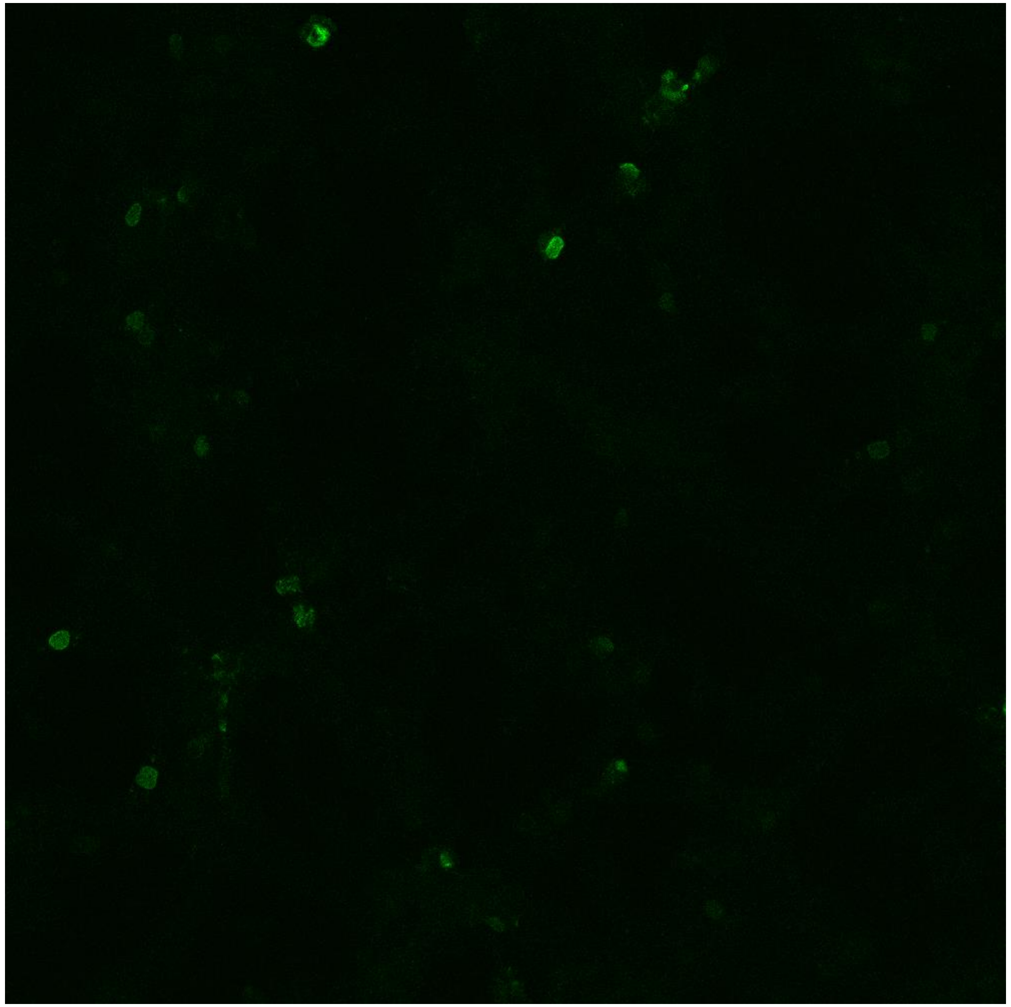

C187Y + DMSO (0.1%)

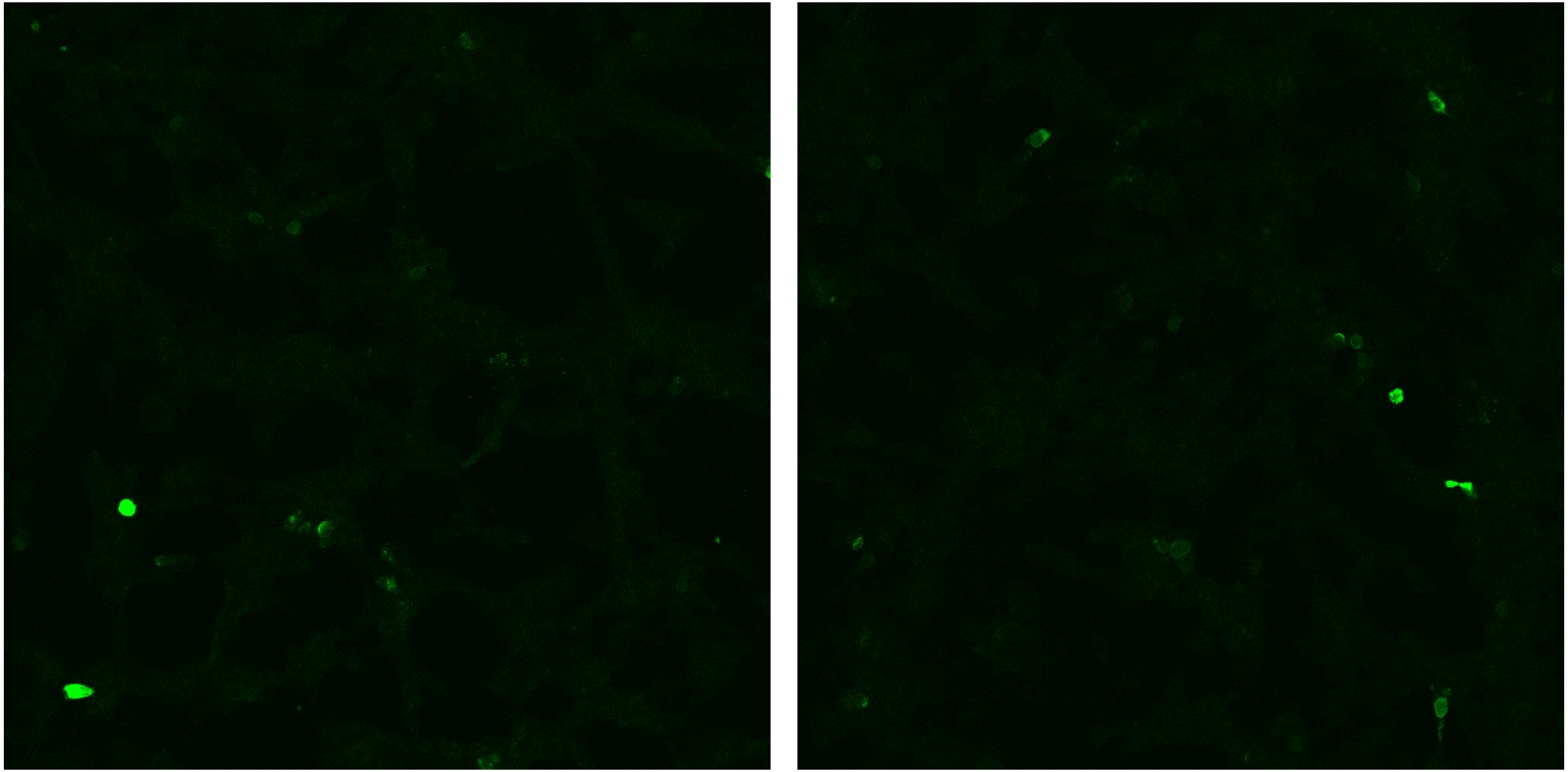

C187Y + 5 µM 9-*cis*-retinal

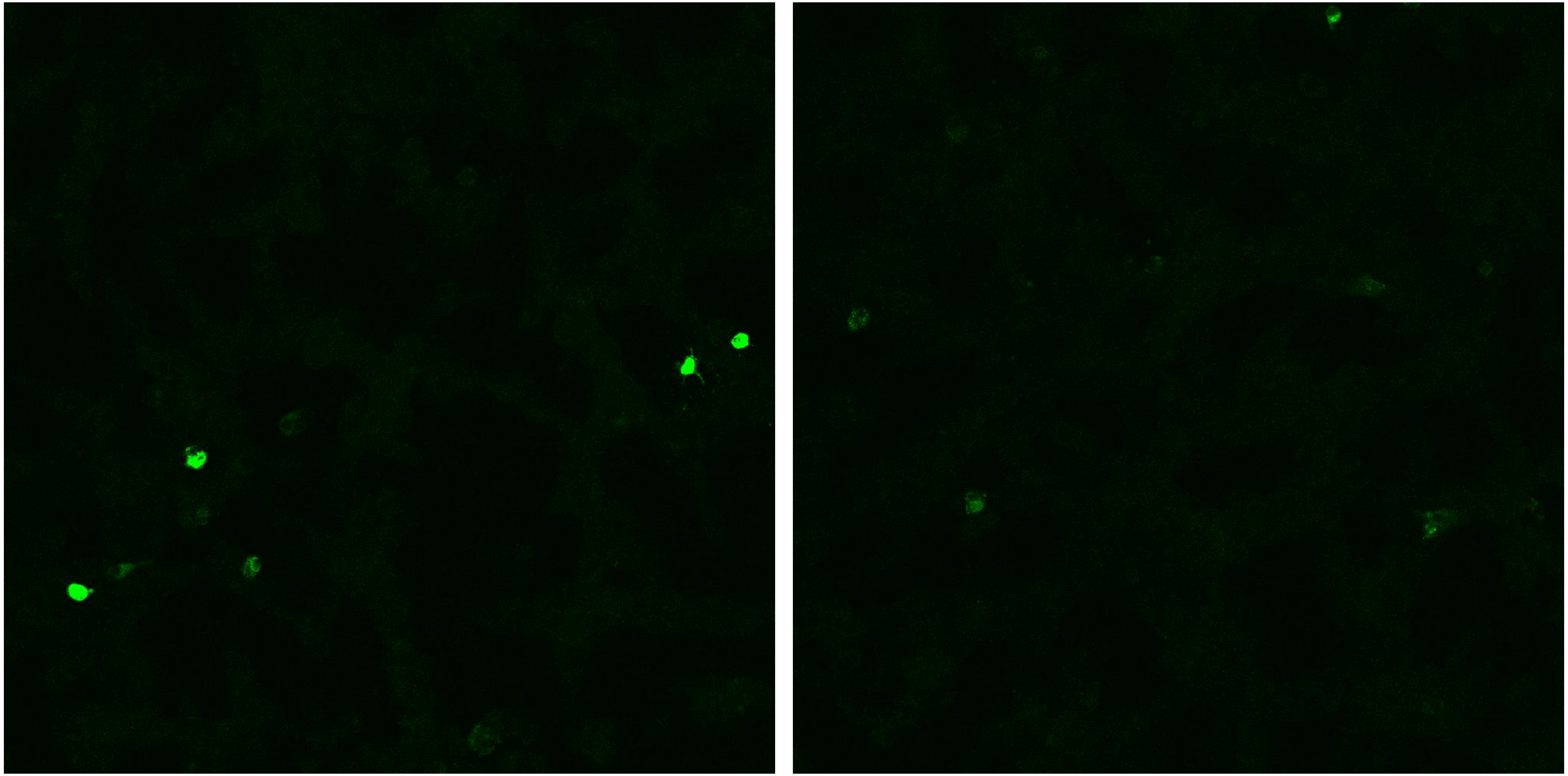

C187Y + 40 μM YC-001

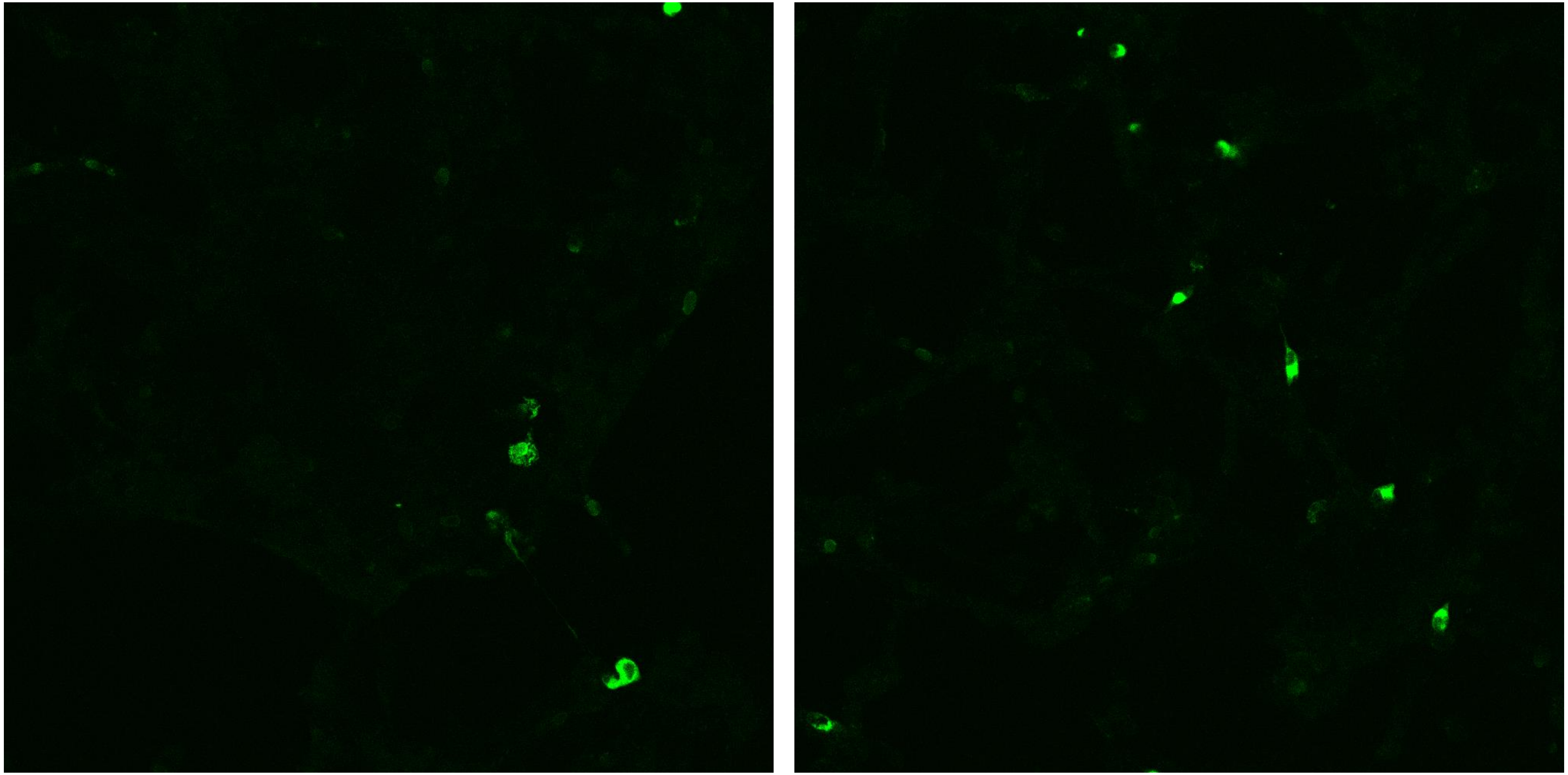

C187Y + 20 μM F5257-0462

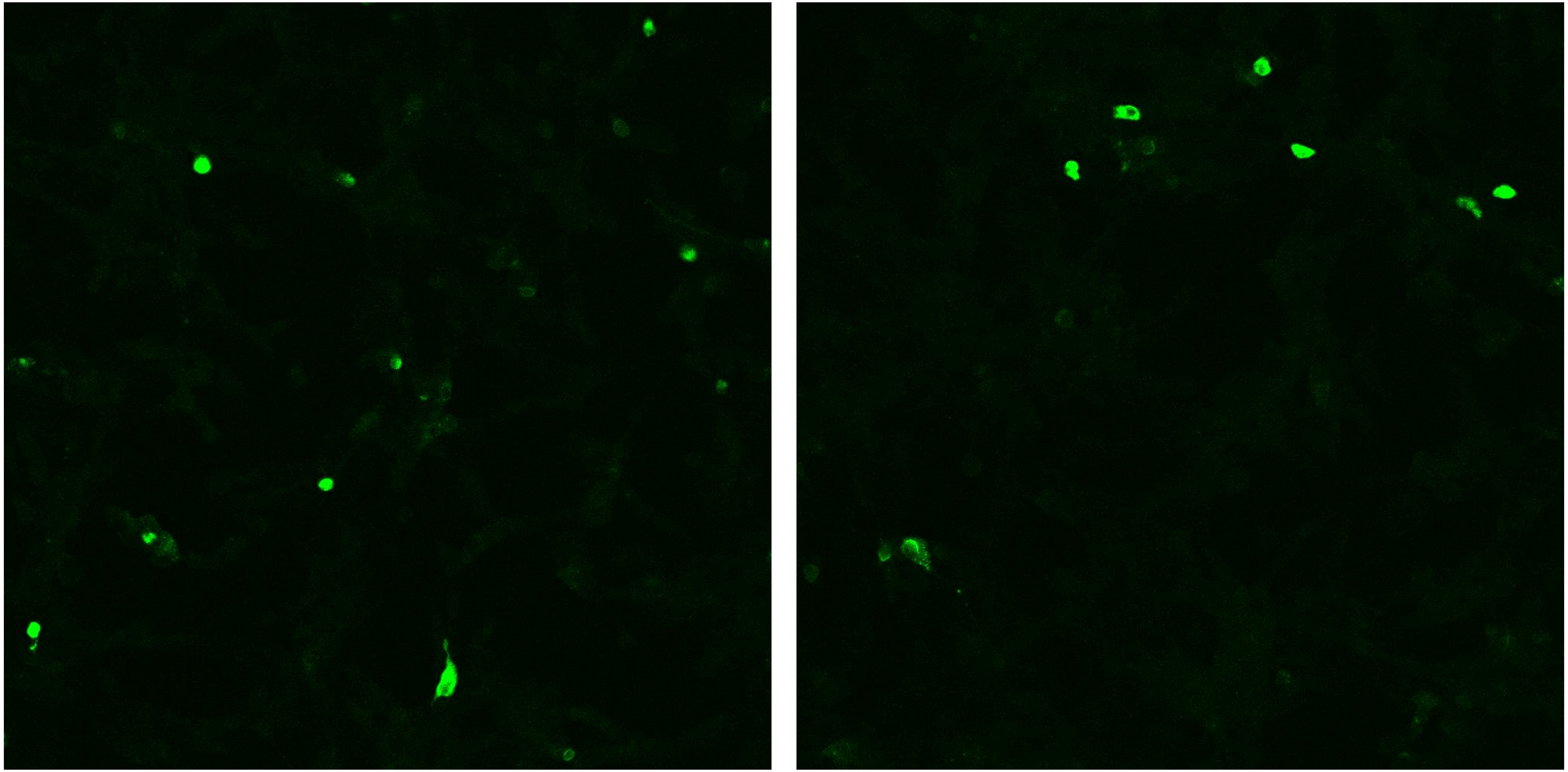

G188R + DMSO (0.1%)

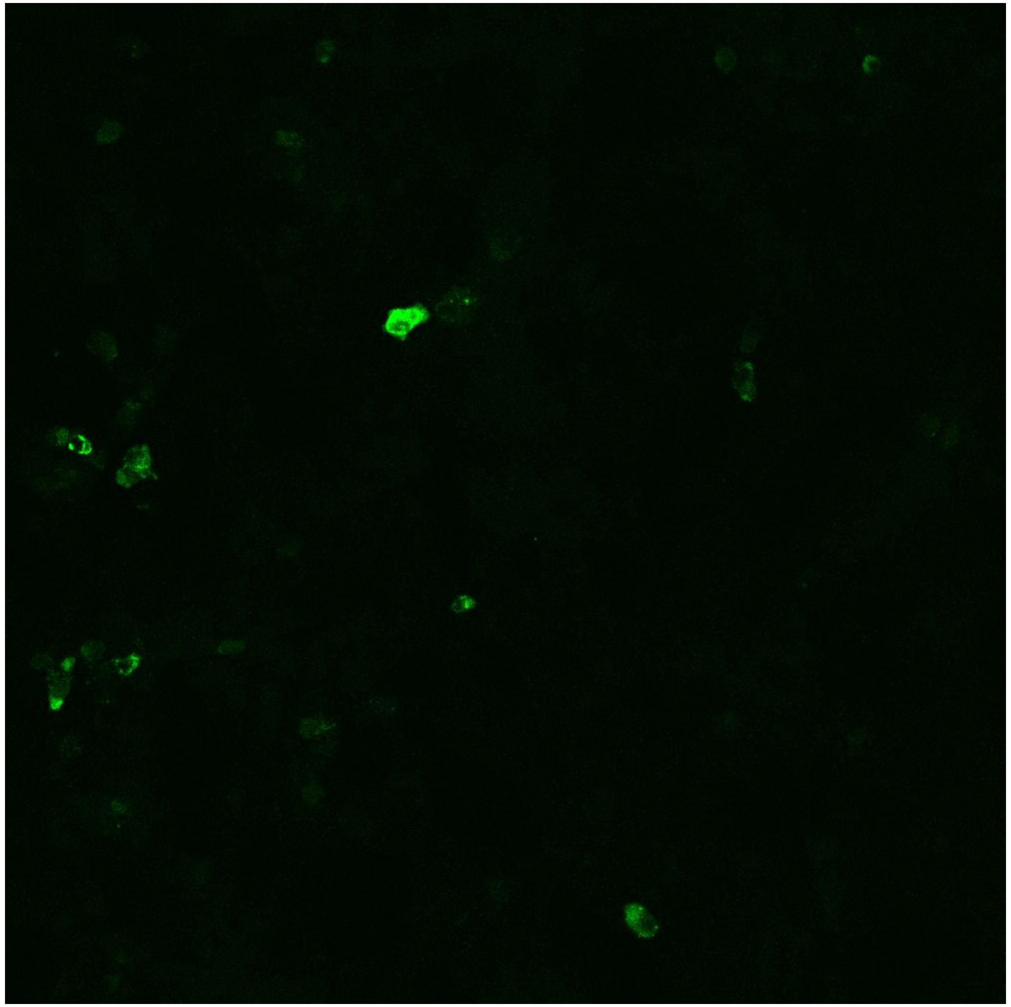

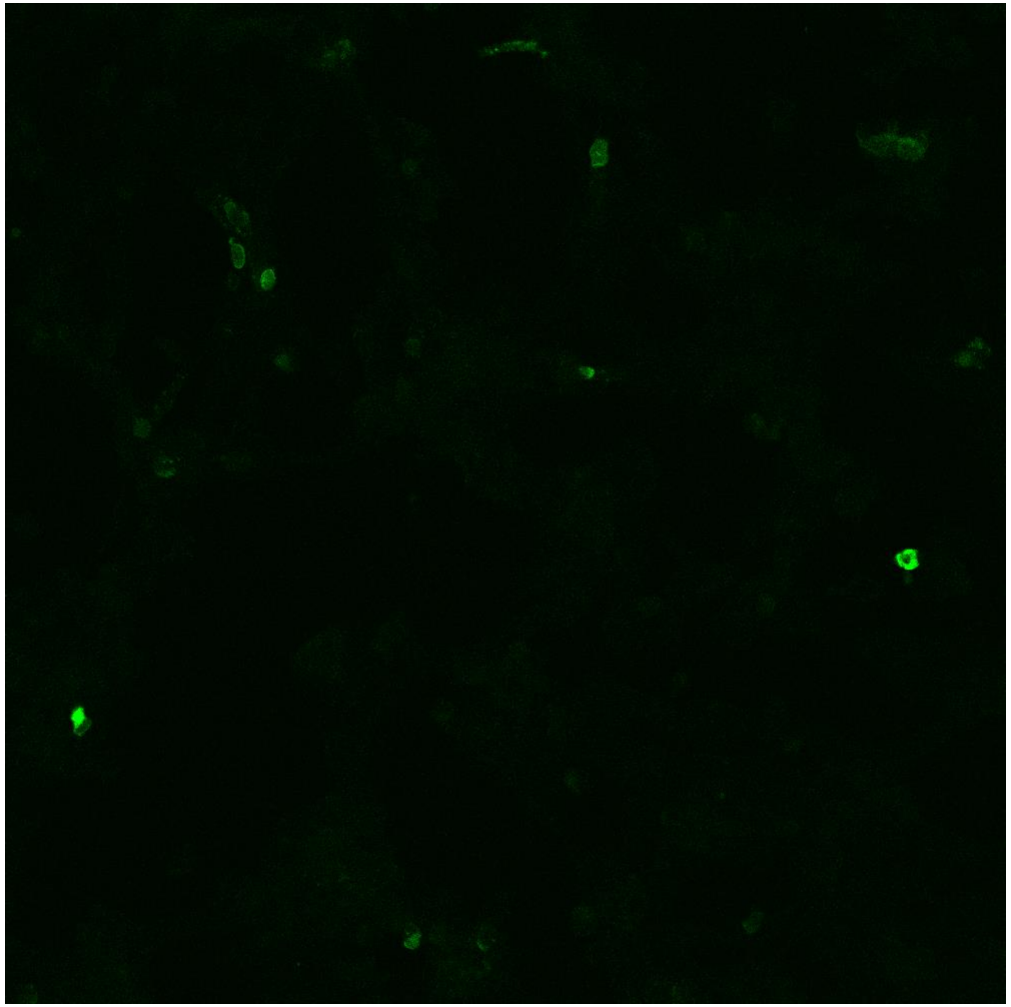

G188R + DMSO (0.1%)

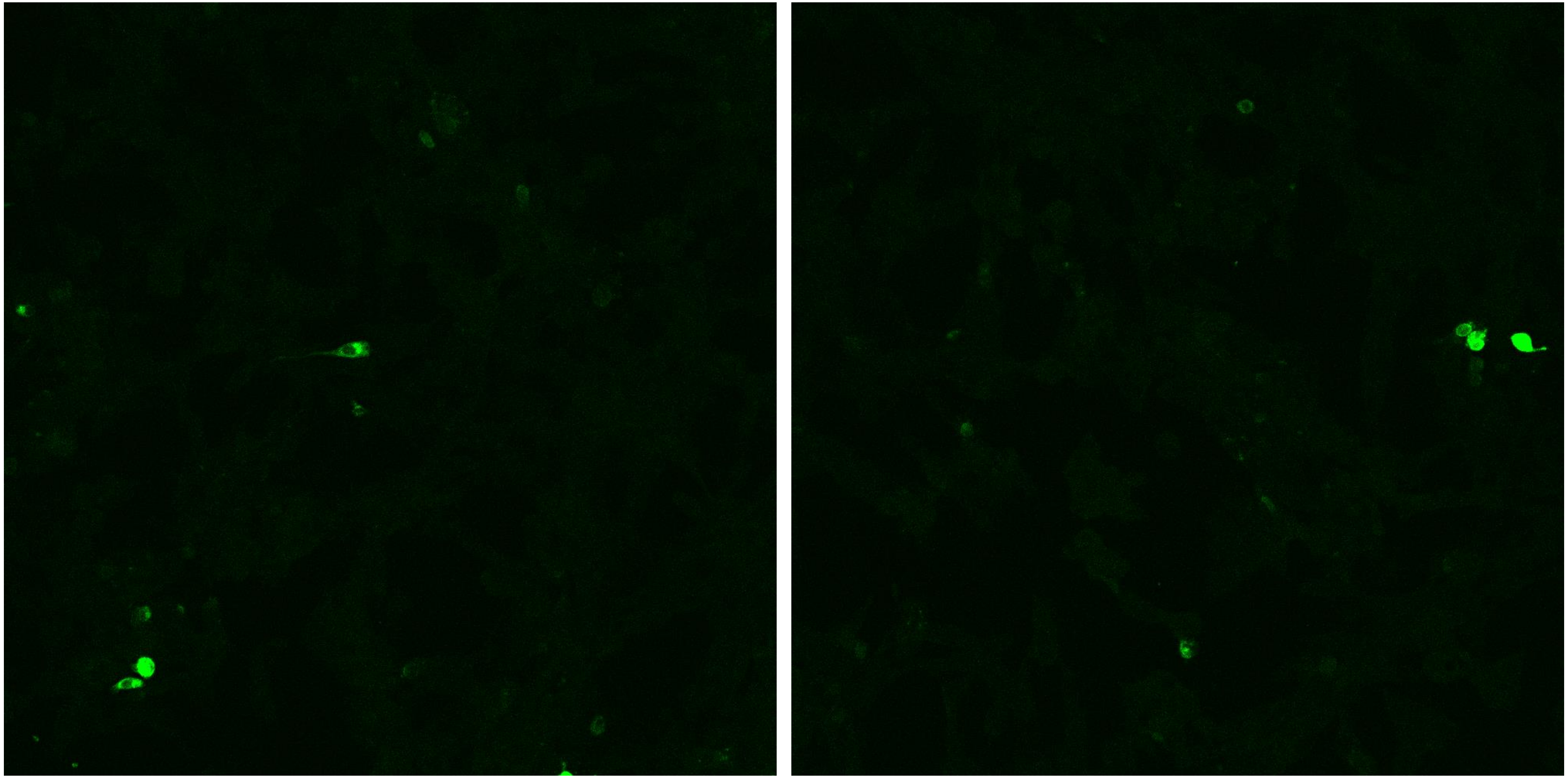

G188R + 5 µM 9-*cis*-retinal

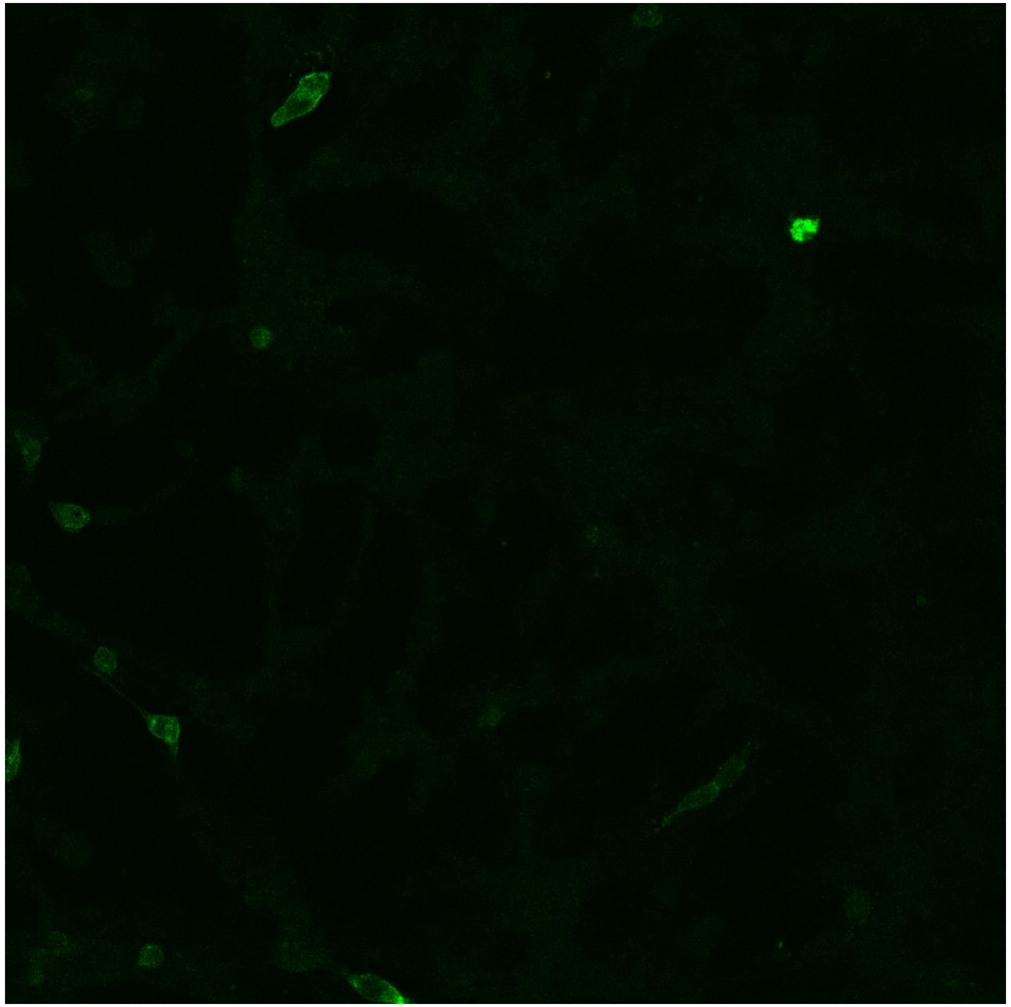

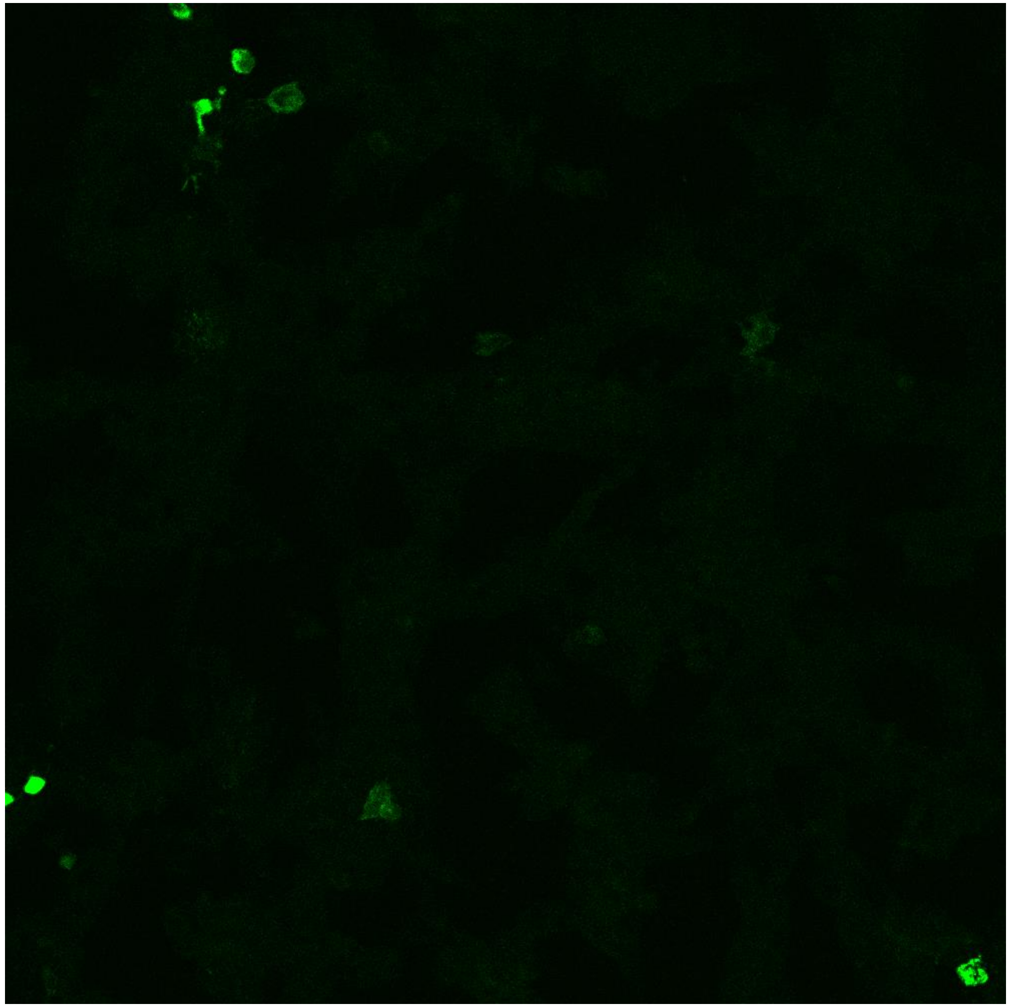

G188R + 40 µM YC-001

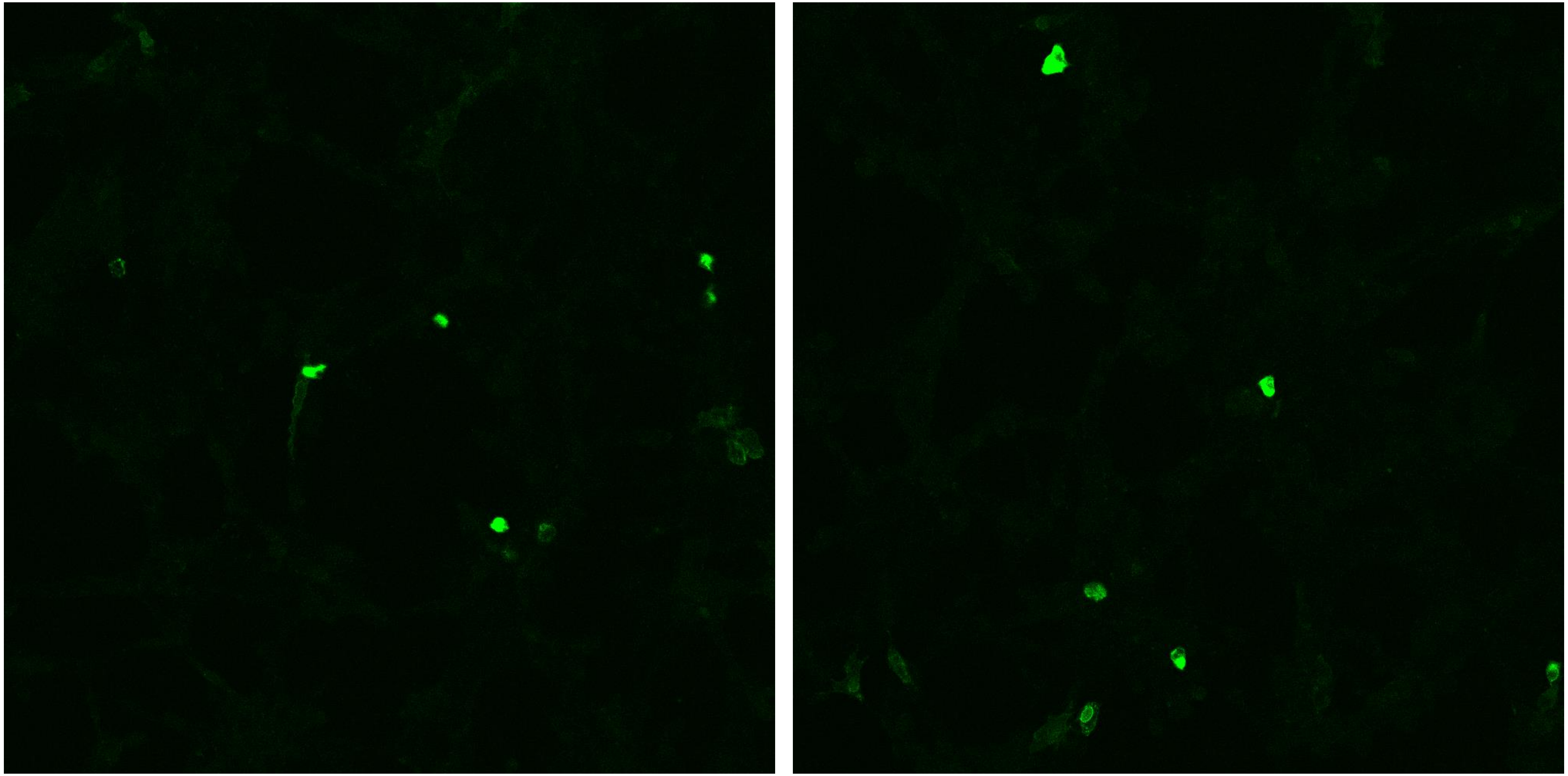

G188R + 20 μM F5257-0462

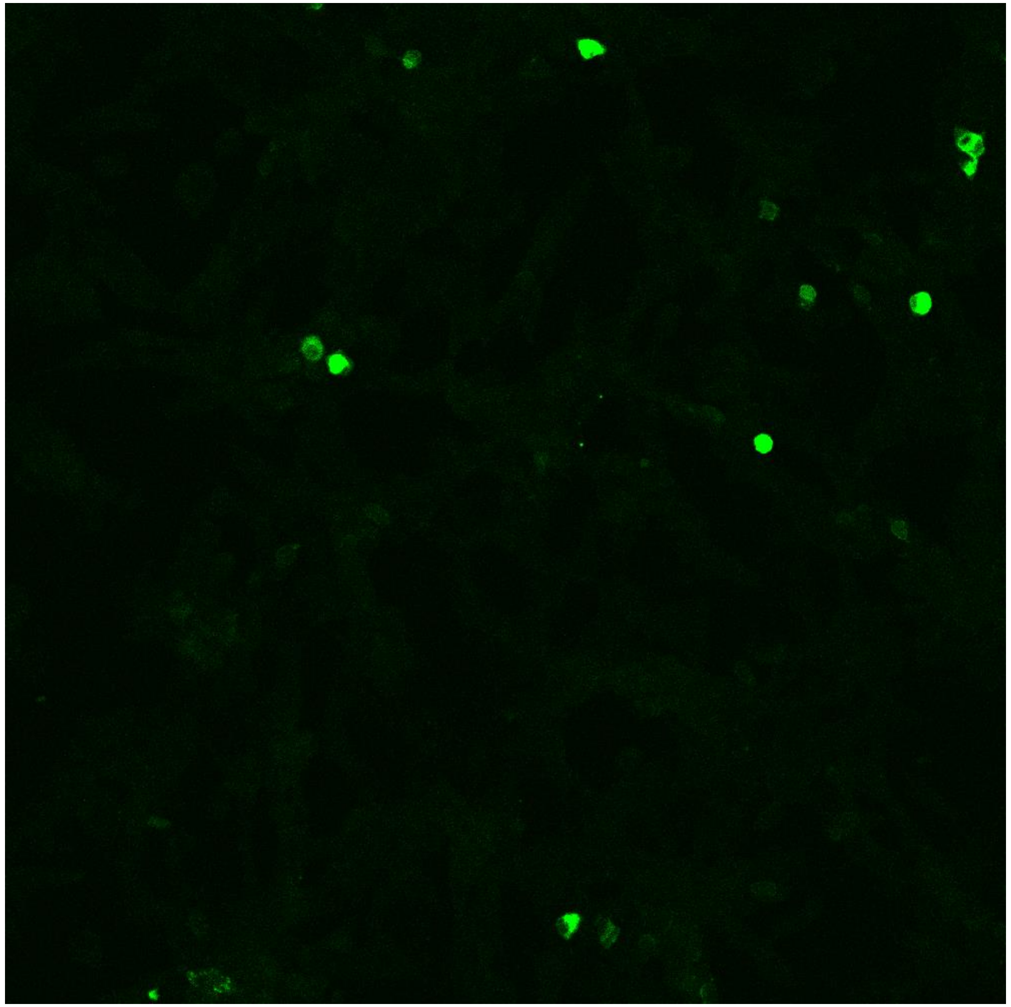

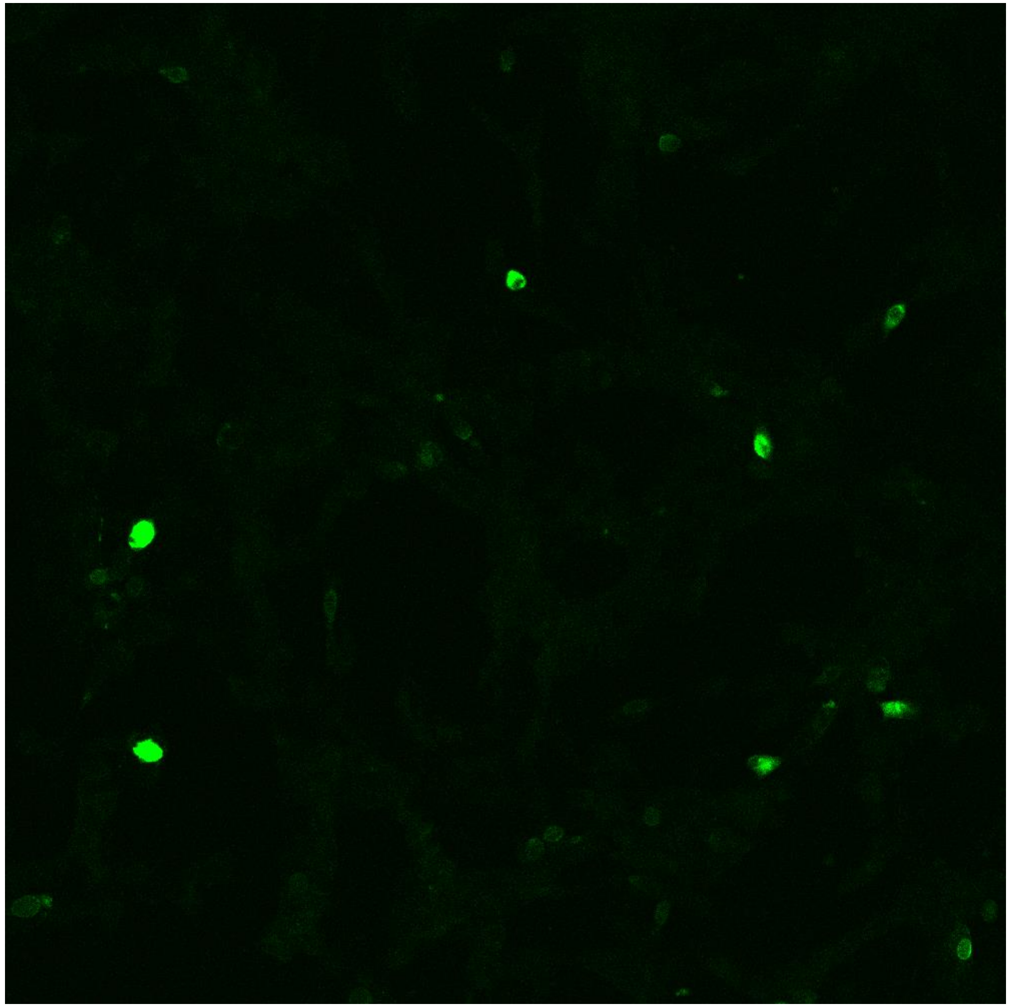

G188E + DMSO (0.1%)

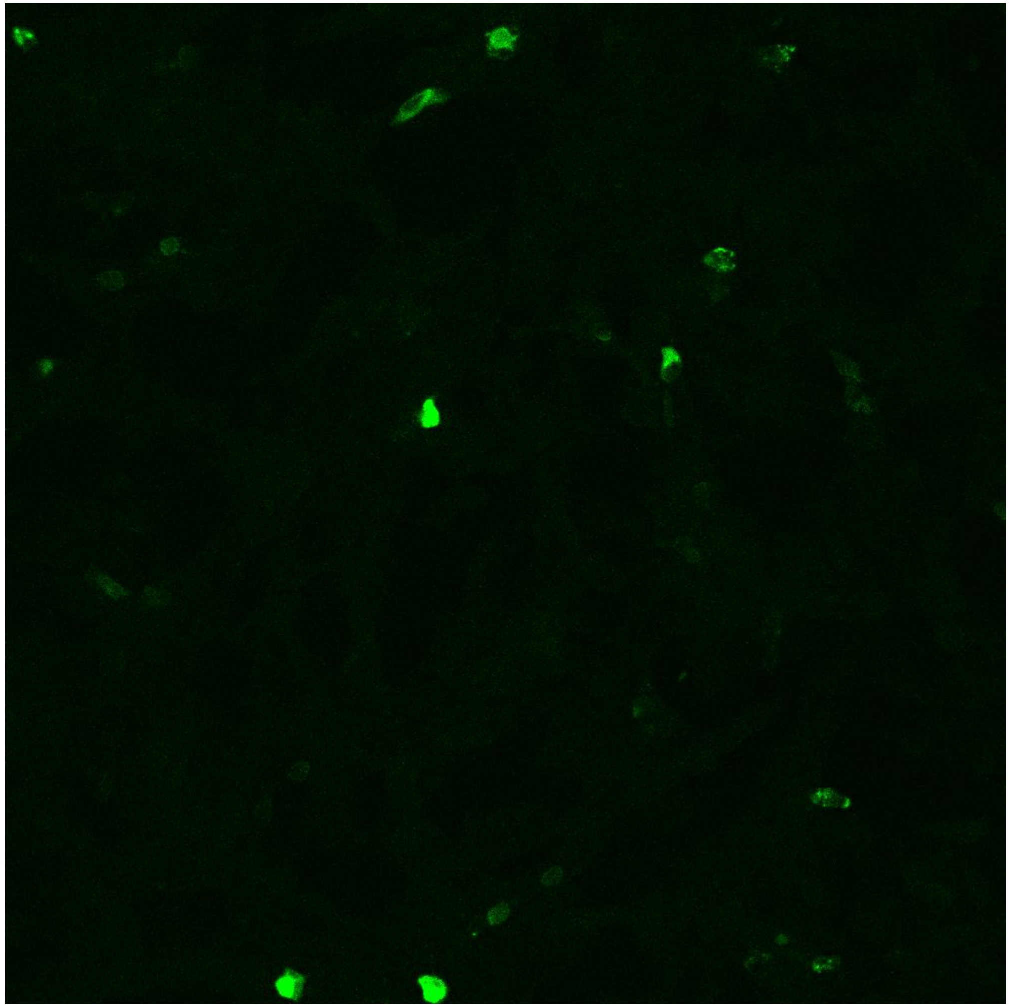

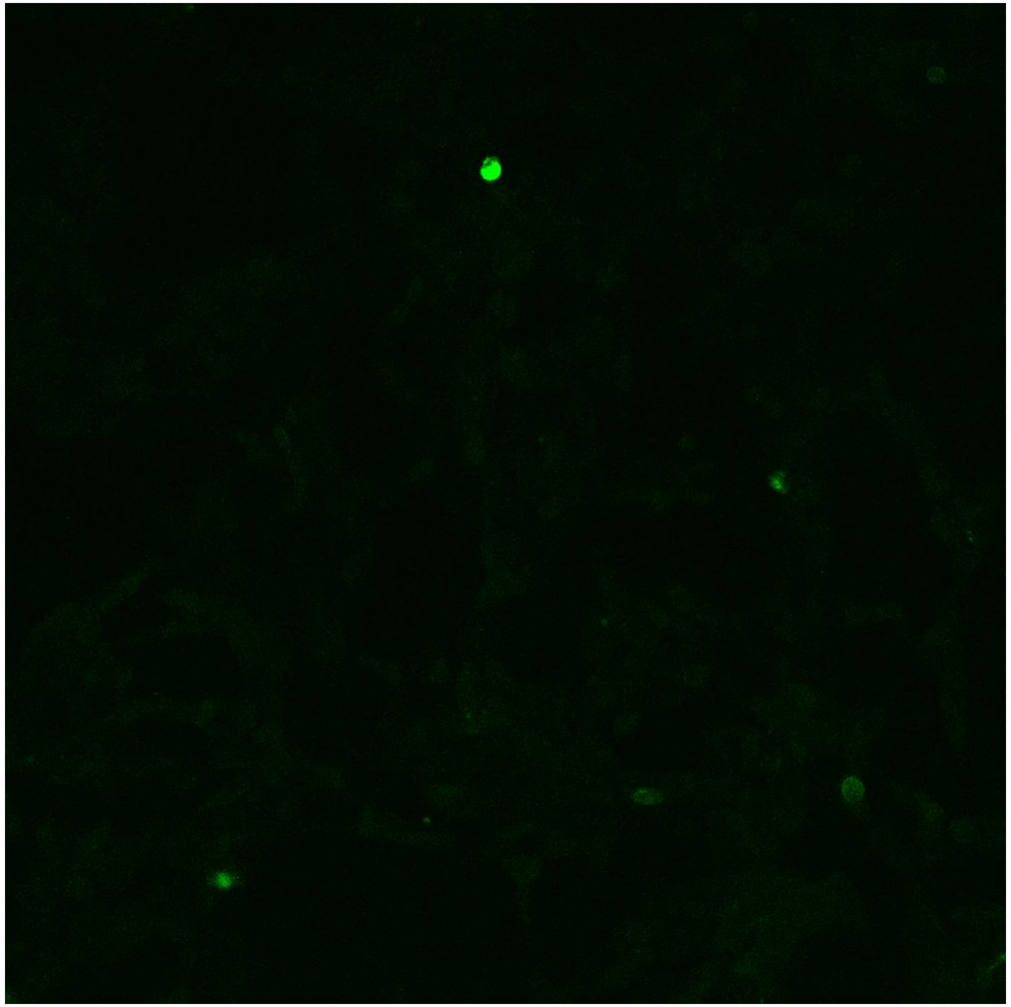

G188E + DMSO (0.1%)

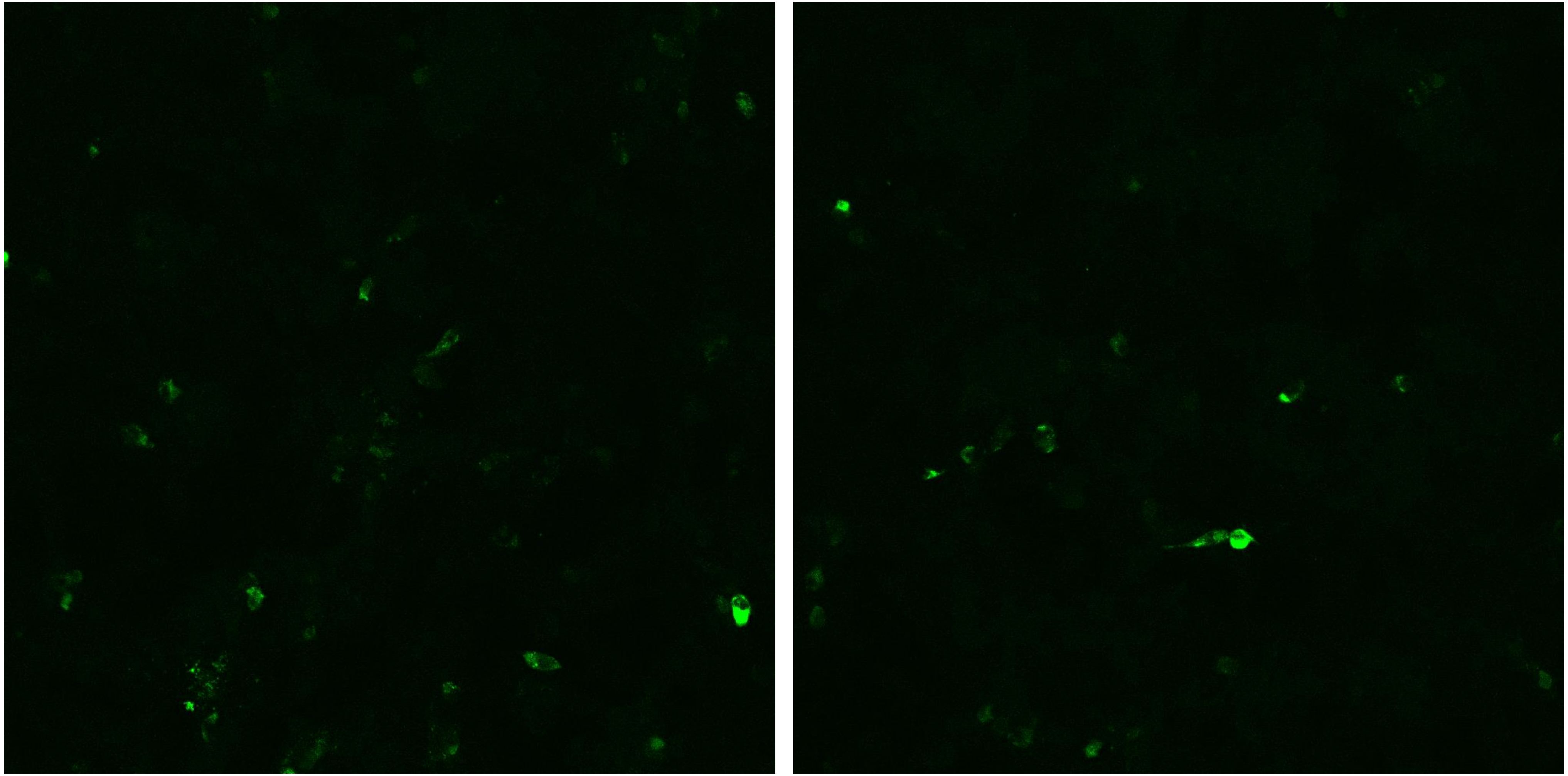

G188E + 5 μM 9-*cis*-retinal

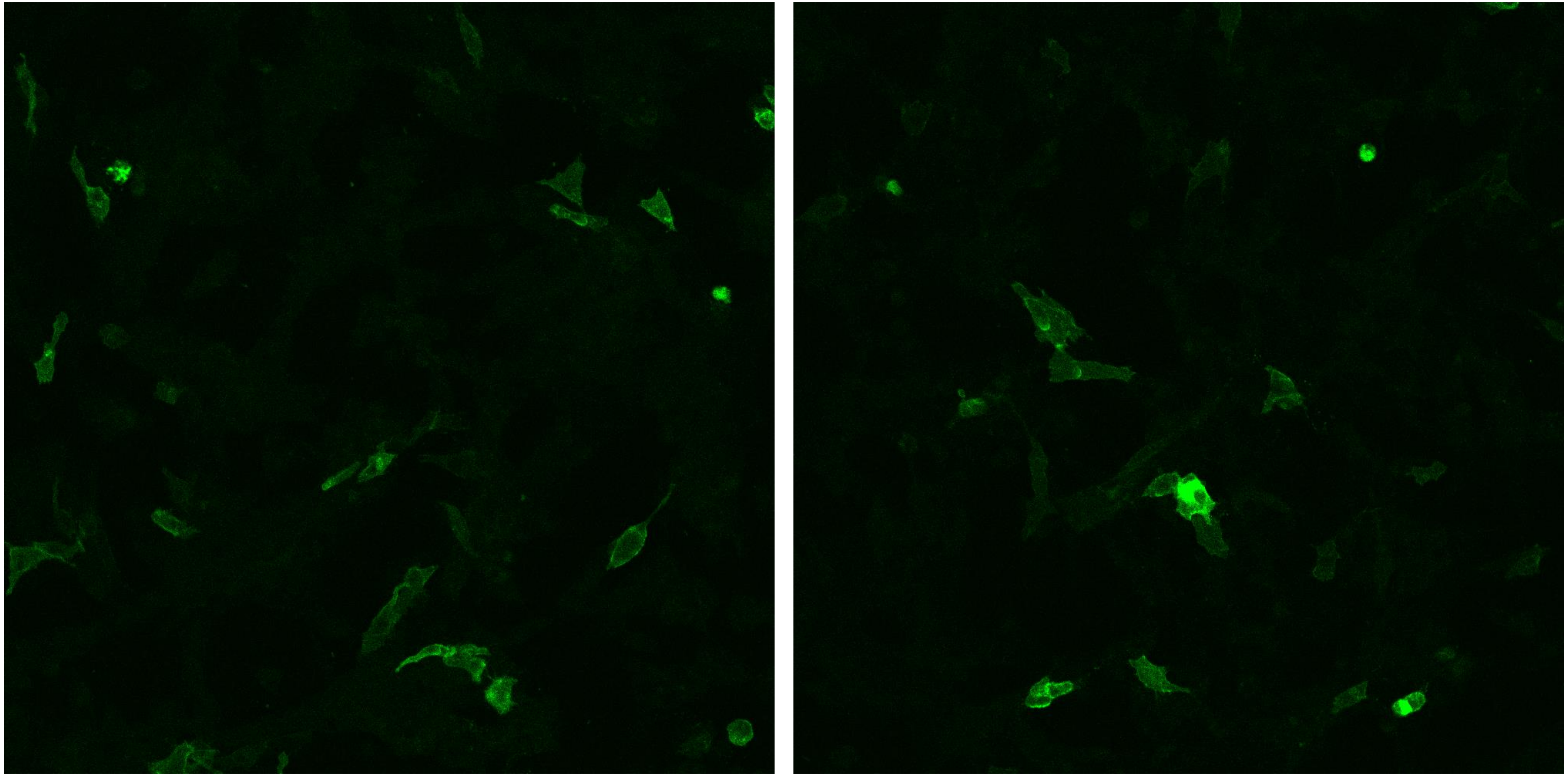

G188E + 40 μM YC-001

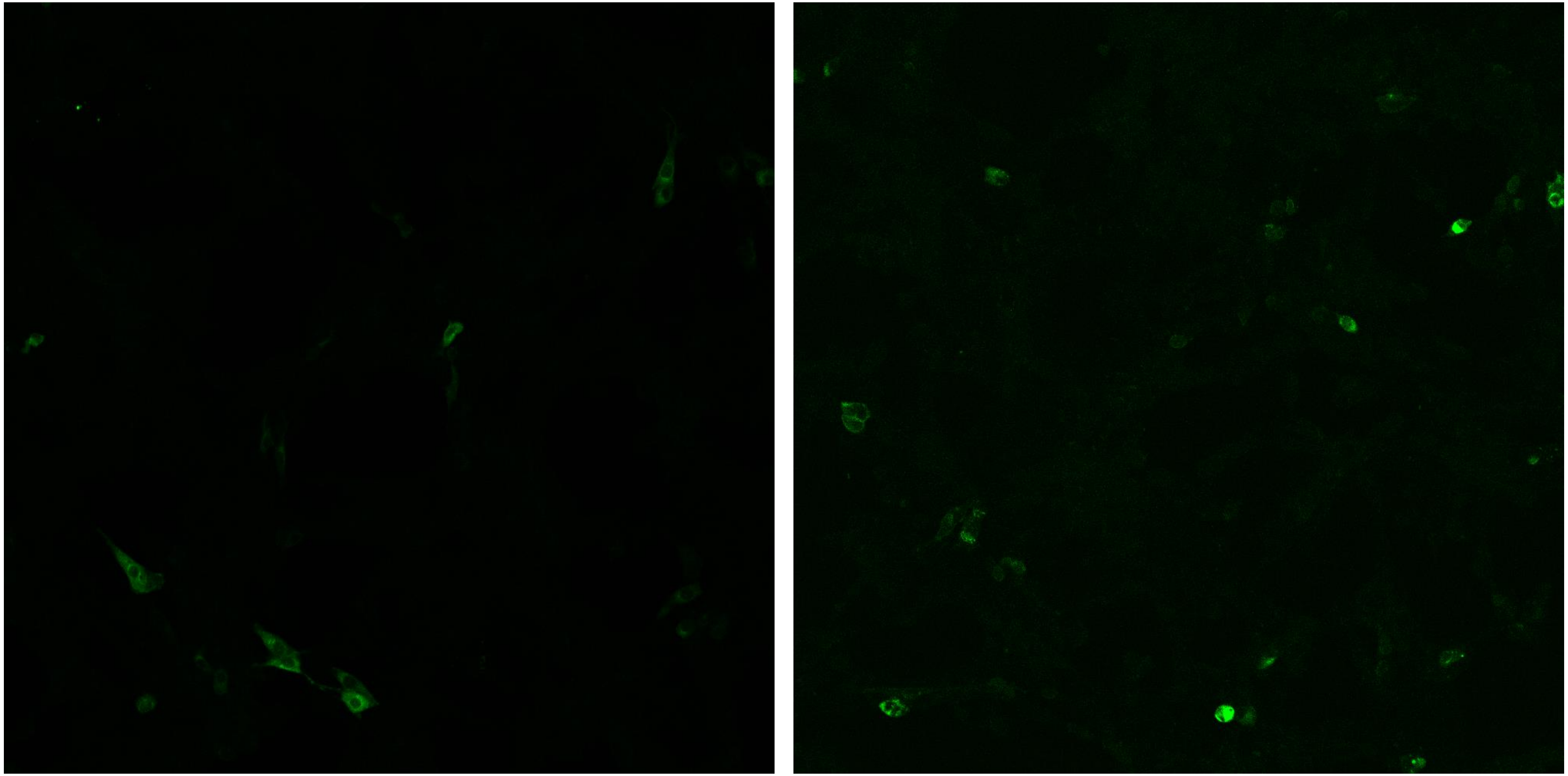

G188E + 20 μM F5257-0462

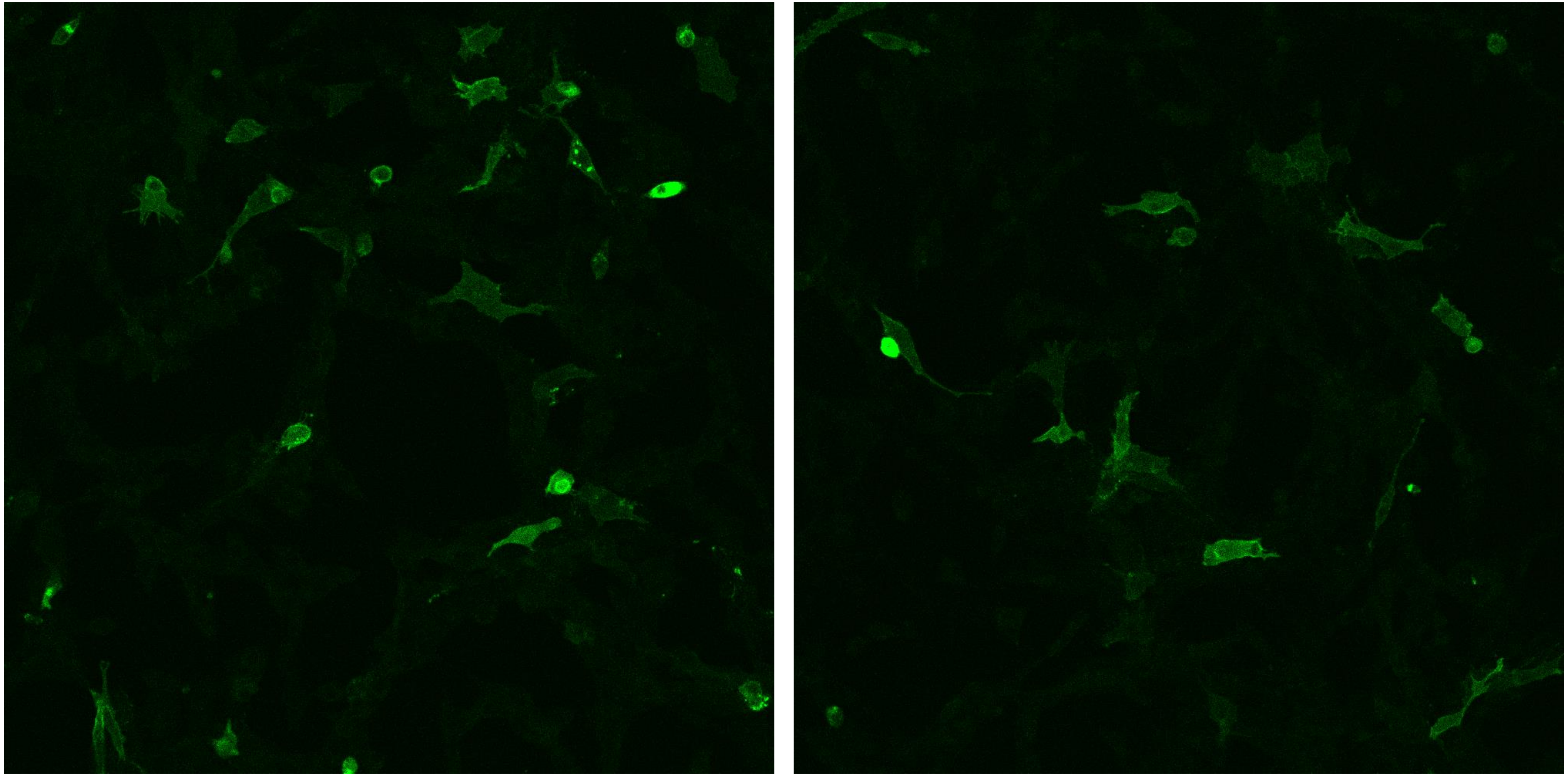

D190N + DMSO (0.1%)

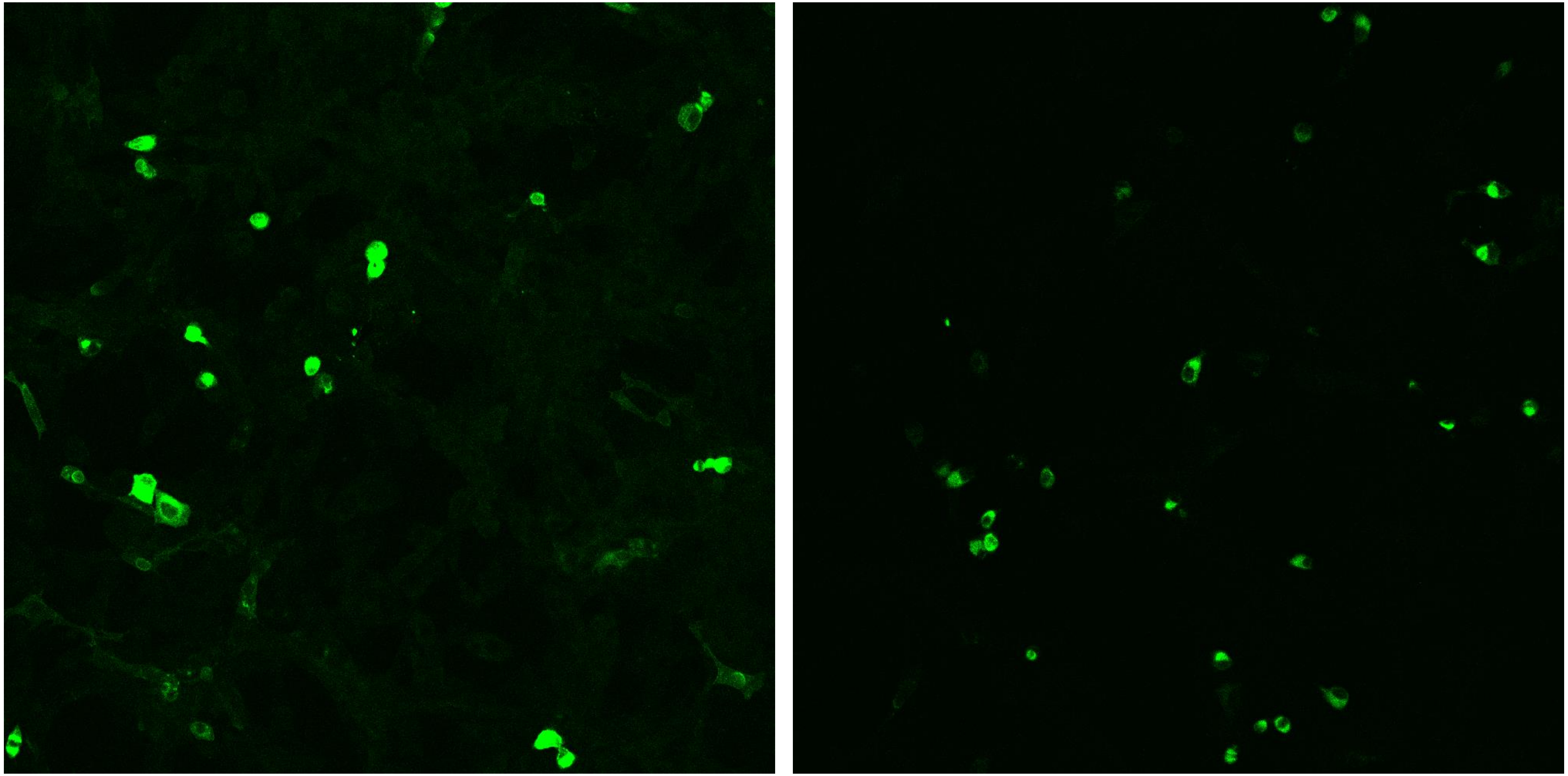

D190N + DMSO (0.1%)

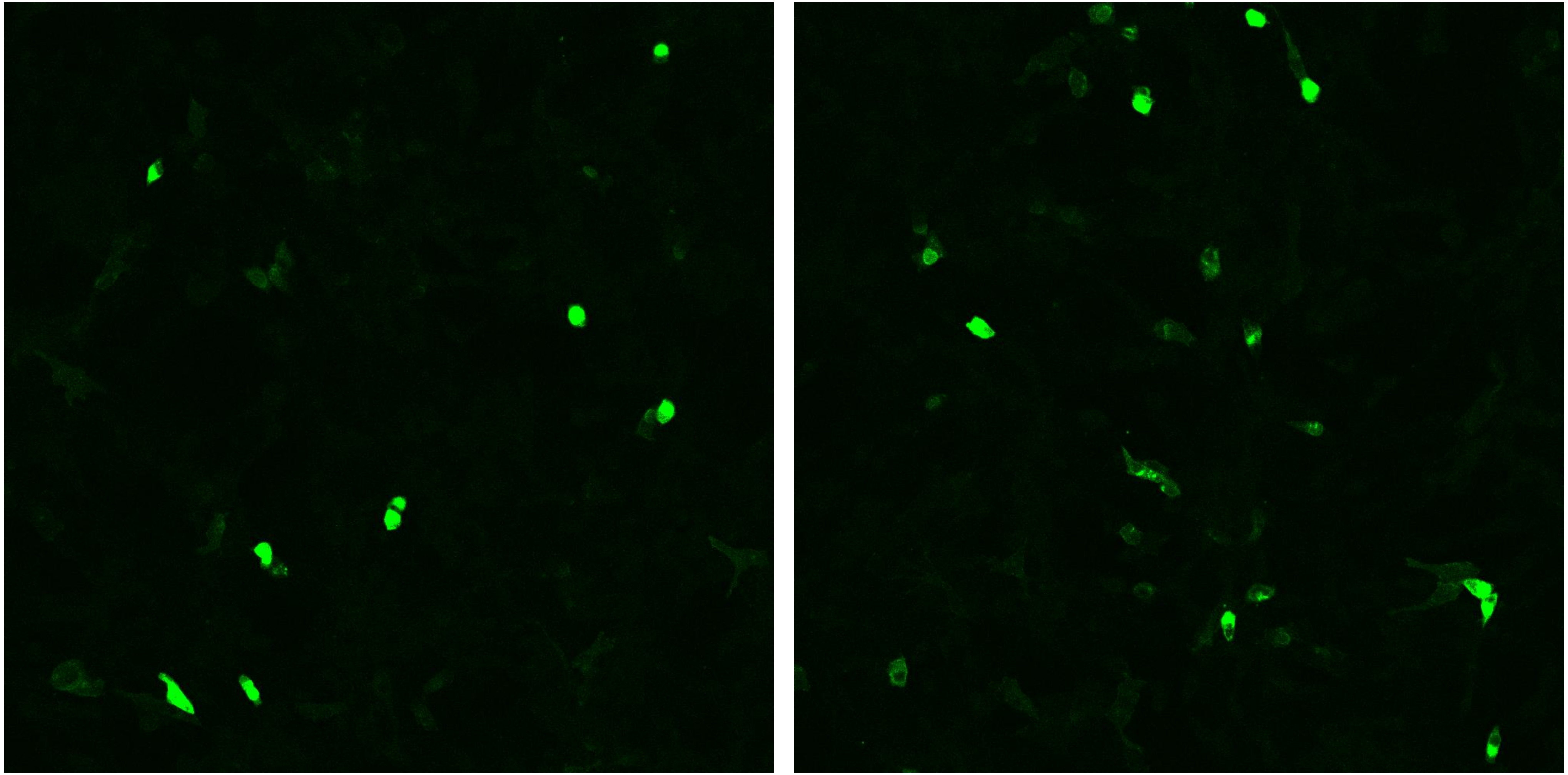

D190N + 5 µM 9-*cis*-retinal

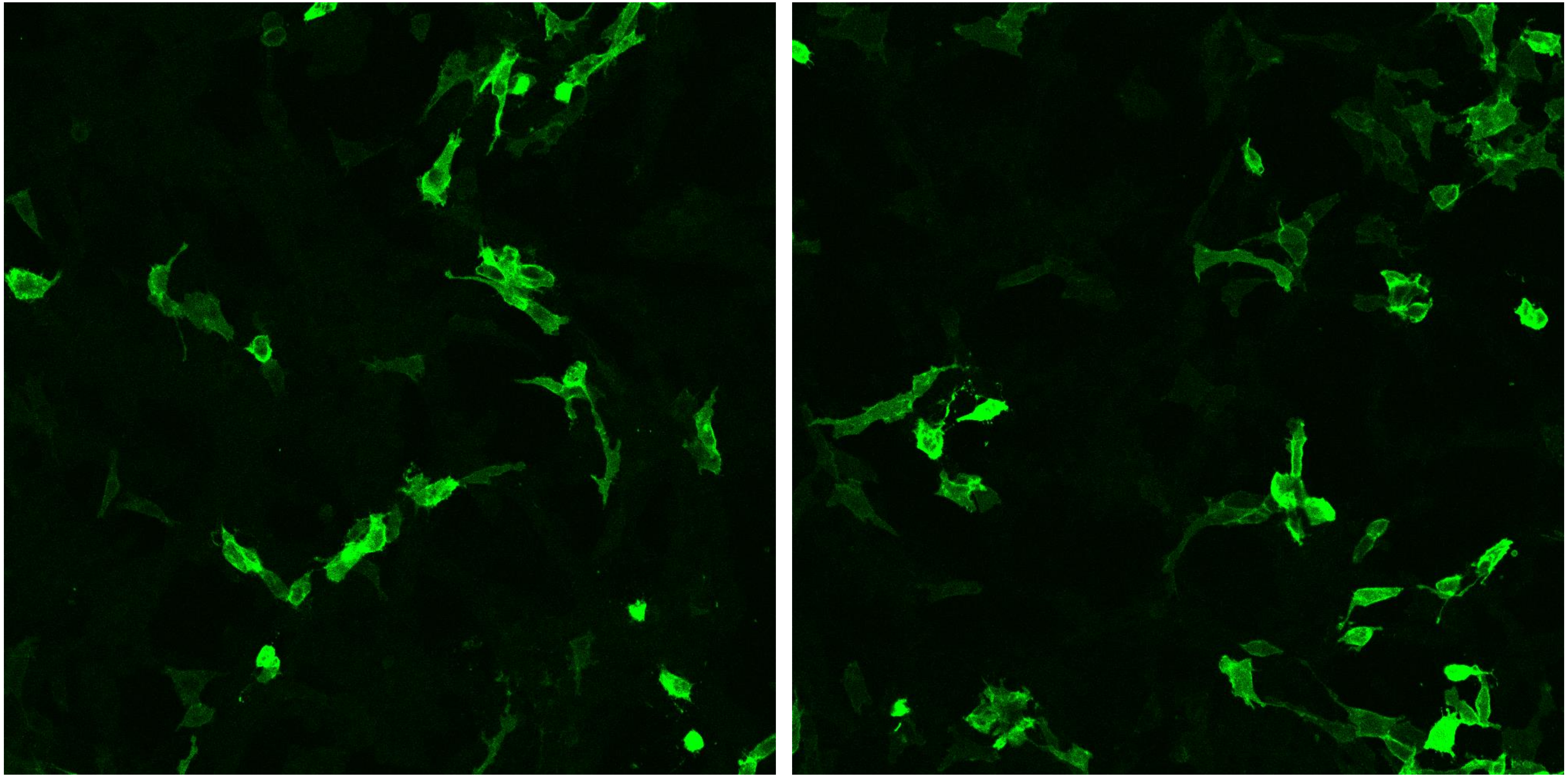

D190N + 40 µM YC-001

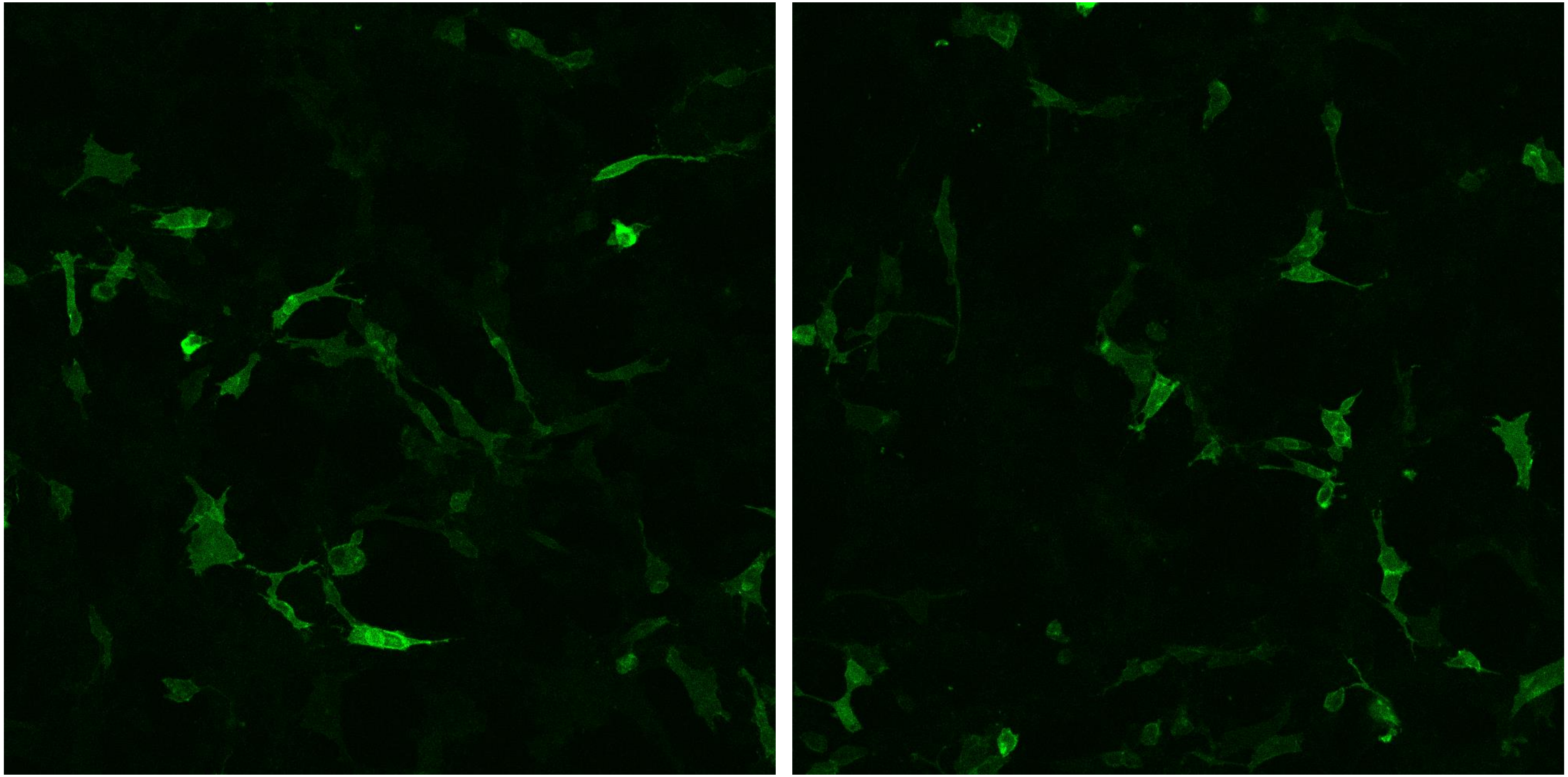

D190N + 20 μM F5257-0462

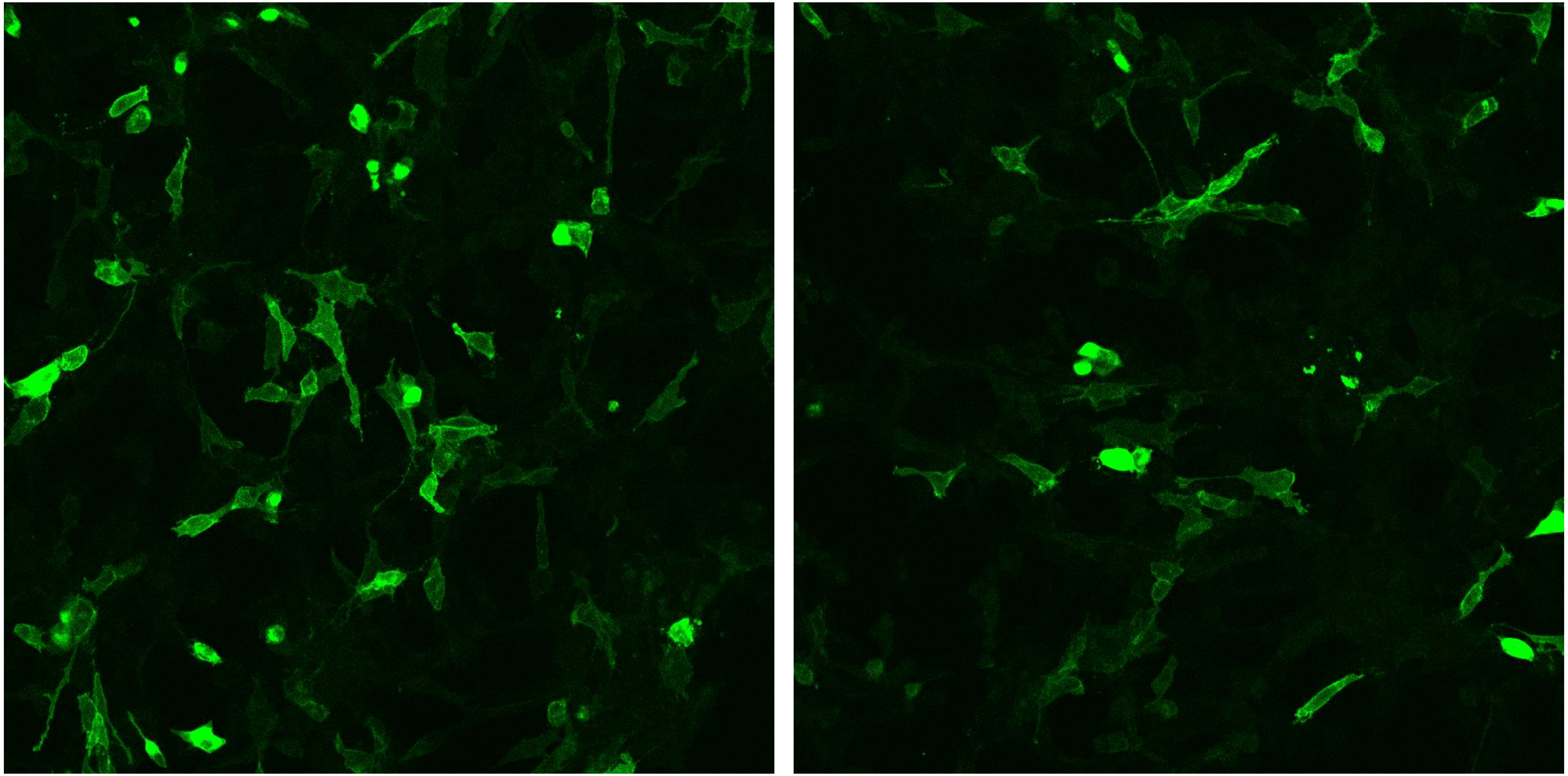

D190Y + DMSO (0.1%)

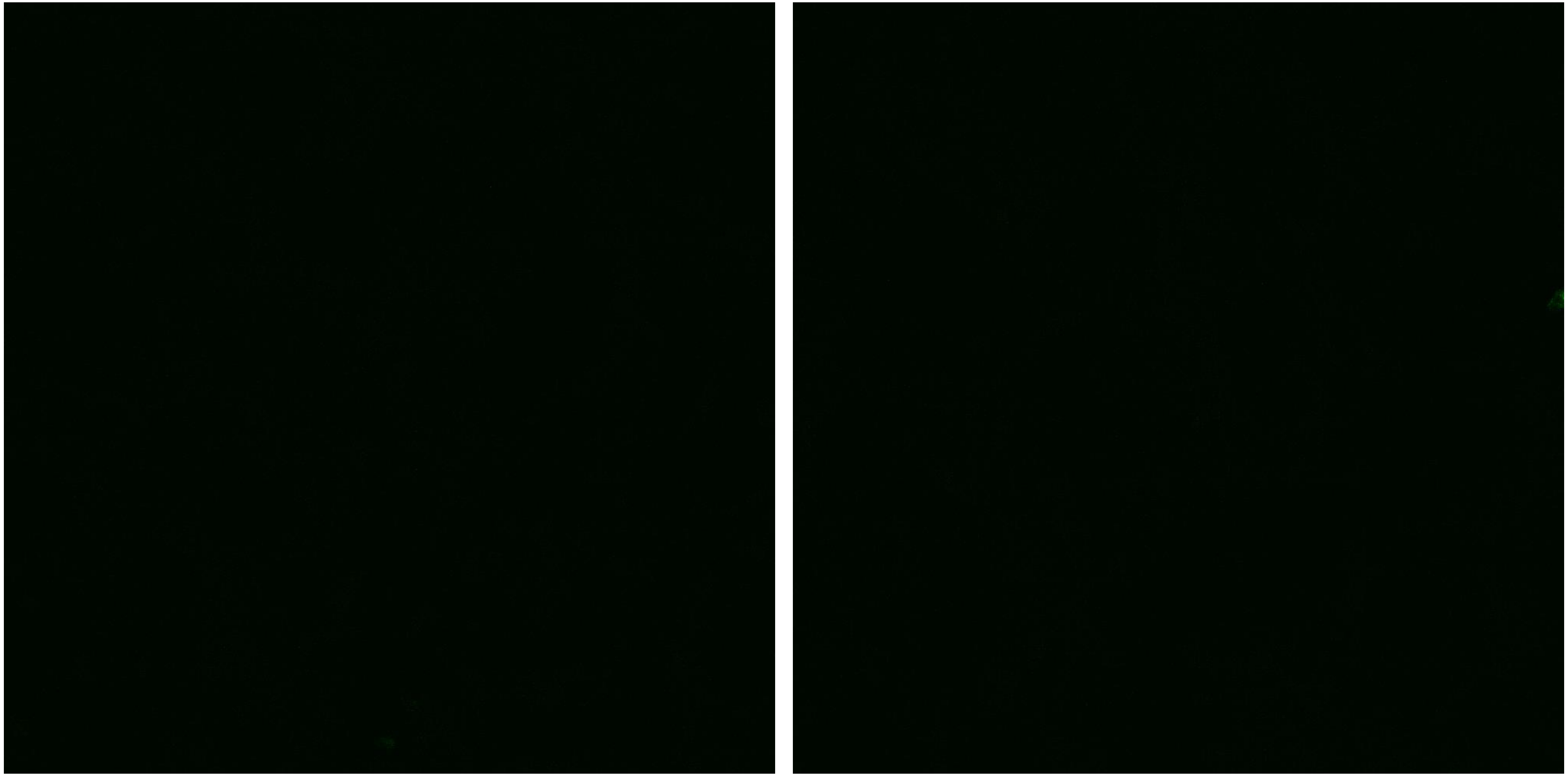

D190Y + DMSO (0.1%)

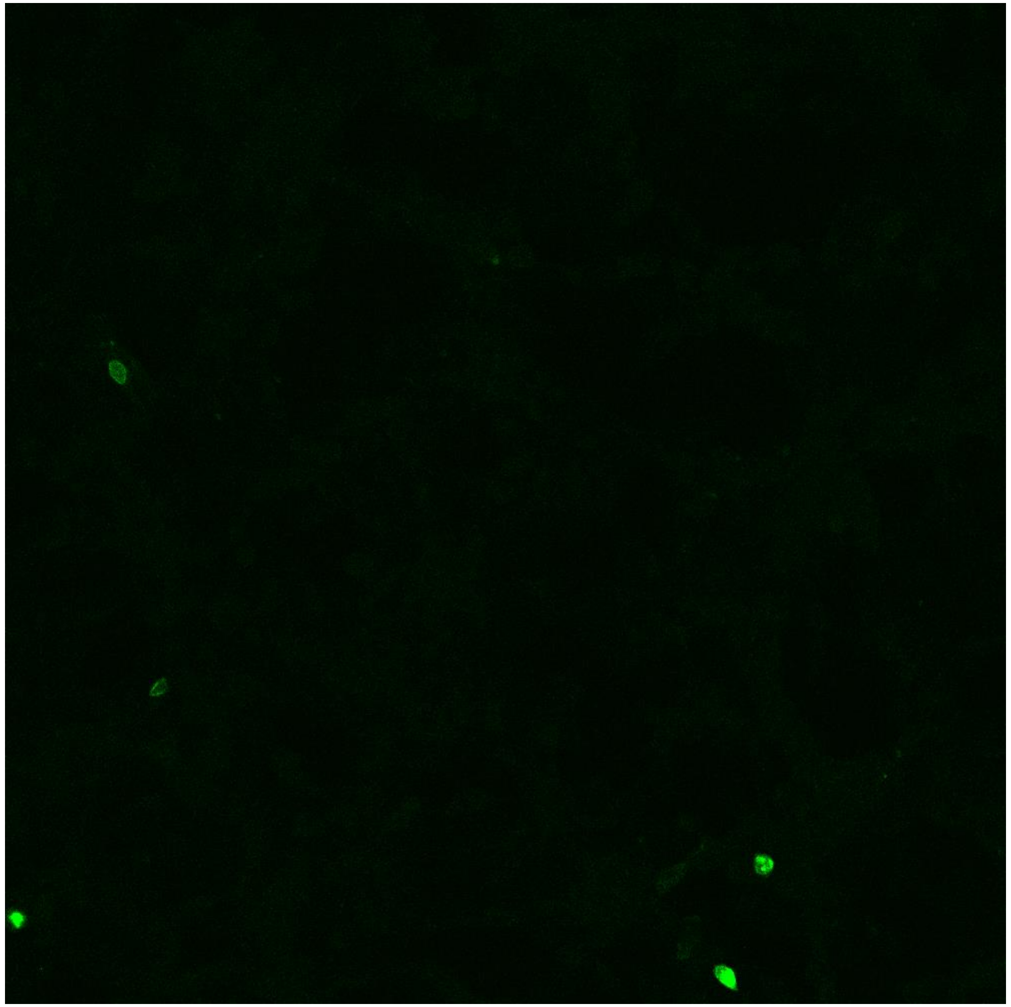

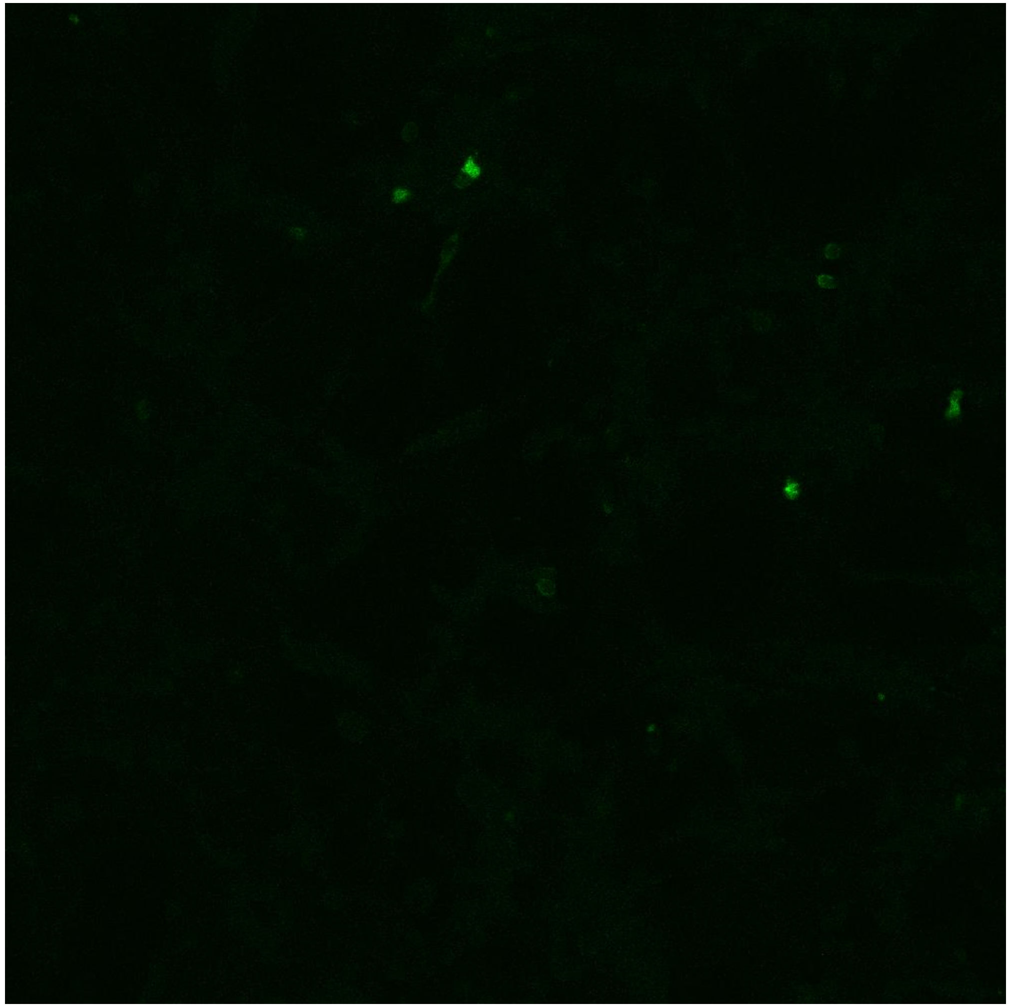

D190Y + 5 µM 9-*cis*-retinal

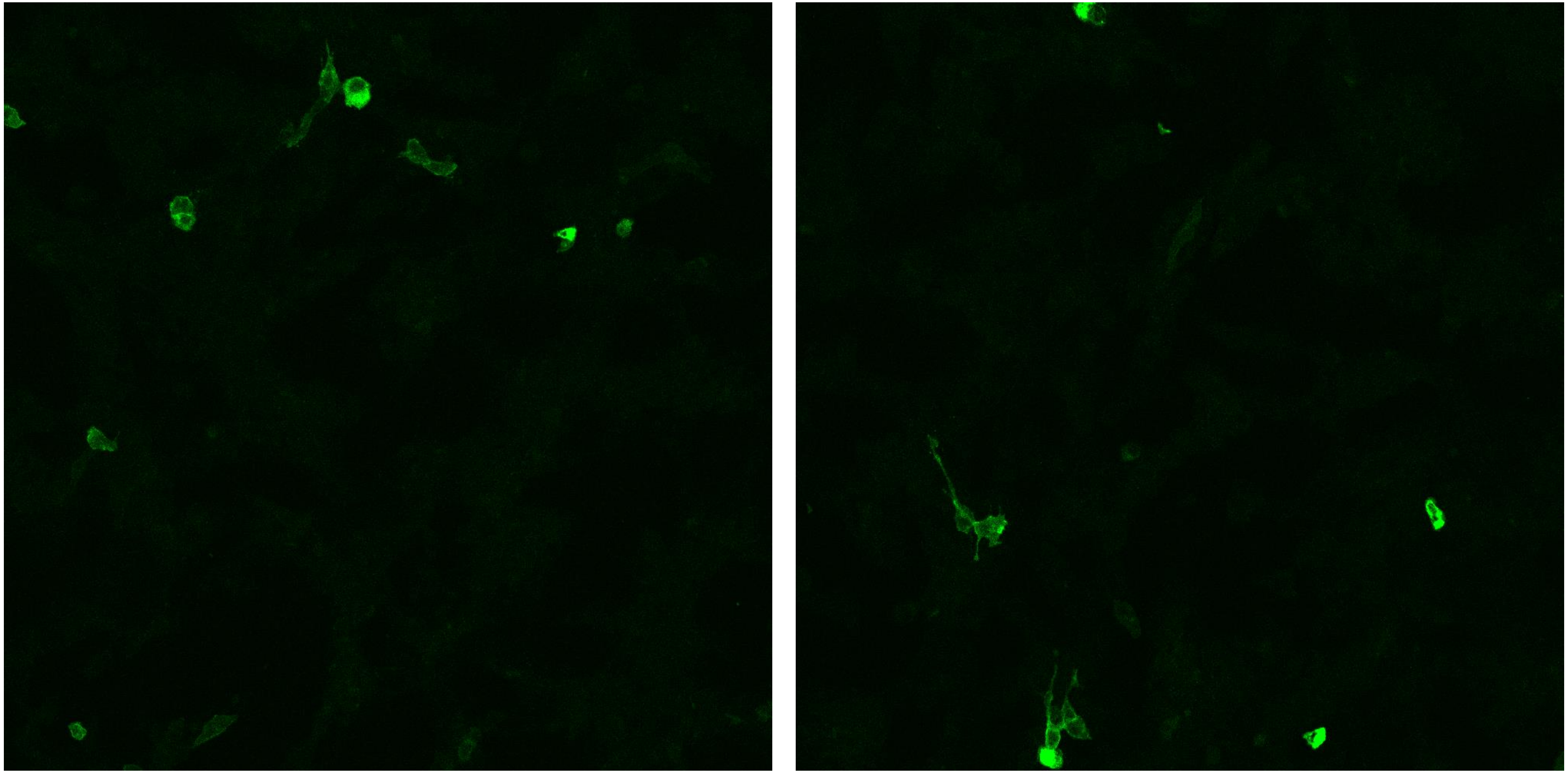

D190Y + 40 μM YC-001

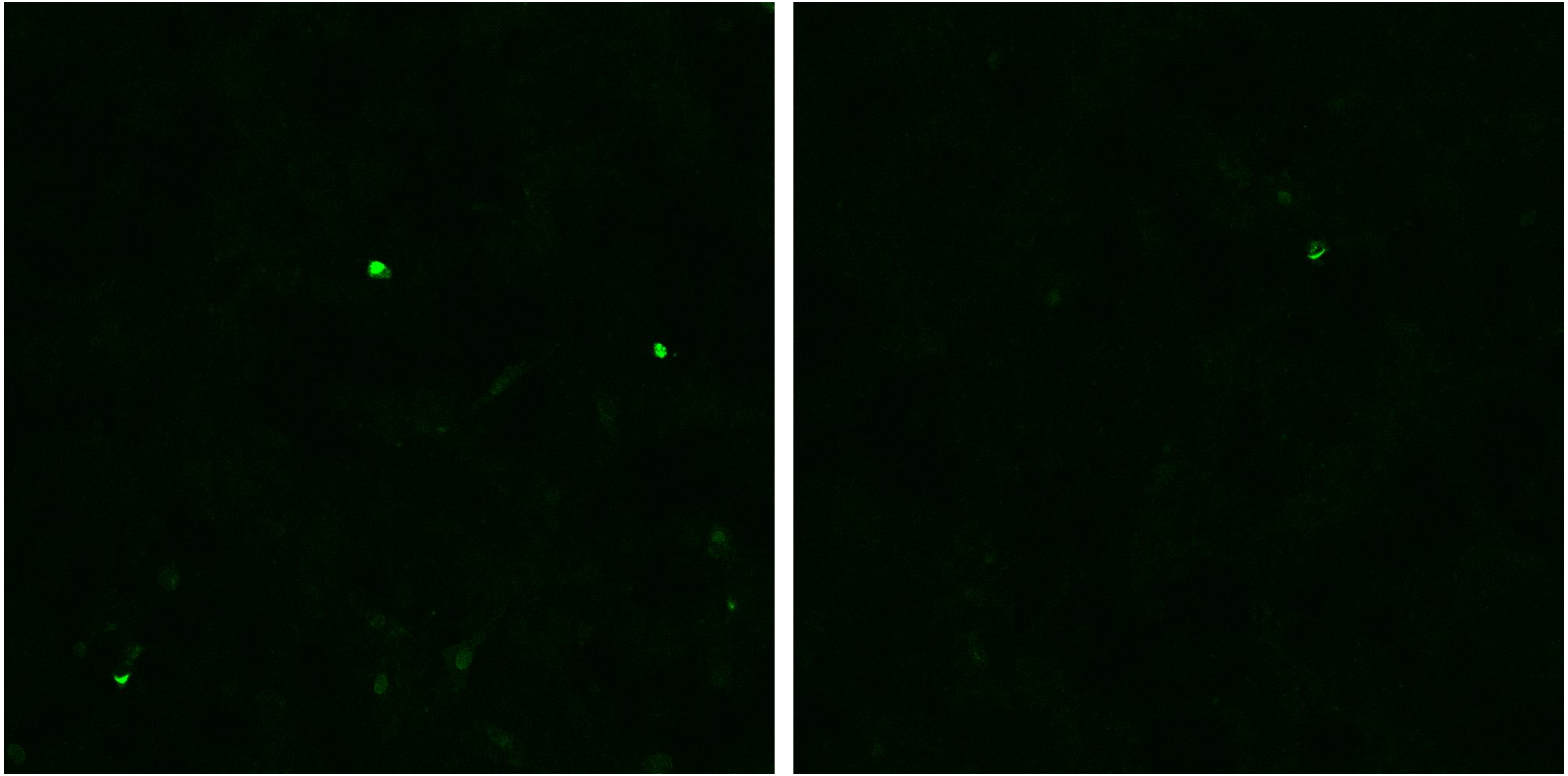

D190Y + 20 μM F5257-0462

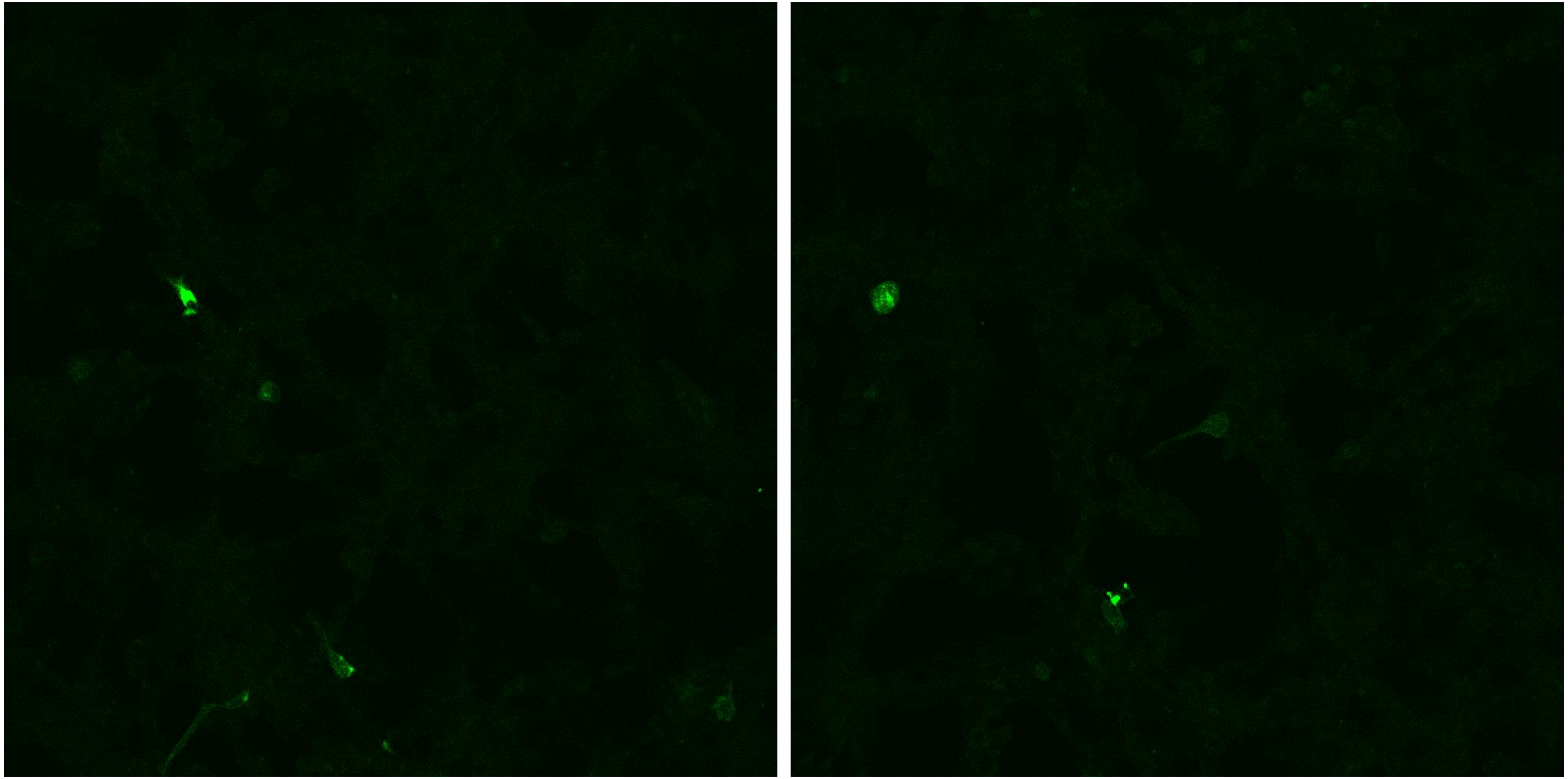

T193M + DMSO (0.1%)

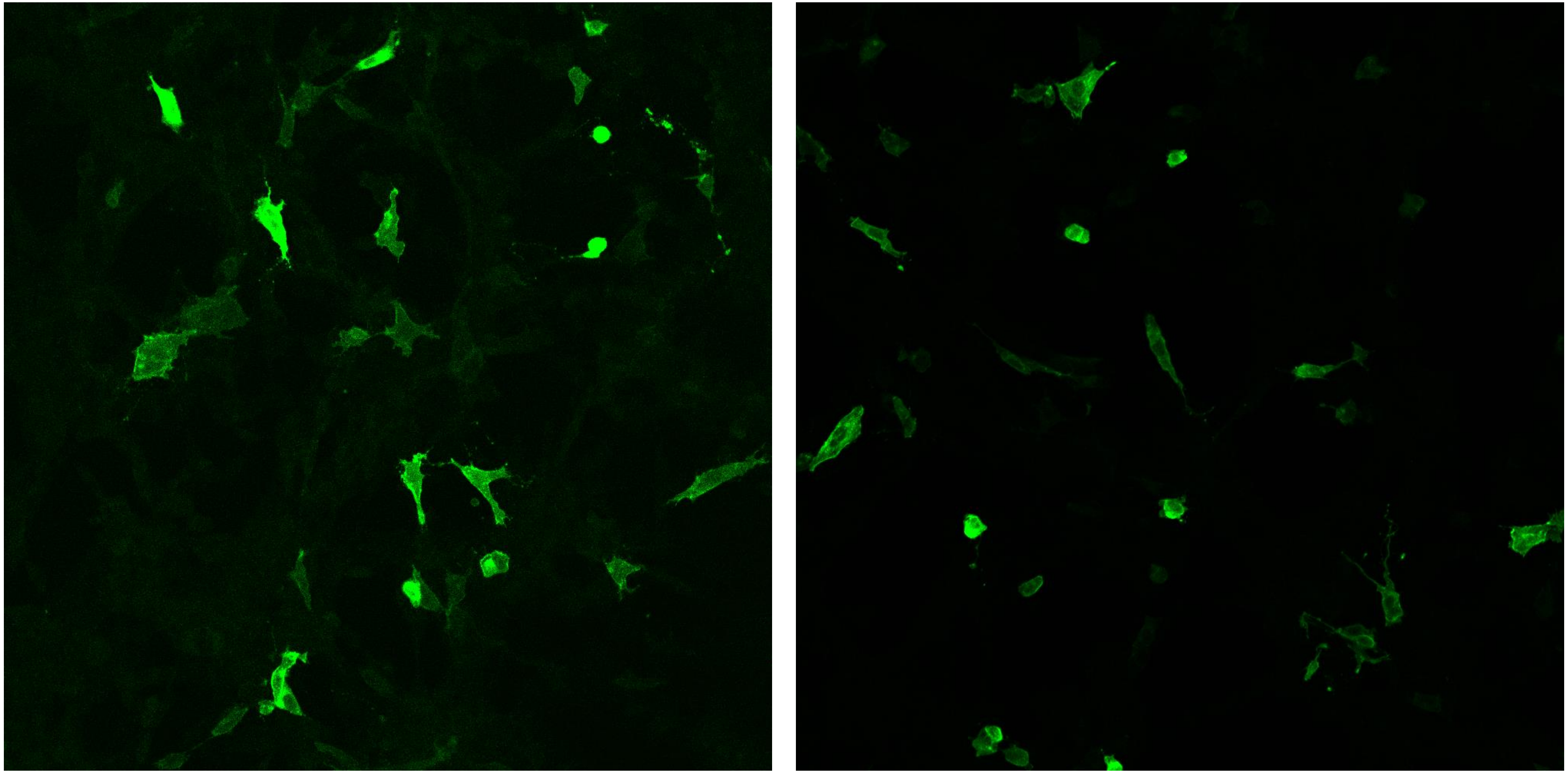

T193M + DMSO (0.1%)

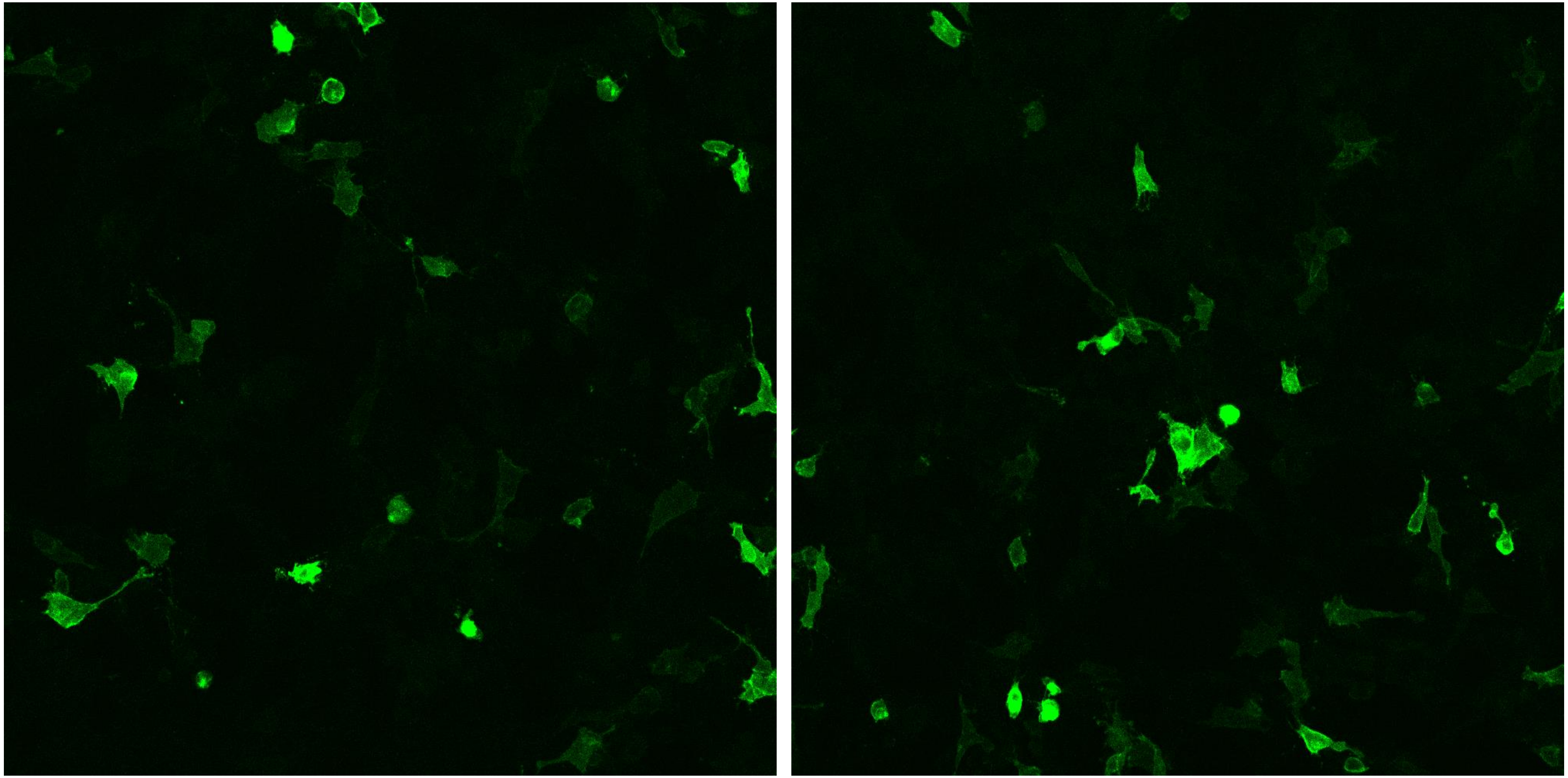

T193M + 5 μM 9-*cis*-retinal

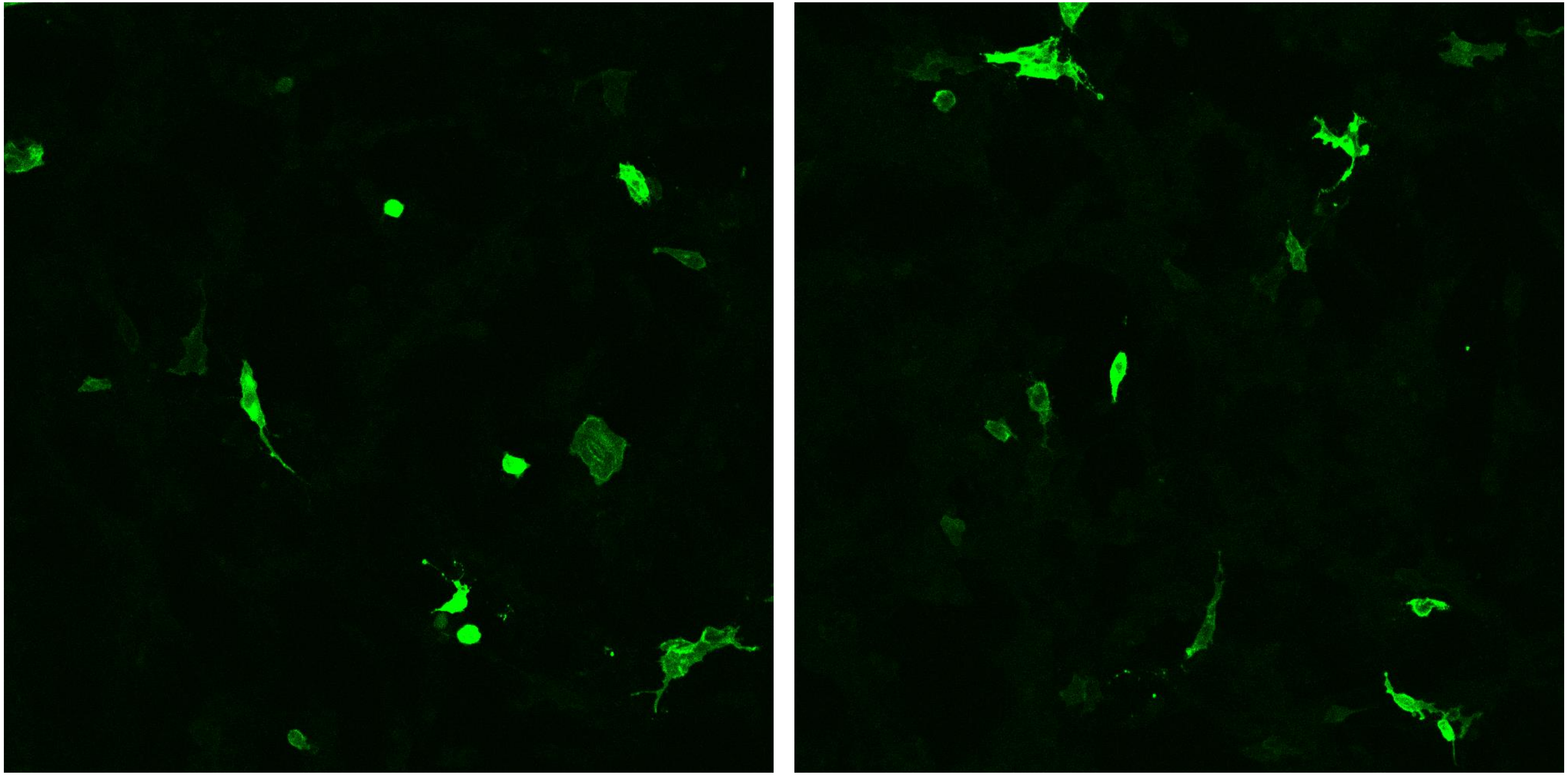

T193M + 40 μM YC-001

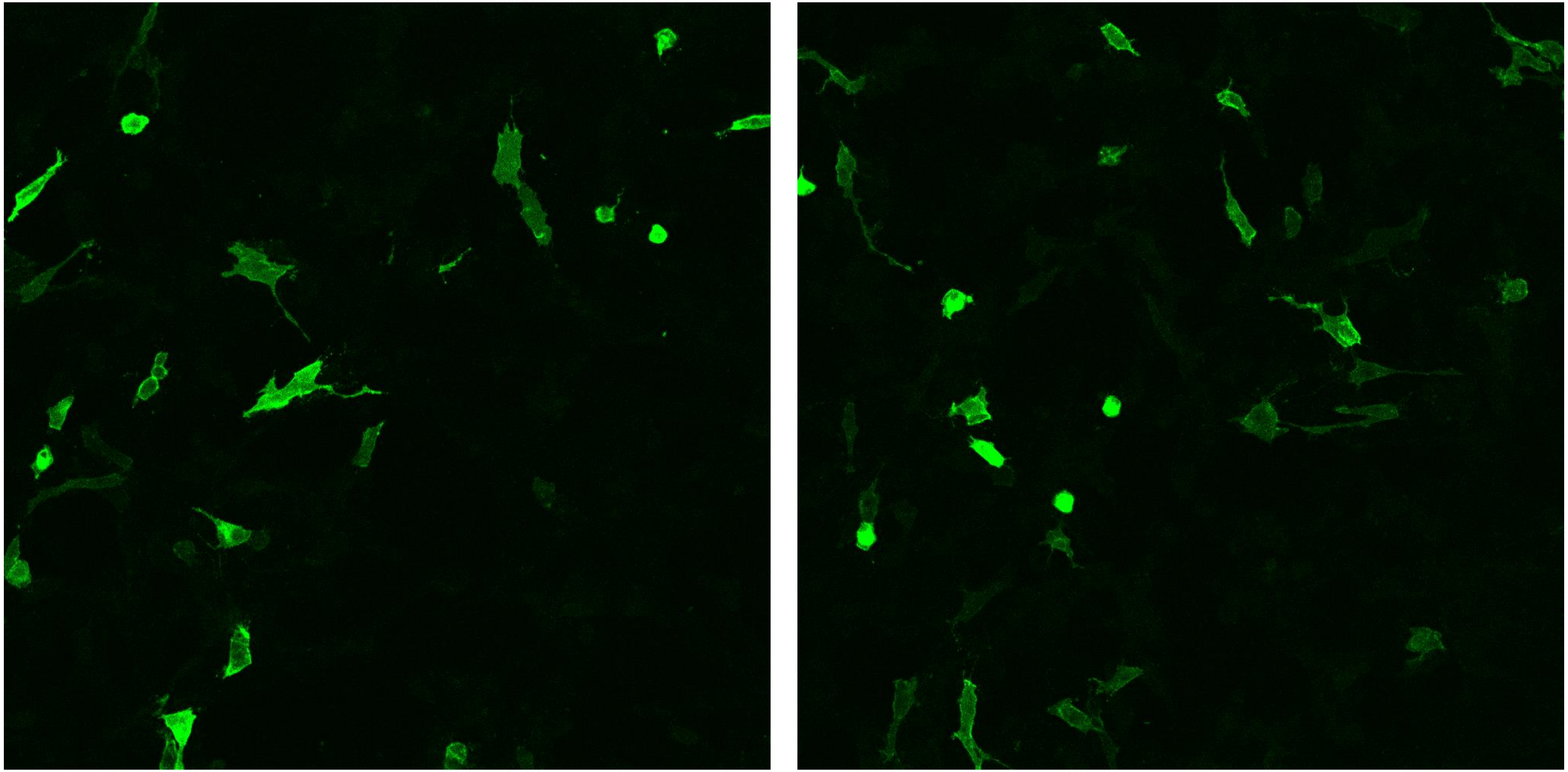

T193M + 20 μM F5257-0462

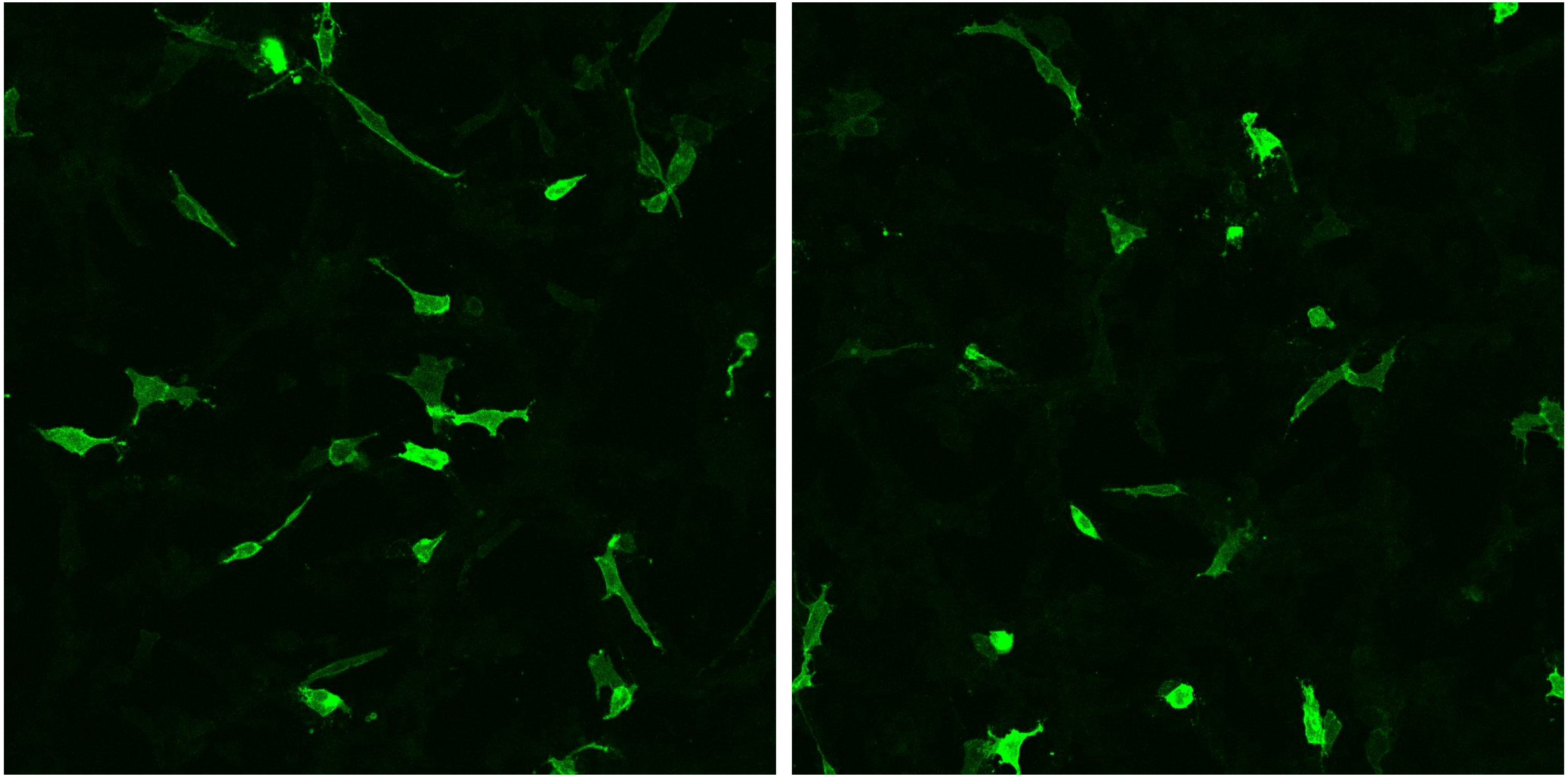

P267L + DMSO (0.1%)

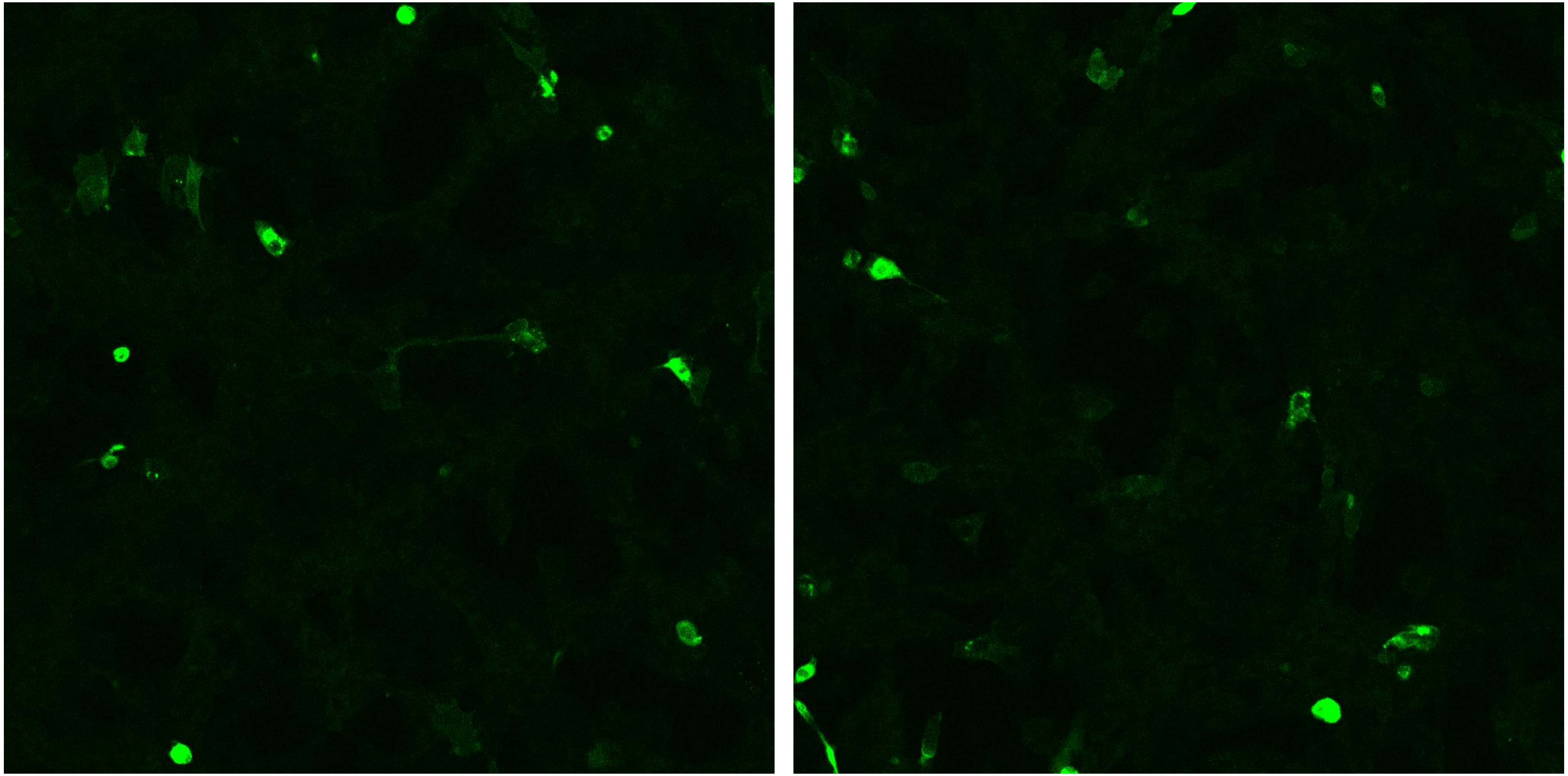

P267L + DMSO (0.1%)

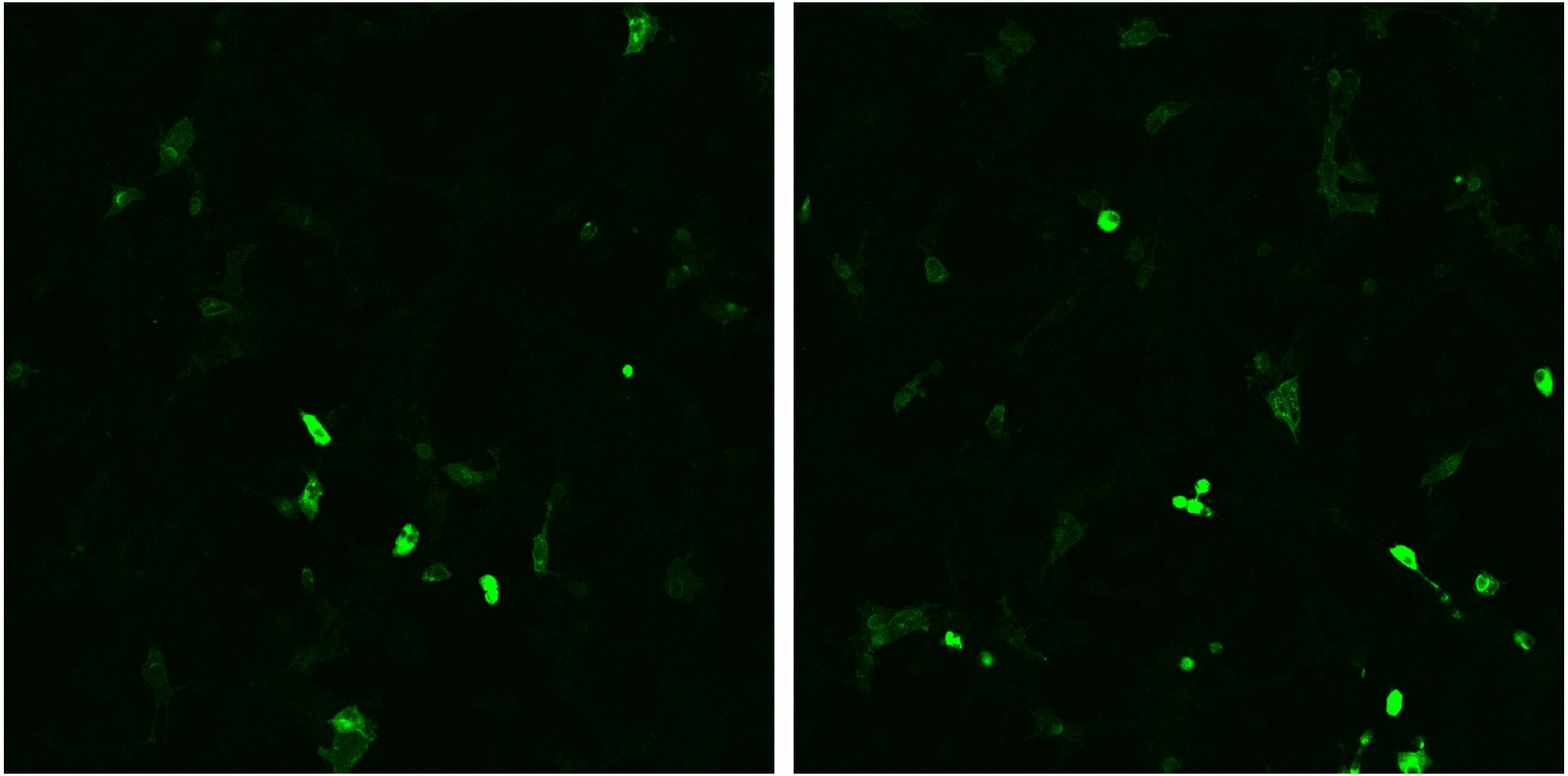

P267L + 5 μM 9-*cis*-retinal

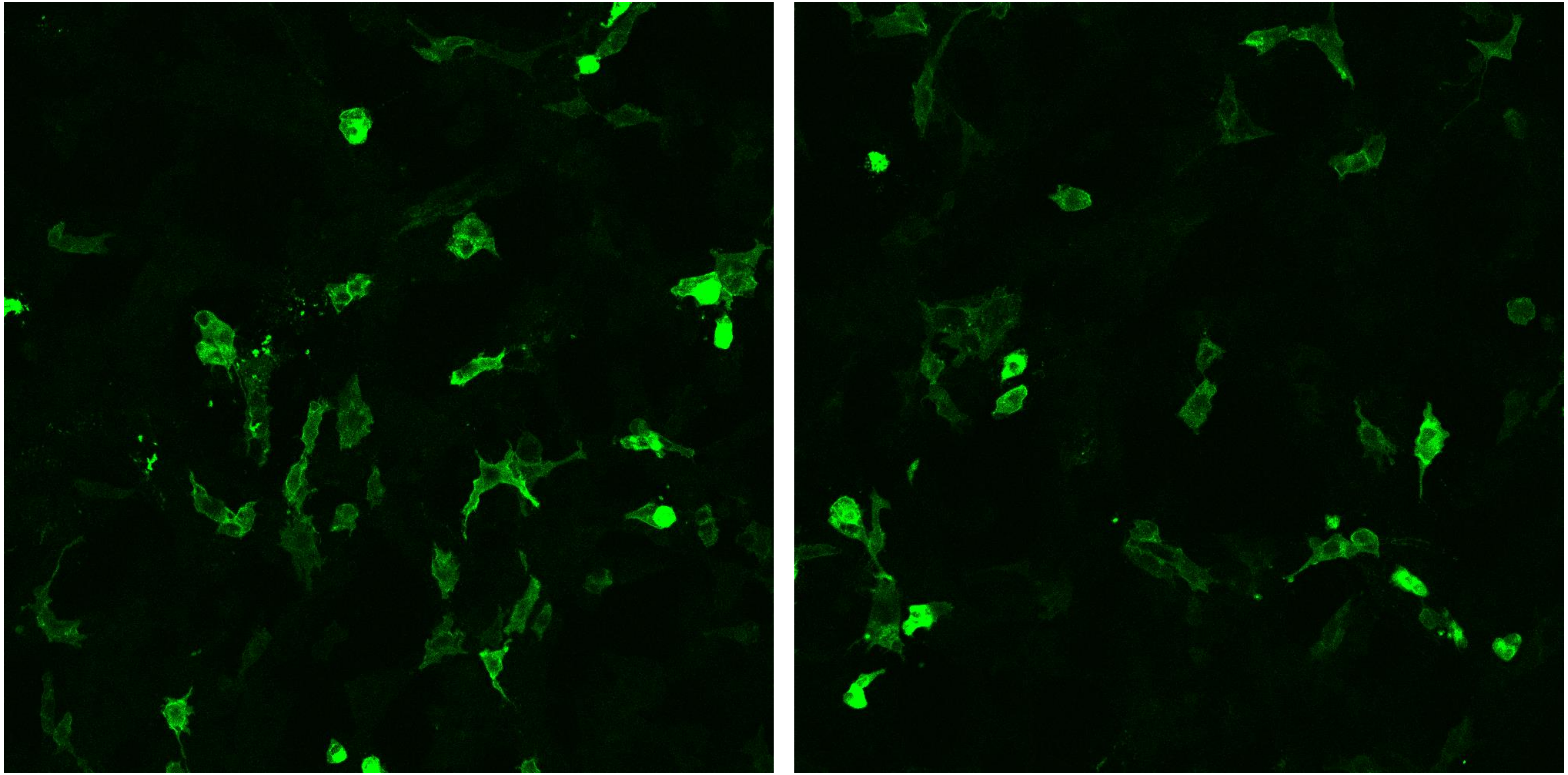

P267L + 40 μM YC-001

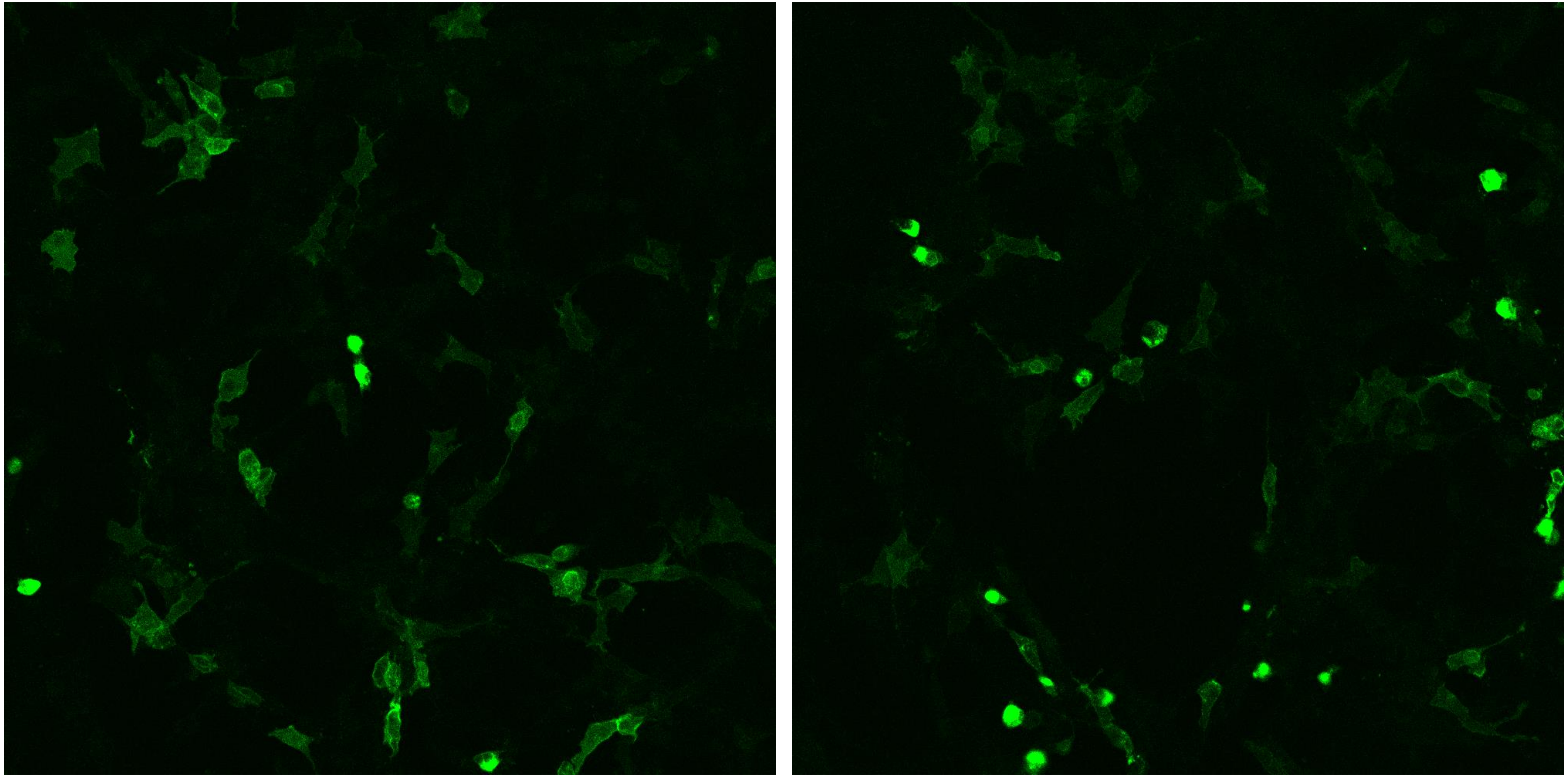

P267L + 20 μM F5257-0462

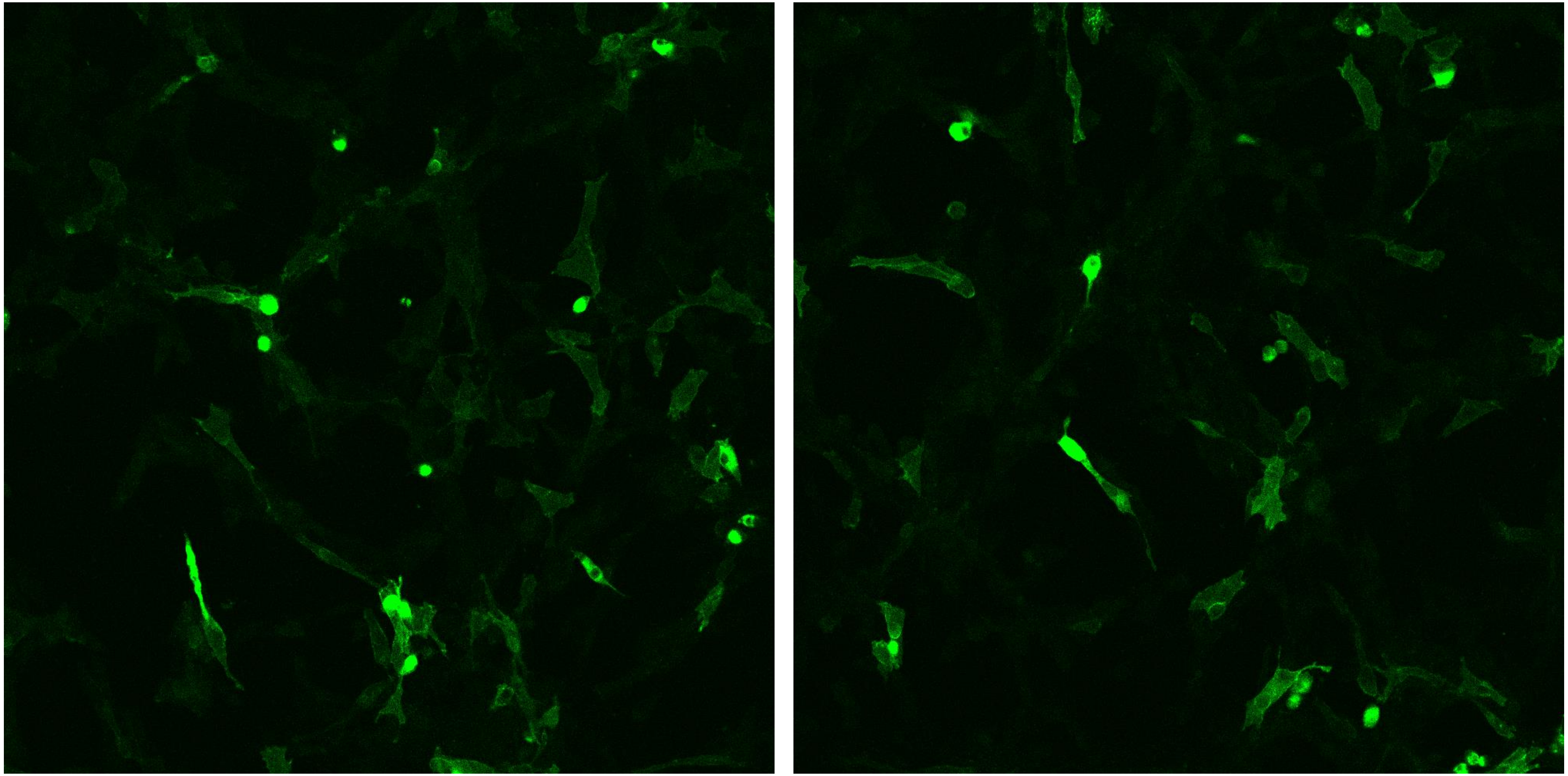

G284S + DMSO (0.1%)

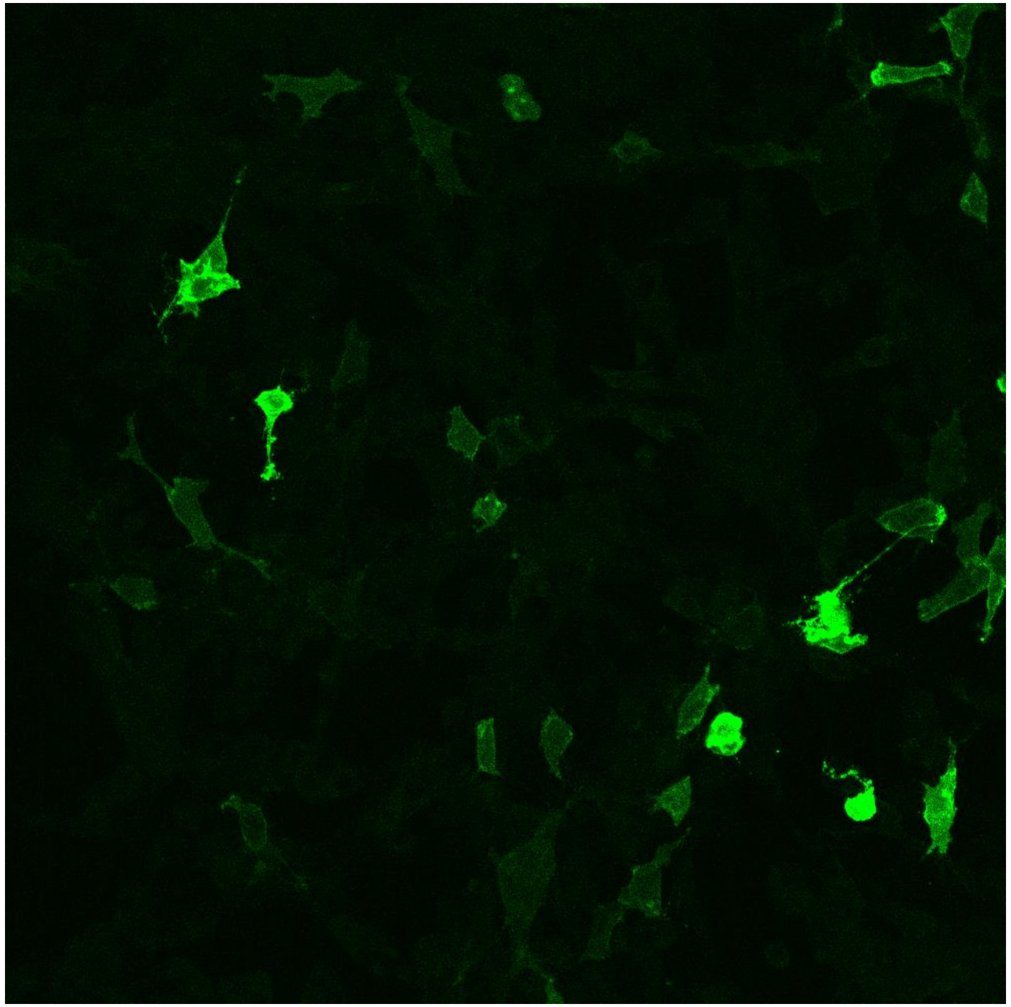

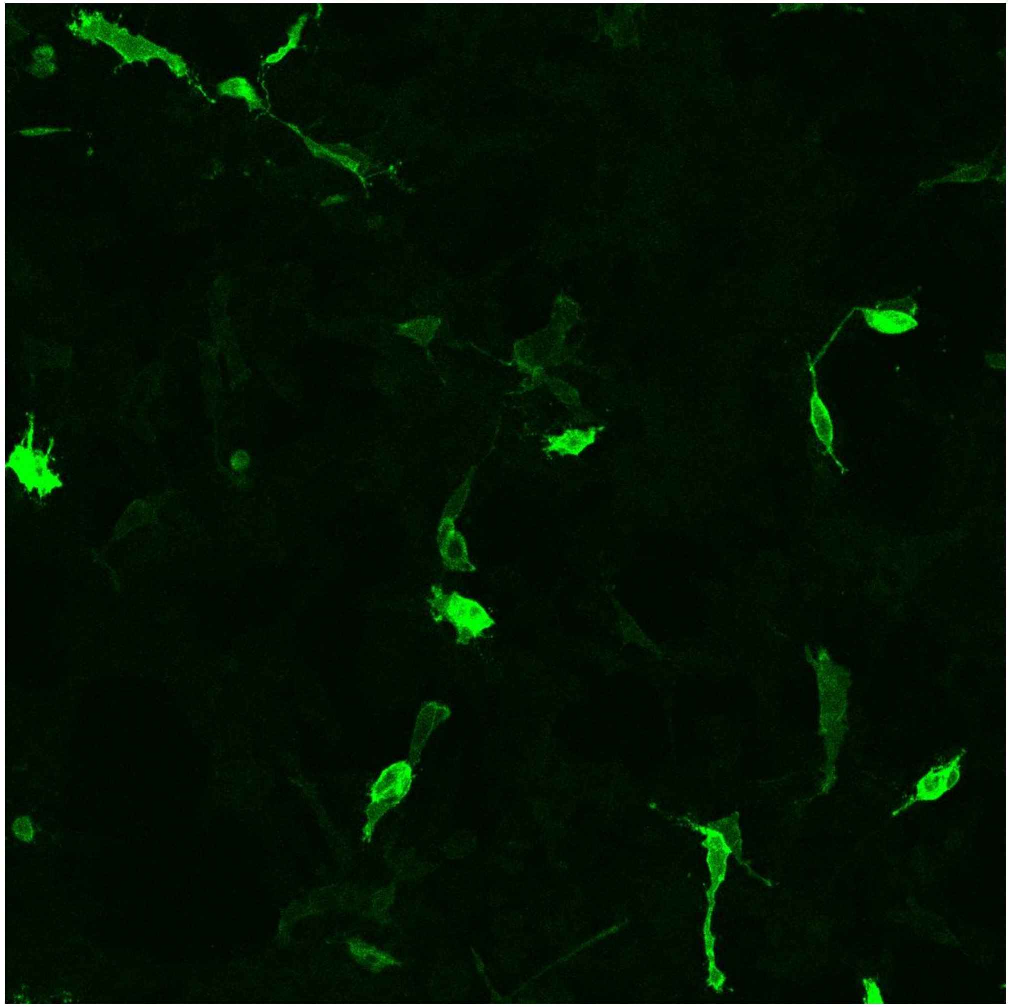

G284S + DMSO (0.1%)

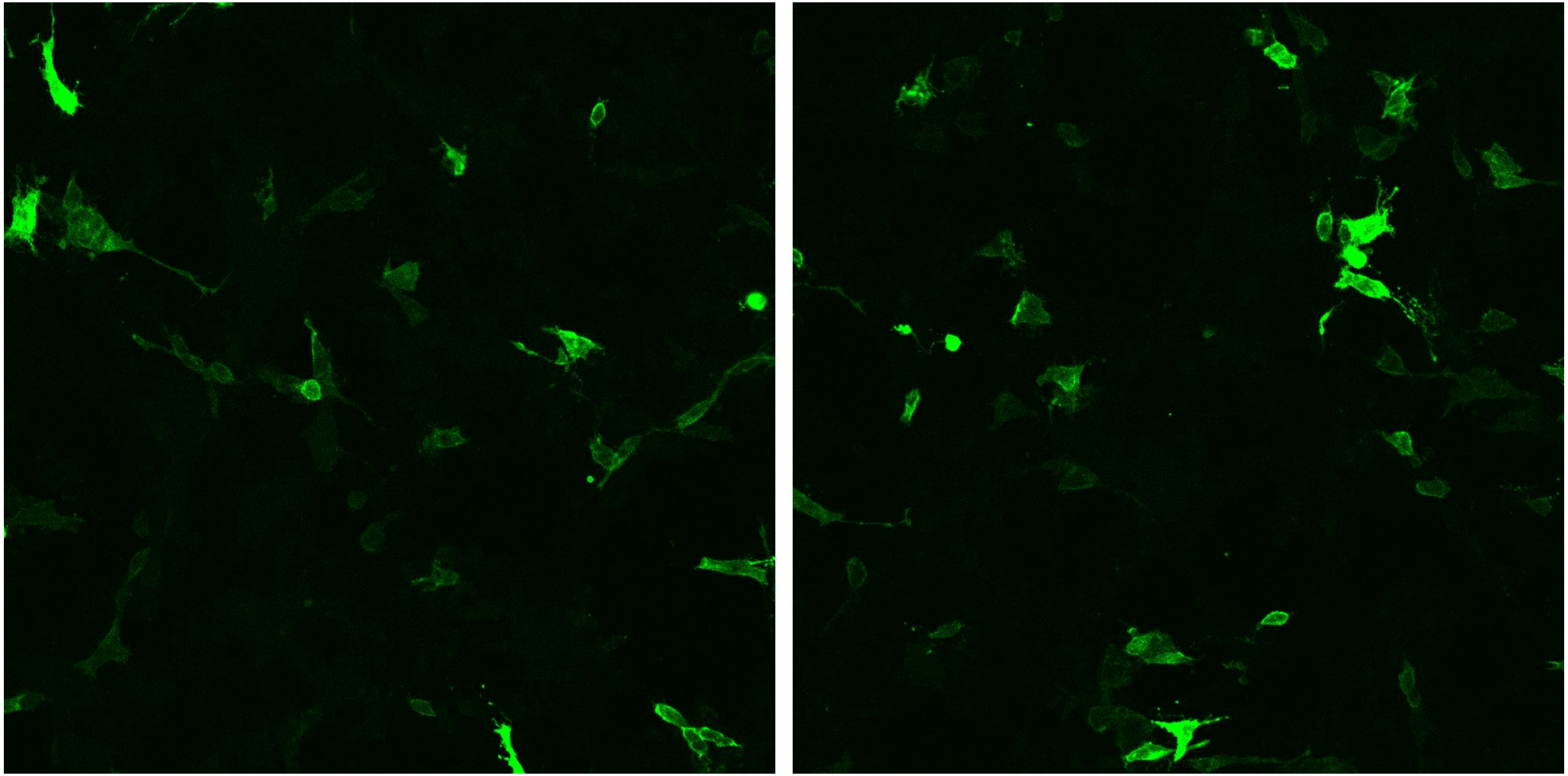

G284S + 5 µM 9-*cis*-retinal

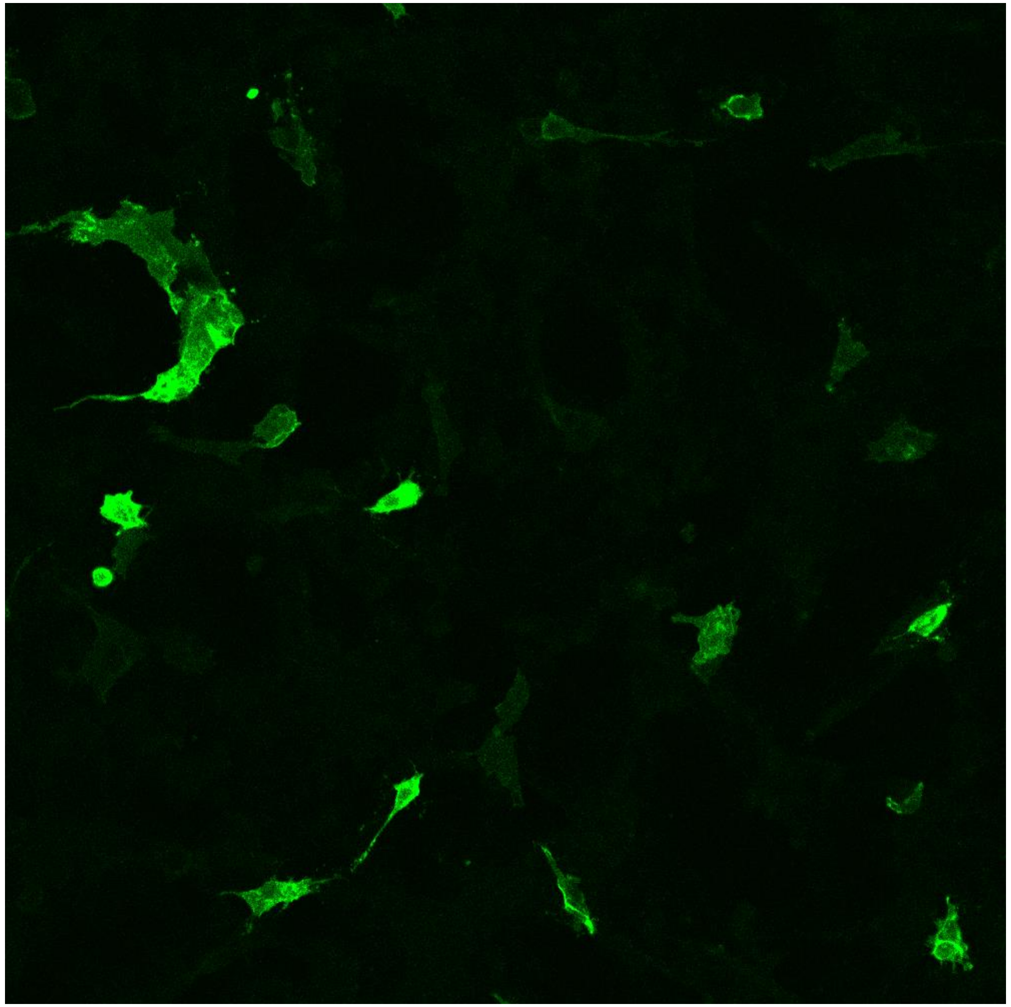

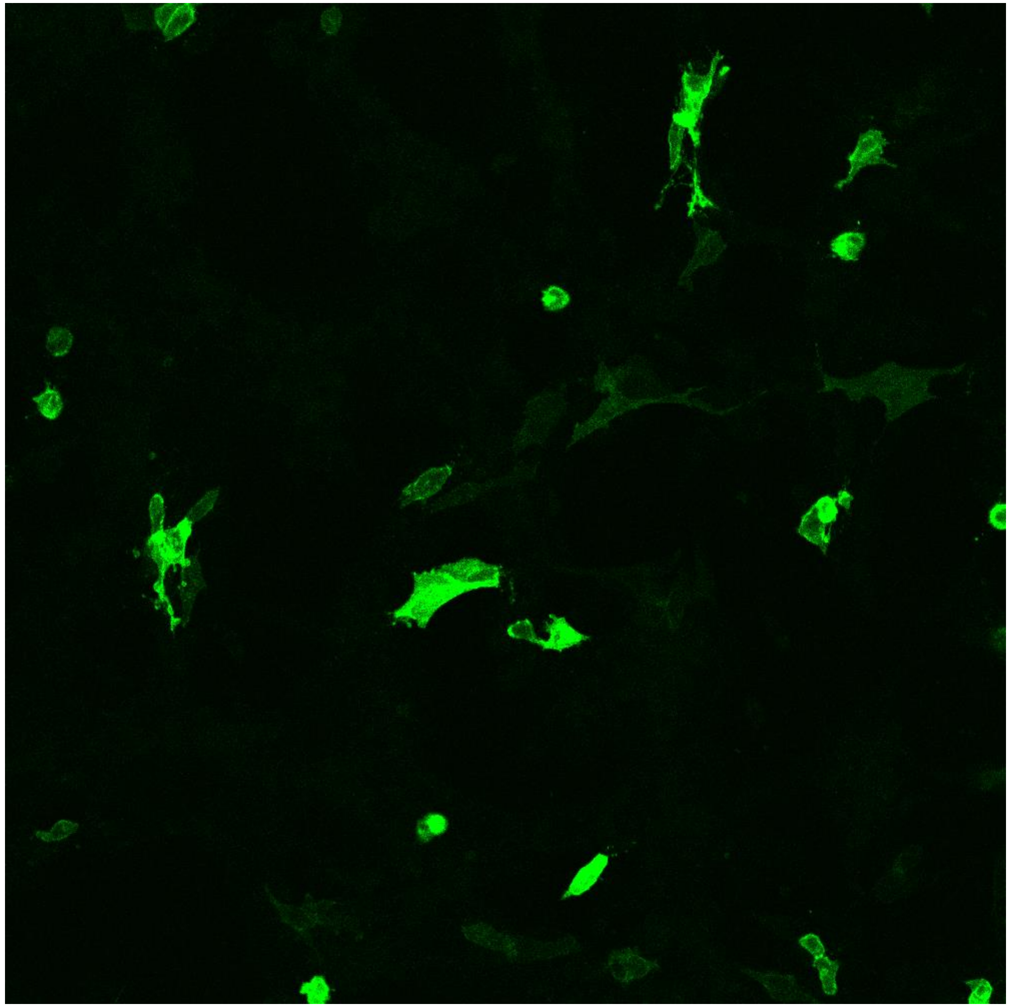

G284S + 40 μM YC-001

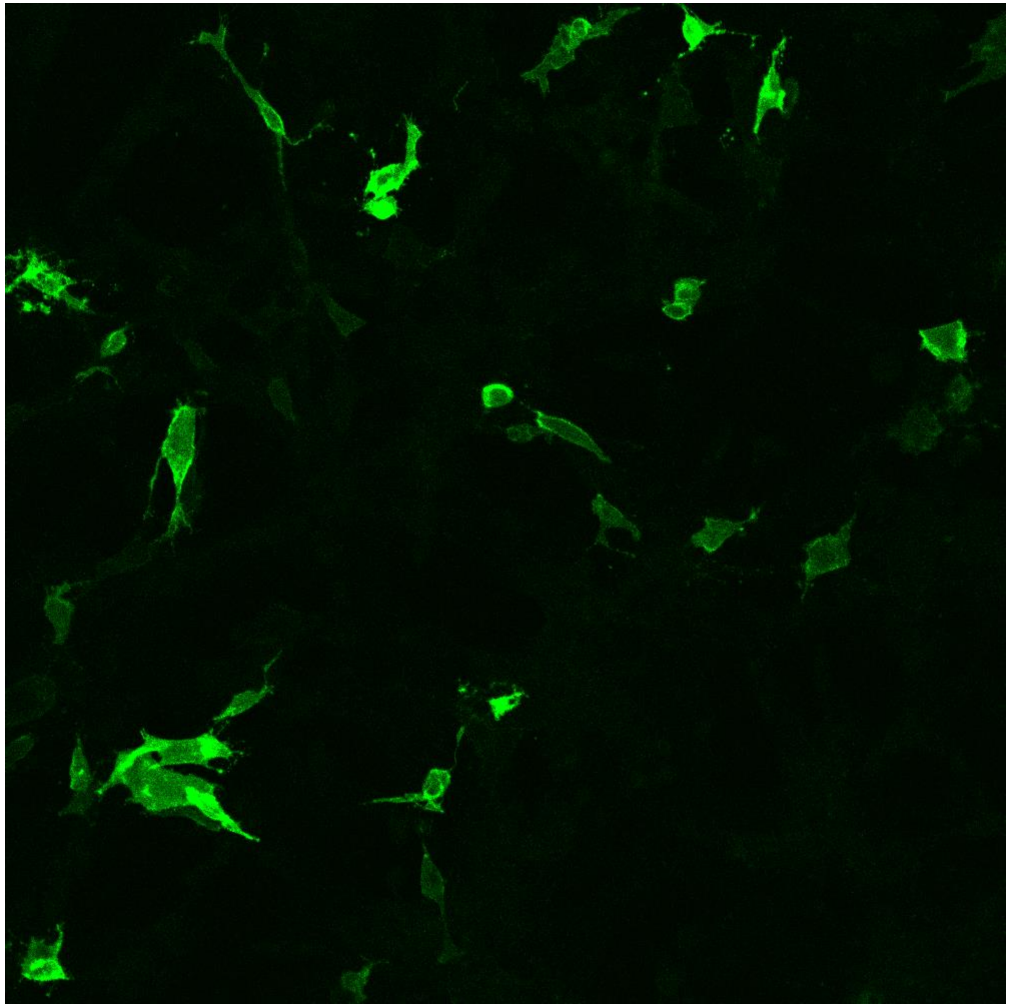

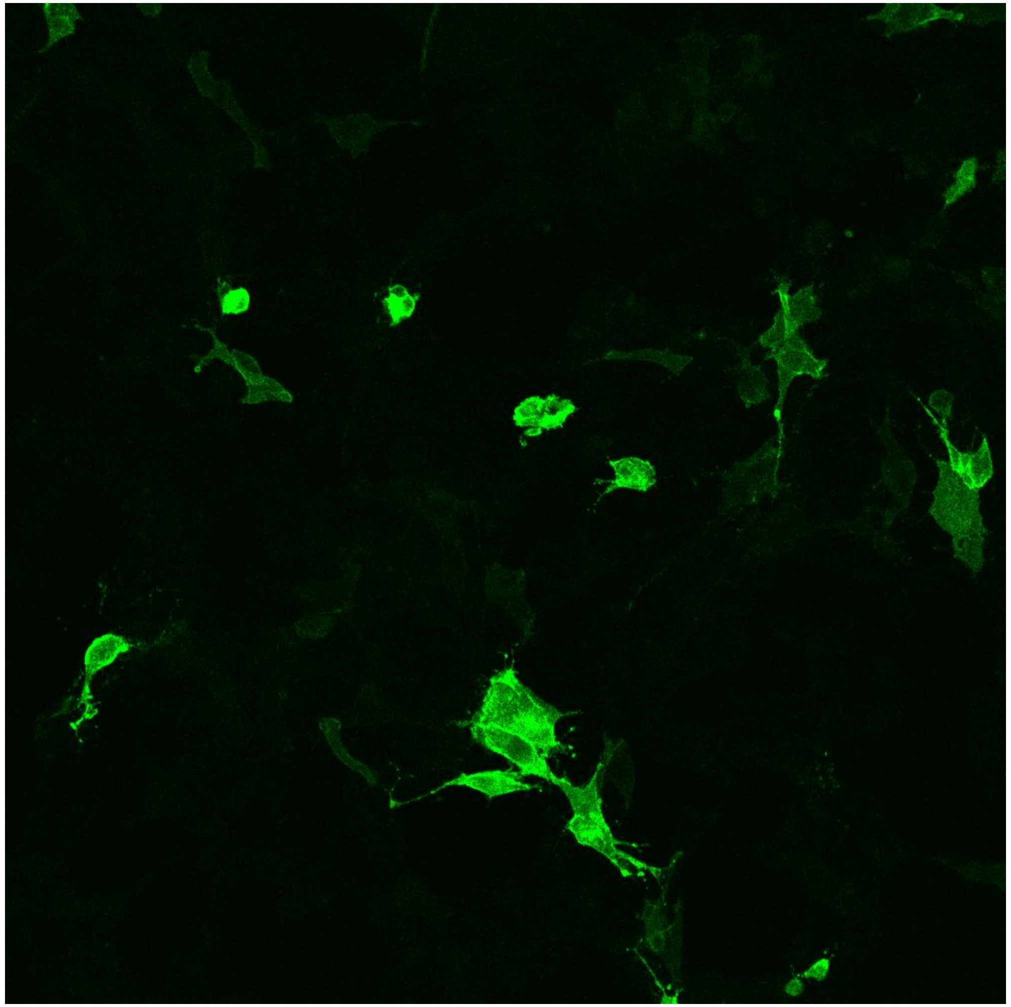

G284S + 20 μM F5257-0462

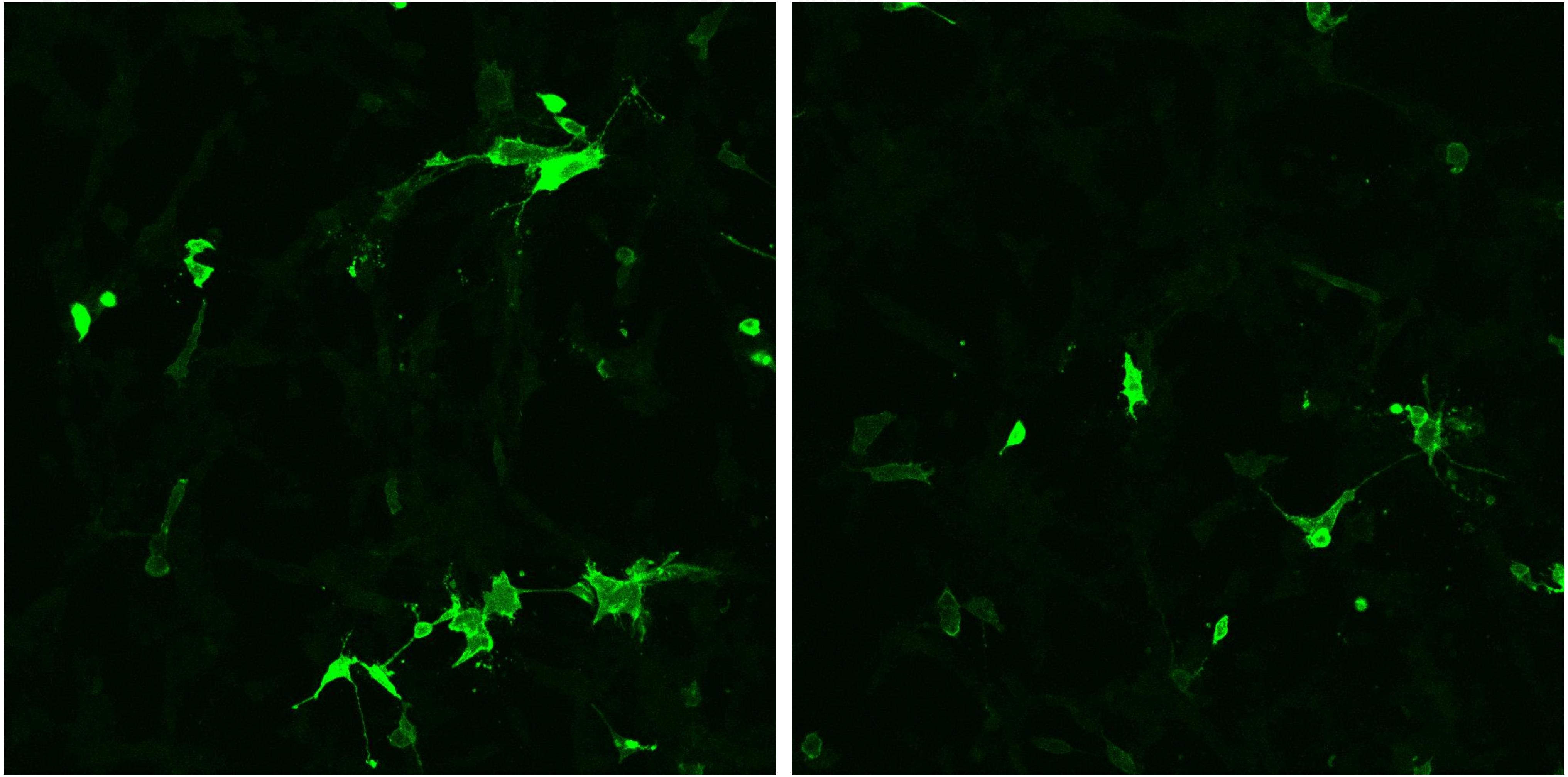

Supplement: Supplemental data [file jciinsight-7-153717-s267.pdf]
